# Investigating the shared genetic architecture between hypothyroidism and rheumatoid arthritis

**DOI:** 10.3389/fimmu.2023.1286491

**Published:** 2024-01-25

**Authors:** Zhifang Peng, Weiping Huang, Mengjun Tang, Binbin Chen, Renqi Yang, Qing Liu, Chaoshui Liu, Panpan Long

**Affiliations:** ^1^ Center of Genetics, Changsha Jiangwan Maternity Hospital, Changsha, Hunan, China; ^2^ Teaching and Research Section of Clinical Nursing, Xiangya Hospital of Central South University, Changsha, China; ^3^ Department of Orthopedics, The 967th Hospital of Joint Logistic Support Force of People's Liberation Army, Dalian, China; ^4^ Hunan Provincial Key Laboratory of the Research and Development of Novel Pharmaceutical Preparations, the “Double-First Class” Application Characteristic Discipline of Hunan (Pharmaceutical Science), Changsha Medical University, Changsha, China

**Keywords:** hypothyroidism, rheumatoid arthritis, genome-wide association studies, shared genetic architecture, shared risk genes

## Abstract

**Background:**

There is still controversy regarding the relationship between hypothyroidism and rheumatoid arthritis (RA), and there has been a dearth of studies on this association. The purpose of our study was to explore the shared genetic architecture between hypothyroidism and RA.

**Methods:**

Using public genome-wide association studies summary statistics of hypothyroidism and RA, we explored shared genetics between hypothyroidism and RA using linkage disequilibrium score regression, ρ-HESS, Pleiotropic analysis under a composite null hypothesis (PLACO), colocalization analysis, Multi-Trait Analysis of GWAS (MTAG), and transcriptome-wide association study (TWAS), and investigated causal associations using Mendelian randomization (MR).

**Results:**

We found a positive genetic association between hypothyroidism and RA, particularly in local genomic regions. Mendelian randomization analysis suggested a potential causal association of hypothyroidism with RA. Incorporating gene expression data, we observed that the genetic associations between hypothyroidism and RA were enriched in various tissues, including the spleen, lung, small intestine, adipose visceral, and blood. A comprehensive approach integrating PLACO, Bayesian colocalization analysis, MTAG, and TWAS, we successfully identified *TYK2*, *IL2RA*, and *IRF5* as shared risk genes for both hypothyroidism and RA.

**Conclusions:**

Our investigation unveiled a shared genetic architecture between these two diseases, providing novel insights into the underlying biological mechanisms and establishing a foundation for more effective interventions.

## 1 Introduction

The condition of hypothyroidism is characterized by low thyroid hormone levels and high thyroid-stimulating hormone (TSH) levels. Approximately 4% to 10% of the population is affected by hypothyroidism, and subclinical hypothyroidism has been reported in up to 10% of individuals (1), with a higher prevalence among women and the elderly (2). Hypothyroidism is associated with cardiac dysfunction, atherosclerosis, hypertension, and coagulation disorders, potentially reducing patients’ lifespans (3).

Rheumatoid arthritis (RA) is a chronic inflammatory autoimmune and autoimmune disease that affects 0.5% to 1% of the world’s population (4). It leads to inflammation and structure damage of joints, reduced mobility, and increased disability (5). Even though their precise etiology is not fully understood, these diseases are believed to be associated with an interplay between genetic predisposition and environmental influences (6–9). Despite notable advancements in RA treatment, patients experience less favorable outcomes concerning quality of life, morbidity, and mortality than the general population (10).

Previous studies have demonstrated an elevated rate of hypothyroidism in RA patients compared to controls (11). Recently, Gao Y et al. provided compelling evidence supporting a causal association between hypothyroidism and increased risk of RA (12). In a cohort study, there was no significant difference in the prevalence of hypothyroidism between RA patients and healthy adults (13). However, RA and hypothyroidism have not always been associated consistently in the literature, and there has been a dearth of studies on this association.

**Figure 1:**
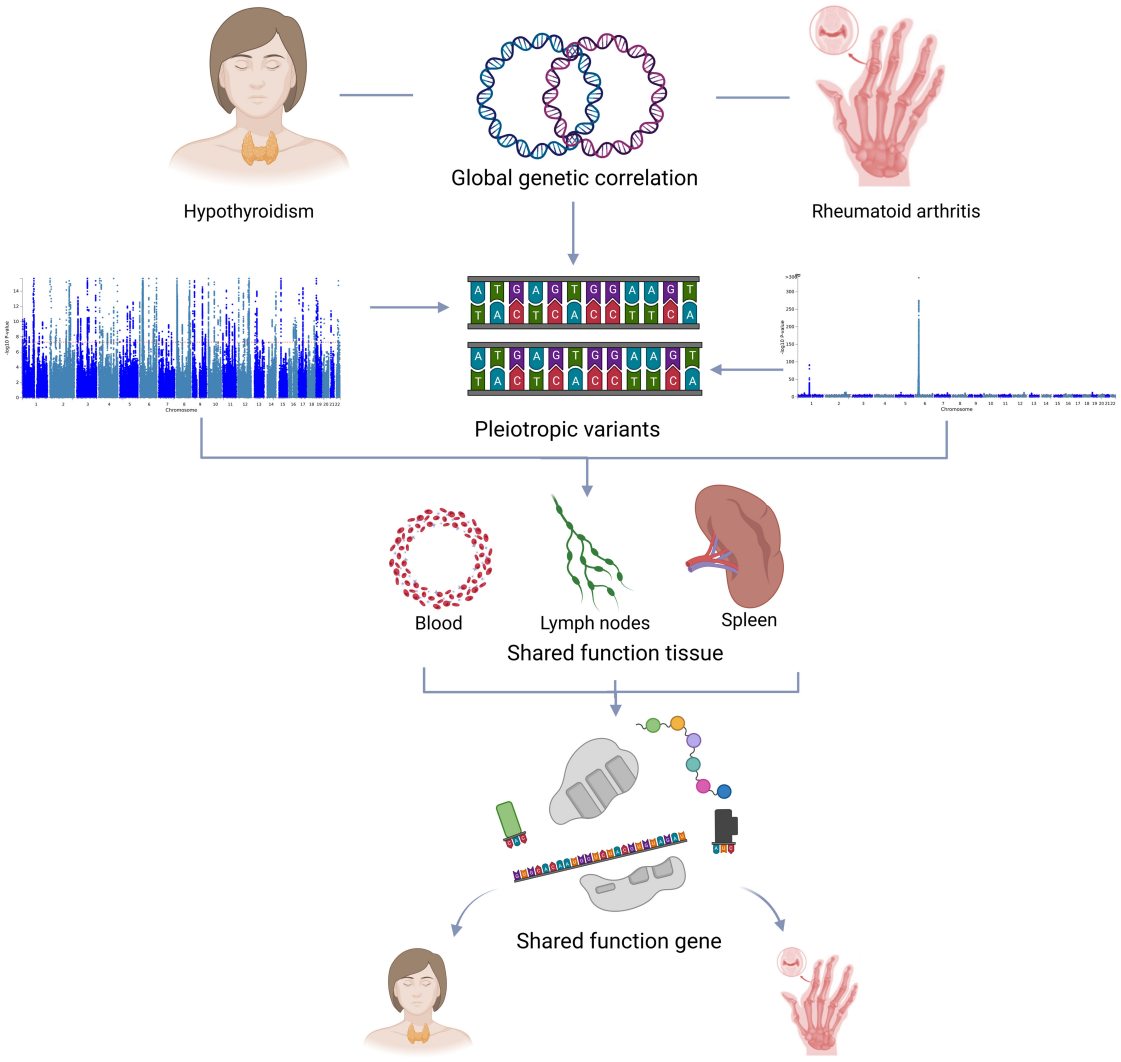
Overall study design.

There is an elusive relationship between hypothyroidism and RA. Our study aims to clarify the shared genetic architecture and molecular pathways of hypothyroidism and RA, providing new information on their underlying biological mechanisms and paving the way for more effective interventions. **Figure 1** illustrates the overall study design.

## 2 Methods

### 2.1 Data source

We used genome-wide association studies (GWAS) summary statistics for hypothyroidism involving 462,933 individuals (22,687 cases and 440,246 controls) of European ancestry from the GWAS Catalog (https://www.ebi.ac.uk/gwas/). For RA, we applied summary statistics for the discovery dataset from GWAS with 253,417 individuals (12,555 cases and 240,862 controls) from the FinnGen Consortium (https://r9.finngen.fi/) (14). To corroborate the findings from the initial discovery dataset, a replication GWAS of RA was performed. This replication involved 58,284 individuals, comprising 14,361 RA cases and 43,923 controls (15). **Supplementary Table S1** presents a comprehensive description of the characteristics of each dataset utilized in our study. To mitigate the potential bias of ethnic diversity, we focused our investigation on individuals of European ancestry. Ethical clearance was not required, as the study relied on publicly available data.

### 2.2 Linkage disequilibrium score regression

Heritability estimates for hypothyroidism and RA were derived using LDSC in Python 2.7 (16). Additionally, we calculated the genetic correlation (r_g_) between the two diseases, quantifying the shared genetic variance relative to the square root of their respective Single Nucleotide Polymorphism (SNP) heritability estimates. The analysis utilized the 1000 Genomes European Reference dataset to convert GWAS summary statistics into precalculated linkage disequilibrium (LD) scores. Sensitivity analysis was conducted with a single-trait heritability intercept constraint.

### 2.3 ρ-HESS

We used the ρ-HESS approach to investigate the local genetic correlations between hypothyroidism and RA (17), which estimates the local heritability of SNPs and genetic covariance based on genomic references constructed from genomes. We could estimate local genetic correlations by calculating local single-tit SNP heritability and local cross-tit genetic correlation. The algorithm was used to calculate the genome into 1,703 regions and quantifies trait correlations attributable to genetic variation within specific regions.

### 2.4 Pleiotropic analysis under composite null hypothesis

Identification of potentially pleiotropic single nucleotide variants (SNVs) using genotype-phenotype association statistics at the aggregation level is a novel approach PLACO employs to investigate pleiotropic loci associated with complex traits (18). Significant pleiotropic variants were defined as single-nucleotide variants with *P*-values less than *P* < 5.0×10^−8^ for PLACO. Subsequently, to identify common causal variants for each pleiotropic locus, the Functional Mapping and Annotation of Genetic Associations (FUMA) tool was used to identify potential pleiotropic loci, followed by Bayesian colocalization analysis (19, 20).

### 2.5 Cross-trait meta-analysis

We employed the Multi-Trait Analysis of GWAS (MTAG) (21) in Python 2.7 to identify risk SNPs associated with hypothyroidism and RA. MTAG allows for joint analysis of GWAS summary statistics for different traits, considering potential sample overlap between GWAS. MTAG presumes that effect sizes across traits have a shared variance-covariance matrix (21). Finally, SNPs associated with joint phenotypes were identified if they exhibited independent associations (LD r^2^<0.001) with both diseases (*P*-value<5.0×10^-8^) in MTAG.

### 2.6 Functional analysis for pleiotropic genes

PLACO was used to identify pleiotropic genes and subsequently subjected to differential expression and gene set enrichment analyses using FUMA (19). Genotype-tissue expression (GTEx) gene expression data from 53 tissues were used in this investigation. Expression normalization was performed as a preliminary step, followed by a two-sided Student’s t-test to compare each gene in a given tissue with all other genes. It identified differentially expressed genes (DEGs) specific to each tissue type based on genes with a Bonferroni corrected *P*-value of less than 0.05 and an absolute log-fold change of 0.58. These results indicate significant differences in gene expression levels compared to other tissues (22–25).

### 2.7 Pathway-based functional enrichment analysis

The Kyoto Encyclopedia of Genes and Genomes (KEGG) Orthology-Based Annotation System version 3.0 online bioinformatics database was used to conduct KEGG pathway enrichment analysis and Gene Ontology (GO) analysis to gain insights into the biological mechanisms associated with hypothyroidism and RA (26).

### 2.8 Mendelian randomization analysis

We employed MR analysis, a widely used instrumental variable approach for causal inference, to establish causal relationships between hypothyroidism and RA. Exposure-related SNPs served as instruments (27, 28), and data from the GWAS summary were used to identify variants associated with hypothyroidism and RA at *P*-value<5.0×10^-8^. The inverse-variance weighted (IVW) approach was used as a primary method, with LD and physical distance thresholds of 0.001 and 10 MB, respectively, Utilizing the European ancestry reference panel from the 1000 Genomes Project.

### 2.9 Transcriptome-wide association studies

FUSION software was used to analyze tissue-related TWAS data from the GWAS summary data (29). An analysis of TWAS used pre-computed gene expression weights in conjunction with GWAS summary statistics to determine the associations between genes and diseases. Cortical RNA sequence reference panels from the GTEx Consortium were integrated with GWAS summary statistics of GWAS for TWAS (30).

## 3 Results

### 3.1 Global genetic correlation

Based on the baseline LD model and stratification LDSC, we estimated the liability-scale SNP heritability of hypothyroidism and RA (31, 32). Bivariate LDSC methodology was used to estimate the genetic correlations between the two diseases. The analysis revealed a significant positive genetic correlation (r_g_ = 0.31, *P* = 6.70×10^-7^) between hypothyroidism and RA (discovery) (**Supplementary Table S2**). The replication aimed to validate the initial observations. Notably, a significant positive genetic correlation was observed between hypothyroidism and RA (replication) (r_g_ = 0.35, *P* = 1.13×10^-10^), supporting the initial findings. Sensitivity analyses were conducted based on LDSC, with the heritability intercept of a single trait constrained. These results revealed significant genetic associations between hypothyroidism and RA.

### 3.2 Local genetic correlations

**Figure 2:**
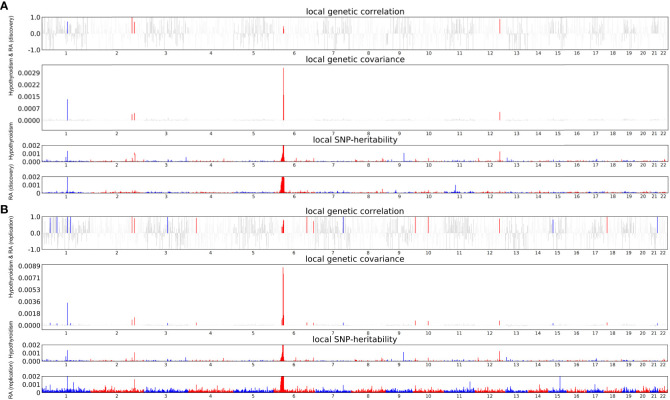
Local Genetic Correlation Between Hypothyroidism and RA. **(A)** Local Genetic Correlation Between Hypothyroidism and RA (discovery). **(B)** Local Genetic Correlation Between Hypothyroidism and RA (replication). The Manhattan plot illustrates the estimates of local genetic correlation and local genetic covariance between hypothyroidism and RA. It also displays the local SNP heritability of hypothyroidism and RA. The colored bars in the “local genetic correlation” and “local genetic covariance” sections indicate significant regions that share SNP heritability, following multiple adjustments (*P*<5.0×10^-8^ in both the local SNP heritability test and *P*<0.05/1703 in the local genetic covariance test).

Considering the significant global genetic correlation between hypothyroidism and RA, we looked for specific genomic regions with local genetic correlations. Following multiple tests of correction (*P* < 0.05/1703), strong local correlations were found in 6 different regions, with 6p21.32-21.33 (chr6: 31571218-32682664) showing the most significant correlation between hypothyroidism and RA (discovery) (*P* = 3.63×10^-52^) (**Figure 2A**; **Supplementary Table S3**). Additionally, to further confirm the results of the discovery dataset, twenty-two distinct regions exhibited strong local correlations between hypothyroidism and RA (replication) (**Figure 2B**; **Supplementary Table S4**).

### 3.3 Shared loci between hypothyroidism and RA

**Figure 3:**
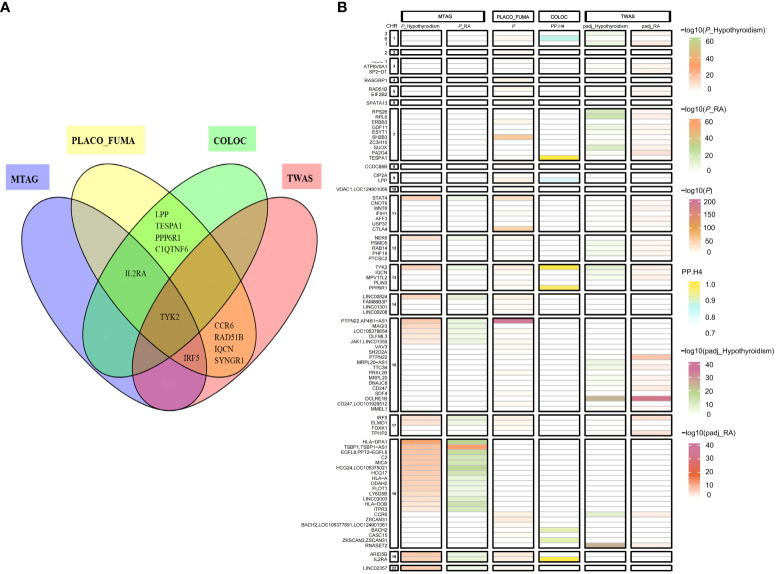
Venn diagrams and heat maps showing shared risk genes in hypothyroidism and RA (discovery) using four methods. **(A)** Venn Diagrams Showing Shared Risk Genes in Hypothyroidism and RA (discovery) Using Four Methods. **(B)** Heat Maps Showing Shared Risk Genes in Hypothyroidism and RA (discovery) Using Four Methods, with the y -axis presenting the gene, the x-axis presenting the method, and and the change in color being based on the -log (*P*-value) or PP.H4 affinities. PLACO_FUMA, Pleiotropic analysis under composite null hypothesis_Functional Mapping and Annotation of Genetic Associations; MTAG, Multi-Trait Analysis of GWAS; COLOC, Bayesian colocalization analysis; TWAS, transcriptome-wide association study.

PLACO identified 805 single nucleotide variants with potential pleiotropic effects (**Supplementary Table S5**). MAGMA analysis yielded 483 significant pleiotropic genes by the FUMA platform (**Supplementary Table S6**). There were 55 independent genomic risk loci identified by FUMA (**Supplementary Table S7**). Based on colocalization analysis, 9 out of 55 potential pleiotropic loci (16%) had PP.H4 higher than 0.75, candidate-shared causal variants were identified at three top SNVs of corresponding loci (**Table 1** and **Figure 3B**). A similar analysis was conducted on the RA (replication) data to validate these findings. This analysis, facilitated by FUMA, identified 66 independent genomic risk loci (**Supplementary Table S8**). Notably, loci rs34536443 (located in *TYK2*), rs706778 (located in *IL2RA*), and rs3807307 (located in *IRF5*) were included. Colocalization analysis further indicated that these specific loci (rs34536443 in *TYK2*, rs6454802 in *BACH2*, and rs229544 in *C1QTNF6*) were consistent across both discovery and replication datasets (**Supplementary Table S9**).

**Table 1:** Nine colocalized loci identified through colocalization analysis on 55 pleiotropic loci.

Top SNV	Locus boundary^a^	Region	Nearest gene	Best causal	SNP.PP.H4
rs1559810	3:188072513-188135783	3q28	*LPP*	rs13319297	0.819
rs2393923;rs57252182;rs72854533;rs9468356;rs112886535;rs3117343;rs116466121;rs116802478;rs1003582;rs362520;rs29242;rs29232	6:27262294-29611431	6p22.1	*ZKSCAN3,ZSCAN31*	rs72854535	0.925
rs383711;rs2854027;rs1704995;rs9277946;rs34859217;rs9296092;rs78075721;rs2229637;rs13219530;rs10947433;rs12194518	6:33173842-33811790	6p21.32-p21.31	*/*	rs206763	1.0
rs6908626	6:90880393-91005743	6q15	*BACH2*	rs6454802	0.922
rs706778;rs41260244	10:6071453-6182251	10p15.1	*IL2RA*	rs3118470	0.999
rs62623446	12:55358844-55368291	12q13.2	*TESPA1*	rs62623446	0.999
rs34536443	19:10427721-10586018	19p13.2	*TYK2*	rs34536443	1.0
rs73068668	19:55739048-55763262	19q13.42	*PPP6R1*	rs73068668	0.982
rs229527	22:37573712-37609342	22q13.1	*C1QTNF6*	rs229544	0.874

a Locus boundary of each pleiotropic genomic risk locus was denoted as “chromosome: start-end” defined by FUMA.

We performed MTAG to identify risk SNPs underlying hypothyroidism and RA(discovery) joint phenotypes. After excluding SNPs in linkage disequilibrium (LD r^2^≥0.001), a total of 39 shared independent SNPs with genome-wide significance were found (**Figure 3B** and **Supplementary Table S10**). Furthermore, the MTAG of hypothyroidism and RA (replication) dataset revealed 53 shared independent SNPs reaching genome-wide significance (**Supplementary Table S11**). Of these, 12 loci were concurrently identified in both the RA (discovery) and RA (replication) datasets, including SNPs rs11085727 (mapped on *TYK2*), rs3118470 (mapped on *IL2RA*), and rs3807307 (mapped on *IRF5*).

### 3.4 Mendelian randomization

A bidirectional MR study was conducted to investigate the potential causal relationship between hypothyroidism and RA (discovery). All SNPs were strong instruments in the Mendelian randomization analysis (F > 10). Various bi-directional MR methods were employed to ensure result stability. Our findings further validated the causal effect of hypothyroidism on RA (discovery) (**Supplementary Table S12**), with estimates remaining directionally consistent across the weighted median, weighted mode, and MR Egger approaches. However, no significant effect of RA (discovery) on hypothyroidism was observed (**Supplementary Table S8**). Moreover, our findings provided validation of the causal effect of hypothyroidism on RA (replication) (**Supplementary Table S13**).

### 3.5 Enrichment analysis for identified pleiotropic genes

**Figure 4:**
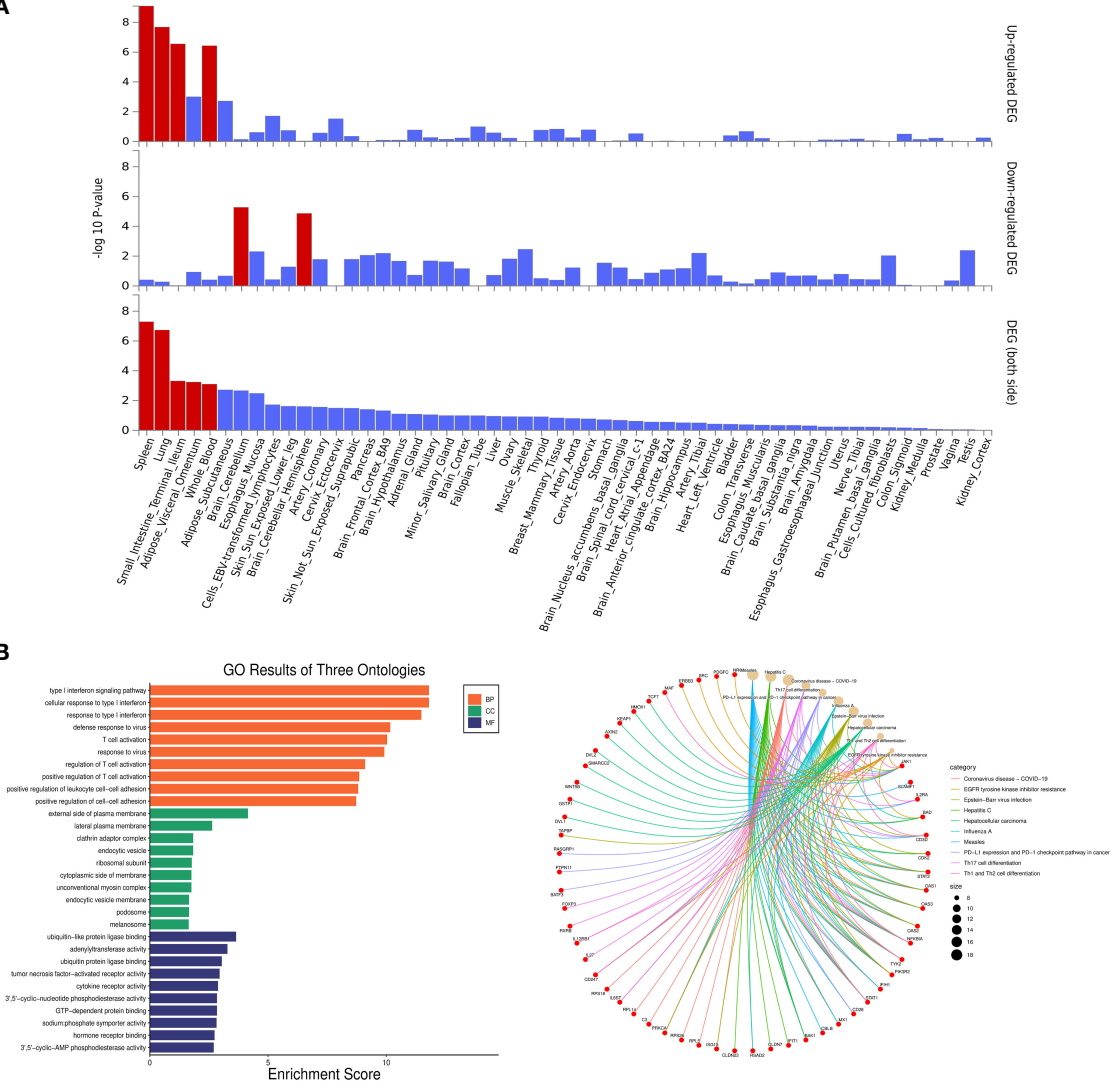
Enrichment analysis for identified pleiotropic genes. **(A)** Enrichment of Differentially Expressed Genes Among All Identified Pleiotropic Genes Across 54 GTEx Tissues. The y-axis shows the *P*-values with a scale of − log10. The bars in red represent significant enrichment with Bonferroni adjustment for multiple hypothesis testing. **(B)** Top 10 Significant Types of Pathways Based on GO and KEGG Enrichment Analyses. BP, Biological Process; CC, Cellular Component; MF, Molecular Function; KEGG, Kyoto encyclopedia of genes and genomes pathway.

Enrichment analyses of pleiotropic genes identified by PLACO were conducted using FUMA. The pleiotropic genes showed differential expression in various tissues, including the spleen, lung, small intestine, adipose visceral, and blood (**Figure 4A**). GO enrichment analysis revealed significant enrichment of pleiotropic genes in the type I interferon signaling pathway (*P* = 1.58×10^-12^), T cell activation (*P* = 9.25×10^-11^), and ubiquitin-protein ligase (*P* = 2.25×10^-3^). Additionally, KEGG enrichment analysis demonstrated significant enrichment of genes involved in Th cell differentiation (*P* = 1.50×10^-5^) and the checkpoint pathway in cancer (*P* = 7.10×10^-5^). The top 10 GO and KEGG pathways in **Figure 4B** and **Supplementary Table S14** provided additional evidence that the identified pleiotropic genes were appropriate.

### 3.6 Transcriptome-wide association study

Subsequently, we conducted Transcriptome-wide association studies (TWAS) to explore the relationship between genetically predicted mRNA levels and disease risk, utilizing whole blood collection from the GTEx consortium (29). We identified 621 genes whose expression in blood was associated with hypothyroidism (**Supplementary Table S15**), 139 genes whose expression in blood was associated with RA (discovery) (**Supplementary Table S16**) and 68 genes whose expression in blood was associated with RA (replication) (**Supplementary Table S17**). In total, 37 genes associated with hypothyroidism and RA (discovery) were identified through TWAS (**Figure 3B** and **Supplementary Table S18**). In addition, by using TWAS, 27 genes associated with hypothyroidism and RA (replication) were identified (**Supplementary Table S19**). Among these, 12 genes, including *TYK2* and *IRF5*, were implicated in RA (discovery), RA (replication), and hypothyroidism, according to TWAS.

### 3.7 Summary findings

We performed an overlap analysis of genes in PLACO_FUMA, MTAG, Bayesian colocalization analyses, and TWAS to identify the most representative genes associated with hypothyroidism and RA. This comprehensive approach identified *TYK2*, *IL2RA*, and *IRF5* in hypothyroidism and RA (discovery) utilizing four or three methods. Similarly, an independent replication dataset was analyzed, confirming the discovery dataset results. *TYK2* was consistently identified by all four methods used, while *IL2RA* and *IRF5* were identified in the hypothyroidism and RA (replication) studies using two or three of these methods. These findings suggest that *TYK2*, *IRF5*, and *IL2RA* may be the most representative genes associated with hypothyroidism and RA (**Figures 3A, B**).

## 4 Discussion

This study represents the first genome-wide cross-trait analysis investigating the shared genetic basis that underlies hypothyroidism and RA. Our findings provide novel evidence supporting a genetic interrelation between these two diseases, as evidenced by the following key discoveries: Firstly, we observed an association between hypothyroidism and RA in specific genomic regions. Secondly, our Mendelian randomization analysis revealed a causal effect of hypothyroidism on RA. Finally, we focused on the genetic commonality between hypothyroidism and RA across various tissues and identified potentially functional genes associated with both diseases.

There have been varying results reported in different studies about the relationship between hypothyroidism and RA. For instance, RA is an independent predictor of thyroid dysfunction by Mahagna H et al. (11). Previous studies have indicated that RA coexisting with hypothyroidism may increase disease activity and joint tenderness (33). Three large-scale GWAS summary datasets were chosen to ensure robust heritability interpretation for hypothyroidism and RA. A significant global genetic correlation was found between hypothyroidism and RA in our study. In our exploratory MR analyses, we found evidence of a causal effect of hypothyroidism on RA, aligning with recent study findings (12). The findings of our study suggest that hypothyroid patients can be monitored for RA risk, which will aid in early detection. The association between hypothyroidism and RA may be attributed to the natural course of autoimmune diseases and their propensity to overlap (34). Our enrichment analysis observed a significant enrichment of genes involved in T cell activation, Th cell differentiation, and the interferon signaling pathway. The peripheral Th1/Th2 cell ratio is linked to the severity of Hashimoto’s disease, while the proportion of Th17 cells is associated with the intractability of Graves’ disease (35). Koumine, an alkaloid, demonstrates promising therapeutic effects against RA. Yang J et al. provided detailed elucidation of koumine’s mechanism of action, revealing its ability to effectively restore the balance of Th subsets and cytokine network systems by inhibiting T cell activation. This process leads to the modulation of Th subset polarization and downstream pro/anti-inflammatory cytokine imbalance, which proves beneficial in RA (36). Additionally, in thyroid tissue, the recruitment of T helper 1 (Th1) lymphocytes appears to be associated with heightened production of IFN-γ and tumor necrosis factor‐alpha (TNF‐α) (37). Studies have demonstrated that aberrant expression of IFN-Is and type I IFN-inducible gene signatures in the serum or tissues of patients with autoimmune disorders is closely associated with the pathogenesis, clinical manifestations, and disease activity (38–41). The shared genetic determinants observed in our study reflect common biological pathways that play a crucial role in regulating hypothyroidism and RA.

Among the shared risk genes we identified, Yuan S et al. previously revealed a Mendelian randomized association of *TYK2* loss-of-function variants with hypothyroidism, inflammatory bowel disease, primary biliary cirrhosis, and type 1 diabetes (42). Additionally, in-silico tools were used by Akhtar M et al. to demonstrate the critical role of *TYK2* (rs34536443) in RA pathogenesis (43). The SNP in exon 21 changes proline to alanine at position 1104 of TYK2’s kinase domain (44, 45). TYK2, a non-receptor tyrosine kinase-linked Janus kinase (JAK), belongs to the Janus kinase/signal transduction and transcription factor 4 (JAK-STAT) pathway (46), mediating cytokine signaling (IL-6, IL-10, IL-12, and IL-23 receptors) and regulating group 1 and 2 cytokine pathways (47). Autoimmune diseases are associated with abnormal expression of IFN-I and other cytokines or members of JAK kinase (48, 49). TYK2 plays an important role in many immune processes, including natural killer cell activity, maturation of B and Treg cells, and differentiation of Th1 and Th17 cells. Dysregulated TYK2 expression has been linked to autoimmune diseases (49). Regarding *IL2RA*, according to Knevel et al., altered genetic constitutions at *IL2RA* may lead to a less destructive course of RA (50). In the Chinese Han population, Yang Y et al. found an association between *IL2RA* and a decreased risk of RA (51). Furthermore, *IL2RA* has been identified as a susceptibility gene for autoimmune thyroid disease (52). IL2RA (CD25) is highly expressed in regulatory T cells of CD4CD25 (Tregs) as an important regulator of immune homeostasis and suppressing autoimmune responses (53). In our study, for *TYK2* and *IL2RA*, the most likely risk genes were identified through four methods (PLACO_FUMA, MTAG, Bayesian colocalization analysis, and TWAS) and three methods (PLACO_FUMA, MTAG, and Bayesian colocalization analysis), respectively. This consistency with previous studies adds to the reliability of our integrative analysis. Our study emphasized their significance in hypothyroidism and RA, suggesting their potential role as important drug targets for these diseases. Furthermore, it can be postulated that different SNPs in *TYK2* and *IL2RA* may play different roles in various types of autoimmune diseases.

*IRF5* has been confirmed as a RA disease-associated loci (54, 55). However, its relationship with hypothyroidism remains undefined. IRF5 is a transcription factor involved in inflammation and autoimmune response, mediating the induction of pro-inflammatory cytokines such as IL-6, IL-12, IL-23, and TNF-α (56, 57). As a consequence of IRF5 overexpression, IL6 and IFN are produced at higher levels, playing a crucial role in the pathogenesis of RA (58). Our study identified *IRF5* as a potential risk gene for hypothyroidism and RA, but further validation with larger sample sizes and functional experiments is needed.

There are several limitations to our study, which we acknowledge. Firstly, due to the use of predominantly European ancestry data sources, our findings may not be generalizable to other ethnic groups. Secondly, due to limited data availability, we could not perform phenotype-specific analyses for hypothyroidism’s primary and secondary forms, and did not separate the datasets of people with both diseases for analysis. Third, while we identified genes relevant to hypothyroidism and RA, future longitudinal studies and experimental investigations are necessary to understand the underlying biological mechanisms fully.

## 5 Conclusion

In conclusion, we provide evidence of genetic correlation, identify shared loci, and infer causal relationships between hypothyroidism and RA in our study. In the future, large prospective longitudinal clinical studies should be conducted to determine if hypothyroidism patients with specific genotypes are more likely to develop RA. These findings may have potential implications for future therapeutic strategies and risk prediction in patients with these diseases.

## Data availability statement

The original contributions presented in the study are included in the article/**Supplementary Material**. Further inquiries can be directed to the corresponding author.

## Ethics statement

Ethical approval was not required for the study involving humans in accordance with the local legislation and institutional requirements. Written informed consent to participate in this study was not required from the participants or the participants’ legal guardians/next of kin in accordance with the national legislation and the institutional requirements.

## Author contributions

ZP: Conceptualization, Methodology, Visualization, Writing – original draft, Writing – review & editing. WH: Conceptualization, Writing – review & editing. MT: Data curation, Writing – review & editing. BC: Data curation, Visualization, Writing – review & editing. RY: Formal analysis, Writing – review & editing. QL: Formal analysis, Writing – review & editing. CL: Visualization, Writing – review & editing. PL: Conceptualization, Investigation, Methodology, Visualization, Writing – original draft, Writing – review & editing.
