# Supplementary material for: Investigating the shared genetic architecture between hypothyroidism and rheumatoid arthritis
Source: Front Immunol. 2024 Jan 25;14:1286491. doi: 10.3389/fimmu.2023.1286491 (PMC10850220; doi:10.3389/fimmu.2023.1286491)
Supplement: Supplementary file 1 [file Table_1.docx]

Supplementary Material

Investigating the Shared Genetic Architecture Between Hypothyroidism and Rheumatoid Arthritis

Zhifang Peng^1^^†^, Weiping Huang^2†^, Mengjun Tang^3^, Binbin Chen^1^, Renqi Yang^1^, Qing Liu^1^, Chaoshui Liu^4^, and Panpan Long^1*^

# * Correspondence: Panpan Long

# Phone: +86-13786675505, E-mail: [286397715@qq.com](mailto:286397715@qq.com)

**Supplemental Table S1. Details of GWAS Summary Data Sources.**

| **Traits** | **Sample size** | **Repository** | **Comment** |
| --- | --- | --- | --- |
| Hypothyroidism | 462,933 (22,687 cases and 440,246 conrols) | <https://gwas.mrcieu.ac.uk/datasets/ukb-b-19732/> | ukb-b-19732 |
| Rheumatoid arthritis  (Discovery) | 253,417 (12,555 cases and 240,862 conrols) | <https://r9.finngen.fi/pheno/M13_RHEUMA> | finngen_R9_M13_RHEUMA |
| Rheumatoid arthritis  (Replication) | 58,284 (14,361 cases and 43,923 conrols) | <http://plaza.umin.ac.jp/~yokada/datasource/software.htm> | Eurpean RA GWAS meta-analysis |

| **LDSC test without constrained intercept** | | |
| --- | --- | --- |
| Trait 1-Trait 2 | hypothyroidism-RA (discovery) | hypothyroidism-RA (replication) |
| r_g_ | 0.3109 | 0.3523 |
| standard error(r_g_) | 0.0626 | 0.0546 |
| *P* value | 6.70E-07 | 1.13E-10 |
| Intercept (genetic correlation) | 0.0125 | 0.0158 |
| Lambda GC (Trait 1) | 1.2564 | 1.2597 |
| Intercept (Trait 1) | 1.064 | 1.064 |
| Lambda GC (Trait 2) | 1.1651 | 1.0466 |
| Intercept (Trait 2) | 1.085 | 0.9548 |
| **LDSC test with constrained intercept** | | |
| r_g_ | 0.257 | 0.4839 |
| standard error(r_g_) | 0.0266 | 0.047 |
| *P* value | 4.20E-22 | 7.22E-55 |
| Lambda GC (Trait 1) | 1.2564 | 1.2597 |
| Intercept (Trait 1) | 1 | 1 |
| Lambda GC (Trait 2) | 1.1651 | 1.0466 |
| Intercept (Trait 2) | 1 | 1 |

**Supplemental Table S2. Genetic correlations estimated using linkage disequilibrium score.**

**Supplemental Table S3. Local genetic correlation between hypothyroidism and RA (discovery).**

| **Chr** | **Start** | **End** | **Number of SNPs** | **local_rhog** | **SE** | ***P*** |
| --- | --- | --- | --- | --- | --- | --- |
| 6 | 31571218 | 32682664 | 3762 | 3.21E-03 | 2.11E-04 | 3.63E-52 |
| 1 | 113273306 | 114873845 | 2191 | 1.28E-03 | 1.09E-04 | 4.29E-32 |
| 6 | 32682664 | 33236497 | 2876 | 1.55E-03 | 1.80E-04 | 7.36E-18 |
| 12 | 110336719 | 113263518 | 2009 | 5.04E-04 | 8.59E-05 | 4.58E-09 |
| 2 | 189882065 | 191973357 | 2525 | 3.60E-04 | 6.88E-05 | 1.63E-07 |
| 2 | 202818637 | 205799241 | 3362 | 4.29E-04 | 8.41E-05 | 3.30E-07 |

RA: rheumatoid arthritis; SE: standard error; SNP: single nucleotide polymorphism. The start and end described the base pair.

**Supplemental Table S4. Local genetic correlation between hypothyroidism and RA (replication).**

| **Chr** | **Start** | **End** | **Number of SNPs** | **local_rhog** | **SE** | ***P*** |
| --- | --- | --- | --- | --- | --- | --- |
| 6 | 32682664 | 33236497 | 2809 | 7.70E-03 | 3.06E-04 | 1.58E-139 |
| 1 | 113273306 | 114873845 | 2058 | 3.37E-03 | 1.61E-04 | 2.47E-97 |
| 6 | 31571218 | 32682664 | 3425 | 8.67E-03 | 4.28E-04 | 2.05E-91 |
| 6 | 33236497 | 35455756 | 3228 | 1.51E-03 | 1.39E-04 | 2.39E-27 |
| 2 | 202818637 | 205799241 | 3000 | 1.20E-03 | 1.16E-04 | 6.02E-25 |
| 2 | 189882065 | 191973357 | 2271 | 8.30E-04 | 8.35E-05 | 2.80E-23 |
| 10 | 5983762 | 7171484 | 2452 | 6.96E-04 | 8.26E-05 | 3.68E-17 |
| 10 | 63341695 | 65794114 | 3640 | 6.31E-04 | 8.06E-05 | 5.05E-15 |
| 6 | 29737971 | 30798168 | 4140 | 1.10E-03 | 1.64E-04 | 2.20E-11 |
| 12 | 110336719 | 113263518 | 1713 | 6.83E-04 | 1.11E-04 | 6.95E-10 |
| 6 | 28917608 | 29737971 | 1833 | 8.40E-04 | 1.37E-04 | 9.10E-10 |
| 6 | 137614218 | 138822629 | 1967 | 4.20E-04 | 7.00E-05 | 1.98E-09 |
| 18 | 11905440 | 14440799 | 3525 | 4.14E-04 | 7.10E-05 | 5.65E-09 |
| 7 | 126869221 | 128778386 | 2618 | 3.98E-04 | 7.27E-05 | 4.38E-08 |
| 4 | 25609322 | 27343722 | 2095 | 4.02E-04 | 7.70E-05 | 1.83E-07 |
| 1 | 37549183 | 38731847 | 1664 | 3.58E-04 | 7.50E-05 | 1.79E-06 |
| 1 | 156336133 | 158027412 | 2282 | 3.20E-04 | 6.78E-05 | 2.39E-06 |
| 21 | 43321528 | 44506268 | 2692 | 3.06E-04 | 6.55E-05 | 2.99E-06 |
| 3 | 104581613 | 106982811 | 3549 | 3.26E-04 | 7.33E-05 | 8.65E-06 |
| 6 | 167024733 | 168042835 | 1669 | 3.65E-04 | 8.27E-05 | 1.01E-05 |
| 1 | 66939404 | 68477895 | 2570 | 2.87E-04 | 6.72E-05 | 1.90E-05 |
| 15 | 38530777 | 40384132 | 2807 | 3.15E-04 | 7.45E-05 | 2.32E-05 |

RA:rheumatoid arthritis; SE: standard error; SNP: single nucleotide polymorphism. The start and end described the base pair.

**Supplemental Table S5. 805 single nucleotide variants identified by PLACO in hypothyroidism and RA (discovery).**

| **SNP** | ***P*.hypothyroidism** | **Z.hypothyroidism** | ***P*.RA (discovery)** | **Z.RA (discovery)** | **T.placo** | ***P*.placo** |
| --- | --- | --- | --- | --- | --- | --- |
| rs10002 | 8.60E-04 | 3.332795085 | 2.72E-07 | 5.141791045 | 17.08756353 | 2.39E-08 |
| rs10012589 | 1.93E-04 | -3.728515338 | 8.87E-14 | 7.456692913 | -27.89345834 | 7.48E-13 |
| rs10012701 | 1.94E-04 | -3.727164132 | 9.42E-14 | 7.448818898 | -27.85386817 | 7.77E-13 |
| rs10013070 | 1.51E-04 | -3.788978184 | 1.14E-12 | 7.112781955 | -27.03527735 | 1.70E-12 |
| rs10013228 | 9.55E-05 | -3.901656305 | 1.85E-13 | 7.359375 | -28.80466464 | 3.13E-13 |
| rs10020668 | 9.62E-05 | -3.900069281 | 2.20E-13 | -7.3359375 | 28.52061021 | 4.11E-13 |
| rs10026340 | 1.51E-04 | -3.789317479 | 8.58E-13 | -7.151515152 | 27.01390351 | 1.73E-12 |
| rs10080229 | 8.37E-04 | 3.340306559 | 2.72E-07 | -5.141791045 | -17.2244309 | 2.09E-08 |
| rs10214450 | 3.58E-04 | 3.569140372 | 1.79E-06 | 4.776173285 | 17.00046822 | 2.60E-08 |
| rs10223515 | 1.60E-03 | 3.155447592 | 2.93E-11 | 6.650306748 | 20.91399484 | 6.01E-10 |
| rs1023691 | 9.82E-05 | -3.894921666 | 2.30E-06 | -4.725352113 | 18.35597298 | 7.03E-09 |
| rs1023692 | 5.18E-05 | -4.047333676 | 7.42E-06 | 4.481203008 | -18.18472085 | 8.29E-09 |
| rs1023693 | 9.19E-05 | -3.910938008 | 2.14E-06 | 4.73943662 | -18.58513651 | 5.64E-09 |
| rs10501121 | 1.58E-06 | -4.800401333 | 1.14E-07 | 5.302521008 | -25.52129376 | 7.24E-12 |
| rs10501122 | 1.54E-06 | -4.805631152 | 9.95E-08 | 5.327731092 | -25.67059244 | 6.28E-12 |
| rs10501124 | 4.83E-05 | -4.063869805 | 7.39E-08 | -5.381355932 | 21.80982361 | 2.54E-10 |
| rs10501125 | 4.80E-05 | -4.065265256 | 7.39E-08 | 5.381355932 | -21.93625953 | 2.25E-10 |
| rs10517343 | 1.95E-04 | -3.725990913 | 9.42E-14 | -7.448818898 | 27.66372503 | 9.32E-13 |
| rs10737462 | 8.12E-05 | 3.940880046 | 3.94E-14 | 7.562962963 | 29.70984291 | 1.32E-13 |
| rs10742238 | 5.85E-06 | -4.531590115 | 7.90E-05 | 3.947368421 | -17.93519986 | 1.05E-08 |
| rs10767832 | 1.43E-06 | -4.821242354 | 1.72E-07 | -5.226890756 | 25.13416763 | 1.05E-11 |
| rs10767833 | 5.86E-06 | -4.5315558 | 7.90E-05 | 3.947368421 | -17.935064 | 1.05E-08 |
| rs10767835 | 5.44E-06 | -4.546883347 | 7.90E-05 | -3.947368421 | 17.9009431 | 1.09E-08 |
| rs10767836 | 5.45E-06 | -4.546548608 | 8.82E-05 | 3.921052632 | -17.87450693 | 1.12E-08 |
| rs10767837 | 5.48E-06 | -4.545590782 | 8.20E-05 | 3.938596491 | -17.95066788 | 1.04E-08 |
| rs10767839 | 6.31E-06 | -4.515736435 | 1.06E-04 | -3.877192982 | 17.46218496 | 1.66E-08 |
| rs10777679 | 8.93E-04 | 3.322133188 | 5.52E-08 | -5.433628319 | -18.10439243 | 8.96E-09 |
| rs10799737 | 4.24E-04 | -3.524395354 | 3.40E-07 | 5.1 | -18.02478696 | 9.67E-09 |
| rs10835617 | 4.73E-05 | -4.068371474 | 7.84E-06 | 4.469565217 | -18.231736 | 7.92E-09 |
| rs10835618 | 4.73E-05 | -4.068360085 | 8.16E-06 | -4.460869565 | 18.1008894 | 8.99E-09 |
| rs10835620 | 4.83E-05 | -4.063609016 | 8.12E-08 | -5.36440678 | 21.73978591 | 2.72E-10 |
| rs10835621 | 4.77E-05 | -4.066375414 | 8.51E-08 | 5.355932203 | -21.83850548 | 2.47E-10 |
| rs10835622 | 4.83E-05 | -4.063824755 | 5.57E-08 | -5.43220339 | 22.01549933 | 2.09E-10 |
| rs10835624 | 4.96E-05 | -4.057566062 | 8.92E-08 | 5.347457627 | -21.75672431 | 2.67E-10 |
| rs10835626 | 5.05E-05 | -4.053274595 | 7.39E-08 | 5.381355932 | -21.8716058 | 2.39E-10 |
| rs10835627 | 4.83E-05 | -4.063869805 | 7.39E-08 | -5.381355932 | 21.80982361 | 2.54E-10 |
| rs10835628 | 4.83E-05 | -4.063869805 | 7.39E-08 | -5.381355932 | 21.80982361 | 2.54E-10 |
| rs10835629 | 4.83E-05 | -4.063869805 | 7.39E-08 | -5.381355932 | 21.80982361 | 2.54E-10 |
| rs10835630 | 4.83E-05 | -4.063869805 | 6.73E-08 | 5.398305085 | -21.99785373 | 2.12E-10 |
| rs10835631 | 2.92E-05 | -4.179531232 | 5.06E-08 | 5.449152542 | -22.83672141 | 9.48E-11 |
| rs10835634 | 9.74E-07 | -4.896741589 | 1.06E-07 | 5.316666667 | -26.10282946 | 4.15E-12 |
| rs10835638 | 7.33E-09 | -5.783078407 | 1.60E-18 | -8.782608696 | 50.64628461 | 2.93E-22 |
| rs10835643 | 7.68E-06 | 4.473977145 | 2.43E-04 | -3.669491525 | -16.46110963 | 4.37E-08 |
| rs10835644 | 4.99E-09 | -5.847476886 | 2.83E-19 | -8.975308642 | 52.33323793 | 5.91E-23 |
| rs10835645 | 4.99E-09 | -5.847476886 | 2.99E-19 | -8.969135802 | 52.29728697 | 6.11E-23 |
| rs10835646 | 4.98E-09 | -5.847772624 | 2.99E-19 | -8.969135802 | 52.29993498 | 6.09E-23 |
| rs10835649 | 3.69E-13 | -7.266428322 | 2.10E-21 | 9.5 | -69.21857557 | 6.72E-30 |
| rs10835655 | 6.09E-09 | -5.814141367 | 6.95E-19 | -8.875776398 | 51.45817214 | 1.35E-22 |
| rs10835658 | 6.92E-09 | -5.792860147 | 6.21E-19 | -8.888198758 | 51.34127942 | 1.51E-22 |
| rs10835660 | 2.50E-07 | -5.157784436 | 7.28E-18 | 8.61038961 | -44.54255348 | 9.62E-20 |
| rs10835661 | 4.97E-06 | -4.566242894 | 1.74E-17 | 8.509803922 | -38.98004138 | 1.91E-17 |
| rs10859856 | 9.21E-04 | 3.313757808 | 8.19E-08 | -5.362831858 | -17.82320746 | 1.17E-08 |
| rs10917119 | 1.61E-03 | 3.154918293 | 4.05E-19 | 8.935483871 | 28.07350844 | 6.30E-13 |
| rs10917123 | 2.90E-03 | 2.978015323 | 1.48E-11 | 6.75 | 20.03057403 | 1.40E-09 |
| rs10917128 | 1.20E-03 | 3.239719771 | 2.32E-20 | -9.246666667 | -30.08232611 | 9.23E-14 |
| rs10917130 | 9.57E-04 | 3.302735934 | 4.01E-20 | -9.187919463 | -30.47011481 | 6.38E-14 |
| rs10917151 | 3.08E-05 | 4.167702132 | 8.09E-23 | 9.833333333 | 40.83354933 | 3.27E-18 |
| rs11030946 | 2.73E-04 | -3.640002237 | 2.37E-08 | -5.582352941 | 20.26184853 | 1.12E-09 |
| rs11030958 | 4.57E-05 | -4.076771797 | 6.13E-06 | 4.52173913 | -18.48268745 | 6.22E-09 |
| rs11030960 | 4.73E-05 | -4.068382863 | 7.53E-06 | 4.47826087 | -18.26726624 | 7.66E-09 |
| rs11030961 | 4.73E-05 | -4.068371474 | 7.84E-06 | -4.469565217 | 18.13621594 | 8.69E-09 |
| rs11030962 | 4.73E-05 | -4.068371474 | 7.84E-06 | 4.469565217 | -18.231736 | 7.92E-09 |
| rs11030966 | 4.83E-05 | -4.06384728 | 8.51E-08 | 5.355932203 | -21.824938 | 2.50E-10 |
| rs11030968 | 4.83E-05 | -4.063824755 | 8.51E-08 | 5.355932203 | -21.82481712 | 2.50E-10 |
| rs11030969 | 4.83E-05 | -4.063824755 | 8.51E-08 | -5.355932203 | 21.70662029 | 2.81E-10 |
| rs11030970 | 4.73E-05 | -4.068734606 | 8.51E-08 | -5.355932203 | 21.73286509 | 2.74E-10 |
| rs11030971 | 4.83E-05 | -4.063824755 | 8.92E-08 | 5.347457627 | -21.79025909 | 2.59E-10 |
| rs11030972 | 4.83E-05 | -4.063824755 | 8.12E-08 | 5.36440678 | -21.85937533 | 2.42E-10 |
| rs11030974 | 4.83E-05 | -4.063824755 | 8.92E-08 | 5.347457627 | -21.79025909 | 2.59E-10 |
| rs11030977 | 4.83E-05 | -4.063869805 | 6.12E-08 | -5.415254237 | 21.94710434 | 2.23E-10 |
| rs11030982 | 2.94E-05 | -4.177757896 | 5.31E-08 | 5.440677966 | -22.79151316 | 9.91E-11 |
| rs11030983 | 2.91E-05 | -4.180753662 | 5.06E-08 | -5.449152542 | 22.72004442 | 1.06E-10 |
| rs11030985 | 2.98E-05 | -4.175032361 | 3.80E-08 | -5.5 | 22.90049378 | 8.92E-11 |
| rs11030986 | 2.99E-05 | -4.173910791 | 3.99E-08 | 5.491525424 | -22.98350114 | 8.24E-11 |
| rs11030987 | 2.92E-05 | -4.179676631 | 5.06E-08 | 5.449152542 | -22.83751531 | 9.48E-11 |
| rs11030988 | 2.98E-05 | -4.175054892 | 3.99E-08 | -5.491525424 | 22.86535719 | 9.23E-11 |
| rs11030989 | 2.99E-05 | -4.173963984 | 2.29E-08 | 5.588235294 | -23.38886109 | 5.59E-11 |
| rs11030991 | 1.40E-06 | -4.824303252 | 1.14E-07 | -5.302521008 | 25.51395269 | 7.29E-12 |
| rs11030992 | 1.47E-06 | -4.815770982 | 1.31E-07 | -5.277310924 | 25.34775929 | 8.55E-12 |
| rs11030993 | 1.62E-06 | -4.795794136 | 1.31E-07 | 5.277310924 | -25.3755541 | 8.32E-12 |
| rs11030997 | 1.34E-06 | -4.834051305 | 1.52E-07 | 5.25 | -25.44553292 | 7.78E-12 |
| rs11030998 | 9.92E-07 | -4.89325416 | 1.27E-07 | -5.283333333 | 25.78506754 | 5.62E-12 |
| rs11031002 | 1.27E-07 | -5.282629025 | 5.07E-16 | -8.10982659 | 42.71902067 | 5.45E-19 |
| rs11031005 | 4.76E-12 | -6.912472482 | 7.48E-19 | 8.86746988 | -61.46184322 | 1.03E-26 |
| rs11031006 | 5.29E-12 | -6.897616678 | 8.34E-19 | -8.855421687 | 60.91698684 | 1.73E-26 |
| rs11031010 | 5.75E-09 | -5.823868364 | 3.88E-20 | -9.191358025 | 53.37482307 | 2.20E-23 |
| rs11031014 | 7.48E-06 | 4.479437858 | 2.43E-04 | -3.669491525 | -16.48121173 | 4.28E-08 |
| rs11031022 | 6.83E-06 | 4.499049591 | 2.35E-04 | 3.677966102 | 16.50331332 | 4.19E-08 |
| rs11031025 | 7.68E-06 | 4.47395553 | 2.35E-04 | 3.677966102 | 16.41131199 | 4.58E-08 |
| rs11031032 | 7.66E-06 | 4.474406932 | 2.06E-04 | 3.711864407 | 16.56431528 | 3.95E-08 |
| rs11031033 | 5.01E-09 | -5.84693147 | 3.54E-19 | -8.950617284 | 52.18456 | 6.80E-23 |
| rs11031034 | 4.91E-09 | -5.850128777 | 2.99E-19 | -8.969135802 | 52.32103174 | 5.97E-23 |
| rs11031035 | 5.00E-09 | -5.847344582 | 2.67E-19 | 8.981481481 | -52.66834986 | 4.30E-23 |
| rs11031038 | 6.50E-06 | -4.509375136 | 7.90E-05 | 3.947368421 | -17.84724596 | 1.15E-08 |
| rs11031039 | 5.01E-09 | -5.846815366 | 2.02E-19 | -9.01242236 | 52.54343634 | 4.84E-23 |
| rs11031040 | 4.18E-13 | -7.249448739 | 1.75E-21 | 9.518987342 | -69.19506703 | 6.87E-30 |
| rs11031047 | 3.69E-13 | -7.266428322 | 8.61E-22 | -9.592356688 | 69.51330897 | 5.09E-30 |
| rs11031052 | 6.10E-09 | -5.814016718 | 8.21E-19 | -8.857142857 | 51.34916324 | 1.50E-22 |
| rs11031056 | 6.37E-09 | -5.806771527 | 1.50E-17 | -8.526946108 | 49.37521608 | 9.77E-22 |
| rs11031058 | 2.50E-07 | -5.157411982 | 7.71E-18 | -8.603896104 | 44.24257517 | 1.28E-19 |
| rs11107959 | 8.87E-04 | 3.324083307 | 5.25E-08 | -5.442477876 | -18.14454839 | 8.62E-09 |
| rs11126134 | 3.53E-05 | -4.136205097 | 2.77E-06 | 4.68707483 | -19.43792709 | 2.48E-09 |
| rs11126138 | 2.90E-05 | -4.181082161 | 2.79E-06 | -4.685714286 | 19.53992731 | 2.25E-09 |
| rs11126143 | 2.43E-04 | -3.669096692 | 2.82E-06 | -4.683333333 | 17.13744138 | 2.27E-08 |
| rs111270458 | 2.30E-06 | 4.72526741 | 1.90E-05 | 4.276923077 | 20.15663381 | 1.24E-09 |
| rs111407432 | 1.38E-05 | -4.347143638 | 2.01E-13 | -7.34841629 | 31.84952927 | 1.71E-14 |
| rs111550108 | 8.21E-06 | -4.459579031 | 6.74E-08 | -5.398089172 | 24.00926841 | 3.08E-11 |
| rs111610638 | 2.84E-05 | -4.185630862 | 8.98E-08 | -5.346153846 | 22.31690416 | 1.56E-10 |
| rs111633949 | 8.43E-06 | -4.4540121 | 4.27E-08 | 5.479233227 | -24.46992857 | 1.98E-11 |
| rs111992780 | 1.27E-03 | 3.223609364 | 4.05E-20 | 9.186666667 | 29.49049693 | 1.63E-13 |
| rs112578997 | 8.50E-06 | -4.452170882 | 4.08E-08 | -5.487261146 | 24.36510707 | 2.19E-11 |
| rs113766362 | 7.36E-06 | -4.483027142 | 1.89E-08 | -5.622222222 | 25.13714206 | 1.05E-11 |
| rs114078082 | 2.56E-04 | 3.656303394 | 5.81E-06 | 4.533112583 | 16.53020202 | 4.08E-08 |
| rs114088263 | 3.71E-04 | 3.560076891 | 2.11E-06 | -4.742857143 | -16.93103368 | 2.77E-08 |
| rs114101204 | 9.76E-09 | -5.734872053 | 2.54E-04 | -3.658482143 | 20.9205697 | 5.97E-10 |
| rs114137400 | 1.12E-06 | 4.868506618 | 1.37E-06 | 4.828793774 | 23.4476986 | 5.28E-11 |
| rs114183675 | 4.30E-04 | 3.520923999 | 1.38E-06 | 4.828571429 | 16.95445699 | 2.71E-08 |
| rs114344942 | 3.29E-04 | 3.591071653 | 2.72E-06 | 4.690647482 | 16.79893843 | 3.15E-08 |
| rs114472600 | 4.33E-04 | 3.519178207 | 1.30E-06 | -4.839285714 | -17.07723692 | 2.41E-08 |
| rs114476612 | 2.74E-04 | 3.638400586 | 3.08E-06 | 4.665467626 | 16.92918891 | 2.78E-08 |
| rs11493214 | 1.30E-06 | -4.839274855 | 1.31E-07 | -5.277310924 | 25.47150068 | 7.59E-12 |
| rs114964168 | 9.87E-09 | -5.732983584 | 2.96E-04 | -3.618834081 | 20.68676304 | 7.47E-10 |
| rs115166491 | 3.74E-04 | 3.557983533 | 1.90E-06 | 4.764285714 | 16.90513703 | 2.85E-08 |
| rs115429675 | 1.92E-06 | 4.761727741 | 9.56E-06 | 4.426966292 | 21.02488298 | 5.40E-10 |
| rs115450053 | 3.64E-04 | 3.56486876 | 4.75E-06 | -4.575539568 | -16.35529784 | 4.84E-08 |
| rs115780893 | 3.73E-04 | 3.558500942 | 1.96E-06 | -4.757142857 | -16.97455798 | 2.66E-08 |
| rs115805051 | 1.29E-07 | 5.280144712 | 1.45E-05 | -4.33557047 | -22.95362481 | 8.48E-11 |
| rs116141873 | 3.36E-04 | 3.585735912 | 4.59E-06 | 4.582733813 | 16.3883161 | 4.68E-08 |
| rs116158112 | 3.15E-04 | 3.602945986 | 4.67E-06 | 4.579136691 | 16.45410683 | 4.40E-08 |
| rs116344531 | 3.77E-04 | 3.55545049 | 2.21E-06 | -4.732851986 | -16.87335109 | 2.93E-08 |
| rs116526250 | 3.62E-04 | 3.566284779 | 1.80E-06 | 4.774193548 | 16.97982033 | 2.65E-08 |
| rs116621273 | 2.08E-07 | 5.192262731 | 1.55E-05 | -4.322147651 | -22.50155315 | 1.31E-10 |
| rs116638091 | 3.34E-04 | 3.587202629 | 4.54E-06 | 4.584837545 | 16.40254531 | 4.62E-08 |
| rs116836493 | 3.54E-04 | 3.572159048 | 2.32E-06 | -4.723021583 | -16.91734893 | 2.81E-08 |
| rs117027891 | 9.27E-07 | 4.906508527 | 2.05E-06 | 4.748062016 | 23.23561465 | 6.47E-11 |
| rs11751190 | 7.55E-04 | 3.368922745 | 9.84E-19 | 8.836879433 | 29.6540277 | 1.39E-13 |
| rs11755383 | 1.75E-02 | 2.376391454 | 4.52E-13 | -7.238993711 | -17.27869925 | 1.98E-08 |
| rs11756362 | 1.75E-02 | 2.375455686 | 4.32E-13 | -7.245283019 | -17.28697855 | 1.97E-08 |
| rs118069541 | 1.39E-07 | 5.266026936 | 1.62E-05 | 4.312080537 | 22.647119 | 1.14E-10 |
| rs11900280 | 1.10E-04 | -3.866888309 | 3.28E-06 | 4.65248227 | -18.03860355 | 9.54E-09 |
| rs12027753 | 1.59E-03 | 3.157105524 | 3.85E-20 | 9.19205298 | 28.8969748 | 2.87E-13 |
| rs12037376 | 1.13E-05 | 4.39065384 | 6.88E-24 | 10.07843137 | 44.09319513 | 1.47E-19 |
| rs12038474 | 5.75E-04 | 3.443095063 | 3.21E-21 | 9.455782313 | 32.42497709 | 9.86E-15 |
| rs12042083 | 2.42E-03 | 3.032554581 | 2.81E-15 | 7.899280576 | 23.86155514 | 3.55E-11 |
| rs12055461 | 5.28E-03 | 2.789611138 | 7.39E-10 | -6.157575758 | -17.23710758 | 2.07E-08 |
| rs12093861 | 6.17E-03 | 2.738587901 | 2.58E-09 | -5.956521739 | -16.36876488 | 4.77E-08 |
| rs1209731 | 9.85E-13 | 7.132589208 | 2.78E-04 | -3.635036496 | -26.01119145 | 4.53E-12 |
| rs12111427 | 7.14E-04 | 3.384060638 | 9.84E-19 | 8.836879433 | 29.78766669 | 1.22E-13 |
| rs12173791 | 2.12E-08 | 5.601546256 | 1.60E-14 | 7.67961165 | 42.89985945 | 4.59E-19 |
| rs1222203 | 6.77E-06 | -4.500637126 | 2.77E-04 | 3.63559322 | -16.40636292 | 4.60E-08 |
| rs1222204 | 6.77E-06 | -4.500637126 | 2.87E-04 | 3.627118644 | -16.36814119 | 4.78E-08 |
| rs1222220 | 6.78E-06 | -4.500633929 | 2.68E-04 | -3.644067797 | 16.35688147 | 4.83E-08 |
| rs12223987 | 4.72E-06 | -4.576996438 | 3.05E-18 | -8.709677419 | 39.73786262 | 9.28E-18 |
| rs12270725 | 4.73E-05 | -4.068360085 | 7.84E-06 | 4.469565217 | -18.23168497 | 7.92E-09 |
| rs12271187 | 4.31E-13 | -7.245509295 | 1.75E-21 | -9.518987342 | 68.78327315 | 1.01E-29 |
| rs12271300 | 4.61E-13 | -7.236441439 | 1.65E-21 | -9.525316456 | 68.74277042 | 1.05E-29 |
| rs12272116 | 4.83E-05 | -4.06384728 | 8.51E-08 | 5.355932203 | -21.824938 | 2.50E-10 |
| rs12272134 | 6.09E-09 | -5.814125786 | 7.76E-19 | 8.863354037 | -51.67991872 | 1.10E-22 |
| rs12278112 | 6.10E-09 | -5.814055671 | 8.21E-19 | 8.857142857 | -51.6430399 | 1.14E-22 |
| rs12278547 | 6.10E-09 | -5.814079043 | 7.34E-19 | 8.869565217 | -51.71576025 | 1.06E-22 |
| rs12278989 | 4.31E-13 | -7.245509295 | 1.65E-21 | 9.525316456 | -69.20350816 | 6.82E-30 |
| rs12281078 | 4.99E-09 | -5.847476886 | 1.80E-19 | 9.02484472 | -52.92412833 | 3.37E-23 |
| rs12282076 | 2.65E-05 | -4.202053472 | 3.99E-08 | -5.491525424 | 23.01332588 | 8.01E-11 |
| rs12282113 | 3.00E-05 | -4.173371697 | 3.99E-08 | -5.491525424 | 22.85613219 | 9.31E-11 |
| rs12282163 | 2.96E-05 | -4.176616705 | 3.99E-08 | -5.491525424 | 22.87391693 | 9.15E-11 |
| rs12282309 | 4.83E-05 | -4.063869805 | 7.39E-08 | 5.381355932 | -21.92873523 | 2.27E-10 |
| rs12284112 | 4.73E-05 | -4.068360085 | 6.94E-06 | -4.495652174 | 18.24199123 | 7.84E-09 |
| rs12285277 | 6.30E-09 | -5.808619373 | 6.57E-19 | 8.881987578 | -51.73969763 | 1.04E-22 |
| rs12287556 | 2.94E-05 | -4.17800764 | 5.31E-08 | 5.440677966 | -22.79287467 | 9.89E-11 |
| rs12290734 | 4.73E-05 | -4.068360085 | 7.84E-06 | 4.469565217 | -18.23168497 | 7.92E-09 |
| rs12290971 | 4.73E-05 | -4.068360085 | 6.66E-06 | 4.504347826 | -18.37360222 | 6.91E-09 |
| rs12291589 | 4.83E-05 | -4.06384728 | 8.51E-08 | -5.355932203 | 21.70674069 | 2.80E-10 |
| rs12293737 | 2.95E-05 | -4.17765278 | 5.31E-08 | 5.440677966 | -22.7909401 | 9.91E-11 |
| rs12294104 | 4.86E-06 | -4.57066748 | 1.65E-17 | -8.516339869 | 38.80348151 | 2.26E-17 |
| rs12294258 | 4.99E-09 | -5.847476886 | 2.02E-19 | -9.01242236 | 52.54938817 | 4.81E-23 |
| rs12320196 | 8.98E-04 | 3.320652485 | 5.25E-08 | -5.442477876 | -18.12584627 | 8.77E-09 |
| rs12331471 | 1.81E-04 | -3.744514045 | 1.06E-13 | 7.433070866 | -27.92399936 | 7.27E-13 |
| rs12331507 | 9.35E-04 | -3.309380809 | 3.86E-12 | -6.94214876 | 22.89704025 | 8.95E-11 |
| rs12331538 | 9.72E-05 | -3.897551741 | 2.08E-13 | 7.34375 | -28.71321552 | 3.42E-13 |
| rs12331597 | 5.25E-04 | -3.467462303 | 1.72E-13 | 7.368852459 | -25.63810836 | 6.47E-12 |
| rs1235089 | 2.02E-07 | -5.197796886 | 1.24E-03 | 3.229508197 | -16.83540292 | 3.04E-08 |
| rs12361594 | 4.84E-09 | -5.852508822 | 2.83E-19 | -8.97515528 | 52.37743048 | 5.66E-23 |
| rs12363432 | 6.91E-09 | -5.792980188 | 6.57E-19 | -8.881987578 | 51.30650739 | 1.56E-22 |
| rs12363824 | 3.69E-13 | -7.266428322 | 9.74E-22 | -9.579617834 | 69.4210617 | 5.55E-30 |
| rs12364889 | 4.94E-09 | -5.849095366 | 1.80E-19 | 9.024691358 | -52.93785906 | 3.33E-23 |
| rs12365913 | 6.35E-09 | -5.807338521 | 6.21E-19 | 8.888198758 | -51.76451657 | 1.01E-22 |
| rs12404660 | 2.99E-05 | 4.17443285 | 6.62E-19 | -8.881118881 | -37.19979722 | 1.04E-16 |
| rs12405695 | 1.24E-03 | 3.230321087 | 3.81E-20 | 9.193333333 | 29.5734745 | 1.50E-13 |
| rs12410251 | 2.12E-03 | 3.072585649 | 2.27E-16 | 8.207142857 | 25.11691095 | 1.07E-11 |
| rs12498317 | 1.23E-03 | -3.231728028 | 1.09E-12 | -7.119047619 | 22.92706422 | 8.70E-11 |
| rs12505096 | 1.47E-03 | -3.180258696 | 2.93E-13 | -7.297709924 | 23.12590913 | 7.19E-11 |
| rs12525200 | 9.57E-04 | 3.302880908 | 2.53E-07 | 5.155555556 | 16.9792871 | 2.65E-08 |
| rs12561946 | 1.23E-04 | 3.840923417 | 1.57E-05 | -4.318965517 | -16.63260668 | 3.70E-08 |
| rs12786317 | 4.83E-05 | -4.063869805 | 6.73E-08 | -5.398305085 | 21.87846435 | 2.38E-10 |
| rs12790814 | 4.74E-05 | -4.067932703 | 7.84E-06 | -4.469565217 | 18.13425948 | 8.70E-09 |
| rs12792185 | 4.75E-05 | -4.06781881 | 7.84E-06 | -4.469565217 | 18.13375163 | 8.71E-09 |
| rs12793581 | 1.55E-06 | -4.804864757 | 1.04E-07 | 5.319327731 | -25.62600536 | 6.55E-12 |
| rs12794770 | 4.70E-05 | -4.069903554 | 7.84E-06 | -4.469565217 | 18.14304742 | 8.63E-09 |
| rs12796113 | 4.70E-05 | -4.069889663 | 7.84E-06 | 4.469565217 | -18.23853784 | 7.87E-09 |
| rs12796334 | 4.70E-05 | -4.069903554 | 7.53E-06 | 4.47826087 | -18.27409252 | 7.61E-09 |
| rs12800683 | 4.43E-05 | -4.083641817 | 9.22E-06 | -4.434782609 | 18.0626697 | 9.32E-09 |
| rs12801198 | 4.73E-05 | -4.068360085 | 7.23E-06 | 4.486956522 | -18.3026432 | 7.40E-09 |
| rs12802602 | 4.68E-05 | -4.071056983 | 7.53E-06 | 4.47826087 | -18.27927019 | 7.57E-09 |
| rs13192494 | 4.57E-04 | 3.505045426 | 7.16E-19 | 8.872340426 | 30.97918441 | 3.92E-14 |
| rs13201167 | 2.68E-05 | 4.199253278 | 1.09E-09 | -6.095652174 | -25.66899946 | 6.28E-12 |
| rs13206045 | 9.08E-03 | 2.609220423 | 8.42E-14 | 7.463576159 | 19.3925148 | 2.59E-09 |
| rs13211170 | 3.73E-04 | 3.558179486 | 5.24E-19 | -8.907142857 | -31.81371387 | 1.77E-14 |
| rs13212683 | 2.46E-05 | 4.218674851 | 3.42E-10 | -6.27826087 | -26.56092749 | 2.68E-12 |
| rs13218956 | 8.74E-03 | 2.622212808 | 4.83E-14 | 7.536423841 | 19.67899099 | 1.97E-09 |
| rs1340030 | 2.91E-05 | -4.180097457 | 5.31E-08 | 5.440677966 | -22.80426761 | 9.78E-11 |
| rs1340032 | 4.95E-05 | -4.058147458 | 6.12E-08 | 5.415254237 | -22.03592404 | 2.05E-10 |
| rs1340033 | 4.83E-05 | -4.063869805 | 7.05E-08 | 5.389830508 | -21.96329439 | 2.19E-10 |
| rs1350545 | 2.96E-04 | -3.618470672 | 8.12E-13 | -7.159090909 | 25.82100745 | 5.43E-12 |
| rs1361421 | 3.03E-05 | -4.171226434 | 4.18E-08 | -5.483050847 | 22.80914673 | 9.74E-11 |
| rs1361422 | 2.95E-05 | -4.177589881 | 5.84E-08 | 5.423728814 | -22.71954935 | 1.06E-10 |
| rs1361423 | 2.94E-05 | -4.178319649 | 5.84E-08 | 5.423728814 | -22.72351541 | 1.06E-10 |
| rs1361424 | 2.95E-05 | -4.177589881 | 6.12E-08 | 5.415254237 | -22.6840257 | 1.10E-10 |
| rs1369811 | 4.41E-05 | -4.084897946 | 7.53E-06 | 4.47826087 | -18.34140165 | 7.13E-09 |
| rs1380068 | 2.98E-04 | -3.61735355 | 9.07E-13 | 7.143939394 | -25.9261675 | 4.91E-12 |
| rs138584427 | 2.33E-06 | 4.722338181 | 1.30E-05 | 4.359375 | 20.53257932 | 8.66E-10 |
| rs139683250 | 5.20E-07 | 5.018752512 | 1.25E-05 | 4.369127517 | 21.86982871 | 2.40E-10 |
| rs139696728 | 4.52E-07 | 5.045667647 | 4.51E-06 | 4.586419753 | 23.0809186 | 7.50E-11 |
| rs139940087 | 3.76E-04 | 3.556331288 | 2.03E-06 | -4.750902527 | -16.94194596 | 2.75E-08 |
| rs139951459 | 2.80E-06 | 4.68513625 | 1.68E-06 | 4.7890625 | 22.37887726 | 1.47E-10 |
| rs140993356 | 1.21E-06 | 4.854696464 | 6.18E-06 | -4.520123839 | -22.0015073 | 2.11E-10 |
| rs141130394 | 3.81E-04 | 3.55302779 | 2.27E-06 | 4.727598566 | 16.75167633 | 3.30E-08 |
| rs141402489 | 6.10E-09 | -5.814063462 | 1.15E-18 | -8.819875776 | 51.13376321 | 1.84E-22 |
| rs1417015 | 1.43E-06 | -4.820993573 | 9.95E-08 | -5.327731092 | 25.61763276 | 6.60E-12 |
| rs1417016 | 1.43E-06 | -4.821015819 | 1.04E-07 | 5.319327731 | -25.71212202 | 6.03E-12 |
| rs142499157 | 5.76E-04 | -3.442459751 | 1.25E-06 | 4.848184818 | -16.73602129 | 3.35E-08 |
| rs142767852 | 3.76E-04 | 3.556558446 | 2.18E-06 | -4.736462094 | -16.89148956 | 2.88E-08 |
| rs142779375 | 4.35E-04 | 3.518167631 | 1.42E-06 | -4.822695035 | -17.01375835 | 2.56E-08 |
| rs1430787 | 6.22E-06 | -4.518808557 | 6.80E-06 | 4.5 | -20.38795177 | 9.96E-10 |
| rs1430788 | 5.25E-06 | -4.554534235 | 8.89E-06 | -4.442622951 | 20.18128951 | 1.21E-09 |
| rs1430789 | 8.14E-05 | -3.940183536 | 2.64E-06 | 4.697183099 | -18.5570359 | 5.79E-09 |
| rs1430790 | 8.94E-05 | -3.917643123 | 2.92E-06 | 4.676056338 | -18.36790057 | 6.95E-09 |
| rs1430791 | 9.00E-05 | -3.915986293 | 2.83E-06 | 4.683098592 | -18.38779984 | 6.82E-09 |
| rs1430792 | 9.00E-05 | -3.916211573 | 3.13E-06 | 4.661971831 | -18.3058616 | 7.38E-09 |
| rs143092818 | 4.35E-04 | 3.51811092 | 1.43E-06 | 4.821428571 | 16.91586022 | 2.82E-08 |
| rs1436098 | 4.03E-05 | -4.106022278 | 8.66E-06 | -4.448275862 | 18.21693048 | 8.04E-09 |
| rs1436099 | 4.34E-05 | -4.088523702 | 8.50E-06 | -4.452173913 | 18.15517095 | 8.53E-09 |
| rs144100226 | 4.06E-04 | 3.536218573 | 1.48E-06 | 4.813620072 | 16.97548418 | 2.66E-08 |
| rs144229896 | 3.71E-04 | 3.560076891 | 2.00E-06 | 4.753571429 | 16.87708037 | 2.92E-08 |
| rs144444583 | 2.85E-03 | 2.983423672 | 3.57E-09 | -5.903030303 | -17.668558 | 1.36E-08 |
| rs144737447 | 5.02E-13 | -7.224746549 | 6.01E-05 | -4.012531328 | 28.90042351 | 2.86E-13 |
| rs145052476 | 6.82E-06 | 4.499315967 | 2.20E-04 | 3.694915254 | 16.58038665 | 3.89E-08 |
| rs145163454 | 1.02E-12 | -7.128093613 | 5.96E-05 | 4.014354067 | -28.70238918 | 3.45E-13 |
| rs147230474 | 4.75E-07 | 5.036029747 | 5.48E-06 | 4.545454545 | 22.83102758 | 9.54E-11 |
| rs147631975 | 9.25E-06 | -4.434071684 | 2.20E-07 | -5.181818182 | 22.91589716 | 8.79E-11 |
| rs1482061 | 5.08E-03 | 2.801661289 | 1.88E-10 | 6.370820669 | 17.78567434 | 1.22E-08 |
| rs148221695 | 3.81E-04 | 3.552982039 | 2.28E-06 | -4.726618705 | -16.83942128 | 3.03E-08 |
| rs149268820 | 4.13E-04 | 3.531348482 | 1.48E-06 | 4.814285714 | 16.9544285 | 2.71E-08 |
| rs149746679 | 2.77E-06 | 4.686952859 | 1.91E-06 | -4.762645914 | -22.38082836 | 1.47E-10 |
| rs150892220 | 4.35E-04 | 3.517843139 | 1.62E-06 | 4.796428571 | 16.82693928 | 3.07E-08 |
| rs151214526 | 3.32E-04 | 3.589319471 | 4.75E-06 | 4.575539568 | 16.37896861 | 4.73E-08 |
| rs1538109 | 6.70E-06 | -4.503152725 | 1.06E-04 | 3.877192982 | -17.50587952 | 1.59E-08 |
| rs1551641 | 4.74E-04 | -3.495048545 | 1.83E-13 | -7.360655738 | 25.63921624 | 6.47E-12 |
| rs1551642 | 4.79E-04 | -3.492412849 | 1.43E-13 | 7.393442623 | -25.90854776 | 5.00E-12 |
| rs1551643 | 4.74E-04 | -3.495041974 | 2.07E-13 | -7.344262295 | 25.58218696 | 6.83E-12 |
| rs1551644 | 4.74E-04 | -3.494971268 | 5.56E-13 | 7.2109375 | -25.28614738 | 9.07E-12 |
| rs1551645 | 1.80E-04 | -3.745578334 | 1.43E-13 | 7.393442623 | -27.78272086 | 8.32E-13 |
| rs1553002 | 1.67E-05 | 4.304434966 | 5.33E-12 | -6.896551724 | -29.77237265 | 1.24E-13 |
| rs1630084 | 7.87E-06 | -4.46855433 | 9.48E-05 | -3.903508772 | 17.39713044 | 1.77E-08 |
| rs16826658 | 5.02E-04 | 3.479778574 | 7.04E-10 | -6.165217391 | -21.51926673 | 3.36E-10 |
| rs1702136 | 1.89E-05 | 4.277624988 | 4.90E-05 | 4.060150376 | 17.32244196 | 1.90E-08 |
| rs17081262 | 2.30E-06 | 4.725325922 | 1.15E-05 | 4.38671875 | 20.67446352 | 7.56E-10 |
| rs17081270 | 2.30E-06 | 4.725532479 | 1.24E-05 | -4.37109375 | -20.7100644 | 7.31E-10 |
| rs17081341 | 4.79E-03 | 2.820900957 | 7.43E-10 | -6.156626506 | -17.42731481 | 1.72E-08 |
| rs17082233 | 9.19E-05 | -3.911131937 | 8.57E-06 | 4.45035461 | -17.45193858 | 1.68E-08 |
| rs17082236 | 1.20E-04 | -3.846949337 | 1.28E-05 | -4.364285714 | 16.74504829 | 3.32E-08 |
| rs17082381 | 8.98E-06 | -4.440347247 | 8.11E-14 | -7.468468468 | 33.0641133 | 5.36E-15 |
| rs1716022 | 7.50E-06 | -4.478992462 | 1.14E-04 | -3.859649123 | 17.24175074 | 2.06E-08 |
| rs1716023 | 7.47E-06 | -4.4798899 | 1.02E-04 | 3.885964912 | -17.45479781 | 1.67E-08 |
| rs1716024 | 7.22E-06 | -4.487104391 | 1.14E-04 | -3.859649123 | 17.27296506 | 2.00E-08 |
| rs17195304 | 9.20E-07 | -4.908031335 | 6.05E-14 | 7.506976744 | -36.94990624 | 1.32E-16 |
| rs17215781 | 5.83E-06 | -4.532545528 | 2.60E-13 | 7.313901345 | -33.24761864 | 4.50E-15 |
| rs1735537 | 7.55E-06 | 4.477714861 | 2.74E-04 | -3.638461538 | -16.33562811 | 4.93E-08 |
| rs17622950 | 1.40E-06 | -4.824814393 | 9.95E-08 | 5.327731092 | -25.77303783 | 5.69E-12 |
| rs17623478 | 2.97E-05 | -4.175854487 | 6.54E-08 | 5.403361345 | -22.62477753 | 1.16E-10 |
| rs1765134 | 2.01E-07 | -5.198853571 | 1.24E-03 | -3.229508197 | 16.74088017 | 3.33E-08 |
| rs1765135 | 2.02E-07 | -5.197680593 | 1.21E-03 | 3.237704918 | -16.87769931 | 2.92E-08 |
| rs1765142 | 2.28E-08 | -5.589253048 | 5.12E-04 | -3.474576271 | 19.36378922 | 2.66E-09 |
| rs17694317 | 4.73E-05 | -4.068360085 | 8.16E-06 | 4.460869565 | -18.19620615 | 8.20E-09 |
| rs17711225 | 2.54E-04 | -3.658533992 | 1.07E-12 | -7.121212121 | 25.96957063 | 4.71E-12 |
| rs17711320 | 1.81E-04 | -3.744319461 | 1.27E-13 | 7.409448819 | -27.83364358 | 7.92E-13 |
| rs17773240 | 1.80E-04 | -3.74489706 | 1.35E-13 | 7.401574803 | -27.8082889 | 8.12E-13 |
| rs17773813 | 8.98E-04 | -3.320772659 | 8.72E-14 | -7.459016393 | 24.68270547 | 1.62E-11 |
| rs17803505 | 6.12E-07 | -4.987498354 | 1.26E-14 | 7.70952381 | -38.56173529 | 2.84E-17 |
| rs17803970 | 9.77E-07 | -4.896287981 | 1.04E-14 | 7.734597156 | -37.98063672 | 4.95E-17 |
| rs1782508 | 1.00E-05 | -4.416138382 | 2.27E-04 | 3.686440678 | -16.32321036 | 4.99E-08 |
| rs1782509 | 7.09E-06 | -4.490892742 | 8.82E-05 | -3.921052632 | 17.5626762 | 1.51E-08 |
| rs1782510 | 7.47E-06 | -4.4798899 | 9.83E-05 | 3.894736842 | -17.49418459 | 1.61E-08 |
| rs17837951 | 4.68E-04 | 3.498397378 | 2.67E-20 | 9.231292517 | 32.16753042 | 1.26E-14 |
| rs1808771 | 2.95E-05 | -4.177589881 | 5.06E-08 | -5.449152542 | 22.70283895 | 1.08E-10 |
| rs1819673 | 2.96E-05 | -4.176462106 | 3.99E-08 | 5.491525424 | -22.9975397 | 8.13E-11 |
| rs1819675 | 4.80E-05 | -4.06539593 | 8.92E-08 | -5.347457627 | 21.68068461 | 2.88E-10 |
| rs1825368 | 2.28E-05 | 4.23555034 | 8.76E-10 | 6.130434783 | 25.89334932 | 5.07E-12 |
| rs1865731 | 4.44E-05 | -4.083543171 | 7.84E-06 | 4.469565217 | -18.29970906 | 7.42E-09 |
| rs1874415 | 2.81E-06 | 4.684033441 | 1.44E-06 | 4.819607843 | 22.51630196 | 1.29E-10 |
| rs1890100 | 4.29E-06 | -4.596929707 | 9.50E-08 | 5.336065574 | -24.59454347 | 1.76E-11 |
| rs1890825 | 4.96E-05 | -4.057605921 | 1.08E-07 | -5.313559322 | 21.50203591 | 3.41E-10 |
| rs1890826 | 4.79E-05 | -4.065429718 | 7.39E-08 | 5.381355932 | -21.93714632 | 2.25E-10 |
| rs1894692 | 4.25E-14 | 7.553245667 | 1.15E-04 | 3.857482185 | 29.0426615 | 2.49E-13 |
| rs1898174 | 4.79E-05 | -4.065521556 | 8.92E-08 | -5.347457627 | 21.68135506 | 2.87E-10 |
| rs1903068 | 8.46E-04 | -3.337166632 | 7.23E-14 | 7.483606557 | -25.06199759 | 1.12E-11 |
| rs1933347 | 8.84E-06 | 4.443860034 | 2.35E-04 | -3.677966102 | -16.38798473 | 4.69E-08 |
| rs1933348 | 5.28E-06 | -4.553227415 | 8.20E-05 | -3.938596491 | 17.88605975 | 1.11E-08 |
| rs1933349 | 5.28E-06 | -4.553227415 | 8.20E-05 | -3.938596491 | 17.88605975 | 1.11E-08 |
| rs1938315 | 4.83E-05 | -4.063869805 | 6.73E-08 | 5.398305085 | -21.99785373 | 2.12E-10 |
| rs1971256 | 6.76E-06 | 4.501044535 | 1.09E-17 | -8.564285714 | -38.6708823 | 2.56E-17 |
| rs2001574 | 9.47E-07 | 4.90229041 | 1.22E-06 | 4.852713178 | 23.72736098 | 4.04E-11 |
| rs2001575 | 9.89E-07 | 4.893761833 | 1.13E-06 | 4.868217054 | 23.76175893 | 3.91E-11 |
| rs2040445 | 6.34E-13 | 7.192960645 | 9.91E-05 | -3.892857143 | -28.08882013 | 6.21E-13 |
| rs2046371 | 4.15E-05 | 4.099104555 | 2.56E-08 | 5.568965517 | 22.7654101 | 1.02E-10 |
| rs2065940 | 6.60E-06 | -4.506204473 | 1.18E-04 | -3.850877193 | 17.30702065 | 1.93E-08 |
| rs2082917 | 4.72E-05 | -4.069110172 | 8.16E-06 | 4.460869565 | -18.1995602 | 8.17E-09 |
| rs2117911 | 4.54E-05 | -4.078292317 | 8.16E-06 | 4.460869565 | -18.24061864 | 7.86E-09 |
| rs2128945 | 2.15E-05 | 4.248635602 | 1.09E-09 | -6.095652174 | -25.97056443 | 4.71E-12 |
| rs2226016 | 5.14E-05 | -4.049285306 | 7.39E-08 | -5.381355932 | 21.7314934 | 2.74E-10 |
| rs2226272 | 4.83E-05 | -4.063869805 | 7.39E-08 | 5.381355932 | -21.92873523 | 2.27E-10 |
| rs2227246 | 1.82E-12 | 7.047990484 | 1.93E-04 | 3.727941176 | 26.19155565 | 3.81E-12 |
| rs2235529 | 5.74E-06 | 4.535638402 | 4.58E-24 | 10.11842105 | 45.73305397 | 3.11E-20 |
| rs2239702 | 1.78E-04 | -3.74844317 | 1.49E-12 | -7.07518797 | 26.4373008 | 3.01E-12 |
| rs2268177 | 4.81E-04 | 3.491198841 | 1.09E-20 | -9.326530612 | -32.6906605 | 7.66E-15 |
| rs2268179 | 4.80E-04 | 3.491642521 | 9.02E-21 | 9.346938776 | 32.50622472 | 9.13E-15 |
| rs2421985 | 2.22E-06 | 4.73190608 | 1.54E-07 | -5.247787611 | -24.89749002 | 1.32E-11 |
| rs2460713 | 4.79E-05 | -4.065503979 | 8.92E-08 | -5.347457627 | 21.68126125 | 2.87E-10 |
| rs2473290 | 1.63E-03 | -3.150699173 | 2.13E-11 | 6.696969697 | -21.1718994 | 4.69E-10 |
| rs2501256 | 1.15E-03 | -3.251923304 | 2.36E-08 | -5.583333333 | 18.10211098 | 8.98E-09 |
| rs2678166 | 7.17E-12 | 6.854151249 | 3.97E-04 | 3.542334096 | 24.2020294 | 2.56E-11 |
| rs2761183 | 4.75E-05 | 4.067370094 | 4.12E-06 | 4.605263158 | 18.68207828 | 5.14E-09 |
| rs2807365 | 4.90E-04 | 3.485999131 | 1.18E-10 | -6.441666667 | -22.52593999 | 1.28E-10 |
| rs2819530 | 5.38E-05 | 4.038655852 | 9.84E-07 | -4.894736842 | -19.82094341 | 1.72E-09 |
| rs28385620 | 1.89E-02 | 2.346603822 | 7.03E-14 | -7.487341772 | -17.65044159 | 1.39E-08 |
| rs28411232 | 1.81E-04 | -3.74435862 | 1.43E-13 | 7.393700787 | -27.77466264 | 8.38E-13 |
| rs28517654 | 9.94E-05 | -3.892159828 | 2.62E-13 | 7.3125 | -28.55133381 | 3.99E-13 |
| rs28584303 | 9.71E-05 | -3.897668836 | 2.08E-13 | -7.34375 | 28.53332584 | 4.06E-13 |
| rs2861640 | 3.50E-05 | -4.138331289 | 2.26E-06 | -4.728571429 | 19.51690332 | 2.30E-09 |
| rs2861644 | 8.86E-05 | -3.919877262 | 2.55E-06 | 4.704225352 | -18.4891358 | 6.18E-09 |
| rs2861645 | 8.86E-05 | -3.919814088 | 3.47E-06 | -4.64084507 | 18.14312561 | 8.63E-09 |
| rs2861648 | 2.30E-05 | -4.233393914 | 2.99E-06 | -4.671428571 | 19.7241687 | 1.88E-09 |
| rs2861681 | 8.51E-05 | -3.929465085 | 4.31E-06 | 4.595744681 | -18.1067448 | 8.94E-09 |
| rs2861686 | 9.36E-05 | -3.90664646 | 4.31E-06 | 4.595744681 | -18.00164172 | 9.89E-09 |
| rs2861687 | 9.36E-05 | -3.90664646 | 4.03E-06 | 4.609929078 | -18.05722625 | 9.37E-09 |
| rs2861689 | 9.43E-05 | -3.90474894 | 4.31E-06 | -4.595744681 | 17.89780199 | 1.09E-08 |
| rs2861694 | 1.03E-05 | -4.410145594 | 1.20E-05 | 4.37704918 | -19.35403478 | 2.69E-09 |
| rs2861695 | 7.21E-05 | -3.969307722 | 5.85E-06 | 4.531914894 | -18.03614055 | 9.56E-09 |
| rs2861698 | 9.42E-05 | -3.90511273 | 2.83E-06 | 4.683098592 | -18.33676634 | 7.16E-09 |
| rs2861700 | 6.29E-05 | -4.001734015 | 2.86E-06 | -4.680851064 | 18.6820643 | 5.14E-09 |
| rs2861701 | 4.86E-05 | -4.062338814 | 2.49E-06 | 4.709219858 | -19.18114941 | 3.18E-09 |
| rs2861703 | 8.78E-05 | -3.922150977 | 2.38E-06 | 4.718309859 | -18.55527071 | 5.80E-09 |
| rs28680424 | 1.41E-03 | -3.193519202 | 1.15E-12 | 7.111111111 | -22.78907534 | 9.93E-11 |
| rs28753985 | 2.70E-07 | -5.143435967 | 4.17E-04 | -3.52892562 | 18.1000558 | 8.99E-09 |
| rs2881823 | 5.46E-04 | -3.457124196 | 3.31E-08 | 5.524115756 | -19.15320978 | 3.26E-09 |
| rs28838369 | 1.28E-03 | -3.219896757 | 1.03E-12 | 7.126984127 | -23.02827684 | 7.89E-11 |
| rs2884134 | 4.83E-05 | -4.063869805 | 7.05E-08 | -5.389830508 | 21.84414408 | 2.46E-10 |
| rs2902007 | 3.14E-05 | -4.16309412 | 2.79E-06 | 4.685714286 | -19.5585698 | 2.21E-09 |
| rs2902008 | 6.60E-05 | -3.990299959 | 3.76E-06 | 4.624113475 | -18.50050094 | 6.12E-09 |
| rs2902023 | 8.56E-05 | -3.928043496 | 4.46E-06 | 4.588652482 | -18.07225307 | 9.24E-09 |
| rs2902025 | 1.04E-04 | -3.881240709 | 4.03E-06 | 4.609929078 | -17.93984805 | 1.05E-08 |
| rs2902026 | 6.64E-06 | -4.504886423 | 1.13E-05 | -4.390625 | 19.72766364 | 1.88E-09 |
| rs34487664 | 8.64E-06 | 4.448568743 | 2.43E-04 | -3.669491525 | -16.36757666 | 4.78E-08 |
| rs34609938 | 6.82E-06 | 4.499294355 | 1.92E-04 | 3.728813559 | 16.73249751 | 3.36E-08 |
| rs35065475 | 5.23E-06 | 4.555480709 | 1.82E-07 | 5.217391304 | 23.70516407 | 4.12E-11 |
| rs35078732 | 4.61E-05 | -4.074472677 | 6.66E-06 | 4.504347826 | -18.40120073 | 6.73E-09 |
| rs35429109 | 7.20E-04 | 3.381712759 | 9.24E-19 | 8.843971631 | 29.79075939 | 1.22E-13 |
| rs35458722 | 8.64E-06 | 4.448595162 | 2.06E-04 | -3.711864407 | -16.55658393 | 3.98E-08 |
| rs35569312 | 1.43E-03 | 3.187621273 | 2.28E-11 | 6.687116564 | 21.24438898 | 4.37E-10 |
| rs3596 | 8.10E-04 | 3.349402118 | 5.00E-08 | -5.451327434 | -18.31233429 | 7.33E-09 |
| rs36019875 | 5.79E-03 | 2.759409682 | 4.24E-16 | -8.131386861 | -22.53438559 | 1.27E-10 |
| rs36069217 | 4.97E-06 | -4.566000548 | 4.88E-13 | 7.228699552 | -33.10205089 | 5.17E-15 |
| rs36146043 | 4.74E-04 | -3.494886441 | 2.20E-13 | 7.336065574 | -25.72522622 | 5.96E-12 |
| rs3734801 | 2.30E-06 | 4.72482227 | 2.36E-05 | -4.22745098 | -20.02664514 | 1.41E-09 |
| rs3754496 | 6.15E-04 | 3.424741392 | 3.45E-20 | 9.204081633 | 31.39571754 | 2.63E-14 |
| rs3757312 | 8.62E-04 | 3.332204291 | 2.40E-07 | 5.165413534 | 17.16292807 | 2.22E-08 |
| rs3757314 | 8.53E-04 | 3.33488569 | 2.30E-07 | -5.172932331 | -17.30078373 | 1.94E-08 |
| rs3757315 | 8.46E-04 | 3.337291039 | 2.21E-07 | 5.180451128 | 17.23914081 | 2.06E-08 |
| rs3765350 | 8.36E-05 | 3.933967976 | 5.54E-14 | -7.518518519 | -29.6719565 | 1.37E-13 |
| rs3768579 | 1.36E-03 | 3.202969316 | 3.23E-19 | 8.960526316 | 28.58209586 | 3.87E-13 |
| rs3775010 | 1.49E-07 | -5.253314535 | 2.33E-04 | 3.680672269 | -19.38965665 | 2.60E-09 |
| rs3778608 | 1.24E-04 | 3.837371712 | 1.97E-09 | -6 | -23.09070954 | 7.43E-11 |
| rs3800272 | 1.25E-04 | 3.835449401 | 1.60E-09 | 6.034482759 | 23.07826642 | 7.52E-11 |
| rs3800273 | 2.16E-05 | 4.247590775 | 7.04E-10 | 6.165217391 | 26.11421351 | 4.10E-12 |
| rs3800274 | 1.52E-05 | 4.325982513 | 8.63E-06 | -4.448818898 | -19.29598984 | 2.84E-09 |
| rs3800275 | 9.35E-07 | 4.90485451 | 4.50E-05 | -4.08045977 | -20.06742241 | 1.35E-09 |
| rs3800276 | 8.48E-07 | 4.92406085 | 4.40E-05 | 4.085271318 | 20.06273986 | 1.36E-09 |
| rs3800278 | 9.48E-07 | 4.902188349 | 4.57E-05 | 4.076628352 | 19.93138766 | 1.54E-09 |
| rs3800280 | 9.36E-07 | 4.904602037 | 4.20E-05 | -4.096153846 | -20.14353046 | 1.26E-09 |
| rs3806982 | 2.28E-05 | 4.23555034 | 8.76E-10 | 6.130434783 | 25.89334932 | 5.07E-12 |
| rs3820282 | 8.46E-06 | 4.453092105 | 7.36E-24 | 10.07189542 | 44.69282405 | 8.34E-20 |
| rs3820687 | 2.97E-03 | 2.970893012 | 1.15E-11 | -6.786764706 | -20.23465186 | 1.15E-09 |
| rs3847631 | 4.67E-05 | -4.071721839 | 8.16E-06 | 4.460869565 | -18.21123838 | 8.08E-09 |
| rs3847632 | 4.76E-05 | -4.067199617 | 6.66E-06 | 4.504347826 | -18.3683627 | 6.95E-09 |
| rs3847633 | 4.83E-05 | -4.063869805 | 1.13E-05 | 4.390243902 | -17.88829494 | 1.10E-08 |
| rs3847635 | 4.83E-05 | -4.063869805 | 7.75E-08 | 5.372881356 | -21.89417626 | 2.34E-10 |
| rs3847637 | 4.82E-05 | -4.064040851 | 6.73E-08 | -5.398305085 | 21.8793859 | 2.38E-10 |
| rs3849798 | 9.58E-04 | 3.302665495 | 3.07E-07 | -5.119402985 | -16.95631902 | 2.71E-08 |
| rs3858429 | 3.69E-13 | -7.266611441 | 9.74E-22 | -9.579617834 | 69.42281243 | 5.54E-30 |
| rs3858477 | 4.83E-05 | -4.063824755 | 8.12E-08 | -5.36440678 | 21.74094093 | 2.71E-10 |
| rs3858478 | 4.83E-05 | -4.063824755 | 7.39E-08 | -5.381355932 | 21.80958166 | 2.54E-10 |
| rs3858479 | 4.79E-05 | -4.065544451 | 7.05E-08 | -5.389830508 | 21.85315239 | 2.44E-10 |
| rs3858480 | 4.82E-05 | -4.064098753 | 7.39E-08 | 5.381355932 | -21.92996972 | 2.26E-10 |
| rs3858481 | 4.83E-05 | -4.063869805 | 7.53E-08 | -5.378151261 | 21.79684524 | 2.57E-10 |
| rs3858482 | 4.83E-05 | -4.063869805 | 7.89E-08 | 5.369747899 | -21.8813982 | 2.37E-10 |
| rs3858484 | 5.03E-05 | -4.054409251 | 6.73E-08 | 5.398305085 | -21.94668201 | 2.23E-10 |
| rs3860810 | 9.84E-04 | 3.295052038 | 2.95E-07 | 5.126865672 | 16.84484258 | 3.02E-08 |
| rs3886880 | 4.76E-05 | -4.067176842 | 6.66E-06 | -4.504347826 | 18.271949 | 7.62E-09 |
| rs3887841 | 2.12E-05 | 4.251894963 | 7.90E-05 | -3.947368421 | -16.82792045 | 3.07E-08 |
| rs3888239 | 2.09E-06 | 4.744105963 | 3.01E-07 | -5.122807018 | -24.36704413 | 2.19E-11 |
| rs3897026 | 5.53E-06 | -4.543632499 | 7.90E-05 | 3.947368421 | -17.98287881 | 1.01E-08 |
| rs3900024 | 1.12E-03 | 3.258690648 | 2.72E-07 | -5.141791045 | -16.80407189 | 3.14E-08 |
| rs3908935 | 4.83E-05 | -4.063847999 | 8.51E-08 | 5.355932203 | -21.82494186 | 2.50E-10 |
| rs3920498 | 2.88E-04 | 3.625817376 | 1.95E-16 | 8.225352113 | 29.7181813 | 1.31E-13 |
| rs3971300 | 1.07E-04 | 3.873707728 | 2.16E-14 | -7.640625 | -29.69369867 | 1.34E-13 |
| rs4071558 | 3.70E-13 | -7.266149285 | 9.74E-22 | -9.579617834 | 69.41839391 | 5.56E-30 |
| rs4071559 | 3.70E-13 | -7.265998727 | 1.86E-21 | -9.512658228 | 68.93209421 | 8.81E-30 |
| rs4071563 | 3.68E-13 | -7.266658529 | 9.16E-22 | -9.585987261 | 69.46938778 | 5.30E-30 |
| rs4120817 | 5.04E-09 | -5.845683288 | 2.83E-19 | -8.975308642 | 52.31716715 | 6.00E-23 |
| rs4120820 | 6.57E-06 | -4.507044347 | 7.08E-05 | -3.973684211 | 17.86248912 | 1.13E-08 |
| rs4120821 | 5.97E-06 | -4.527343951 | 7.62E-05 | -3.956140351 | 17.86366837 | 1.13E-08 |
| rs41269028 | 3.28E-04 | 3.591867033 | 2.77E-06 | 4.68705036 | 16.78978547 | 3.18E-08 |
| rs41408948 | 2.15E-04 | -3.70032426 | 1.41E-12 | -7.082706767 | 26.12499976 | 4.06E-12 |
| rs4141819 | 1.04E-05 | -4.409510897 | 7.63E-06 | -4.475409836 | 19.68289362 | 1.96E-09 |
| rs4233939 | 3.04E-05 | -4.170775105 | 1.02E-06 | 4.887323944 | -20.43804237 | 9.49E-10 |
| rs4260227 | 2.39E-05 | -4.225067134 | 4.52E-06 | -4.585714286 | 19.32424891 | 2.77E-09 |
| rs4267043 | 7.69E-06 | 4.473622491 | 2.20E-04 | 3.694915254 | 16.48575221 | 4.26E-08 |
| rs4285851 | 4.74E-05 | -4.067936562 | 8.16E-06 | -4.460869565 | 18.09900462 | 9.00E-09 |
| rs4313561 | 2.91E-05 | -4.18078746 | 5.06E-08 | 5.449152542 | -22.84358057 | 9.42E-11 |
| rs4337008 | 4.83E-05 | -4.063824755 | 8.51E-08 | -5.355932203 | 21.70662029 | 2.81E-10 |
| rs4349334 | 2.72E-05 | -4.195528383 | 2.99E-06 | -4.671428571 | 19.54769867 | 2.23E-09 |
| rs4362541 | 6.00E-05 | -4.012678907 | 4.94E-06 | 4.567375887 | -18.37586548 | 6.90E-09 |
| rs4381768 | 3.18E-05 | -4.159961575 | 4.39E-06 | 4.591836735 | -19.15218898 | 3.27E-09 |
| rs4396264 | 3.01E-05 | -4.172666328 | 5.06E-08 | 5.449152542 | -22.79923828 | 9.83E-11 |
| rs4410279 | 7.39E-05 | -3.963346094 | 2.49E-06 | 4.709219858 | -18.71392898 | 4.98E-09 |
| rs4427542 | 6.68E-06 | -4.503497911 | 1.10E-04 | -3.868421053 | 17.37546203 | 1.81E-08 |
| rs4471514 | 8.91E-04 | 3.322895456 | 5.25E-08 | 5.442477876 | 18.03174416 | 9.61E-09 |
| rs4472361 | 5.99E-06 | -4.526593148 | 1.24E-07 | -5.286885246 | 23.86840688 | 3.53E-11 |
| rs4512795 | 4.83E-05 | -4.063869805 | 7.05E-08 | -5.389830508 | 21.84414408 | 2.46E-10 |
| rs4516787 | 8.44E-04 | -3.33790216 | 1.83E-13 | 7.360655738 | -24.65472155 | 1.66E-11 |
| rs4531442 | 8.63E-06 | 4.448908537 | 2.43E-04 | -3.669491525 | -16.36882749 | 4.77E-08 |
| rs4533457 | 1.01E-04 | -3.888210576 | 2.05E-06 | 4.748251748 | -18.51157345 | 6.05E-09 |
| rs4619594 | 2.25E-04 | -3.688857172 | 5.48E-06 | 4.545454545 | -16.81245262 | 3.11E-08 |
| rs4654783 | 6.96E-04 | -3.391016673 | 9.60E-09 | 5.737704918 | -19.51486411 | 2.30E-09 |
| rs4654785 | 3.61E-04 | 3.566861981 | 1.95E-14 | -7.653846154 | -27.393627 | 1.21E-12 |
| rs4671192 | 1.01E-04 | -3.888934214 | 4.31E-06 | 4.595744681 | -17.92005967 | 1.07E-08 |
| rs4671806 | 1.11E-04 | -3.865715738 | 1.23E-05 | 4.371621622 | -16.94408764 | 2.74E-08 |
| rs4671811 | 3.02E-05 | -4.171961332 | 2.99E-06 | -4.671428571 | 19.43786395 | 2.48E-09 |
| rs4671812 | 5.99E-05 | -4.013038271 | 5.47E-06 | -4.546099291 | 18.19571766 | 8.20E-09 |
| rs4671813 | 9.49E-05 | -3.90330768 | 3.89E-06 | 4.617021277 | -18.0695693 | 9.26E-09 |
| rs4671817 | 8.11E-05 | -3.94105149 | 2.46E-06 | 4.711267606 | -18.61680321 | 5.47E-09 |
| rs475305 | 4.68E-05 | -4.071176293 | 9.35E-08 | 5.338983051 | -21.79502946 | 2.58E-10 |
| rs4762308 | 8.64E-04 | 3.331566498 | 4.76E-08 | 5.460176991 | 18.13757488 | 8.67E-09 |
| rs4762326 | 8.84E-04 | 3.325061598 | 5.25E-08 | 5.442477876 | 18.0435146 | 9.50E-09 |
| rs484686 | 1.45E-03 | 3.184347707 | 3.15E-07 | -5.114035088 | -16.33242973 | 4.94E-08 |
| rs4869729 | 8.40E-04 | 3.339303649 | 2.30E-07 | -5.172932331 | -17.32367625 | 1.90E-08 |
| rs4869732 | 5.07E-03 | 2.802255097 | 1.57E-10 | 6.398176292 | 17.86565432 | 1.13E-08 |
| rs4869733 | 1.89E-08 | 5.621340248 | 1.48E-14 | 7.689320388 | 43.10596161 | 3.77E-19 |
| rs4869734 | 5.17E-03 | 2.796240934 | 1.25E-10 | 6.433333333 | 17.92493681 | 1.06E-08 |
| rs4869735 | 5.32E-03 | 2.786998493 | 7.82E-10 | -6.148484848 | -17.19551807 | 2.15E-08 |
| rs4869758 | 2.19E-06 | 4.735337265 | 6.00E-07 | -4.991304348 | -23.69756207 | 4.15E-11 |
| rs4869759 | 5.16E-06 | 4.558211723 | 3.17E-07 | -5.113043478 | -23.36784112 | 5.70E-11 |
| rs4869761 | 7.68E-05 | 3.954273189 | 2.94E-06 | 4.675213675 | 18.43817645 | 6.49E-09 |
| rs4869762 | 5.45E-06 | 4.546614471 | 3.48E-07 | 5.095652174 | 23.10714749 | 7.32E-11 |
| rs4870016 | 1.00E-03 | 3.289437964 | 2.32E-07 | 5.171641791 | 16.96279448 | 2.69E-08 |
| rs4870021 | 4.00E-03 | 2.878383068 | 1.42E-11 | 6.75625 | 19.37669425 | 2.63E-09 |
| rs4870022 | 4.47E-08 | 5.471347073 | 5.50E-14 | -7.519417476 | -41.25469033 | 2.19E-18 |
| rs4870023 | 4.47E-08 | 5.471234955 | 4.92E-14 | -7.533980583 | -41.33381101 | 2.03E-18 |
| rs4870024 | 1.82E-06 | 4.772866807 | 4.36E-17 | -8.402777778 | -40.22771374 | 5.82E-18 |
| rs4870088 | 7.69E-05 | 3.953961851 | 1.50E-06 | 4.811965812 | 18.97574422 | 3.87E-09 |
| rs4870089 | 5.59E-06 | 4.541296309 | 2.76E-07 | 5.139130435 | 23.27697819 | 6.22E-11 |
| rs4870090 | 8.81E-05 | 3.921198658 | 2.10E-06 | -4.743589744 | -18.65020844 | 5.30E-09 |
| rs4870091 | 1.04E-04 | 3.881354811 | 2.19E-06 | 4.735042735 | 18.32949544 | 7.21E-09 |
| rs489096 | 7.89E-06 | -4.468257173 | 8.82E-05 | -3.921052632 | 17.47418548 | 1.64E-08 |
| rs490937 | 2.68E-07 | 5.144933733 | 1.04E-03 | -3.278688525 | -16.91741466 | 2.81E-08 |
| rs4922833 | 2.82E-05 | -4.187426368 | 5.84E-08 | -5.423728814 | 22.65023295 | 1.13E-10 |
| rs4922834 | 2.91E-05 | -4.180674801 | 5.06E-08 | 5.449152542 | -22.84296544 | 9.43E-11 |
| rs4923621 | 4.23E-05 | -4.094360371 | 9.99E-06 | -4.417391304 | 18.0390844 | 9.54E-09 |
| rs4923622 | 4.32E-05 | -4.089774851 | 6.39E-06 | 4.513043478 | -18.50595689 | 6.08E-09 |
| rs4923623 | 4.34E-05 | -4.088829439 | 8.50E-06 | -4.452173913 | 18.15652889 | 8.52E-09 |
| rs4923626 | 1.69E-06 | -4.787205651 | 1.50E-07 | 5.25210084 | -25.20908833 | 9.76E-12 |
| rs4923629 | 6.78E-06 | 4.500513754 | 2.43E-04 | 3.669491525 | 16.47062248 | 4.33E-08 |
| rs492828 | 5.29E-06 | -4.552785214 | 5.47E-05 | -4.034782609 | 18.32123834 | 7.27E-09 |
| rs494779 | 5.52E-06 | -4.544096569 | 5.47E-05 | -4.034782609 | 18.28628465 | 7.52E-09 |
| rs495590 | 7.34E-04 | 3.376672414 | 3.81E-07 | 5.07826087 | 17.09911508 | 2.36E-08 |
| rs506197 | 7.19E-06 | -4.488107277 | 9.14E-05 | 3.912280702 | -17.60520402 | 1.45E-08 |
| rs506306 | 7.19E-06 | -4.488107277 | 9.48E-05 | 3.903508772 | -17.56574475 | 1.50E-08 |
| rs518357 | 7.85E-06 | -4.469299751 | 7.08E-05 | -3.973684211 | 17.71294597 | 1.31E-08 |
| rs523875 | 6.27E-06 | -4.517052764 | 7.90E-05 | -3.947368421 | 17.78354352 | 1.22E-08 |
| rs532667 | 7.73E-06 | -4.472666839 | 7.90E-05 | -3.947368421 | 17.60885642 | 1.44E-08 |
| rs537263 | 7.47E-06 | -4.4798899 | 9.14E-05 | 3.912280702 | -17.57295877 | 1.49E-08 |
| rs538236 | 7.31E-06 | -4.484451063 | 9.48E-05 | -3.903508772 | 17.45899782 | 1.67E-08 |
| rs540948 | 5.66E-06 | -4.538663973 | 6.57E-05 | 3.991304348 | -18.16307487 | 8.46E-09 |
| rs543401 | 6.95E-06 | -4.495326878 | 8.82E-05 | 3.921052632 | -17.67305692 | 1.36E-08 |
| rs549346 | 6.04E-06 | -4.52482396 | 6.10E-05 | 4.00877193 | -18.18689177 | 8.27E-09 |
| rs550312 | 7.17E-06 | -4.488560708 | 7.62E-05 | 3.956140351 | -17.80430257 | 1.20E-08 |
| rs552501 | 2.97E-05 | -4.175381596 | 4.39E-08 | 5.474576271 | -22.92058154 | 8.75E-11 |
| rs553190 | 6.20E-06 | -4.519583493 | 7.90E-05 | -3.947368421 | 17.7935034 | 1.21E-08 |
| rs553406 | 6.31E-06 | -4.515723616 | 8.20E-05 | 3.938596491 | -17.83267851 | 1.16E-08 |
| rs555621 | 5.61E-06 | -4.540718299 | 5.67E-05 | 4.026086957 | -18.32960247 | 7.21E-09 |
| rs55692303 | 3.27E-04 | 3.592738795 | 2.13E-06 | 4.741007194 | 16.98705232 | 2.63E-08 |
| rs55747952 | 1.70E-06 | 4.786635204 | 3.39E-05 | 4.145299145 | 19.78974709 | 1.77E-09 |
| rs55938609 | 2.67E-06 | 4.694898851 | 1.97E-22 | 9.743589744 | 45.59253406 | 3.55E-20 |
| rs559519 | 1.65E-06 | 4.792473066 | 8.71E-05 | -3.923976608 | -18.85584229 | 4.34E-09 |
| rs560078 | 7.47E-06 | -4.479832699 | 8.20E-05 | 3.938596491 | -17.69089557 | 1.33E-08 |
| rs560109 | 7.20E-06 | -4.487683309 | 9.14E-05 | -3.912280702 | 17.51085336 | 1.59E-08 |
| rs56104760 | 5.35E-05 | 4.039852178 | 4.02E-18 | -8.678321678 | -35.1791845 | 7.13E-16 |
| rs56318008 | 2.76E-06 | 4.687579741 | 1.97E-22 | 9.743589744 | 45.52130895 | 3.80E-20 |
| rs56410019 | 3.30E-04 | 3.590716075 | 2.20E-06 | 4.73381295 | 16.95173797 | 2.72E-08 |
| rs571174 | 1.79E-07 | 5.219607802 | 1.43E-03 | -3.18852459 | -16.69187528 | 3.49E-08 |
| rs572883 | 7.26E-06 | -4.485896644 | 9.14E-05 | -3.912280702 | 17.50388434 | 1.60E-08 |
| rs57689380 | 1.17E-07 | 5.297434593 | 6.10E-06 | -4.522875817 | -24.02323994 | 3.04E-11 |
| rs57968001 | 1.34E-07 | 5.272940405 | 1.86E-05 | -4.280936455 | -22.63359104 | 1.15E-10 |
| rs580646 | 7.17E-06 | -4.488560708 | 7.62E-05 | 3.956140351 | -17.80430257 | 1.20E-08 |
| rs58415480 | 1.87E-03 | 3.109964447 | 4.88E-23 | -9.884146341 | -30.87993435 | 4.31E-14 |
| rs584336 | 7.98E-04 | 3.3534249 | 7.65E-16 | 8.059701493 | 26.92815392 | 1.88E-12 |
| rs58450883 | 2.66E-05 | -4.200808703 | 7.52E-08 | -5.378205128 | 22.53207418 | 1.27E-10 |
| rs586326 | 5.96E-06 | -4.52772974 | 4.89E-05 | -4.060869565 | 18.3382811 | 7.15E-09 |
| rs588929 | 4.68E-05 | -4.071176293 | 9.35E-08 | -5.338983051 | 21.67715025 | 2.89E-10 |
| rs58952168 | 8.45E-06 | 4.453536198 | 1.59E-05 | 4.31640625 | 19.17311074 | 3.20E-09 |
| rs590833 | 8.60E-06 | -4.449658113 | 8.82E-05 | -3.921052632 | 17.40147395 | 1.76E-08 |
| rs594982 | 7.02E-06 | -4.493029832 | 1.10E-04 | 3.868421053 | -17.42701013 | 1.72E-08 |
| rs595496 | 7.17E-06 | -4.488560708 | 7.90E-05 | 3.947368421 | -17.76483831 | 1.24E-08 |
| rs597235 | 4.59E-05 | 4.075539474 | 5.67E-05 | -4.026086957 | -16.45149881 | 4.41E-08 |
| rs600333 | 5.67E-06 | -4.538333307 | 6.33E-05 | -4 | 18.10560867 | 8.95E-09 |
| rs600768 | 5.78E-06 | -4.534254962 | 7.07E-05 | -3.973913043 | 17.97132981 | 1.02E-08 |
| rs601640 | 5.75E-06 | -4.535410035 | 5.47E-05 | -4.034782609 | 18.25133925 | 7.77E-09 |
| rs601681 | 5.65E-06 | -4.538953561 | 4.89E-05 | -4.060869565 | 18.38372684 | 6.84E-09 |
| rs6025 | 6.21E-14 | -7.503549031 | 1.84E-03 | -3.115662651 | 23.29238062 | 6.13E-11 |
| rs60275968 | 5.31E-03 | 2.787667754 | 8.33E-10 | 6.138554217 | 17.05293842 | 2.47E-08 |
| rs60781817 | 7.83E-06 | 4.469651296 | 2.27E-04 | 3.686440678 | 16.43332845 | 4.49E-08 |
| rs609896 | 7.18E-06 | -4.488141596 | 9.14E-05 | 3.912280702 | -17.60533869 | 1.45E-08 |
| rs611246 | 4.28E-06 | -4.597308719 | 7.62E-05 | 3.956140351 | -18.23581977 | 7.89E-09 |
| rs61436491 | 5.22E-03 | 2.792901541 | 8.33E-10 | -6.138554217 | -17.20396118 | 2.13E-08 |
| rs615577 | 7.87E-06 | -4.468630503 | 8.20E-05 | -3.938596491 | 17.55386226 | 1.52E-08 |
| rs6169 | 6.04E-06 | -4.524934611 | 1.10E-04 | -3.868421053 | 17.45813573 | 1.67E-08 |
| rs61768001 | 8.79E-06 | 4.444962632 | 5.27E-24 | -10.10457516 | -45.07409427 | 5.81E-20 |
| rs61778045 | 1.59E-03 | 3.156952329 | 1.23E-19 | 9.066225166 | 28.50133653 | 4.18E-13 |
| rs618611 | 6.15E-06 | -4.521268142 | 6.57E-05 | -3.99122807 | 17.99798081 | 9.92E-09 |
| rs61886116 | 7.35E-06 | 4.483205098 | 2.43E-04 | 3.669491525 | 16.40731138 | 4.60E-08 |
| rs621448 | 6.04E-06 | -4.524902044 | 6.11E-05 | -4.008695652 | 18.0912986 | 9.07E-09 |
| rs622356 | 7.82E-06 | -4.470026685 | 7.90E-05 | 3.947368421 | -17.6914601 | 1.33E-08 |
| rs623837 | 1.50E-06 | -4.810921613 | 1.31E-07 | -5.277310924 | 25.32222852 | 8.76E-12 |
| rs626869 | 7.77E-06 | -4.471455255 | 7.90E-05 | -3.947368421 | 17.60408799 | 1.45E-08 |
| rs633891 | 5.52E-04 | 3.454342632 | 6.63E-09 | 5.8 | 19.97573973 | 1.48E-09 |
| rs637542 | 4.68E-05 | -4.071176293 | 5.57E-08 | 5.43220339 | -22.1758613 | 1.79E-10 |
| rs639403 | 7.18E-06 | -4.488153036 | 8.82E-05 | -3.921052632 | 17.55196576 | 1.52E-08 |
| rs640748 | 7.76E-06 | -4.471792197 | 8.50E-05 | 3.929824561 | -17.6198162 | 1.43E-08 |
| rs6484476 | 4.83E-05 | -4.063869805 | 7.75E-08 | -5.372881356 | 21.77550296 | 2.63E-10 |
| rs6484477 | 6.32E-06 | -4.515537048 | 7.08E-05 | -3.973684211 | 17.89613648 | 1.09E-08 |
| rs6484479 | 3.69E-13 | -7.266428322 | 9.16E-22 | -9.585987261 | 69.46718539 | 5.31E-30 |
| rs6484481 | 6.50E-06 | -4.509452993 | 1.18E-04 | -3.850877193 | 17.31949209 | 1.91E-08 |
| rs6532516 | 1.22E-07 | -5.290484916 | 2.74E-04 | -3.638655462 | 19.19647784 | 3.13E-09 |
| rs6538618 | 8.53E-04 | 3.334850654 | 8.60E-08 | -5.353982301 | -17.90687283 | 1.08E-08 |
| rs6538622 | 9.18E-04 | 3.314520696 | 9.04E-08 | -5.345132743 | -17.76839306 | 1.24E-08 |
| rs6546317 | 8.71E-05 | -3.923890159 | 3.51E-06 | 4.638297872 | -18.24855539 | 7.80E-09 |
| rs6546320 | 8.95E-05 | -3.917468753 | 2.22E-06 | 4.732394366 | -18.58848041 | 5.62E-09 |
| rs6546322 | 8.95E-05 | -3.917468753 | 2.14E-06 | 4.73943662 | -18.61615562 | 5.47E-09 |
| rs6546324 | 6.64E-06 | -4.504819196 | 6.05E-06 | 4.524590164 | -20.43589931 | 9.51E-10 |
| rs6546325 | 7.37E-05 | -3.963862634 | 3.39E-06 | -4.645390071 | 18.36505615 | 6.97E-09 |
| rs654669 | 7.44E-06 | -4.480639431 | 8.20E-05 | 3.938596491 | -17.69408244 | 1.33E-08 |
| rs6557132 | 1.88E-04 | 3.735061536 | 7.66E-08 | -5.375 | -20.13210665 | 1.27E-09 |
| rs6557134 | 1.04E-03 | 3.280778196 | 2.23E-07 | -5.179104478 | -17.04075252 | 2.50E-08 |
| rs6557135 | 1.50E-04 | 3.790841757 | 5.80E-08 | -5.425 | -20.62272567 | 7.94E-10 |
| rs6557138 | 1.27E-04 | 3.832442902 | 2.86E-09 | 5.939655172 | 22.69821458 | 1.08E-10 |
| rs6557139 | 1.54E-04 | 3.78422023 | 1.20E-08 | 5.7 | 21.50899983 | 3.39E-10 |
| rs6557140 | 4.17E-05 | 4.098003982 | 2.56E-07 | -5.153333333 | -21.17520426 | 4.67E-10 |
| rs6557142 | 2.32E-06 | 4.723569501 | 1.15E-05 | 4.38671875 | 20.66678024 | 7.62E-10 |
| rs6557210 | 6.04E-05 | 4.011177094 | 1.70E-06 | 4.786324786 | 19.14793843 | 3.28E-09 |
| rs663173 | 5.92E-05 | -4.015784294 | 9.35E-08 | -5.338983051 | 21.38199697 | 3.83E-10 |
| rs664147 | 5.97E-06 | -4.527586702 | 5.07E-05 | -4.052173913 | 18.29842359 | 7.43E-09 |
| rs6717863 | 7.21E-05 | -3.969171554 | 6.25E-06 | -4.517730496 | 17.88448646 | 1.11E-08 |
| rs6730286 | 7.28E-05 | -3.967020002 | 3.17E-06 | -4.659574468 | 18.43578832 | 6.51E-09 |
| rs673740 | 7.84E-06 | -4.469430203 | 2.06E-04 | -3.711864407 | 16.54590041 | 4.02E-08 |
| rs6746394 | 8.04E-05 | -3.943226506 | 3.76E-06 | 4.624113475 | -18.28233818 | 7.55E-09 |
| rs6752799 | 9.63E-05 | -3.899864106 | 2.83E-06 | -4.683098592 | 18.21501319 | 8.05E-09 |
| rs675635 | 8.80E-06 | -4.444755387 | 1.92E-04 | -3.728813559 | 16.52976826 | 4.09E-08 |
| rs676349 | 7.47E-06 | -4.4798899 | 9.48E-05 | 3.903508772 | -17.53357158 | 1.55E-08 |
| rs689233 | 8.09E-06 | -4.462874737 | 2.06E-04 | -3.711864407 | 16.5216438 | 4.12E-08 |
| rs6901631 | 3.59E-07 | -5.089590405 | 9.03E-11 | 6.482352941 | -33.08155643 | 5.27E-15 |
| rs6901934 | 4.59E-06 | -4.582879454 | 9.08E-08 | 5.344262295 | -24.55708055 | 1.82E-11 |
| rs6904364 | 9.11E-05 | 3.913240846 | 1.78E-08 | -5.632478632 | -22.10289716 | 1.92E-10 |
| rs6912830 | 1.15E-04 | 3.856777186 | 1.43E-09 | -6.051724138 | -23.40764326 | 5.49E-11 |
| rs6913515 | 7.63E-05 | 3.955882008 | 5.34E-10 | 6.208695652 | 24.49017469 | 1.94E-11 |
| rs6914656 | 7.66E-05 | 3.954795957 | 5.34E-10 | -6.208695652 | -24.62514181 | 1.71E-11 |
| rs6921042 | 2.04E-04 | 3.713496347 | 1.22E-09 | -6.077586207 | -22.63557254 | 1.15E-10 |
| rs6921228 | 1.06E-04 | 3.875315421 | 1.85E-06 | -4.769230769 | -18.53177464 | 5.93E-09 |
| rs6930557 | 2.47E-05 | 4.217862467 | 7.50E-10 | 6.155172414 | 25.889053 | 5.09E-12 |
| rs6931104 | 2.20E-05 | 4.243550527 | 3.06E-10 | 6.295652174 | 26.6407345 | 2.48E-12 |
| rs6933660 | 6.35E-07 | 4.98027699 | 9.66E-12 | 6.81147541 | 33.83017538 | 2.58E-15 |
| rs6936936 | 7.66E-05 | 3.954744841 | 5.34E-10 | -6.208695652 | -24.62482392 | 1.71E-11 |
| rs6937816 | 2.09E-04 | 3.707825622 | 1.46E-14 | -7.691056911 | -28.61260767 | 3.76E-13 |
| rs6937888 | 1.04E-04 | 3.881559674 | 1.70E-06 | 4.786324786 | 18.5288807 | 5.95E-09 |
| rs6939382 | 4.05E-05 | 4.104398477 | 2.36E-06 | -4.720338983 | -19.42544278 | 2.51E-09 |
| rs6940667 | 9.41E-07 | 4.90361385 | 4.42E-05 | 4.084291188 | 19.97467474 | 1.48E-09 |
| rs6940851 | 5.23E-06 | 4.555412371 | 1.90E-07 | 5.208695652 | 23.66531432 | 4.29E-11 |
| rs7101406 | 4.83E-05 | -4.063944066 | 9.35E-08 | 5.338983051 | -21.75633953 | 2.67E-10 |
| rs7102959 | 2.98E-05 | -4.175061454 | 3.99E-08 | -5.491525424 | 22.86539315 | 9.23E-11 |
| rs7109221 | 1.71E-06 | -4.784664631 | 1.72E-07 | 5.226890756 | -25.07474272 | 1.11E-11 |
| rs7112118 | 2.91E-05 | -4.1802587 | 5.06E-08 | 5.449152542 | -22.84069348 | 9.45E-11 |
| rs7112549 | 2.90E-05 | -4.180836981 | 5.31E-08 | -5.440677966 | 22.68518713 | 1.10E-10 |
| rs7113720 | 4.83E-05 | -4.063869805 | 7.05E-08 | -5.389830508 | 21.84414408 | 2.46E-10 |
| rs7114540 | 4.85E-05 | -4.062753755 | 8.51E-08 | 5.355932203 | -21.81906949 | 2.52E-10 |
| rs7116893 | 4.79E-09 | -5.854157583 | 7.34E-19 | 8.869565217 | -52.07185334 | 7.57E-23 |
| rs7117691 | 6.10E-09 | -5.814055671 | 7.34E-19 | 8.869565217 | -51.7155526 | 1.06E-22 |
| rs7118949 | 6.10E-09 | -5.814079043 | 6.95E-19 | 8.875776398 | -51.7520169 | 1.02E-22 |
| rs7121963 | 6.10E-09 | -5.814079043 | 7.34E-19 | 8.869565217 | -51.71576025 | 1.06E-22 |
| rs7122449 | 6.09E-09 | -5.814133576 | 7.34E-19 | -8.869565217 | 51.42213434 | 1.40E-22 |
| rs7122731 | 1.45E-06 | -4.818639544 | 1.45E-07 | -5.258333333 | 25.27167617 | 9.19E-12 |
| rs7123331 | 1.62E-06 | -4.796122355 | 1.31E-07 | -5.277310924 | 25.2443136 | 9.44E-12 |
| rs7123626 | 5.28E-09 | -5.838228199 | 2.13E-19 | -9.00621118 | 52.43005962 | 5.39E-23 |
| rs7126848 | 7.85E-06 | -4.46928467 | 3.07E-05 | -4.168067227 | 18.57957598 | 5.67E-09 |
| rs7128357 | 1.41E-06 | -4.823398552 | 1.04E-07 | -5.319327731 | 25.58999955 | 6.78E-12 |
| rs7129554 | 4.83E-05 | -4.063824755 | 8.12E-08 | 5.36440678 | -21.85937533 | 2.42E-10 |
| rs71575926 | 1.94E-02 | 2.337098304 | 3.93E-13 | 7.257861635 | 16.88643781 | 2.90E-08 |
| rs72478520 | 6.93E-04 | 3.39241989 | 3.86E-18 | 8.682758621 | 29.3421471 | 1.87E-13 |
| rs725158 | 6.15E-04 | 3.425012659 | 2.85E-20 | 9.224489796 | 31.46761886 | 2.46E-14 |
| rs72665317 | 1.18E-03 | 3.244872785 | 2.14E-20 | -9.255033557 | -30.15737041 | 8.60E-14 |
| rs72884326 | 4.45E-05 | -4.082682542 | 8.16E-06 | 4.460869565 | -18.26024984 | 7.71E-09 |
| rs72885907 | 2.99E-05 | -4.174010907 | 2.65E-08 | -5.56302521 | 23.15704499 | 6.98E-11 |
| rs729847 | 1.89E-05 | 4.278019148 | 6.54E-05 | 3.992424242 | 17.03501529 | 2.51E-08 |
| rs73007778 | 6.46E-04 | -3.411601422 | 1.18E-06 | 4.85915493 | -16.62370198 | 3.73E-08 |
| rs7309252 | 8.16E-04 | 3.347294586 | 4.76E-08 | -5.460176991 | -18.33057544 | 7.20E-09 |
| rs7310833 | 9.47E-04 | 3.305737057 | 8.19E-08 | 5.362831858 | 17.67634264 | 1.35E-08 |
| rs73236104 | 2.03E-04 | -3.715545565 | 2.43E-12 | -7.007462687 | 25.95447241 | 4.78E-12 |
| rs73236106 | 9.68E-05 | -3.898377513 | 2.20E-13 | -7.3359375 | 28.50821667 | 4.16E-13 |
| rs73236109 | 9.35E-04 | -3.309342177 | 8.72E-14 | -7.459016393 | 24.59754382 | 1.75E-11 |
| rs73236111 | 1.51E-03 | -3.171860698 | 3.59E-12 | 6.952380952 | -22.12848594 | 1.87E-10 |
| rs733189 | 1.95E-06 | 4.758447246 | 4.35E-07 | 5.053097345 | 23.98207221 | 3.16E-11 |
| rs733190 | 2.02E-06 | 4.751197455 | 4.55E-07 | -5.044247788 | -24.0291635 | 3.02E-11 |
| rs733191 | 2.01E-06 | 4.752420597 | 4.15E-07 | -5.061946903 | -24.11969666 | 2.77E-11 |
| rs73625113 | 1.35E-05 | -4.351239491 | 1.58E-08 | -5.652996845 | 24.53117474 | 1.87E-11 |
| rs73625120 | 7.26E-06 | -4.485929277 | 2.24E-08 | -5.592356688 | 25.01988691 | 1.17E-11 |
| rs73625127 | 1.53E-05 | -4.32386835 | 1.14E-08 | -5.709265176 | 24.61921777 | 1.72E-11 |
| rs73625130 | 2.88E-05 | -4.182889603 | 1.45E-07 | -5.258785942 | 21.93803748 | 2.25E-10 |
| rs73625131 | 2.88E-05 | -4.18304002 | 1.19E-07 | -5.294871795 | 22.08927851 | 1.94E-10 |
| rs73625140 | 7.31E-06 | -4.484466142 | 1.45E-07 | -5.258899676 | 23.52106571 | 4.92E-11 |
| rs73625169 | 8.93E-06 | -4.441634877 | 1.01E-07 | 5.324840764 | -23.71402589 | 4.09E-11 |
| rs73632499 | 8.78E-05 | -3.922139896 | 4.28E-06 | -4.597173145 | 17.98313447 | 1.01E-08 |
| rs73633307 | 6.14E-04 | -3.425370419 | 3.05E-07 | 5.120521173 | -17.58942387 | 1.47E-08 |
| rs73781102 | 1.64E-06 | -4.793093818 | 3.39E-13 | 7.277777778 | -34.982599 | 8.60E-16 |
| rs73783017 | 1.01E-06 | 4.889676845 | 4.06E-05 | -4.103846154 | -20.11989866 | 1.29E-09 |
| rs73783019 | 9.38E-07 | 4.904173834 | 5.00E-05 | -4.055762082 | -19.94325088 | 1.53E-09 |
| rs73783022 | 8.47E-07 | 4.924289301 | 5.04E-05 | -4.053846154 | -20.01563851 | 1.42E-09 |
| rs73783025 | 9.53E-07 | 4.901129817 | 4.13E-05 | 4.1 | 20.04138436 | 1.39E-09 |
| rs73783029 | 2.20E-06 | 4.733876509 | 1.24E-05 | 4.37109375 | 20.63807864 | 7.83E-10 |
| rs739730 | 1.34E-07 | -5.273630536 | 3.12E-04 | 3.605042017 | -19.06514414 | 3.55E-09 |
| rs7412010 | 2.37E-05 | 4.226392252 | 5.08E-23 | 9.88 | 41.60605884 | 1.57E-18 |
| rs7425711 | 7.62E-05 | -3.956092971 | 4.77E-06 | 4.574468085 | -18.14496727 | 8.61E-09 |
| rs74331022 | 6.88E-09 | -5.793695784 | 1.35E-18 | -8.801242236 | 50.84690221 | 2.42E-22 |
| rs74485684 | 3.28E-13 | -7.282328638 | 3.77E-22 | 9.67721519 | -70.66492356 | 1.71E-30 |
| rs7453575 | 1.87E-06 | 4.76679478 | 4.73E-06 | -4.576335878 | -21.87169394 | 2.39E-10 |
| rs74636305 | 8.96E-06 | -4.440948104 | 5.76E-14 | -7.513513514 | 33.26775555 | 4.41E-15 |
| rs74798609 | 3.90E-04 | 3.547023069 | 2.40E-07 | -5.165562914 | -18.37383304 | 6.91E-09 |
| rs75034466 | 3.30E-04 | 3.590288941 | 2.24E-06 | 4.730215827 | 16.93684971 | 2.76E-08 |
| rs75107694 | 1.28E-07 | 5.281013461 | 1.50E-05 | -4.32885906 | -22.92188404 | 8.74E-11 |
| rs7515106 | 2.25E-03 | 3.055214113 | 9.59E-15 | -7.744525547 | -23.75196873 | 3.94E-11 |
| rs7521902 | 1.67E-05 | 4.304731816 | 3.96E-15 | 7.856060606 | 33.71354312 | 2.89E-15 |
| rs75318086 | 5.25E-03 | 2.791243226 | 2.48E-10 | 6.328313253 | 17.60143489 | 1.45E-08 |
| rs75525300 | 4.28E-13 | -7.246468009 | 1.75E-21 | 9.518987342 | -69.16663681 | 7.06E-30 |
| rs7569813 | 8.52E-05 | -3.929276375 | 2.70E-06 | -4.692307692 | 18.38852607 | 6.81E-09 |
| rs75730028 | 5.34E-06 | -4.550811793 | 1.24E-13 | 7.412556054 | -33.83229575 | 2.58E-15 |
| rs7595894 | 8.03E-05 | -3.943379791 | 2.58E-06 | 4.701388889 | -18.58871917 | 5.62E-09 |
| rs7598280 | 9.39E-05 | -3.905767853 | 3.76E-06 | 4.624113475 | -18.10873952 | 8.92E-09 |
| rs76010945 | 1.31E-05 | 4.358245245 | 1.48E-06 | 4.814102564 | 20.92604732 | 5.94E-10 |
| rs760383 | 1.98E-05 | 4.267077449 | 6.33E-05 | -4 | -17.11315228 | 2.33E-08 |
| rs7673527 | 1.43E-07 | -5.261760016 | 8.50E-04 | -3.336134454 | 17.50330837 | 1.60E-08 |
| rs76959488 | 2.50E-07 | -5.157812114 | 1.18E-17 | -8.55483871 | 43.99408251 | 1.62E-19 |
| rs76969132 | 4.29E-06 | 4.596709862 | 2.24E-06 | -4.73046875 | -21.80162512 | 2.56E-10 |
| rs77055031 | 1.40E-04 | -3.808062515 | 1.92E-09 | 6.004366812 | -22.93125792 | 8.66E-11 |
| rs7739424 | 1.01E-03 | 3.288080578 | 1.88E-07 | 5.210526316 | 17.08311468 | 2.40E-08 |
| rs7740302 | 1.38E-06 | 4.827219762 | 1.90E-17 | -8.5 | -41.15657973 | 2.41E-18 |
| rs7740449 | 7.63E-05 | 3.955803715 | 2.28E-06 | -4.726495726 | -18.74688546 | 4.82E-09 |
| rs7742927 | 6.44E-03 | 2.724606032 | 3.14E-16 | 8.167883212 | 22.1574833 | 1.82E-10 |
| rs7743748 | 8.79E-05 | -3.921831935 | 5.07E-06 | -4.561837456 | 17.84356344 | 1.15E-08 |
| rs77483076 | 4.25E-04 | 3.524284837 | 1.50E-06 | 4.811634349 | 16.91117617 | 2.83E-08 |
| rs7748711 | 2.30E-06 | 4.725137803 | 2.62E-05 | 4.203921569 | 19.81194601 | 1.73E-09 |
| rs7748785 | 9.38E-07 | 4.904297532 | 3.87E-05 | 4.115384615 | 20.12961746 | 1.28E-09 |
| rs7749424 | 8.62E-04 | 3.332194478 | 2.72E-07 | 5.141791045 | 17.08448055 | 2.39E-08 |
| rs7752091 | 2.69E-04 | 3.643718137 | 1.29E-08 | -5.688 | -20.78527025 | 6.80E-10 |
| rs7753324 | 1.87E-06 | 4.767174914 | 4.56E-06 | 4.583969466 | 21.7955467 | 2.58E-10 |
| rs77577224 | 3.59E-04 | 3.568739221 | 1.80E-06 | 4.774193548 | 16.99151549 | 2.62E-08 |
| rs7759516 | 1.04E-06 | 4.884381454 | 2.07E-17 | -8.489655172 | -41.59242502 | 1.59E-18 |
| rs77600257 | 1.90E-06 | 4.763685237 | 7.65E-06 | 4.474708171 | 21.26039761 | 4.31E-10 |
| rs7762223 | 2.74E-06 | 4.689417474 | 3.04E-06 | -4.66798419 | -21.94751816 | 2.23E-10 |
| rs7762963 | 8.04E-06 | 4.464178533 | 5.53E-06 | -4.543726236 | -20.33719344 | 1.05E-09 |
| rs7763242 | 5.13E-06 | 4.55927025 | 2.64E-07 | 5.147826087 | 23.40866453 | 5.48E-11 |
| rs7763324 | 1.84E-06 | 4.770632803 | 5.65E-10 | -6.2 | -29.65814124 | 1.38E-13 |
| rs7764129 | 8.18E-04 | 3.346616882 | 2.21E-07 | -5.180451128 | -17.38683578 | 1.79E-08 |
| rs7765741 | 8.61E-04 | 3.332243708 | 2.62E-07 | -5.149253731 | -17.20787085 | 2.12E-08 |
| rs7769780 | 8.30E-04 | 3.342578194 | 1.54E-07 | -5.248120301 | -17.59299181 | 1.47E-08 |
| rs7770865 | 8.40E-04 | 3.33915595 | 2.21E-07 | 5.180451128 | 17.24878568 | 2.04E-08 |
| rs7771156 | 1.61E-04 | 3.774102969 | 7.66E-08 | 5.375 | 20.22954525 | 1.16E-09 |
| rs7771204 | 8.63E-04 | 3.331616829 | 2.04E-07 | 5.195488722 | 17.25969096 | 2.02E-08 |
| rs7772990 | 1.00E-03 | 3.289622001 | 1.67E-07 | 5.233082707 | 17.16502762 | 2.21E-08 |
| rs7774446 | 9.72E-04 | 3.298556766 | 1.48E-07 | -5.255639098 | -17.38648284 | 1.79E-08 |
| rs7775640 | 1.11E-03 | 3.261801194 | 3.54E-07 | 5.092198582 | 16.56203912 | 3.96E-08 |
| rs7775980 | 1.07E-06 | 4.877872168 | 2.48E-17 | -8.468965517 | -41.43569904 | 1.85E-18 |
| rs7776230 | 5.97E-06 | -4.527414371 | 5.64E-08 | 5.429752066 | -24.64825244 | 1.67E-11 |
| rs7776399 | 1.29E-05 | -4.360962744 | 1.03E-05 | 4.41025641 | -19.28339153 | 2.88E-09 |
| rs77942641 | 5.03E-03 | 2.804954092 | 8.79E-10 | -6.129909366 | -17.25364789 | 2.03E-08 |
| rs77977611 | 3.04E-05 | -4.170788606 | 1.71E-05 | 4.3 | -17.98143273 | 1.01E-08 |
| rs77988055 | 3.31E-04 | 3.589868502 | 3.03E-06 | -4.669064748 | -16.80679562 | 3.13E-08 |
| rs78026352 | 4.45E-05 | -4.082721359 | 7.53E-06 | 4.47826087 | -18.33163101 | 7.20E-09 |
| rs78114378 | 3.40E-04 | 3.582391743 | 1.84E-06 | 4.770609319 | 17.04377259 | 2.49E-08 |
| rs78181855 | 4.67E-03 | 2.829049788 | 1.13E-09 | -6.090361446 | -17.28901515 | 1.96E-08 |
| rs78375092 | 8.19E-07 | 4.930867975 | 1.78E-05 | -4.2909699 | -21.21421343 | 4.50E-10 |
| rs78436453 | 5.20E-03 | 2.794562628 | 8.92E-10 | 6.127659574 | 17.06494171 | 2.44E-08 |
| rs78516619 | 1.03E-09 | -6.105052213 | 5.62E-03 | 2.769369369 | -16.96601919 | 2.68E-08 |
| rs78588171 | 1.11E-05 | 4.395301507 | 6.98E-06 | 4.494423792 | 19.70281357 | 1.92E-09 |
| rs78900103 | 1.09E-06 | -4.875074421 | 1.03E-13 | 7.437209302 | -36.36058922 | 2.31E-16 |
| rs78959534 | 5.20E-03 | 2.794597656 | 1.01E-09 | 6.108433735 | 17.01173481 | 2.57E-08 |
| rs79028439 | 6.82E-06 | 4.499294355 | 1.99E-04 | -3.720338983 | -16.78357911 | 3.20E-08 |
| rs79050195 | 1.61E-03 | 3.154832524 | 3.22E-19 | 8.961038961 | 28.15276738 | 5.84E-13 |
| rs7929660 | 3.69E-13 | -7.266428322 | 9.16E-22 | 9.585987261 | -69.84554587 | 3.71E-30 |
| rs7930471 | 2.98E-05 | -4.175083985 | 3.99E-08 | -5.491525424 | 22.86551664 | 9.23E-11 |
| rs7935077 | 4.44E-05 | -4.083543171 | 7.84E-06 | -4.469565217 | 18.20386559 | 8.14E-09 |
| rs79364962 | 7.83E-04 | 3.358584532 | 5.11E-07 | 5.022321429 | 16.82027887 | 3.09E-08 |
| rs7940387 | 6.82E-06 | 4.499114428 | 2.35E-04 | 3.677966102 | 16.50355103 | 4.19E-08 |
| rs7940816 | 1.56E-06 | -4.802786802 | 1.65E-07 | 5.235294118 | -25.21016793 | 9.75E-12 |
| rs7943756 | 1.40E-06 | -4.824626256 | 1.57E-07 | 5.243697479 | -25.36543806 | 8.40E-12 |
| rs7943867 | 3.25E-05 | -4.155496522 | 8.65E-08 | 5.352941176 | -22.30432223 | 1.58E-10 |
| rs7945546 | 2.99E-05 | -4.17457748 | 3.99E-08 | 5.491525424 | -22.98716958 | 8.21E-11 |
| rs7947350 | 3.69E-13 | -7.266428322 | 9.16E-22 | 9.585987261 | -69.84554587 | 3.71E-30 |
| rs7948109 | 4.62E-05 | -4.074108277 | 7.39E-08 | -5.381355932 | 21.86481191 | 2.41E-10 |
| rs7949790 | 2.52E-07 | -5.156383487 | 1.32E-17 | 8.541935484 | -44.17595849 | 1.36E-19 |
| rs7951733 | 1.54E-04 | -3.784604595 | 4.05E-10 | 6.252212389 | -23.7321498 | 4.02E-11 |
| rs79540726 | 2.29E-06 | 4.72568081 | 1.21E-05 | 4.375 | 20.62077074 | 7.96E-10 |
| rs79554419 | 9.41E-06 | 4.430319234 | 3.41E-06 | 4.644268775 | 20.52187029 | 8.75E-10 |
| rs79626929 | 8.38E-04 | 3.339794076 | 3.32E-07 | -5.104477612 | -17.09667141 | 2.37E-08 |
| rs7966079 | 8.31E-04 | 3.342204651 | 6.54E-08 | -5.403508772 | -18.11253579 | 8.89E-09 |
| rs7967229 | 9.09E-04 | 3.31725198 | 5.52E-08 | -5.433628319 | -18.07782721 | 9.19E-09 |
| rs79699315 | 5.25E-03 | 2.791264685 | 3.19E-10 | 6.289156627 | 17.49291924 | 1.61E-08 |
| rs79984746 | 3.29E-04 | 3.591594091 | 1.95E-06 | 4.758122744 | 17.04289594 | 2.49E-08 |
| rs80173514 | 1.60E-03 | 3.156465405 | 1.77E-19 | 9.026490066 | 28.37244258 | 4.73E-13 |
| rs80268871 | 3.50E-06 | -4.638999603 | 4.57E-12 | -6.918367347 | 32.00380536 | 1.47E-14 |
| rs80347787 | 2.95E-06 | 4.67453565 | 5.36E-06 | 4.550185874 | 21.21451163 | 4.50E-10 |
| rs8180642 | 2.80E-05 | 4.189148052 | 2.21E-08 | -5.594827586 | -23.50159251 | 5.01E-11 |
| rs892949 | 2.95E-05 | -4.177589881 | 5.31E-08 | -5.440677966 | 22.66755606 | 1.12E-10 |
| rs892950 | 4.35E-05 | -4.088064497 | 9.60E-06 | 4.426086957 | -18.14171703 | 8.64E-09 |
| rs892951 | 4.35E-05 | -4.088041719 | 9.22E-06 | -4.434782609 | 18.08213543 | 9.15E-09 |
| rs892952 | 4.36E-05 | -4.087734223 | 7.23E-06 | -4.486956522 | 18.29344098 | 7.47E-09 |
| rs9284955 | 2.98E-04 | -3.616798662 | 8.58E-13 | -7.151515152 | 25.78179468 | 5.64E-12 |
| rs930320 | 4.64E-05 | -4.072977493 | 8.92E-08 | 5.347457627 | -21.83930068 | 2.47E-10 |
| rs930321 | 4.97E-05 | -4.05684091 | 7.75E-08 | 5.372881356 | -21.85633595 | 2.43E-10 |
| rs9309402 | 9.70E-05 | -3.89797023 | 2.22E-06 | 4.732394366 | -18.49600583 | 6.14E-09 |
| rs9312655 | 1.98E-04 | -3.722156955 | 8.87E-14 | 7.456692913 | -27.84598357 | 7.83E-13 |
| rs9312656 | 1.80E-04 | -3.746127485 | 1.00E-13 | 7.440944882 | -27.96565845 | 6.98E-13 |
| rs9312657 | 1.93E-04 | -3.72792073 | 1.00E-13 | -7.440944882 | 27.64888056 | 9.45E-13 |
| rs931423 | 4.66E-06 | -4.579346014 | 8.68E-08 | 5.352459016 | -24.57580498 | 1.79E-11 |
| rs9322320 | 7.90E-05 | 3.947453254 | 4.05E-10 | -6.252173913 | -24.75181496 | 1.51E-11 |
| rs9322321 | 2.72E-04 | 3.640394355 | 3.91E-09 | -5.888 | -21.49744369 | 3.43E-10 |
| rs9322364 | 8.78E-05 | 3.922179743 | 2.10E-06 | 4.743589744 | 18.55580524 | 5.80E-09 |
| rs9322367 | 5.69E-06 | -4.537637945 | 9.49E-06 | 4.428571429 | -20.14795449 | 1.25E-09 |
| rs9341077 | 8.71E-06 | -4.446892599 | 1.18E-07 | 5.296875 | -23.61733374 | 4.49E-11 |
| rs9371219 | 6.08E-07 | 4.988717984 | 1.52E-11 | -6.745901639 | -33.74566945 | 2.80E-15 |
| rs9371245 | 2.52E-05 | 4.213036363 | 6.26E-07 | 4.982905983 | 20.93763696 | 5.87E-10 |
| rs9371246 | 1.24E-04 | 3.83794542 | 1.07E-08 | 5.719298246 | 21.88847809 | 2.36E-10 |
| rs9371513 | 9.82E-04 | 3.295593154 | 1.88E-07 | -5.210526316 | -17.22158978 | 2.10E-08 |
| rs9371516 | 1.25E-04 | 3.835366968 | 1.87E-09 | 6.00862069 | 22.97898582 | 8.27E-11 |
| rs9371517 | 1.25E-04 | 3.835400404 | 1.77E-09 | 6.017241379 | 23.01211487 | 8.02E-11 |
| rs9371518 | 1.01E-04 | 3.888809575 | 2.34E-05 | -4.23015873 | -16.49356301 | 4.23E-08 |
| rs9371519 | 1.39E-05 | 4.344759236 | 1.11E-06 | 4.871794872 | 21.11120759 | 4.97E-10 |
| rs9371529 | 1.11E-06 | 4.871095481 | 8.33E-17 | -8.326530612 | -40.68127417 | 3.78E-18 |
| rs9383568 | 6.01E-07 | 4.990800462 | 1.44E-05 | 4.337792642 | 21.59203716 | 3.13E-10 |
| rs9383615 | 5.09E-06 | 4.561209648 | 3.48E-07 | -5.095652174 | -23.30364743 | 6.06E-11 |
| rs9383887 | 8.45E-04 | 3.337451274 | 2.21E-07 | 5.180451128 | 17.23996951 | 2.06E-08 |
| rs9383888 | 8.44E-04 | 3.338009036 | 1.74E-07 | -5.22556391 | -17.4933694 | 1.61E-08 |
| rs9383889 | 1.33E-05 | 4.354572288 | 1.24E-04 | 3.837837838 | 16.6682063 | 3.58E-08 |
| rs9383890 | 4.60E-07 | 5.042127102 | 1.63E-05 | -4.311036789 | -21.7944832 | 2.58E-10 |
| rs9383893 | 2.46E-05 | 4.218551688 | 7.50E-10 | -6.155172414 | -26.03889335 | 4.41E-12 |
| rs9383900 | 2.15E-05 | 4.248546635 | 3.24E-10 | -6.286956522 | -26.78588932 | 2.16E-12 |
| rs9383906 | 7.70E-08 | 5.374034576 | 1.17E-14 | -7.719047619 | -41.59837617 | 1.58E-18 |
| rs9383911 | 1.90E-08 | 5.621148505 | 1.72E-14 | -7.669902913 | -43.23218547 | 3.34E-19 |
| rs9383915 | 3.92E-03 | 2.884838185 | 1.02E-09 | 6.105740181 | 17.55456927 | 1.52E-08 |
| rs9383980 | 5.23E-06 | 4.55529522 | 2.19E-07 | 5.182608696 | 23.54622534 | 4.80E-11 |
| rs9383982 | 1.06E-04 | 3.87636838 | 2.01E-06 | -4.752136752 | -18.47033119 | 6.30E-09 |
| rs939433 | 7.45E-06 | -4.480314456 | 8.20E-05 | -3.938596491 | 17.59974424 | 1.46E-08 |
| rs9397047 | 9.58E-04 | 3.302694786 | 3.19E-07 | 5.111940299 | 16.83486578 | 3.04E-08 |
| rs9397050 | 4.33E-05 | 4.089031299 | 5.54E-07 | 5.006666667 | 20.4179217 | 9.67E-10 |
| rs9397091 | 5.16E-06 | 4.558241007 | 3.81E-07 | -5.07826087 | -23.20897913 | 6.64E-11 |
| rs9397092 | 7.79E-05 | 3.950705689 | 2.59E-06 | -4.700854701 | -18.62111991 | 5.45E-09 |
| rs9397400 | 1.14E-03 | 3.252453036 | 2.51E-07 | 5.156716418 | 16.72349386 | 3.39E-08 |
| rs9397401 | 1.01E-03 | 3.289093699 | 2.32E-07 | 5.171641791 | 16.96101702 | 2.70E-08 |
| rs9397403 | 1.60E-04 | 3.775716807 | 8.02E-08 | 5.366666667 | 20.20685627 | 1.18E-09 |
| rs9397405 | 1.26E-04 | 3.834818418 | 1.97E-09 | 6 | 22.94277162 | 8.57E-11 |
| rs9397409 | 2.94E-04 | 3.620837109 | 6.32E-09 | 5.808 | 20.96871961 | 5.70E-10 |
| rs9397412 | 6.08E-06 | 4.523529929 | 7.66E-12 | 6.844827586 | 30.87498332 | 4.33E-14 |
| rs9397413 | 6.03E-07 | 4.990249445 | 1.61E-11 | -6.737704918 | -33.71497212 | 2.88E-15 |
| rs9397414 | 6.06E-07 | 4.989382106 | 9.66E-12 | -6.81147541 | -34.07849543 | 2.04E-15 |
| rs9397422 | 3.56E-07 | 5.091104413 | 8.03E-11 | 6.5 | 33.00327577 | 5.68E-15 |
| rs9397495 | 5.31E-06 | 4.552301459 | 2.29E-07 | -5.173913043 | -23.61546827 | 4.49E-11 |
| rs9478208 | 5.76E-05 | 4.022455158 | 6.79E-12 | -6.862068966 | -27.68527447 | 9.13E-13 |
| rs9479029 | 1.00E-03 | 3.289425281 | 2.06E-07 | -5.194029851 | -17.13490992 | 2.28E-08 |
| rs9479041 | 2.46E-05 | 4.218266702 | 9.84E-10 | -6.112068966 | -25.85462164 | 5.26E-12 |
| rs9479289 | 6.21E-03 | 2.736489045 | 3.54E-16 | 8.153284672 | 22.21482067 | 1.72E-10 |
| rs954238 | 6.50E-07 | 4.975616618 | 9.89E-17 | 8.306122449 | 41.20579047 | 2.30E-18 |
| rs963193 | 2.09E-05 | 4.255346997 | 4.78E-10 | -6.226086957 | -26.56870006 | 2.66E-12 |
| rs9637989 | 4.33E-05 | 4.089015136 | 5.35E-07 | -5.013333333 | -20.55445821 | 8.48E-10 |
| rs9689096 | 7.86E-04 | 3.357540835 | 3.71E-07 | -5.083333333 | -17.11614045 | 2.32E-08 |
| rs972409 | 1.24E-07 | -5.287882921 | 3.02E-04 | -3.613445378 | 19.05397615 | 3.59E-09 |
| rs979210 | 4.38E-05 | -4.086488 | 8.50E-06 | 4.452173913 | -18.24163 | 7.85E-09 |
| rs980227 | 5.85E-05 | -4.018834966 | 2.24E-06 | 4.730496454 | -19.06158948 | 3.56E-09 |
| rs9992737 | 9.75E-05 | -3.896740827 | 2.20E-13 | -7.3359375 | 28.49622664 | 4.20E-13 |
| rs9997685 | 1.47E-03 | -3.17972484 | 5.62E-12 | 6.888888889 | -21.98018845 | 2.16E-10 |
| rs9998950 | 1.51E-04 | -3.789064909 | 1.20E-12 | -7.105263158 | 26.83770888 | 2.05E-12 |

SNP: single nucleotide polymorphisms; RA: rheumatoid arthritis.

**Supplemental Table S6. Candidate Pleiotropic Genes Identified by MAGMA.**

| **ensg** | **symbol** | **chr** | **start** | **end** | **pLI** | **posMapSNPs** | **posMapMaxCADD** | **eqtlMapSNPs** | **eqtlMapminP** | **eqtlMapminQ** | **eqtlMapts** | **minGwasP** | **IndSigSNPs** |
| --- | --- | --- | --- | --- | --- | --- | --- | --- | --- | --- | --- | --- | --- |
| ENSG00000187608 | ISG15 | 1 | 948803 | 949920 | 0.009847813 | 0 | 0 | 1 | 3.56E-10 | 2.15E-05 | eQTLGen_trans_eQTLs | 4.00E-08 | rs1990760 |
| ENSG00000162571 | TTLL10 | 1 | 1109264 | 1133315 | 1.95E-05 | 0 | 0 | 1 | 1.54E-05 | 0.010069577 | BIOSQTL/BIOS_eQTL_geneLevel | 1.13E-07 | rs2649599 |
| ENSG00000186891 | TNFRSF18 | 1 | 1138888 | 1142071 | 8.37E-07 | 0 | 0 | 1 | 3.74E-49 | 0 | eQTLcatalogue/Fairfax_2014_LPS2:eQTLcatalogue/Lepik_2017_ge_blood:eQTLGen_cis_eQTLs:BIOSQTL/BIOS_eQTL_geneLevel | 1.13E-07 | rs2649599 |
| ENSG00000186827 | TNFRSF4 | 1 | 1146706 | 1149518 | 0.00720933 | 0 | 0 | 1 | 8.37E-30 | 0 | eQTLGen_cis_eQTLs:BIOSQTL/BIOS_eQTL_geneLevel | 1.13E-07 | rs2649599 |
| ENSG00000078808 | SDF4 | 1 | 1152288 | 1167411 | 0.001309827 | 0 | 0 | 1 | 2.69E-19 | 0 | eQTLGen_cis_eQTLs | 1.13E-07 | rs2649599 |
| ENSG00000176022 | B3GALT6 | 1 | 1167629 | 1170421 | 0.048104466 | 0 | 0 | 1 | 3.66E-131 | 0 | eQTLcatalogue/Fairfax_2012_B-cell_CD19:eQTLcatalogue/Fairfax_2014_IFN24:eQTLcatalogue/Fairfax_2014_naive:eQTLcatalogue/Lepik_2017_ge_blood:eQTLGen_cis_eQTLs:BIOSQTL/BIOS_eQTL_geneLevel | 1.13E-07 | rs2649599 |
| ENSG00000162572 | SCNN1D | 1 | 1215816 | 1227409 | 2.82E-15 | 0 | 0 | 1 | 5.30E-23 | 0 | eQTLGen_cis_eQTLs:BIOSQTL/BIOS_eQTL_geneLevel | 1.13E-07 | rs2649599 |
| ENSG00000131584 | ACAP3 | 1 | 1227756 | 1244989 | 0.03943093 | 1 | 16.23 | 1 | 1.14E-16 | 1.07E-13 | GTEx/v8/Thyroid | 1.13E-07 | rs2649599 |
| ENSG00000169972 | PUSL1 | 1 | 1243947 | 1247057 | 6.59E-12 | 1 | 16.23 | 1 | 3.71E-09 | 5.46E-06 | eQTLGen_cis_eQTLs:BIOSQTL/BIOS_eQTL_geneLevel:GTEx/v8/Thyroid | 1.13E-07 | rs2649599 |
| ENSG00000127054 | CPSF3L | 1 | 1246965 | 1260071 | 5.79E-09 | 1 | 16.23 | 1 | 1.95E-23 | 0 | eQTLcatalogue/Lepik_2017_ge_blood:eQTLGen_cis_eQTLs:BIOSQTL/BIOS_eQTL_geneLevel:GTEx/v8/Whole_Blood:GTEx/v8/Thyroid | 1.13E-07 | rs2649599 |
| ENSG00000224051 | GLTPD1 | 1 | 1260136 | 1264277 | 0.00138279 | 0 | 0 | 1 | 5.43E-28 | 0 | eQTLcatalogue/Lepik_2017_ge_blood:eQTLGen_cis_eQTLs:BIOSQTL/BIOS_eQTL_geneLevel | 1.13E-07 | rs2649599 |
| ENSG00000169962 | TAS1R3 | 1 | 1266694 | 1270686 | NA | 0 | 0 | 1 | 1.49E-18 | 0 | eQTLGen_cis_eQTLs:BIOSQTL/BIOS_eQTL_geneLevel:GTEx/v8/Whole_Blood | 1.13E-07 | rs2649599 |
| ENSG00000107404 | DVL1 | 1 | 1270656 | 1284730 | 0.157164972 | 0 | 0 | 1 | 1.34E-05 | 0.008957944 | BIOSQTL/BIOS_eQTL_geneLevel | 1.13E-07 | rs2649599 |
| ENSG00000224870 | RP4-758J18.2 | 1 | 1334902 | 1337426 | NA | 0 | 0 | 1 | 6.09E-07 | 0.000514999 | BIOSQTL/BIOS_eQTL_geneLevel | 1.13E-07 | rs2649599 |
| ENSG00000242485 | MRPL20 | 1 | 1337288 | 1342693 | 0.000171803 | 0 | 0 | 1 | 1.17E-79 | 0 | eQTLcatalogue/Fairfax_2012_B-cell_CD19:eQTLcatalogue/Fairfax_2014_IFN24:eQTLcatalogue/Fairfax_2014_LPS24:eQTLcatalogue/Fairfax_2014_LPS2:eQTLcatalogue/Fairfax_2014_naive:eQTLGen_cis_eQTLs:BIOSQTL/BIOS_eQTL_geneLevel:GTEx/v8/Whole_Blood:GTEx/v8/Thyroid | 1.13E-07 | rs2649599 |
| ENSG00000235098 | ANKRD65 | 1 | 1353800 | 1357149 | NA | 0 | 0 | 1 | 7.51E-13 | 0 | eQTLGen_cis_eQTLs:BIOSQTL/BIOS_eQTL_geneLevel:GTEx/v8/Spleen:GTEx/v8/Thyroid | 1.13E-07 | rs2649599 |
| ENSG00000197785 | ATAD3A | 1 | 1447531 | 1470067 | 0.040130598 | 0 | 0 | 1 | 1.06E-07 | 0.000351604 | eQTLGen_cis_eQTLs | 1.13E-07 | rs2649599 |
| ENSG00000160075 | SSU72 | 1 | 1477053 | 1510249 | 0.606848849 | 0 | 0 | 1 | 8.37E-44 | 0 | eQTLGen_cis_eQTLs:BIOSQTL/BIOS_eQTL_geneLevel | 1.13E-07 | rs2649599 |
| ENSG00000215014 | AL645728.1 | 1 | 1510355 | 1511373 | NA | 0 | 0 | 1 | 1.68E-06 | 0.004961344 | eQTLGen_cis_eQTLs | 1.13E-07 | rs2649599 |
| ENSG00000149527 | PLCH2 | 1 | 2357419 | 2436969 | 1.71E-08 | 0 | 0 | 1 | 1.05E-42 | 0 | eQTLGen_cis_eQTLs:BIOSQTL/BIOS_eQTL_geneLevel | 1.09E-09 | rs60733400 |
| ENSG00000157881 | PANK4 | 1 | 2439972 | 2458039 | 0.945333402 | 0 | 0 | 1 | 6.70E-06 | 0.019121694 | eQTLGen_cis_eQTLs | 1.09E-09 | rs60733400 |
| ENSG00000157873 | TNFRSF14 | 1 | 2487078 | 2496821 | 0.819999054 | 0 | 0 | 1 | 6.66E-109 | 0 | eQTLcatalogue/GEUVADIS_ge_LCL:eQTLcatalogue/TwinsUK_ge_LCL:eQTLGen_cis_eQTLs:BIOSQTL/BIOS_eQTL_geneLevel:GTEx/v8/Thyroid | 1.09E-09 | rs60733400 |
| ENSG00000157870 | FAM213B | 1 | 2517930 | 2522908 | 0.0001667 | 0 | 0 | 1 | 5.72E-261 | 0 | eQTLcatalogue/BLUEPRINT_ge_monocyte:eQTLcatalogue/BLUEPRINT_ge_T-cell:eQTLcatalogue/CEDAR_monocyte_CD14:eQTLcatalogue/Fairfax_2014_IFN24:eQTLcatalogue/Fairfax_2014_naive:eQTLcatalogue/Lepik_2017_ge_blood:eQTLcatalogue/TwinsUK_ge_blood:eQTLGen_cis_eQTLs:BIOSQTL/BIOS_eQTL_geneLevel:GTEx/v8/Whole_Blood:GTEx/v8/Spleen:GTEx/v8/Thyroid | 1.09E-09 | rs60733400 |
| ENSG00000142606 | MMEL1 | 1 | 2522078 | 2564481 | 8.88E-13 | 1 | 13.86 | 1 | 3.37E-225 | 0 | eQTLcatalogue/Fairfax_2014_naive:eQTLcatalogue/GENCORD_ge_T-cell:eQTLcatalogue/HipSci_ge_iPSC:eQTLcatalogue/Lepik_2017_ge_blood:DICE/T_CD8_naive:eQTLGen_cis_eQTLs:BIOSQTL/BIOS_eQTL_geneLevel:GTEx/v8/Whole_Blood:GTEx/v8/Spleen:GTEx/v8/Thyroid | 1.09E-09 | rs60733400 |
| ENSG00000215912 | TTC34 | 1 | 2567415 | 2718286 | NA | 0 | 0 | 1 | 3.52E-70 | 0 | eQTLcatalogue/BLUEPRINT_ge_T-cell:eQTLcatalogue/GENCORD_ge_T-cell:eQTLcatalogue/TwinsUK_ge_fat:eQTLcatalogue/TwinsUK_ge_LCL:DICE/T_CD8_naive_activated:eQTLGen_cis_eQTLs:BIOSQTL/BIOS_eQTL_geneLevel:GTEx/v8/Cells_EBV-transformed_lymphocytes:GTEx/v8/Whole_Blood:GTEx/v8/Spleen:GTEx/v8/Thyroid | 1.09E-09 | rs60733400 |
| ENSG00000120949 | TNFRSF8 | 1 | 12123434 | 12204264 | 0.845965387 | 0 | 0 | 1 | 2.09E-06 | 0.016064639 | eQTLGen_trans_eQTLs | 4.84E-12 | rs1559810;rs7640550 |
| ENSG00000074964 | ARHGEF10L | 1 | 17866330 | 18024369 | 0.038306294 | 0 | 0 | 1 | 3.35E-08 | 0.000434846 | eQTLGen_trans_eQTLs | 4.84E-12 | rs1559810;rs7640550 |
| ENSG00000126709 | IFI6 | 1 | 27992572 | 27998729 | 0.230931225 | 0 | 0 | 2 | 3.32E-27 | 0 | eQTLGen_trans_eQTLs | 3.62E-08 | rs1990760;rs35667974 |
| ENSG00000126106 | TMEM53 | 1 | 45100910 | 45140227 | 0.038975168 | 0 | 0 | 1 | 1.60E-08 | 0.000203252 | eQTLGen_trans_eQTLs | 4.84E-12 | rs1559810;rs7640550 |
| ENSG00000198520 | C1orf228 | 1 | 45140364 | 45191263 | NA | 0 | 0 | 1 | 2.97E-11 | 0 | eQTLGen_trans_eQTLs | 9.18E-11 | rs6908626 |
| ENSG00000117450 | PRDX1 | 1 | 45976708 | 45988719 | 1.44E-07 | 0 | 0 | 1 | 2.77E-06 | 0.020320329 | eQTLGen_trans_eQTLs | 9.18E-11 | rs6908626 |
| ENSG00000162437 | RAVER2 | 1 | 65210778 | 65298915 | 1.86E-06 | 0 | 0 | 2 | 1.99E-17 | 0 | eQTLGen_cis_eQTLs:BIOSQTL/BIOS_eQTL_geneLevel | 9.87E-08 | rs72922282 |
| ENSG00000162434 | JAK1 | 1 | 65298912 | 65432187 | 0.99590968 | 2 | 15.64 | 2 | 3.29E-17 | 3.95E-19 | GTEx/v8/Thyroid | 9.87E-08 | rs72922282 |
| ENSG00000162433 | AK4 | 1 | 65613232 | 65697828 | 0.236133361 | 0 | 0 | 1 | 6.19E-07 | 4.34E-14 | eQTLGen_cis_eQTLs:GTEx/v8/Thyroid | 6.99E-06 | rs72922282 |
| ENSG00000213625 | LEPROT | 1 | 65886270 | 65901690 | 0.094789357 | 0 | 0 | 1 | 5.63E-07 | 0.001836332 | eQTLGen_cis_eQTLs | 6.99E-06 | rs72922282 |
| ENSG00000116729 | WLS | 1 | 68564142 | 68698803 | 0.344541187 | 0 | 0 | 1 | 1.66E-06 | 0.012909873 | eQTLGen_trans_eQTLs | 1.72E-196 | rs2476601 |
| ENSG00000137959 | IFI44L | 1 | 79085607 | 79111830 | 2.22E-10 | 0 | 0 | 2 | 1.23E-37 | 0 | eQTLGen_trans_eQTLs | 3.62E-08 | rs1990760;rs35667974 |
| ENSG00000137965 | IFI44 | 1 | 79115481 | 79129763 | 4.72E-14 | 0 | 0 | 2 | 6.30E-30 | 0 | eQTLGen_trans_eQTLs | 3.62E-08 | rs1990760;rs35667974 |
| ENSG00000122406 | RPL5 | 1 | 93297582 | 93307481 | 0.994762229 | 0 | 0 | 1 | 1.62E-07 | 0.001618242 | eQTLGen_trans_eQTLs | 2.93E-13 | rs11611029 |
| ENSG00000155366 | RHOC | 1 | 113243728 | 113250056 | 0.586036085 | 0 | 0 | 1 | 2.41E-09 | 7.37E-05 | eQTLGen_trans_eQTLs | 4.84E-12 | rs1559810;rs7640550 |
| ENSG00000081026 | MAGI3 | 1 | 113933371 | 114228545 | 0.570580157 | 4 | 17.38 | 12 | 2.88E-11 | 0 | eQTLcatalogue/TwinsUK_ge_fat:PsychENCODE_eQTLs:eQTLGen_cis_eQTLs:BIOSQTL/BIOS_eQTL_geneLevel | 1.83E-77 | rs2358816;rs11102658;rs7515189;rs11102678;rs3789599;rs1217422;rs1230686;rs11102694;rs12029185;rs7511816 |
| ENSG00000116793 | PHTF1 | 1 | 114239453 | 114302111 | 5.47E-18 | 2 | 21.6 | 22 | 2.58E-63 | 0 | eQTLcatalogue/Alasoo_2018_ge_macrophage_IFNg:eQTLcatalogue/Alasoo_2018_ge_macrophage_naive:eQTLcatalogue/CEDAR_monocyte_CD14:eQTLcatalogue/Fairfax_2014_LPS24:eQTLcatalogue/GENCORD_ge_fibroblast:eQTLcatalogue/Nedelec_2016_ge_macrophage_Listeria:eQTLcatalogue/Nedelec_2016_ge_macrophage_Salmonella:eQTLcatalogue/Quach_2016_ge_monocyte_IAV:eQTLcatalogue/Quach_2016_ge_monocyte_LPS:eQTLcatalogue/Quach_2016_ge_monocyte_naive:eQTLcatalogue/Quach_2016_ge_monocyte_Pam3CSK4:eQTLcatalogue/Quach_2016_ge_monocyte_R848:PsychENCODE_eQTLs:eQTLGen_cis_eQTLs:BIOSQTL/BIOS_eQTL_geneLevel:GTEx/v8/Thyroid | 1.72E-196 | rs3789599;rs1217422;rs2358816;rs11102658;rs11102678;rs2476601;rs7515189;rs1217401;rs11102694;rs3811019;rs12127377;rs7511816 |
| ENSG00000081019 | RSBN1 | 1 | 114304454 | 114355098 | 0.805139688 | 4 | 21.6 | 10 | 3.87E-20 | 0 | eQTLGen_cis_eQTLs:GTEx/v8/Thyroid | 1.83E-77 | rs2358816;rs11102658;rs3789599;rs1217422;rs7515189;rs11102678;rs1230686;rs1217401 |
| ENSG00000134242 | PTPN22 | 1 | 114356433 | 114414381 | 3.08E-16 | 7 | 17.25 | 28 | 1.06E-122 | 0 | eQTLcatalogue/BLUEPRINT_ge_neutrophil:eQTLcatalogue/CEDAR_B-cell_CD19:eQTLcatalogue/CEDAR_T-cell_CD4:eQTLcatalogue/CEDAR_T-cell_CD8:eQTLcatalogue/Fairfax_2012_B-cell_CD19:eQTLcatalogue/Fairfax_2014_IFN24:eQTLcatalogue/Fairfax_2014_naive:eQTLcatalogue/Lepik_2017_ge_blood:PsychENCODE_eQTLs:DICE/B_cell_naive:eQTLGen_cis_eQTLs:BIOSQTL/BIOS_eQTL_geneLevel | 1.72E-196 | rs3789599;rs1217422;rs2358816;rs11102658;rs1230686;rs2476601;rs7511816;rs12127377;rs11102694;rs7515189;rs11102678;rs1217401;rs3811019;rs1000528;rs36064592 |
| ENSG00000188761 | BCL2L15 | 1 | 114420790 | 114430169 | 0.003614688 | 3 | 23.1 | 26 | 2.09E-32 | 0 | eQTLcatalogue/CEDAR_ileum:eQTLcatalogue/CEDAR_transverse_colon:eQTLcatalogue/TwinsUK_ge_LCL:DICE/B_cell_naive:DICE/T_CD4_TH17:DICE/T_CD4_TH2:DICE/T_CD4_naive_TREG:eQTLGen_cis_eQTLs:BIOSQTL/BIOS_eQTL_geneLevel:GTEx/v8/Whole_Blood | 1.72E-196 | rs2358816;rs11102658;rs1217401;rs7511816;rs11102694;rs2476601;rs3811019;rs7515189;rs11102678;rs3789599;rs1217422;rs1230686;rs36064592;rs12127377 |
| ENSG00000134262 | AP4B1 | 1 | 114437370 | 114447823 | 2.69E-08 | 3 | 23.1 | 26 | 1.48E-129 | 0 | eQTLcatalogue/Alasoo_2018_ge_macrophage_Salmonella:eQTLcatalogue/BLUEPRINT_ge_monocyte:eQTLcatalogue/BLUEPRINT_ge_neutrophil:eQTLcatalogue/CEDAR_neutrophil_CD15:eQTLcatalogue/CEDAR_rectum:eQTLcatalogue/CEDAR_transverse_colon:eQTLcatalogue/Fairfax_2012_B-cell_CD19:eQTLcatalogue/Fairfax_2014_IFN24:eQTLcatalogue/Fairfax_2014_LPS24:eQTLcatalogue/Fairfax_2014_LPS2:eQTLcatalogue/TwinsUK_ge_fat:PsychENCODE_eQTLs:eQTLGen_cis_eQTLs:BIOSQTL/BIOS_eQTL_geneLevel:GTEx/v8/Whole_Blood:GTEx/v8/Spleen:GTEx/v8/Thyroid | 1.72E-196 | rs1217401;rs11102694;rs3789599;rs1217422;rs2358816;rs11102658;rs2476601;rs12127377;rs7515189;rs11102678;rs1230686;rs7511816;rs3811019;rs1000528 |
| ENSG00000118655 | DCLRE1B | 1 | 114447763 | 114456708 | 0.107858132 | 2 | 23.1 | 27 | 3.46E-182 | 0 | eQTLcatalogue/Alasoo_2018_ge_macrophage_IFNg:eQTLcatalogue/BLUEPRINT_ge_monocyte:eQTLcatalogue/CEDAR_monocyte_CD14:eQTLcatalogue/CEDAR_T-cell_CD4:eQTLcatalogue/CEDAR_T-cell_CD8:eQTLcatalogue/Fairfax_2012_B-cell_CD19:eQTLcatalogue/Fairfax_2014_IFN24:eQTLcatalogue/Fairfax_2014_LPS24:eQTLcatalogue/Fairfax_2014_LPS2:eQTLcatalogue/Fairfax_2014_naive:eQTLcatalogue/Lepik_2017_ge_blood:eQTLcatalogue/Quach_2016_ge_monocyte_naive:PsychENCODE_eQTLs:eQTLGen_cis_eQTLs:BIOSQTL/BIOS_eQTL_geneLevel:GTEx/v8/Thyroid | 1.72E-196 | rs1217401;rs11102694;rs2476601;rs12127377;rs2358816;rs11102658;rs7511816;rs1000528;rs3811019;rs11102678;rs36064592;rs3789599;rs1217422;rs1230686 |
| ENSG00000163349 | HIPK1 | 1 | 114471814 | 114520426 | 0.999942915 | 6 | 18.16 | 7 | 7.79E-09 | 4.51E-05 | PsychENCODE_eQTLs:eQTLGen_cis_eQTLs:BIOSQTL/BIOS_eQTL_geneLevel | 5.78E-37 | rs3811019;rs36064592;rs12127377;rs1000528 |
| ENSG00000116774 | OLFML3 | 1 | 114522063 | 114524876 | 0.000588287 | 2 | 18.16 | 6 | 6.47E-15 | 6.47E-11 | eQTLcatalogue/TwinsUK_ge_fat:PsychENCODE_eQTLs:GTEx/v8/Thyroid | 4.58E-15 | rs12127377;rs1000528;rs36064592 |
| ENSG00000014914 | MTMR11 | 1 | 149900543 | 149908791 | 7.33E-13 | 0 | 0 | 1 | 1.42E-07 | 0.001542097 | eQTLGen_trans_eQTLs | 4.84E-12 | rs1559810;rs7640550 |
| ENSG00000143554 | SLC27A3 | 1 | 153746830 | 153752633 | 7.80E-11 | 0 | 0 | 1 | 3.14E-10 | 2.17E-05 | eQTLGen_trans_eQTLs | 4.84E-12 | rs1559810;rs7640550 |
| ENSG00000158481 | CD1C | 1 | 158259576 | 158263420 | 6.44E-07 | 0 | 0 | 1 | 3.07E-07 | 0.00282089 | eQTLGen_trans_eQTLs | 9.18E-11 | rs6908626 |
| ENSG00000158488 | CD1E | 1 | 158323254 | 158327343 | 8.78E-13 | 0 | 0 | 1 | 1.88E-09 | 5.63E-05 | eQTLGen_trans_eQTLs | 9.18E-11 | rs6908626 |
| ENSG00000179639 | FCER1A | 1 | 159259504 | 159278014 | 0.002558028 | 0 | 0 | 1 | 3.51E-09 | 0.000107181 | eQTLGen_trans_eQTLs | 9.18E-11 | rs6908626 |
| ENSG00000117090 | SLAMF1 | 1 | 160577890 | 160617085 | 0.135406432 | 0 | 0 | 2 | 4.65E-13 | 0 | eQTLGen_trans_eQTLs | 1.72E-196 | rs2476601;rs6908626 |
| ENSG00000162745 | OLFML2B | 1 | 161952982 | 161993644 | 3.02E-06 | 0 | 0 | 1 | 5.52E-07 | 0.004893707 | eQTLGen_trans_eQTLs | 4.84E-12 | rs1559810;rs7640550 |
| ENSG00000198821 | CD247 | 1 | 167399877 | 167487847 | 0.661382967 | 0 | 0 | 2 | 1.33E-235 | 0 | eQTLcatalogue/CEDAR_T-cell_CD4:DICE/T_CD4_naive_TREG:eQTLGen_cis_eQTLs:eQTLGen_trans_eQTLs:BIOSQTL/BIOS_eQTL_geneLevel | 1.72E-196 | rs1214598;rs2476601 |
| ENSG00000090104 | RGS1 | 1 | 192544857 | 192549161 | 0.635710552 | 0 | 0 | 1 | 8.28E-07 | 0.006998708 | eQTLGen_trans_eQTLs | 1.72E-196 | rs2476601 |
| ENSG00000143847 | PPFIA4 | 1 | 202995626 | 203047868 | 0.001515533 | 0 | 0 | 1 | 1.01E-06 | 0.00822632 | eQTLGen_trans_eQTLs | 4.84E-12 | rs1559810;rs7640550 |
| ENSG00000123685 | BATF3 | 1 | 212859760 | 212873327 | 0.030761382 | 0 | 0 | 1 | 2.57E-13 | 0 | eQTLGen_trans_eQTLs | 4.84E-12 | rs1559810;rs7640550 |
| ENSG00000168264 | IRF2BP2 | 1 | 234740015 | 234745271 | 0.470291319 | 0 | 0 | 1 | 3.18E-07 | 0.002904424 | eQTLGen_trans_eQTLs | 4.84E-12 | rs1559810;rs7640550 |
| ENSG00000077585 | GPR137B | 1 | 236305832 | 236385165 | 0.00025303 | 0 | 0 | 1 | 1.60E-07 | 0.001620228 | eQTLGen_trans_eQTLs | 4.84E-12 | rs1559810;rs7640550 |
| ENSG00000134321 | RSAD2 | 2 | 7005937 | 7038370 | 4.59E-12 | 0 | 0 | 1 | 2.53E-08 | 0.000329193 | eQTLGen_trans_eQTLs | 4.00E-08 | rs1990760 |
| ENSG00000163001 | CCDC104 | 2 | 55746740 | 55773015 | 0.000457853 | 0 | 0 | 1 | 4.69E-06 | 0.031250935 | eQTLGen_trans_eQTLs | 9.18E-11 | rs6908626 |
| ENSG00000152672 | CLEC4F | 2 | 71035775 | 71047732 | 4.19E-12 | 0 | 0 | 1 | 1.86E-19 | 0 | eQTLGen_trans_eQTLs | 4.84E-12 | rs1559810;rs7640550 |
| ENSG00000196843 | ARID5A | 2 | 97202480 | 97218375 | 0.971125744 | 0 | 0 | 1 | 1.43E-06 | 0.011197797 | eQTLGen_trans_eQTLs | 4.84E-12 | rs1559810;rs7640550 |
| ENSG00000204634 | TBC1D8 | 2 | 101624079 | 101869328 | 5.26E-07 | 0 | 0 | 1 | 3.33E-08 | 0.000420656 | eQTLGen_trans_eQTLs | 4.84E-12 | rs1559810;rs7640550 |
| ENSG00000119147 | C2orf40 | 2 | 106679702 | 106694615 | 0.001678641 | 0 | 0 | 1 | 1.73E-08 | 0.000232666 | eQTLGen_trans_eQTLs | 9.18E-11 | rs6908626 |
| ENSG00000135960 | EDAR | 2 | 109510927 | 109605828 | 8.31E-05 | 0 | 0 | 1 | 5.46E-06 | 0.035420758 | eQTLGen_trans_eQTLs | 9.18E-11 | rs6908626 |
| ENSG00000144136 | SLC20A1 | 2 | 113403434 | 113421404 | 0.725656583 | 0 | 0 | 1 | 2.32E-07 | 0.002217009 | eQTLGen_trans_eQTLs | 4.84E-12 | rs1559810;rs7640550 |
| ENSG00000019169 | MARCO | 2 | 119699742 | 119752236 | 6.42E-08 | 0 | 0 | 1 | 3.89E-17 | 0 | eQTLGen_trans_eQTLs | 4.84E-12 | rs1559810;rs7640550 |
| ENSG00000115267 | IFIH1 | 2 | 163123589 | 163175213 | 6.00E-26 | 1 | 15.24 | 1 | 3.83E-07 | 0.0019146 | eQTLcatalogue/Quach_2016_ge_monocyte_Pam3CSK4 | 3.62E-08 | rs1990760;rs35667974 |
| ENSG00000151690 | MFSD6 | 2 | 191273081 | 191373931 | 0.001846694 | 0 | 0 | 1 | 1.18E-14 | 0 | eQTLGen_cis_eQTLs | 1.13E-12 | rs3024861 |
| ENSG00000189362 | TMEM194B | 2 | 191369068 | 191399448 | NA | 0 | 0 | 1 | 3.25E-07 | 0.00162415 | eQTLcatalogue/GEUVADIS_ge_LCL | NA | rs10199181 |
| ENSG00000115419 | GLS | 2 | 191745553 | 191830278 | 0.9993026 | 0 | 0 | 1 | 2.30E-09 | 2.61E-05 | eQTLGen_cis_eQTLs | 1.13E-12 | rs3024861 |
| ENSG00000115415 | STAT1 | 2 | 191829084 | 191885686 | 0.999994277 | 0 | 0 | 1 | 2.90E-06 | 0.021072797 | eQTLGen_trans_eQTLs | 4.84E-12 | rs1559810;rs7640550 |
| ENSG00000197121 | PGAP1 | 2 | 197697728 | 197792520 | 0.008785882 | 0 | 0 | 1 | 3.21E-06 | 0.022882782 | eQTLGen_trans_eQTLs | 9.18E-11 | rs6908626 |
| ENSG00000163596 | ICA1L | 2 | 203640690 | 203736708 | 0.442027586 | 0 | 0 | 1 | 2.78E-11 | 0 | eQTLGen_cis_eQTLs | 3.84E-09 | rs35988305 |
| ENSG00000173166 | RAPH1 | 2 | 204259068 | 204400133 | 0.957851233 | 0 | 0 | 2 | 4.58E-09 | 3.24E-05 | eQTLGen_cis_eQTLs | 3.84E-09 | rs35988305;rs10497873 |
| ENSG00000178562 | CD28 | 2 | 204571198 | 204603635 | 0.511989197 | 1 | 14.37 | 1 | 9.64E-13 | 0 | eQTLGen_trans_eQTLs | 9.18E-11 | rs35988305;rs6908626 |
| ENSG00000163599 | CTLA4 | 2 | 204732509 | 204738683 | 0.577322419 | 0 | 0 | 6 | 1.96E-43 | 0 | eQTLcatalogue/CEDAR_T-cell_CD4:eQTLcatalogue/Kasela_2017_T-cell_CD4:eQTLcatalogue/Kasela_2017_T-cell_CD8:eQTLGen_cis_eQTLs:eQTLGen_trans_eQTLs | 1.72E-196 | rs58716662;rs3087243;rs35988305;rs231779;rs10497873;rs2476601 |
| ENSG00000163600 | ICOS | 2 | 204801471 | 204826300 | 0.052009229 | 1 | 12.98 | 2 | 1.23E-12 | 0 | eQTLGen_cis_eQTLs:eQTLGen_trans_eQTLs | 9.18E-11 | rs10497873;rs3087243;rs6908626 |
| ENSG00000114948 | ADAM23 | 2 | 207308263 | 207485851 | 0.995404041 | 0 | 0 | 1 | 5.38E-14 | 0 | eQTLGen_trans_eQTLs | 9.18E-11 | rs6908626 |
| ENSG00000115687 | PASK | 2 | 242045514 | 242089679 | 9.06E-20 | 0 | 0 | 1 | 2.79E-07 | 0.002672325 | eQTLGen_trans_eQTLs | 9.18E-11 | rs6908626 |
| ENSG00000157150 | TIMP4 | 3 | 12194551 | 12200851 | 0.003554031 | 0 | 0 | 1 | 1.07E-06 | 0.000528993 | eQTLcatalogue/TwinsUK_ge_fat:PsychENCODE_eQTLs | 2.51E-06 | rs12629337 |
| ENSG00000132170 | PPARG | 3 | 12328867 | 12475855 | 0.668218174 | 0 | 0 | 1 | 4.86E-13 | 0 | eQTLcatalogue/Fairfax_2014_LPS24:eQTLcatalogue/Fairfax_2014_LPS2:eQTLGen_cis_eQTLs | 2.51E-06 | rs12629337 |
| ENSG00000163520 | FBLN2 | 3 | 13573824 | 13679922 | 0.629825153 | 0 | 0 | 1 | 1.22E-06 | 0.009724702 | eQTLGen_trans_eQTLs | 1.72E-196 | rs2476601 |
| ENSG00000188846 | RPL14 | 3 | 40498783 | 40503861 | 0.936435428 | 0 | 0 | 1 | 6.76E-07 | 0.005861256 | eQTLGen_trans_eQTLs | 2.93E-13 | rs11611029 |
| ENSG00000121807 | CCR2 | 3 | 46395225 | 46402419 | 0.011463318 | 0 | 0 | 1 | 2.33E-21 | 0 | eQTLGen_trans_eQTLs | 4.84E-12 | rs1559810;rs7640550 |
| ENSG00000114650 | SCAP | 3 | 47455203 | 47518616 | 0.02255475 | 0 | 0 | 1 | 5.06E-09 | 0.000120815 | eQTLGen_trans_eQTLs | 4.84E-12 | rs1559810;rs7640550 |
| ENSG00000010327 | STAB1 | 3 | 52529354 | 52558511 | 2.80E-25 | 0 | 0 | 1 | 6.47E-06 | 0.040820203 | eQTLGen_trans_eQTLs | 9.18E-11 | rs6908626 |
| ENSG00000189283 | FHIT | 3 | 59735036 | 61237133 | 0.001391839 | 0 | 0 | 1 | 6.54E-06 | 0.041204972 | eQTLGen_trans_eQTLs | 4.84E-12 | rs1559810;rs7640550 |
| ENSG00000114423 | CBLB | 3 | 105374305 | 105588396 | 0.913125167 | 0 | 0 | 2 | 8.00E-10 | 4.00E-06 | eQTLcatalogue/Quach_2016_ge_monocyte_LPS:eQTLcatalogue/Quach_2016_ge_monocyte_naive:eQTLcatalogue/Quach_2016_ge_monocyte_Pam3CSK4:eQTLcatalogue/Quach_2016_ge_monocyte_R848:DICE/T_CD4_naive_activated:eQTLGen_cis_eQTLs | 1.25E-07 | rs13090803;rs1020364 |
| ENSG00000198919 | DZIP3 | 3 | 108308529 | 108413693 | 3.74E-10 | 0 | 0 | 1 | 1.72E-12 | 1.99E-25 | eQTLcatalogue/Lepik_2017_ge_blood:GTEx/v8/Whole_Blood | NA | rs2603121 |
| ENSG00000158186 | MRAS | 3 | 138066539 | 138124375 | 0.859946833 | 0 | 0 | 1 | 3.76E-07 | 0.003387399 | eQTLGen_trans_eQTLs | 4.84E-12 | rs1559810;rs7640550 |
| ENSG00000181631 | P2RY13 | 3 | 151044100 | 151047336 | 0.327928324 | 0 | 0 | 1 | 2.40E-13 | 0 | eQTLGen_trans_eQTLs | 4.84E-12 | rs1559810;rs7640550 |
| ENSG00000113916 | BCL6 | 3 | 187439165 | 187463515 | 0.982525139 | 0 | 0 | 1 | 2.53E-22 | 0 | eQTLGen_cis_eQTLs | 4.84E-12 | rs1559810;rs7640550 |
| ENSG00000145012 | LPP | 3 | 187871072 | 188608460 | 0.582399908 | 1 | 13.87 | 0 | NA | NA | NA | 4.84E-12 | rs1559810;rs7640550 |
| ENSG00000159788 | RGS12 | 4 | 3294755 | 3441640 | 0.000560374 | 0 | 0 | 1 | 2.41E-12 | 0 | eQTLGen_trans_eQTLs | 4.84E-12 | rs1559810;rs7640550 |
| ENSG00000132405 | TBC1D14 | 4 | 6910969 | 7034845 | 0.909001498 | 0 | 0 | 1 | 8.06E-07 | 0.006832236 | eQTLGen_trans_eQTLs | 4.84E-12 | rs1559810;rs7640550 |
| ENSG00000125089 | SH3TC1 | 4 | 8183799 | 8243530 | 1.08E-27 | 0 | 0 | 1 | 4.03E-06 | 0.027692663 | eQTLGen_trans_eQTLs | 4.84E-12 | rs1559810;rs7640550 |
| ENSG00000169116 | PARM1 | 4 | 75858305 | 75975325 | 0.006063363 | 0 | 0 | 1 | 3.17E-06 | 0.022669985 | eQTLGen_trans_eQTLs | 4.84E-12 | rs1559810;rs7640550 |
| ENSG00000138642 | HERC6 | 4 | 89299891 | 89364263 | 3.19E-09 | 0 | 0 | 1 | 1.71E-10 | 0 | eQTLGen_trans_eQTLs | 4.00E-08 | rs1990760 |
| ENSG00000138646 | HERC5 | 4 | 89378268 | 89427314 | 5.21E-05 | 0 | 0 | 2 | 7.23E-28 | 0 | eQTLGen_trans_eQTLs | 3.62E-08 | rs1990760;rs35667974 |
| ENSG00000138801 | PAPSS1 | 4 | 108511433 | 108641608 | 2.62E-06 | 0 | 0 | 1 | 1.97E-06 | 0.015111111 | eQTLGen_trans_eQTLs | 4.84E-12 | rs1559810;rs7640550 |
| ENSG00000145431 | PDGFC | 4 | 157681606 | 157892546 | 0.969061049 | 0 | 0 | 1 | 1.19E-08 | 0.000176254 | eQTLGen_trans_eQTLs | 4.84E-12 | rs1559810;rs7640550 |
| ENSG00000137628 | DDX60 | 4 | 169137444 | 169239958 | 3.67E-10 | 0 | 0 | 1 | 1.58E-06 | 0.012351931 | eQTLGen_trans_eQTLs | 4.00E-08 | rs1990760 |
| ENSG00000129116 | PALLD | 4 | 169418217 | 169849608 | 6.64E-05 | 0 | 0 | 1 | 4.47E-16 | 0 | eQTLGen_trans_eQTLs | 4.84E-12 | rs1559810;rs7640550 |
| ENSG00000113407 | TARS | 5 | 33440802 | 33469644 | 1.19E-09 | 0 | 0 | 1 | 7.35E-12 | 0 | eQTLGen_trans_eQTLs | 4.84E-12 | rs1559810;rs7640550 |
| ENSG00000134352 | IL6ST | 5 | 55230923 | 55290821 | 0.997842778 | 0 | 0 | 1 | 5.68E-06 | 0.036629165 | eQTLGen_trans_eQTLs | 4.84E-12 | rs1559810;rs7640550 |
| ENSG00000113231 | PDE8B | 5 | 76506274 | 76725632 | 0.986862336 | 0 | 0 | 1 | 2.86E-11 | 0 | eQTLGen_trans_eQTLs | 4.84E-12 | rs1559810;rs7640550 |
| ENSG00000174136 | RGMB | 5 | 98104354 | 98134347 | 0.060755423 | 0 | 0 | 1 | 2.24E-10 | 0 | eQTLGen_trans_eQTLs | 9.18E-11 | rs6908626 |
| ENSG00000173930 | SLCO4C1 | 5 | 101569690 | 101632253 | 1.37E-06 | 0 | 0 | 1 | 1.95E-13 | 0 | eQTLGen_cis_eQTLs | 9.83E-09 | rs1991797 |
| ENSG00000145730 | PAM | 5 | 102089685 | 102366809 | 0.030444397 | 0 | 0 | 3 | 3.27170000000001e-310 | 0 | eQTLcatalogue/BLUEPRINT_ge_monocyte:eQTLcatalogue/BLUEPRINT_ge_neutrophil:eQTLcatalogue/CEDAR_monocyte_CD14:eQTLcatalogue/CEDAR_neutrophil_CD15:eQTLcatalogue/CEDAR_transverse_colon:eQTLcatalogue/Fairfax_2014_naive:eQTLcatalogue/GEUVADIS_ge_LCL:eQTLcatalogue/Lepik_2017_ge_blood:eQTLcatalogue/Naranbhai_2015_neutrophil_CD16:eQTLcatalogue/TwinsUK_ge_blood:eQTLGen_cis_eQTLs:GTEx/v8/Whole_Blood | 9.83E-09 | rs1991797 |
| ENSG00000145723 | GIN1 | 5 | 102421704 | 102455855 | 0.006677682 | 0 | 0 | 3 | 2.84E-24 | 0 | eQTLcatalogue/BLUEPRINT_ge_neutrophil:eQTLcatalogue/CEDAR_T-cell_CD4:eQTLcatalogue/CEDAR_T-cell_CD8:eQTLcatalogue/Fairfax_2012_B-cell_CD19:eQTLcatalogue/Fairfax_2014_IFN24:eQTLcatalogue/Fairfax_2014_LPS24:eQTLcatalogue/Fairfax_2014_naive:eQTLGen_cis_eQTLs | 9.83E-09 | rs1991797 |
| ENSG00000145725 | PPIP5K2 | 5 | 102455853 | 102548500 | 4.05E-10 | 0 | 0 | 3 | 7.33E-288 | 0 | eQTLcatalogue/BLUEPRINT_ge_monocyte:eQTLcatalogue/BLUEPRINT_ge_neutrophil:eQTLcatalogue/BLUEPRINT_ge_T-cell:eQTLcatalogue/GENCORD_ge_LCL:eQTLcatalogue/GEUVADIS_ge_LCL:eQTLcatalogue/HipSci_ge_iPSC:eQTLcatalogue/Lepik_2017_ge_blood:eQTLcatalogue/Quach_2016_ge_monocyte_naive:eQTLcatalogue/TwinsUK_ge_fat:eQTLcatalogue/TwinsUK_ge_LCL:eQTLGen_cis_eQTLs:BIOSQTL/BIOS_eQTL_geneLevel:GTEx/v8/Whole_Blood:GTEx/v8/Spleen:GTEx/v8/Thyroid | 9.83E-09 | rs1991797 |
| ENSG00000181751 | C5orf30 | 5 | 102594403 | 102614361 | 0.778880852 | 1 | 16.1 | 3 | 3.47E-08 | 0.00050682 | eQTLcatalogue/GENCORD_ge_T-cell:DICE/T_CD4_memory_TREG:eQTLGen_cis_eQTLs | 9.83E-09 | rs1991797 |
| ENSG00000081059 | TCF7 | 5 | 133450402 | 133487556 | 0.36155457 | 1 | 17.17 | 4 | 9.59E-18 | 0 | eQTLcatalogue/GEUVADIS_ge_LCL:eQTLGen_cis_eQTLs:BIOSQTL/BIOS_eQTL_geneLevel | 7.77E-12 | rs244672 |
| ENSG00000113558 | SKP1 | 5 | 133484633 | 133512729 | 0.224830619 | 0 | 0 | 4 | 6.78E-16 | 0 | eQTLcatalogue/CEDAR_B-cell_CD19:eQTLcatalogue/CEDAR_monocyte_CD14:eQTLcatalogue/CEDAR_neutrophil_CD15:eQTLcatalogue/CEDAR_platelet:eQTLcatalogue/CEDAR_T-cell_CD4:eQTLcatalogue/CEDAR_T-cell_CD8:eQTLcatalogue/CEDAR_transverse_colon:eQTLcatalogue/Fairfax_2012_B-cell_CD19:eQTLcatalogue/Fairfax_2014_IFN24:eQTLcatalogue/Fairfax_2014_LPS24:eQTLcatalogue/Fairfax_2014_LPS2:eQTLcatalogue/Fairfax_2014_naive:eQTLcatalogue/GEUVADIS_ge_LCL:eQTLcatalogue/Lepik_2017_ge_blood:eQTLGen_cis_eQTLs:BIOSQTL/BIOS_eQTL_geneLevel | 7.77E-12 | rs244672 |
| ENSG00000119048 | UBE2B | 5 | 133706870 | 133727683 | 0.320977559 | 0 | 0 | 4 | 3.25E-38 | 0 | eQTLGen_cis_eQTLs | 7.77E-12 | rs244672 |
| ENSG00000164615 | CAMLG | 5 | 134074191 | 134087847 | 0.00402054 | 0 | 0 | 3 | 2.95E-06 | 0.008577305 | eQTLGen_cis_eQTLs | 7.77E-12 | rs244672 |
| ENSG00000113621 | TXNDC15 | 5 | 134209493 | 134237215 | 0.109182713 | 0 | 0 | 2 | 6.34E-06 | 0.018112688 | eQTLGen_cis_eQTLs | 4.55E-07 | rs244672 |
| ENSG00000255833 | TIFAB | 5 | 134779908 | 134788089 | 1.15E-06 | 0 | 0 | 1 | 3.03E-08 | 0.000380618 | eQTLGen_trans_eQTLs | 9.18E-11 | rs6908626 |
| ENSG00000120738 | EGR1 | 5 | 137801179 | 137805004 | 0.674344947 | 0 | 0 | 1 | 4.83E-09 | 0.000121401 | eQTLGen_trans_eQTLs | 4.84E-12 | rs1559810;rs7640550 |
| ENSG00000184584 | TMEM173 | 5 | 138855119 | 138862520 | 0.032330621 | 0 | 0 | 1 | 1.46E-15 | 0 | eQTLGen_trans_eQTLs | 4.84E-12 | rs1559810;rs7640550 |
| ENSG00000112149 | CD83 | 6 | 14117872 | 14137149 | 0.017780686 | 0 | 0 | 1 | 6.49E-06 | 0.04095064 | eQTLGen_trans_eQTLs | 4.84E-12 | rs1559810;rs7640550 |
| ENSG00000112294 | ALDH5A1 | 6 | 24495080 | 24537435 | 0.002904262 | 0 | 0 | 1 | 3.48E-06 | 0.024567365 | eQTLGen_trans_eQTLs | 7.99E-30 | rs231779 |
| ENSG00000124568 | SLC17A1 | 6 | 25783125 | 25832287 | 2.59E-10 | 0 | 0 | 2 | 4.27E-08 | 4.55E-05 | DICE/NK:BIOSQTL/BIOS_eQTL_geneLevel | 9.88E-07 | rs9393681 |
| ENSG00000124564 | SLC17A3 | 6 | 25833294 | 25882514 | 9.88E-14 | 0 | 0 | 2 | 8.31E-64 | 0 | DICE/NK:DICE/T_CD4_TFH:eQTLGen_cis_eQTLs:BIOSQTL/BIOS_eQTL_geneLevel | 9.88E-07 | rs9393681 |
| ENSG00000112343 | TRIM38 | 6 | 25963030 | 25987384 | 4.75E-06 | 1 | 12.87 | 3 | 2.73E-206 | 0 | eQTLcatalogue/BLUEPRINT_ge_T-cell:eQTLcatalogue/CEDAR_monocyte_CD14:eQTLcatalogue/CEDAR_T-cell_CD4:eQTLcatalogue/CEDAR_T-cell_CD8:eQTLcatalogue/GENCORD_ge_fibroblast:eQTLcatalogue/GEUVADIS_ge_LCL:eQTLcatalogue/Nedelec_2016_ge_macrophage_naive:eQTLcatalogue/TwinsUK_ge_LCL:eQTLGen_cis_eQTLs:BIOSQTL/BIOS_eQTL_geneLevel:GTEx/v8/Whole_Blood:GTEx/v8/Thyroid | 9.88E-07 | rs9393681 |
| ENSG00000124693 | HIST1H3B | 6 | 26031817 | 26032288 | 0.307282993 | 0 | 0 | 2 | 2.32E-10 | 0 | eQTLGen_cis_eQTLs | 9.88E-07 | rs9393681 |
| ENSG00000187837 | HIST1H1C | 6 | 26055968 | 26056699 | NA | 0 | 0 | 2 | 3.34E-51 | 0 | eQTLGen_cis_eQTLs:BIOSQTL/BIOS_eQTL_geneLevel | 9.88E-07 | rs9393681 |
| ENSG00000197061 | HIST1H4C | 6 | 26104104 | 26104518 | 8.86E-05 | 0 | 0 | 3 | 5.08E-10 | 2.54E-06 | eQTLcatalogue/Fairfax_2014_IFN24:eQTLcatalogue/Fairfax_2014_LPS24 | 9.88E-07 | rs9393681 |
| ENSG00000158373 | HIST1H2BD | 6 | 26158349 | 26171577 | 0.000277979 | 0 | 0 | 2 | 4.53E-25 | 0 | eQTLGen_cis_eQTLs | 9.88E-07 | rs9393681 |
| ENSG00000197697 | HIST1H2BE | 6 | 26183958 | 26184454 | 0.000307872 | 0 | 0 | 2 | 1.36E-05 | 0.009074793 | BIOSQTL/BIOS_eQTL_geneLevel | 9.88E-07 | rs9393681 |
| ENSG00000196966 | HIST1H3E | 6 | 26225383 | 26225844 | 0.000813429 | 0 | 0 | 1 | 2.25E-09 | 6.07E-06 | eQTLGen_cis_eQTLs:BIOSQTL/BIOS_eQTL_geneLevel | 9.88E-07 | rs9393681 |
| ENSG00000186470 | BTN3A2 | 6 | 26365387 | 26378546 | 0.000124519 | 0 | 0 | 2 | 2.34E-174 | 0 | eQTLcatalogue/Fairfax_2014_naive:eQTLcatalogue/TwinsUK_ge_LCL:eQTLGen_cis_eQTLs | 9.88E-07 | rs9393681 |
| ENSG00000124508 | BTN2A2 | 6 | 26383324 | 26395102 | 0.000150288 | 0 | 0 | 2 | 3.69E-20 | 0 | eQTLGen_cis_eQTLs | 9.88E-07 | rs9393681 |
| ENSG00000112763 | BTN2A1 | 6 | 26458150 | 26476849 | 1.07E-18 | 0 | 0 | 1 | 3.86E-07 | 0.001212721 | eQTLGen_cis_eQTLs | 9.88E-07 | rs9393681 |
| ENSG00000146109 | ABT1 | 6 | 26597180 | 26600278 | 0.027105567 | 0 | 0 | 1 | 1.47E-06 | 0.0073655 | eQTLcatalogue/HipSci_ge_iPSC | 7.47E-05 | rs9393681 |
| ENSG00000124635 | HIST1H2BJ | 6 | 27093676 | 27100541 | 0.478842114 | 0 | 0 | 4 | 5.04E-15 | 0 | eQTLGen_cis_eQTLs | 2.19E-07 | rs57252182;rs72854533;rs1233704 |
| ENSG00000197903 | HIST1H2BK | 6 | 27106073 | 27114619 | 0.490754148 | 0 | 0 | 11 | 9.28E-129 | 0 | eQTLGen_cis_eQTLs:GTEx/v8/Thyroid | 2.19E-07 | rs57252182;rs9393925;rs72854533;rs1233704 |
| ENSG00000112812 | PRSS16 | 6 | 27215480 | 27224403 | 2.24E-06 | 0 | 0 | 10 | 3.12E-25 | 0 | eQTLcatalogue/TwinsUK_ge_LCL:eQTLGen_cis_eQTLs:GTEx/v8/Spleen:GTEx/v8/Thyroid | 3.67E-05 | rs9393925;rs57252182;rs1233704 |
| ENSG00000158553 | POM121L2 | 6 | 27253682 | 27279949 | NA | 0 | 0 | 1 | 0.000302075 | 0.021732156 | PsychENCODE_eQTLs | NA | rs57252182 |
| ENSG00000124613 | ZNF391 | 6 | 27342394 | 27371683 | 9.26E-07 | 0 | 0 | 21 | 2.89E-13 | 0 | eQTLcatalogue/TwinsUK_ge_LCL:eQTLGen_cis_eQTLs:GTEx/v8/Spleen:GTEx/v8/Thyroid | 3.98E-20 | rs57252182;rs1233704;rs9393925;rs72854533;rs733743 |
| ENSG00000096654 | ZNF184 | 6 | 27418522 | 27440897 | 0.133748636 | 0 | 0 | 11 | 1.96E-08 | 0.000102165 | eQTLGen_cis_eQTLs | 3.98E-20 | rs72854533;rs1233704;rs733743 |
| ENSG00000197238 | HIST1H4J | 6 | 27791884 | 27792257 | 0.42375383 | 0 | 0 | 2 | 1.39E-09 | 6.13E-06 | PsychENCODE_eQTLs:BIOSQTL/BIOS_eQTL_geneLevel | 0.003313957 | rs9393925;rs57252182 |
| ENSG00000197914 | HIST1H4K | 6 | 27798952 | 27799305 | 0.429118935 | 0 | 0 | 18 | 8.03E-17 | 0 | eQTLGen_cis_eQTLs:BIOSQTL/BIOS_eQTL_geneLevel | 3.19E-07 | rs57252182;rs9393925;rs1233704;rs9468356 |
| ENSG00000184348 | HIST1H2AK | 6 | 27805658 | 27806117 | 0.068428502 | 0 | 0 | 5 | 1.86E-05 | 0.012127941 | BIOSQTL/BIOS_eQTL_geneLevel | 0.002354082 | rs9393925 |
| ENSG00000198558 | HIST1H4L | 6 | 27840926 | 27841289 | 0.191234624 | 0 | 0 | 1 | 0.000184922 | 0.014322754 | PsychENCODE_eQTLs | 0.003313957 | rs9393925 |
| ENSG00000196331 | HIST1H2BO | 6 | 27861203 | 27861669 | 3.07E-05 | 0 | 0 | 18 | 5.62E-12 | 0 | eQTLGen_cis_eQTLs:BIOSQTL/BIOS_eQTL_geneLevel | 3.98E-20 | rs57252182;rs72854533;rs1233704;rs733743;rs116802478 |
| ENSG00000168131 | OR2B2 | 6 | 27878963 | 27880174 | 6.87E-05 | 2 | 23.2 | 0 | NA | NA | NA | 0.002354082 | rs9393925 |
| ENSG00000197279 | ZNF165 | 6 | 28048753 | 28057341 | 0.000563292 | 2 | 13.56 | 34 | 1.14E-23 | 0 | eQTLcatalogue/BLUEPRINT_ge_neutrophil:eQTLcatalogue/BLUEPRINT_ge_T-cell:eQTLcatalogue/Fairfax_2012_B-cell_CD19:eQTLcatalogue/GEUVADIS_ge_LCL:eQTLcatalogue/Quach_2016_ge_monocyte_Pam3CSK4:PsychENCODE_eQTLs:DICE/B_cell_naive:eQTLGen_cis_eQTLs:BIOSQTL/BIOS_eQTL_geneLevel:GTEx/v8/Thyroid | 3.98E-20 | rs72854533;rs1233704;rs9393925;rs9468356;rs3117343;rs4713186;rs1794588;rs733743;rs116802478;rs57252182 |
| ENSG00000196812 | ZSCAN16 | 6 | 28092338 | 28097860 | 2.67E-06 | 0 | 0 | 33 | 5.64E-71 | 0 | eQTLcatalogue/BLUEPRINT_ge_neutrophil:eQTLcatalogue/CEDAR_transverse_colon:PsychENCODE_eQTLs:eQTLGen_cis_eQTLs:BIOSQTL/BIOS_eQTL_geneLevel | 3.98E-20 | rs9393925;rs1233704;rs9468356;rs57252182;rs72854533;rs733743;rs116802478;rs3117343;rs4713186;rs1794588 |
| ENSG00000198315 | ZKSCAN8 | 6 | 28109688 | 28127250 | 0.000231595 | 2 | 22.3 | 20 | 6.34E-13 | 3.17E-09 | eQTLcatalogue/BLUEPRINT_ge_neutrophil:eQTLcatalogue/BrainSeq_ge_brain:PsychENCODE_eQTLs:DICE/T_CD4_naive_TREG:eQTLGen_cis_eQTLs:GTEx/v8/Thyroid | 2.04E-07 | rs72854533;rs9393925;rs1233704;rs9468356 |
| ENSG00000137185 | ZSCAN9 | 6 | 28192664 | 28201260 | 4.10E-08 | 0 | 0 | 47 | 1.09E-216 | 0 | eQTLcatalogue/CEDAR_rectum:eQTLcatalogue/CEDAR_T-cell_CD4:eQTLcatalogue/Fairfax_2012_B-cell_CD19:eQTLcatalogue/Fairfax_2014_naive:eQTLcatalogue/Lepik_2017_ge_blood:PsychENCODE_eQTLs:DICE/T_CD4_TH1_17:DICE/T_CD4_TH2:DICE/T_CD4_memory_TREG:eQTLGen_cis_eQTLs:BIOSQTL/BIOS_eQTL_geneLevel:GTEx/v8/Whole_Blood:GTEx/v8/Thyroid | 3.98E-20 | rs57252182;rs1233704;rs3117343;rs4713186;rs1794588;rs9468356;rs72854533;rs733743;rs116802478;rs9393925;rs362520;rs209165 |
| ENSG00000187626 | ZKSCAN4 | 6 | 28212401 | 28227011 | 6.74E-05 | 0 | 0 | 43 | 4.86E-103 | 0 | eQTLcatalogue/BLUEPRINT_ge_monocyte:eQTLcatalogue/BLUEPRINT_ge_neutrophil:eQTLcatalogue/Lepik_2017_ge_blood:eQTLcatalogue/Quach_2016_ge_monocyte_naive:DICE/T_CD8_naive:eQTLGen_cis_eQTLs:BIOSQTL/BIOS_eQTL_geneLevel:GTEx/v8/Whole_Blood:GTEx/v8/Spleen | 3.98E-20 | rs9393925;rs1233704;rs9468356;rs3117343;rs57252182;rs72854533;rs733743;rs116802478;rs209165;rs4713186;rs1794588 |
| ENSG00000189134 | NKAPL | 6 | 28227098 | 28228736 | 0.000165621 | 0 | 0 | 14 | 1.08E-07 | 8.32E-05 | eQTLGen_cis_eQTLs:BIOSQTL/BIOS_eQTL_geneLevel | 3.98E-20 | rs72854533;rs733743;rs116802478 |
| ENSG00000137338 | PGBD1 | 6 | 28249314 | 28270326 | 1.47E-05 | 4 | 14.75 | 37 | 1.13E-46 | 0 | eQTLcatalogue/Fairfax_2012_B-cell_CD19:eQTLcatalogue/HipSci_ge_iPSC:PsychENCODE_eQTLs:DICE/T_CD4_memory_TREG:eQTLGen_cis_eQTLs:BIOSQTL/BIOS_eQTL_geneLevel:GTEx/v8/Thyroid | 3.98E-20 | rs72854533;rs9393925;rs733743;rs9468356;rs57252182;rs1233704;rs116802478;rs3117343;rs4713186;rs1794588 |
| ENSG00000235109 | ZSCAN31 | 6 | 28292470 | 28324048 | 3.77E-09 | 4 | 15.37 | 43 | 7.16E-41 | 7.05E-48 | eQTLcatalogue/BrainSeq_ge_brain:eQTLcatalogue/CEDAR_transverse_colon:eQTLcatalogue/TwinsUK_ge_fat:eQTLcatalogue/TwinsUK_ge_skin:PsychENCODE_eQTLs:GTEx/v8/Spleen:GTEx/v8/Thyroid | 3.98E-20 | rs9393925;rs72854533;rs733743;rs1233704;rs116802478;rs3117343;rs4713186;rs57252182;rs9468356;rs1794588 |
| ENSG00000189298 | ZKSCAN3 | 6 | 28317691 | 28336947 | 0.000122608 | 4 | 15.37 | 44 | 3.65E-105 | 0 | eQTLcatalogue/HipSci_ge_iPSC:eQTLcatalogue/TwinsUK_ge_fat:eQTLcatalogue/TwinsUK_ge_LCL:eQTLcatalogue/TwinsUK_ge_skin:PsychENCODE_eQTLs:eQTLGen_cis_eQTLs:BIOSQTL/BIOS_eQTL_geneLevel:GTEx/v8/Whole_Blood:GTEx/v8/Thyroid | 3.98E-20 | rs9393925;rs72854533;rs733743;rs9468356;rs3117343;rs4713186;rs57252182;rs1233704;rs1794588;rs116802478;rs362520;rs209165 |
| ENSG00000158691 | ZSCAN12 | 6 | 28346732 | 28367511 | 1.44E-05 | 2 | 13.14 | 6 | 1.33E-11 | 0 | eQTLcatalogue/Quach_2016_ge_monocyte_naive:DICE/Monocyte_classical:DICE/Monocyte_non_classical:eQTLGen_cis_eQTLs:GTEx/v8/Thyroid | 3.19E-07 | rs72854533;rs733743;rs9468356;rs1233704;rs209165 |
| ENSG00000187987 | ZSCAN23 | 6 | 28399707 | 28411279 | 0.002702951 | 1 | 13.61 | 38 | 5.65E-90 | 0 | eQTLcatalogue/BLUEPRINT_ge_T-cell:eQTLcatalogue/BrainSeq_ge_brain:eQTLcatalogue/GENCORD_ge_LCL:eQTLcatalogue/GEUVADIS_ge_LCL:eQTLcatalogue/HipSci_ge_iPSC:eQTLcatalogue/Schwartzentruber_2018_ge_sensory_neuron:eQTLcatalogue/TwinsUK_ge_LCL:eQTLcatalogue/TwinsUK_ge_skin:PsychENCODE_eQTLs:DICE/T_CD4_naive:DICE/T_CD8_naive:DICE/T_CD4_TFH:DICE/T_CD4_TH17:DICE/T_CD4_memory_TREG:BIOSQTL/BIOS_eQTL_geneLevel:GTEx/v8/Cells_EBV-transformed_lymphocytes:GTEx/v8/Thyroid | 3.98E-20 | rs9468356;rs72854533;rs1233704;rs733743;rs116802478;rs57252182;rs3117343;rs4713186;rs1794588;rs9393925;rs209165;rs9257837 |
| ENSG00000198704 | GPX6 | 6 | 28471073 | 28495992 | 3.14E-05 | 1 | 24 | 0 | NA | NA | NA | 5.47E-07 | rs72854533;rs733743;rs116802478 |
| ENSG00000232040 | SCAND3 | 6 | 28539407 | 28583989 | 9.71E-09 | 2 | 13.8 | 0 | NA | NA | NA | 1.59E-06 | rs72854533;rs733743;rs116802478 |
| ENSG00000204713 | TRIM27 | 6 | 28870779 | 28891766 | 0.878751858 | 0 | 0 | 42 | 3.27170000000001e-310 | 0 | eQTLcatalogue/BLUEPRINT_ge_monocyte:eQTLcatalogue/BLUEPRINT_ge_neutrophil:eQTLcatalogue/Lepik_2017_ge_blood:eQTLcatalogue/Nedelec_2016_ge_macrophage_Listeria:eQTLcatalogue/Nedelec_2016_ge_macrophage_Salmonella:eQTLcatalogue/Quach_2016_ge_monocyte_LPS:eQTLcatalogue/Quach_2016_ge_monocyte_naive:eQTLcatalogue/Quach_2016_ge_monocyte_Pam3CSK4:eQTLcatalogue/Quach_2016_ge_monocyte_R848:eQTLcatalogue/TwinsUK_ge_blood:eQTLcatalogue/TwinsUK_ge_fat:eQTLcatalogue/TwinsUK_ge_skin:DICE/Monocyte_classical:DICE/Monocyte_non_classical:eQTLGen_cis_eQTLs:BIOSQTL/BIOS_eQTL_geneLevel:GTEx/v8/Whole_Blood:GTEx/v8/Spleen:GTEx/v8/Thyroid | 2.25E-15 | rs3117343;rs4713186;rs1794588;rs9393925;rs209165;rs1233387;rs9468356;rs72854533;rs1233704;rs733743;rs116802478;rs362520;rs9257837;rs378956;rs34927823 |
| ENSG00000204709 | C6orf100 | 6 | 28911654 | 28912314 | NA | 0 | 0 | 35 | 4.01E-40 | 2.01E-36 | eQTLcatalogue/Alasoo_2018_ge_macrophage_IFNg_Salmonella:eQTLcatalogue/Alasoo_2018_ge_macrophage_IFNg:eQTLcatalogue/Fairfax_2014_IFN24:eQTLcatalogue/Fairfax_2014_LPS24:eQTLcatalogue/GEUVADIS_ge_LCL:eQTLcatalogue/HipSci_ge_iPSC:eQTLcatalogue/Quach_2016_ge_monocyte_IAV:eQTLcatalogue/TwinsUK_ge_fat:eQTLcatalogue/TwinsUK_ge_LCL:PsychENCODE_eQTLs | 3.98E-20 | rs1233704;rs9468356;rs3117343;rs4713186;rs1794588;rs9393925;rs72854533;rs733743;rs116802478 |
| ENSG00000197935 | ZNF311 | 6 | 28962562 | 28973093 | 0.000129446 | 0 | 0 | 26 | 2.71E-16 | 0 | eQTLGen_cis_eQTLs:BIOSQTL/BIOS_eQTL_geneLevel:GTEx/v8/Spleen:GTEx/v8/Thyroid | 3.98E-20 | rs72854533;rs1233704;rs733743;rs116802478;rs3117343;rs4713186;rs1794588;rs9257837;rs209165;rs362520 |
| ENSG00000243729 | OR5V1 | 6 | 29323007 | 29399744 | 0.003442619 | 1 | 13.02 | 0 | NA | NA | NA | 1.59E-07 | rs9257837 |
| ENSG00000204694 | OR11A1 | 6 | 29393281 | 29424848 | 0.043484403 | 1 | 13.02 | 0 | NA | NA | NA | 1.59E-07 | rs9257837 |
| ENSG00000204681 | GABBR1 | 6 | 29523406 | 29601753 | 0.999969901 | 1 | 12.47 | 9 | 1.99E-25 | 0 | eQTLcatalogue/TwinsUK_ge_LCL:eQTLGen_cis_eQTLs:BIOSQTL/BIOS_eQTL_geneLevel | 6.26E-10 | rs34927823;rs9393925;rs3117343;rs4713186 |
| ENSG00000204657 | OR2H2 | 6 | 29555683 | 29556745 | 0.089945925 | 1 | 12.47 | 7 | 1.05E-05 | 0.007249322 | BIOSQTL/BIOS_eQTL_geneLevel:GTEx/v8/Whole_Blood | 6.26E-10 | rs34927823;rs9393925 |
| ENSG00000204231 | RXRB | 6 | 33161365 | 33168630 | 0.997594351 | 0 | 0 | 3 | 5.48E-07 | 0.001762929 | eQTLGen_cis_eQTLs | 1.08E-18 | rs1704997;rs34859217 |
| ENSG00000204228 | HSD17B8 | 6 | 33172419 | 33174608 | 0.744497984 | 0 | 0 | 2 | 1.12E-05 | 0.031536132 | DICE/T_CD4_naive_TREG:eQTLGen_cis_eQTLs | 1.08E-18 | rs1704997;rs9277936 |
| ENSG00000204227 | RING1 | 6 | 33176272 | 33180499 | 0.915792422 | 0 | 0 | 2 | 4.47E-06 | 0.000952639 | GTEx/v8/Cells_EBV-transformed_lymphocytes:GTEx/v8/Thyroid | 7.84E-07 | rs34859217;rs9277936 |
| ENSG00000223501 | VPS52 | 6 | 33218049 | 33239824 | 0.015963263 | 0 | 0 | 10 | 1.39E-34 | 0 | eQTLGen_cis_eQTLs:BIOSQTL/BIOS_eQTL_geneLevel | 1.08E-18 | rs9277936;rs1704997;rs76766085 |
| ENSG00000231500 | RPS18 | 6 | 33239787 | 33244287 | 0.962013321 | 0 | 0 | 8 | 9.64E-18 | 0 | eQTLcatalogue/Lepik_2017_ge_blood:PsychENCODE_eQTLs:eQTLGen_cis_eQTLs:BIOSQTL/BIOS_eQTL_geneLevel:GTEx/v8/Whole_Blood:GTEx/v8/Spleen:GTEx/v8/Thyroid | 1.08E-18 | rs9277936;rs76766085;rs1704997;rs35964955;rs34859217 |
| ENSG00000235863 | B3GALT4 | 6 | 33244917 | 33252609 | 0.236708748 | 2 | 26.9 | 2 | 1.72E-05 | 0.001776274 | PsychENCODE_eQTLs | 1.08E-18 | rs1704997;rs76766085 |
| ENSG00000227057 | WDR46 | 6 | 33246885 | 33257304 | 8.42E-06 | 2 | 26.9 | 10 | 1.70E-26 | 0 | eQTLcatalogue/BLUEPRINT_ge_T-cell:eQTLGen_cis_eQTLs:BIOSQTL/BIOS_eQTL_geneLevel:GTEx/v8/Thyroid | 1.08E-18 | rs1704997;rs9277936;rs76766085 |
| ENSG00000204220 | PFDN6 | 6 | 33257079 | 33266178 | 0.85942218 | 2 | 26.9 | 0 | NA | NA | NA | 1.08E-18 | rs1704997 |
| ENSG00000237441 | RGL2 | 6 | 33259431 | 33267101 | 0.0747683 | 2 | 26.9 | 0 | NA | NA | NA | 1.08E-18 | rs1704997 |
| ENSG00000231925 | TAPBP | 6 | 33267471 | 33282164 | 0.000393582 | 0 | 0 | 10 | 2.65E-25 | 0 | eQTLGen_cis_eQTLs:BIOSQTL/BIOS_eQTL_geneLevel | 1.08E-18 | rs1704997;rs76766085;rs34859217 |
| ENSG00000236104 | ZBTB22 | 6 | 33282183 | 33285719 | 0.235099437 | 0 | 0 | 6 | 8.83E-13 | 0 | PsychENCODE_eQTLs:eQTLGen_cis_eQTLs | 1.08E-18 | rs76766085;rs34859217;rs1704997 |
| ENSG00000204209 | DAXX | 6 | 33286335 | 33297046 | 0.84273688 | 0 | 0 | 10 | 2.52E-49 | 0 | eQTLcatalogue/BLUEPRINT_ge_T-cell:eQTLGen_cis_eQTLs:BIOSQTL/BIOS_eQTL_geneLevel:GTEx/v8/Whole_Blood | 1.08E-18 | rs1704997;rs76766085;rs34859217 |
| ENSG00000237649 | KIFC1 | 6 | 33359313 | 33377701 | 0.015578034 | 1 | 16.31 | 0 | NA | NA | NA | 0.000261966 | rs76766085 |
| ENSG00000112511 | PHF1 | 6 | 33378176 | 33384230 | 0.999558759 | 3 | 16.31 | 10 | 9.36E-24 | 0 | eQTLcatalogue/Fairfax_2012_B-cell_CD19:eQTLGen_cis_eQTLs:BIOSQTL/BIOS_eQTL_geneLevel | 1.08E-18 | rs76766085;rs1704997;rs9277936 |
| ENSG00000112514 | CUTA | 6 | 33384219 | 33386094 | 1.14E-05 | 2 | 14.1 | 18 | 1.22E-96 | 0 | eQTLcatalogue/Fairfax_2014_IFN24:eQTLcatalogue/Fairfax_2014_LPS24:eQTLcatalogue/Fairfax_2014_naive:eQTLcatalogue/Lepik_2017_ge_blood:eQTLcatalogue/TwinsUK_ge_skin:PsychENCODE_eQTLs:eQTLGen_cis_eQTLs:BIOSQTL/BIOS_eQTL_geneLevel:GTEx/v8/Whole_Blood:GTEx/v8/Thyroid | 1.08E-18 | rs76766085;rs1704997;rs35964955;rs9277936;rs34942621;rs9461896;rs34527391 |
| ENSG00000197283 | SYNGAP1 | 6 | 33387847 | 33421466 | 0.99999845 | 6 | 22.5 | 0 | NA | NA | NA | 1.14E-16 | rs76766085;rs34859217 |
| ENSG00000030110 | BAK1 | 6 | 33540329 | 33548019 | 0.003486548 | 0 | 0 | 24 | 2.95E-101 | 0 | eQTLcatalogue/BLUEPRINT_ge_monocyte:eQTLcatalogue/BLUEPRINT_ge_neutrophil:eQTLcatalogue/GEUVADIS_ge_LCL:eQTLcatalogue/Lepik_2017_ge_blood:eQTLcatalogue/TwinsUK_ge_LCL:eQTLcatalogue/TwinsUK_ge_skin:eQTLGen_cis_eQTLs:BIOSQTL/BIOS_eQTL_geneLevel:GTEx/v8/Cells_EBV-transformed_lymphocytes:GTEx/v8/Whole_Blood:GTEx/v8/Thyroid | 1.14E-16 | rs34859217;rs4713669;rs1547669;rs76766085;rs34942621;rs9461896;rs35964955;rs34527391 |
| ENSG00000204188 | GGNBP1 | 6 | 33551515 | 33556803 | NA | 0 | 0 | 5 | 3.71E-05 | 0.003518259 | PsychENCODE_eQTLs | 1.33E-08 | rs35964955 |
| ENSG00000096433 | ITPR3 | 6 | 33588142 | 33664351 | 4.98E-12 | 6 | 20.2 | 29 | 3.27170000000001e-310 | 0 | eQTLcatalogue/BLUEPRINT_ge_monocyte:eQTLcatalogue/BLUEPRINT_ge_T-cell:eQTLcatalogue/Fairfax_2014_IFN24:eQTLcatalogue/Fairfax_2014_LPS24:eQTLcatalogue/Fairfax_2014_naive:eQTLcatalogue/Lepik_2017_ge_blood:eQTLcatalogue/Nedelec_2016_ge_macrophage_Listeria:eQTLcatalogue/Nedelec_2016_ge_macrophage_Salmonella:eQTLcatalogue/Quach_2016_ge_monocyte_LPS:eQTLcatalogue/TwinsUK_ge_blood:eQTLcatalogue/TwinsUK_ge_skin:DICE/Monocyte_classical:eQTLGen_cis_eQTLs:BIOSQTL/BIOS_eQTL_geneLevel:GTEx/v8/Whole_Blood:GTEx/v8/Spleen:GTEx/v8/Thyroid | 1.08E-18 | rs13219530;rs4713669;rs34942621;rs9461896;rs35964955;rs34527391;rs76766085;rs1547669;rs1704997;rs34859217 |
| ENSG00000137288 | UQCC2 | 6 | 33662070 | 33679504 | 0.059547733 | 7 | 20.2 | 9 | 2.00E-26 | 0 | eQTLcatalogue/CEDAR_rectum:eQTLcatalogue/Fairfax_2012_B-cell_CD19:eQTLcatalogue/Fairfax_2014_LPS24:eQTLcatalogue/Fairfax_2014_LPS2:eQTLcatalogue/GEUVADIS_ge_LCL:eQTLcatalogue/HipSci_ge_iPSC:eQTLcatalogue/Lepik_2017_ge_blood:eQTLcatalogue/Nedelec_2016_ge_macrophage_Listeria:eQTLcatalogue/Nedelec_2016_ge_macrophage_naive:eQTLcatalogue/Nedelec_2016_ge_macrophage_Salmonella:DICE/T_CD4_TH2:eQTLGen_cis_eQTLs:BIOSQTL/BIOS_eQTL_geneLevel:GTEx/v8/Spleen:GTEx/v8/Thyroid | 4.05E-09 | rs13219530;rs4713669;rs34942621;rs9461896;rs35964955;rs34527391 |
| ENSG00000269490 | SBP1 | 6 | 33663196 | 33663474 | 0.002385472 | 4 | 16.57 | 14 | 3.06E-15 | 0 | eQTLGen_cis_eQTLs:BIOSQTL/BIOS_eQTL_geneLevel | 4.05E-09 | rs13219530;rs4713669;rs34942621;rs9461896;rs35964955;rs34527391 |
| ENSG00000161896 | IP6K3 | 6 | 33689444 | 33714762 | 0.048533853 | 8 | 23.2 | 23 | 1.56E-19 | 7.93E-17 | eQTLcatalogue/van_de_Bunt_2015_ge_pancreatic_islet:GTEx/v8/Thyroid | 1.14E-16 | rs4713669;rs13219530;rs34942621;rs9461896;rs35964955;rs34527391;rs76766085 |
| ENSG00000161904 | LEMD2 | 6 | 33738979 | 33756913 | 0.302373361 | 5 | 22 | 19 | 6.80E-81 | 0 | eQTLcatalogue/Lepik_2017_ge_blood:eQTLcatalogue/TwinsUK_ge_skin:PsychENCODE_eQTLs:eQTLGen_cis_eQTLs:BIOSQTL/BIOS_eQTL_geneLevel:GTEx/v8/Thyroid | 4.05E-09 | rs35964955;rs34942621;rs9461896;rs34527391;rs76766085;rs34859217;rs4713669 |
| ENSG00000096395 | MLN | 6 | 33762450 | 33771788 | 0.001658574 | 5 | 22 | 6 | 8.96E-06 | 0.006124537 | BIOSQTL/BIOS_eQTL_geneLevel | 1.33E-08 | rs35964955;rs1547669 |
| ENSG00000146070 | PLA2G7 | 6 | 46671938 | 46703430 | 8.02E-11 | 0 | 0 | 1 | 6.54E-06 | 0.041188453 | eQTLGen_trans_eQTLs | 4.84E-12 | rs1559810;rs7640550 |
| ENSG00000112182 | BACH2 | 6 | 90636248 | 91006627 | 0.874039851 | 1 | 20.4 | 1 | 9.04E-97 | 0 | eQTLcatalogue/BLUEPRINT_ge_T-cell:DICE/T_CD4_naive:DICE/T_CD8_naive:DICE/T_CD4_naive_TREG:eQTLGen_cis_eQTLs:BIOSQTL/BIOS_eQTL_geneLevel | 9.18E-11 | rs6908626 |
| ENSG00000004809 | SLC22A16 | 6 | 110745890 | 110797844 | 2.09E-22 | 0 | 0 | 1 | 7.31E-06 | 0.044805106 | eQTLGen_trans_eQTLs | 3.62E-08 | rs35667974 |
| ENSG00000118514 | ALDH8A1 | 6 | 135238528 | 135271260 | 9.46E-06 | 0 | 0 | 2 | 5.76E-26 | 0 | eQTLcatalogue/TwinsUK_ge_fat:eQTLcatalogue/TwinsUK_ge_skin:eQTLGen_cis_eQTLs:BIOSQTL/BIOS_eQTL_geneLevel | 8.28E-09 | rs12191243 |
| ENSG00000112339 | HBS1L | 6 | 135281516 | 135424194 | 0.924522221 | 0 | 0 | 1 | 6.01E-09 | 4.52E-05 | eQTLGen_cis_eQTLs | 1.65E-07 | rs12191243 |
| ENSG00000135541 | AHI1 | 6 | 135604670 | 135818914 | 3.26E-14 | 0 | 0 | 1 | 5.58E-09 | 3.88E-05 | eQTLGen_cis_eQTLs | 1.65E-07 | rs12191243 |
| ENSG00000071242 | RPS6KA2 | 6 | 166822852 | 167319939 | 0.084700886 | 0 | 0 | 3 | 3.27170000000001e-310 | 0 | eQTLcatalogue/BLUEPRINT_ge_monocyte:eQTLcatalogue/BLUEPRINT_ge_neutrophil:eQTLcatalogue/BLUEPRINT_ge_T-cell:eQTLcatalogue/CEDAR_monocyte_CD14:eQTLcatalogue/CEDAR_neutrophil_CD15:eQTLcatalogue/CEDAR_T-cell_CD4:eQTLcatalogue/Fairfax_2014_naive:eQTLcatalogue/Lepik_2017_ge_blood:DICE/Monocyte_classical:DICE/NK:eQTLGen_cis_eQTLs:BIOSQTL/BIOS_eQTL_geneLevel:GTEx/v8/Whole_Blood | 2.44E-18 | rs415890;rs968334;rs204295;rs150112 |
| ENSG00000249141 | RP11-514O12.4 | 6 | 167271582 | 167369612 | NA | 0 | 0 | 3 | 3.27167000000001e-310 | 0 | eQTLcatalogue/BLUEPRINT_ge_neutrophil:BIOSQTL/BIOS_eQTL_geneLevel | 2.44E-18 | rs415890;rs968334;rs204295;rs150112 |
| ENSG00000026297 | RNASET2 | 6 | 167342992 | 167370679 | 0.009480406 | 1 | 12.46 | 3 | 3.27167000000001e-310 | 0 | eQTLcatalogue/BLUEPRINT_ge_monocyte:eQTLcatalogue/BLUEPRINT_ge_neutrophil:eQTLcatalogue/BLUEPRINT_ge_T-cell:eQTLcatalogue/CEDAR_neutrophil_CD15:eQTLcatalogue/CEDAR_platelet:eQTLcatalogue/CEDAR_T-cell_CD4:eQTLcatalogue/CEDAR_T-cell_CD8:eQTLcatalogue/CEDAR_transverse_colon:eQTLcatalogue/Fairfax_2014_IFN24:eQTLcatalogue/Fairfax_2014_LPS24:eQTLcatalogue/Fairfax_2014_LPS2:eQTLcatalogue/Fairfax_2014_naive:eQTLcatalogue/GENCORD_ge_fibroblast:eQTLcatalogue/GENCORD_ge_T-cell:eQTLcatalogue/HipSci_ge_iPSC:eQTLcatalogue/Kasela_2017_T-cell_CD4:eQTLcatalogue/Kasela_2017_T-cell_CD8:eQTLcatalogue/Lepik_2017_ge_blood:eQTLcatalogue/Naranbhai_2015_neutrophil_CD16:eQTLcatalogue/Nedelec_2016_ge_macrophage_naive:eQTLcatalogue/Nedelec_2016_ge_macrophage_Salmonella:eQTLcatalogue/Quach_2016_ge_monocyte_IAV:eQTLcatalogue/Quach_2016_ge_monocyte_naive:eQTLcatalogue/Quach_2016_ge_monocyte_Pam3CSK4:eQTLcatalogue/TwinsUK_ge_blood:eQTLcatalogue/TwinsUK_ge_skin:PsychENCODE_eQTLs:scRNA_eQTLs/T_CD4:scRNA_eQTLs/PBMC:DICE/B_cell_naive:DICE/T_CD4_naive:DICE/T_CD4_naive_activated:DICE/T_CD8_naive:DICE/T_CD8_naive_activated:DICE/Monocyte_classical:DICE/NK:DICE/T_CD4_TFH:DICE/T_CD4_TH1:DICE/T_CD4_TH17:DICE/T_CD4_TH1_17:DICE/T_CD4_TH2:DICE/T_CD4_memory_TREG:DICE/T_CD4_naive_TREG:eQTLGen_cis_eQTLs:BIOSQTL/BIOS_eQTL_geneLevel:GTEx/v8/Whole_Blood:GTEx/v8/Thyroid | 2.44E-18 | rs415890;rs968334;rs204295;rs150112 |
| ENSG00000213066 | FGFR1OP | 6 | 167412670 | 167466201 | 0.71081771 | 1 | 13.78 | 3 | 3.19E-28 | 0 | eQTLcatalogue/Lepik_2017_ge_blood:eQTLGen_cis_eQTLs:BIOSQTL/BIOS_eQTL_geneLevel:GTEx/v8/Whole_Blood:GTEx/v8/Thyroid | 2.44E-18 | rs968334;rs204295;rs150112;rs415890 |
| ENSG00000112486 | CCR6 | 6 | 167525295 | 167553184 | 0.066481499 | 0 | 0 | 3 | 2.09E-113 | 0 | eQTLcatalogue/Fairfax_2012_B-cell_CD19:DICE/T_CD4_TFH:DICE/T_CD4_TH17:DICE/T_CD4_TH1_17:eQTLGen_cis_eQTLs:BIOSQTL/BIOS_eQTL_geneLevel:GTEx/v8/Whole_Blood:GTEx/v8/Spleen | 2.44E-18 | rs415890;rs968334;rs204295;rs150112 |
| ENSG00000130396 | MLLT4 | 6 | 168227602 | 168372703 | 0.999998022 | 0 | 0 | 3 | 5.20E-19 | 0 | eQTLGen_cis_eQTLs | 2.44E-18 | rs415890;rs968334;rs204295;rs150112 |
| ENSG00000164916 | FOXK1 | 7 | 4683388 | 4811074 | 0.962670921 | 1 | 12.98 | 1 | 1.03E-262 | 0 | eQTLcatalogue/BLUEPRINT_ge_neutrophil:eQTLcatalogue/BLUEPRINT_ge_T-cell:eQTLcatalogue/CEDAR_T-cell_CD4:eQTLcatalogue/Fairfax_2014_LPS24:eQTLcatalogue/Lepik_2017_ge_blood:eQTLGen_cis_eQTLs:BIOSQTL/BIOS_eQTL_geneLevel:GTEx/v8/Whole_Blood | 4.26E-08 | rs10277273 |
| ENSG00000106049 | HIBADH | 7 | 27565061 | 27702614 | 0.250720099 | 0 | 0 | 1 | 2.49E-09 | 7.35E-05 | eQTLGen_trans_eQTLs | 4.84E-12 | rs1559810;rs7640550 |
| ENSG00000106066 | CPVL | 7 | 29034847 | 29235067 | 4.07E-07 | 0 | 0 | 1 | 2.24E-06 | 0.016904416 | eQTLGen_trans_eQTLs | 4.84E-12 | rs1559810;rs7640550 |
| ENSG00000106341 | PPP1R17 | 7 | 31726329 | 31748069 | 0.000392906 | 0 | 0 | 1 | 1.79E-08 | 0.00023227 | eQTLGen_trans_eQTLs | 4.84E-12 | rs1559810;rs7640550 |
| ENSG00000155849 | ELMO1 | 7 | 36893961 | 37488852 | 0.993000632 | 0 | 0 | 1 | 2.33E-21 | 1.16E-17 | eQTLcatalogue/BLUEPRINT_ge_T-cell:eQTLcatalogue/CEDAR_ileum:eQTLcatalogue/CEDAR_T-cell_CD8:eQTLcatalogue/CEDAR_transverse_colon:eQTLcatalogue/Kasela_2017_T-cell_CD4:eQTLcatalogue/Lepik_2017_ge_blood | NA | rs60600003;rs10279209 |
| ENSG00000105851 | PIK3CG | 7 | 106505723 | 106547590 | 0.563963871 | 0 | 0 | 1 | 5.08E-08 | 0.000694541 | eQTLGen_trans_eQTLs | 4.84E-12 | rs1559810;rs7640550 |
| ENSG00000173114 | LRRN3 | 7 | 110731062 | 110765510 | 0.68645235 | 0 | 0 | 1 | 7.50E-25 | 0 | eQTLGen_trans_eQTLs | 9.18E-11 | rs6908626 |
| ENSG00000128604 | IRF5 | 7 | 128577666 | 128590089 | 0.288476016 | 0 | 0 | 3 | 7.81E-21 | 0 | eQTLcatalogue/GEUVADIS_ge_LCL:PsychENCODE_eQTLs:eQTLGen_cis_eQTLs:BIOSQTL/BIOS_eQTL_geneLevel:GTEx/v8/Whole_Blood:GTEx/v8/Thyroid | 1.04E-12 | rs13246321;rs34644138;rs12674059 |
| ENSG00000064419 | TNPO3 | 7 | 128594948 | 128695198 | 0.001389201 | 1 | 14.35 | 3 | 6.92E-206 | 0 | eQTLcatalogue/BLUEPRINT_ge_monocyte:eQTLcatalogue/BLUEPRINT_ge_neutrophil:eQTLcatalogue/CEDAR_neutrophil_CD15:eQTLcatalogue/Lepik_2017_ge_blood:eQTLcatalogue/TwinsUK_ge_blood:eQTLGen_cis_eQTLs:BIOSQTL/BIOS_eQTL_geneLevel:GTEx/v8/Whole_Blood:GTEx/v8/Thyroid | 1.04E-12 | rs13246321;rs34644138;rs12674059 |
| ENSG00000158457 | TSPAN33 | 7 | 128784712 | 128808671 | 0.000103178 | 0 | 0 | 3 | 6.65E-21 | 0 | eQTLcatalogue/Fairfax_2014_IFN24:eQTLGen_cis_eQTLs:GTEx/v8/Thyroid | 1.04E-12 | rs12674059;rs13246321;rs34644138 |
| ENSG00000128602 | SMO | 7 | 128828713 | 128853386 | 0.063711815 | 0 | 0 | 1 | 3.42E-14 | 7.40E-27 | GTEx/v8/Thyroid | 9.87E-08 | rs12674059 |
| ENSG00000133561 | GIMAP6 | 7 | 150322463 | 150329473 | 0.09734362 | 0 | 0 | 1 | 4.87E-08 | 0.000641741 | eQTLGen_trans_eQTLs | 4.84E-12 | rs1559810;rs7640550 |
| ENSG00000186480 | INSIG1 | 7 | 155089486 | 155101945 | 0.124543666 | 0 | 0 | 1 | 2.61E-08 | 0.000358209 | eQTLGen_trans_eQTLs | 4.84E-12 | rs1559810;rs7640550 |
| ENSG00000268955 | LRLE1 | 8 | 8046158 | 8046331 | NA | 0 | 0 | 3 | 1.92E-11 | 0 | eQTLcatalogue/BLUEPRINT_ge_neutrophil:BIOSQTL/BIOS_eQTL_geneLevel | 2.04E-07 | rs7826238;rs2945250 |
| ENSG00000182319 | SGK223 | 8 | 8175258 | 8244008 | 5.12E-20 | 0 | 0 | 3 | 2.34E-36 | 0 | eQTLGen_cis_eQTLs:BIOSQTL/BIOS_eQTL_geneLevel | 2.04E-07 | rs2945250;rs7826238 |
| ENSG00000253958 | CLDN23 | 8 | 8559448 | 8561616 | 2.47E-05 | 0 | 0 | 3 | 3.81E-16 | 0 | eQTLcatalogue/CEDAR_monocyte_CD14:eQTLcatalogue/Fairfax_2014_naive:BIOSQTL/BIOS_eQTL_geneLevel | 2.04E-07 | rs7826238;rs2945250 |
| ENSG00000147324 | MFHAS1 | 8 | 8640864 | 8751155 | 2.23E-05 | 0 | 0 | 3 | 3.32E-48 | 0 | eQTLcatalogue/Fairfax_2012_B-cell_CD19:eQTLcatalogue/Fairfax_2014_LPS2:eQTLcatalogue/Lepik_2017_ge_blood:DICE/T_CD4_TH1:DICE/T_CD4_TH2:DICE/T_CD4_memory_TREG:eQTLGen_cis_eQTLs | 2.04E-07 | rs2945250;rs7826238 |
| ENSG00000104626 | ERI1 | 8 | 8859657 | 8974256 | 0.000454685 | 0 | 0 | 3 | 9.98E-14 | 4.99E-10 | eQTLcatalogue/Fairfax_2014_IFN24:eQTLcatalogue/Fairfax_2014_LPS24:eQTLcatalogue/Fairfax_2014_LPS2:eQTLcatalogue/Fairfax_2014_naive:eQTLcatalogue/GENCORD_ge_fibroblast:eQTLGen_cis_eQTLs | 2.04E-07 | rs2945250;rs7826238 |
| ENSG00000175806 | MSRA | 8 | 9911778 | 10286401 | 1.70E-06 | 0 | 0 | 3 | 1.27E-144 | 0 | eQTLcatalogue/CEDAR_T-cell_CD4:eQTLcatalogue/Fairfax_2012_B-cell_CD19:eQTLcatalogue/Fairfax_2014_naive:eQTLGen_cis_eQTLs | 1.58E-06 | rs4841567;rs7004825 |
| ENSG00000254093 | PINX1 | 8 | 10622473 | 10697394 | 4.94E-09 | 0 | 0 | 5 | 2.94E-07 | 4.34E-05 | PsychENCODE_eQTLs | 1.58E-06 | rs4841567;rs7004825;rs11250127 |
| ENSG00000171044 | XKR6 | 8 | 10753555 | 11058875 | 0.956776701 | 3 | 14.04 | 7 | 4.22E-23 | 0 | eQTLcatalogue/CEDAR_B-cell_CD19:eQTLcatalogue/CEDAR_T-cell_CD4:eQTLcatalogue/CEDAR_T-cell_CD8:eQTLcatalogue/Fairfax_2012_B-cell_CD19:eQTLcatalogue/Fairfax_2014_naive:eQTLcatalogue/Kasela_2017_T-cell_CD4:eQTLcatalogue/Kasela_2017_T-cell_CD8:PsychENCODE_eQTLs:eQTLGen_cis_eQTLs:BIOSQTL/BIOS_eQTL_geneLevel | 1.58E-06 | rs4841567;rs7004825;rs11250127 |
| ENSG00000215346 | AF131215.5 | 8 | 10983980 | 10987745 | NA | 0 | 0 | 2 | 0.000237787 | 0.017752174 | PsychENCODE_eQTLs | 1.58E-06 | rs4841567;rs7004825 |
| ENSG00000104643 | MTMR9 | 8 | 11141925 | 11185646 | 0.210866149 | 1 | 22 | 8 | 3.57E-21 | 0 | eQTLcatalogue/CEDAR_T-cell_CD4:eQTLcatalogue/Kasela_2017_T-cell_CD4:eQTLcatalogue/Nedelec_2016_ge_macrophage_Listeria:eQTLcatalogue/Nedelec_2016_ge_macrophage_Salmonella:eQTLGen_cis_eQTLs:BIOSQTL/BIOS_eQTL_geneLevel:GTEx/v8/Thyroid | 1.58E-06 | rs11250127;rs4841567;rs7004825;rs4841566 |
| ENSG00000177710 | SLC35G5 | 8 | 11188397 | 11189717 | 0.027484635 | 1 | 22 | 8 | 5.08E-59 | 0 | eQTLcatalogue/BLUEPRINT_ge_T-cell:eQTLcatalogue/van_de_Bunt_2015_ge_pancreatic_islet:eQTLGen_cis_eQTLs:BIOSQTL/BIOS_eQTL_geneLevel:GTEx/v8/Whole_Blood:GTEx/v8/Spleen:GTEx/v8/Thyroid | 1.58E-06 | rs11250127;rs4841567;rs7004825;rs4841566 |
| ENSG00000184608 | C8orf12 | 8 | 11225911 | 11296167 | 0.31126891 | 2 | 14.74 | 1 | 0.00018401 | 0.00295771 | GTEx/v8/Thyroid | 1.28E-05 | rs11250127 |
| ENSG00000154319 | FAM167A | 8 | 11278972 | 11332224 | 0.000192998 | 0 | 0 | 8 | 1.38E-242 | 0 | eQTLcatalogue/CEDAR_B-cell_CD19:eQTLcatalogue/Fairfax_2012_B-cell_CD19:eQTLcatalogue/Fairfax_2014_naive:eQTLcatalogue/GENCORD_ge_LCL:eQTLcatalogue/GEUVADIS_ge_LCL:eQTLcatalogue/Lepik_2017_ge_blood:eQTLcatalogue/TwinsUK_ge_blood:eQTLcatalogue/TwinsUK_ge_LCL:PsychENCODE_eQTLs:eQTLGen_cis_eQTLs:BIOSQTL/BIOS_eQTL_geneLevel:GTEx/v8/Cells_EBV-transformed_lymphocytes:GTEx/v8/Whole_Blood | 1.58E-06 | rs4841567;rs7004825;rs11250127;rs4841566 |
| ENSG00000136573 | BLK | 8 | 11351510 | 11422113 | 2.86E-07 | 1 | 12.6 | 8 | 1.45E-178 | 0 | eQTLcatalogue/BLUEPRINT_ge_T-cell:eQTLcatalogue/CEDAR_B-cell_CD19:eQTLcatalogue/GENCORD_ge_LCL:eQTLcatalogue/GENCORD_ge_T-cell:eQTLcatalogue/GEUVADIS_ge_LCL:eQTLcatalogue/Kasela_2017_T-cell_CD4:eQTLcatalogue/Kasela_2017_T-cell_CD8:eQTLcatalogue/Lepik_2017_ge_blood:eQTLcatalogue/TwinsUK_ge_LCL:eQTLGen_cis_eQTLs:BIOSQTL/BIOS_eQTL_geneLevel:GTEx/v8/Cells_EBV-transformed_lymphocytes:GTEx/v8/Whole_Blood:GTEx/v8/Spleen | 1.58E-06 | rs4841566;rs4841567;rs7004825;rs11250127 |
| ENSG00000154328 | NEIL2 | 8 | 11627148 | 11644855 | 2.11E-09 | 0 | 0 | 8 | 4.38E-53 | 0 | eQTLcatalogue/BLUEPRINT_ge_monocyte:eQTLcatalogue/BLUEPRINT_ge_T-cell:eQTLcatalogue/CEDAR_T-cell_CD4:eQTLcatalogue/Kasela_2017_T-cell_CD4:eQTLcatalogue/Nedelec_2016_ge_macrophage_Salmonella:DICE/NK:DICE/T_CD4_TH1:DICE/T_CD4_TH2:eQTLGen_cis_eQTLs:BIOSQTL/BIOS_eQTL_geneLevel:GTEx/v8/Whole_Blood:GTEx/v8/Thyroid | 1.58E-06 | rs4841567;rs7004825;rs11250127;rs4841566 |
| ENSG00000079459 | FDFT1 | 8 | 11653082 | 11696818 | NA | 0 | 0 | 8 | 3.10E-224 | 0 | eQTLcatalogue/BLUEPRINT_ge_T-cell:eQTLcatalogue/CEDAR_B-cell_CD19:eQTLcatalogue/CEDAR_T-cell_CD4:eQTLcatalogue/CEDAR_T-cell_CD8:eQTLcatalogue/Fairfax_2012_B-cell_CD19:eQTLcatalogue/Fairfax_2014_naive:eQTLcatalogue/Kasela_2017_T-cell_CD4:eQTLcatalogue/Kasela_2017_T-cell_CD8:eQTLcatalogue/Lepik_2017_ge_blood:eQTLGen_cis_eQTLs:GTEx/v8/Whole_Blood | 1.58E-06 | rs4841567;rs7004825;rs11250127;rs4841566 |
| ENSG00000255046 | RP11-297N6.4 | 8 | 11655437 | 11660077 | NA | 0 | 0 | 6 | 3.31E-14 | 0 | eQTLGen_cis_eQTLs:GTEx/v8/Whole_Blood | 1.58E-06 | rs4841567;rs7004825;rs11250127 |
| ENSG00000164733 | CTSB | 8 | 11700033 | 11726957 | NA | 0 | 0 | 8 | 6.07E-23 | 0 | eQTLcatalogue/BLUEPRINT_ge_monocyte:eQTLcatalogue/Fairfax_2014_LPS2:eQTLcatalogue/Lepik_2017_ge_blood:eQTLcatalogue/TwinsUK_ge_skin:PsychENCODE_eQTLs:eQTLGen_cis_eQTLs | 1.58E-06 | rs4841567;rs7004825;rs11250127;rs4841566 |
| ENSG00000255098 | RP11-481A20.11 | 8 | 11870545 | 11873043 | NA | 0 | 0 | 4 | 5.42E-11 | 1.35E-08 | PsychENCODE_eQTLs | 1.58E-06 | rs7004825;rs4841567;rs11250127;rs4841566 |
| ENSG00000223443 | USP17L2 | 8 | 11994677 | 11996586 | 0.013858726 | 0 | 0 | 1 | 0.000369023 | 0.02569514 | PsychENCODE_eQTLs | 1.58E-06 | rs4841567;rs7004825 |
| ENSG00000129422 | MTUS1 | 8 | 17501304 | 17658426 | 1.14E-05 | 0 | 0 | 1 | 6.92E-06 | 0.04297211 | eQTLGen_trans_eQTLs | 9.18E-11 | rs6908626 |
| ENSG00000168546 | GFRA2 | 8 | 21547915 | 21669869 | 0.337127995 | 0 | 0 | 1 | 2.76E-30 | 0 | eQTLGen_trans_eQTLs | 4.84E-12 | rs1559810;rs7640550 |
| ENSG00000120915 | EPHX2 | 8 | 27348296 | 27403081 | 4.92E-13 | 0 | 0 | 1 | 9.95E-07 | 0.008143921 | eQTLGen_trans_eQTLs | 9.18E-11 | rs6908626 |
| ENSG00000133872 | TMEM66 | 8 | 29920528 | 29940723 | 0.009701338 | 0 | 0 | 1 | 7.54E-06 | 0.046200504 | eQTLGen_trans_eQTLs | 9.18E-11 | rs6908626 |
| ENSG00000157168 | NRG1 | 8 | 31496902 | 32622548 | 0.947798762 | 0 | 0 | 1 | 3.24E-34 | 0 | eQTLGen_trans_eQTLs | 4.84E-12 | rs1559810;rs7640550 |
| ENSG00000070718 | AP3M2 | 8 | 42010464 | 42029191 | 4.40E-05 | 0 | 0 | 1 | 4.88E-08 | 0.000641652 | eQTLGen_trans_eQTLs | 9.18E-11 | rs6908626 |
| ENSG00000178538 | CA8 | 8 | 61099906 | 61193971 | 0.783927467 | 0 | 0 | 3 | 1.99E-116 | 0 | eQTLcatalogue/Lepik_2017_ge_blood:DICE/Monocyte_classical:DICE/Monocyte_non_classical:eQTLGen_cis_eQTLs | 9.43E-10 | rs6992869 |
| ENSG00000104388 | RAB2A | 8 | 61429416 | 61536186 | 0.970735513 | 1 | 15.7 | 3 | 3.28E-258 | 0 | eQTLcatalogue/Alasoo_2018_ge_macrophage_IFNg_Salmonella:eQTLcatalogue/Alasoo_2018_ge_macrophage_IFNg:eQTLcatalogue/Alasoo_2018_ge_macrophage_naive:eQTLcatalogue/Alasoo_2018_ge_macrophage_Salmonella:eQTLcatalogue/BLUEPRINT_ge_monocyte:eQTLcatalogue/BLUEPRINT_ge_T-cell:eQTLcatalogue/CEDAR_monocyte_CD14:eQTLcatalogue/CEDAR_T-cell_CD4:eQTLcatalogue/CEDAR_T-cell_CD8:eQTLcatalogue/CEDAR_transverse_colon:eQTLcatalogue/Fairfax_2012_B-cell_CD19:eQTLcatalogue/Fairfax_2014_IFN24:eQTLcatalogue/Fairfax_2014_LPS24:eQTLcatalogue/Fairfax_2014_LPS2:eQTLcatalogue/Fairfax_2014_naive:eQTLcatalogue/GENCORD_ge_fibroblast:eQTLcatalogue/GEUVADIS_ge_LCL:eQTLcatalogue/Lepik_2017_ge_blood:eQTLcatalogue/Nedelec_2016_ge_macrophage_Listeria:eQTLcatalogue/Nedelec_2016_ge_macrophage_naive:eQTLcatalogue/Nedelec_2016_ge_macrophage_Salmonella:eQTLcatalogue/Quach_2016_ge_monocyte_LPS:eQTLcatalogue/Quach_2016_ge_monocyte_Pam3CSK4:eQTLcatalogue/Quach_2016_ge_monocyte_R848:eQTLcatalogue/TwinsUK_ge_blood:eQTLcatalogue/TwinsUK_ge_fat:eQTLcatalogue/TwinsUK_ge_LCL:eQTLGen_cis_eQTLs:BIOSQTL/BIOS_eQTL_geneLevel:GTEx/v8/Cells_EBV-transformed_lymphocytes:GTEx/v8/Whole_Blood:GTEx/v8/Spleen:GTEx/v8/Thyroid | 9.43E-10 | rs6992869 |
| ENSG00000156804 | FBXO32 | 8 | 124510129 | 124553446 | 0.972568188 | 0 | 0 | 1 | 9.47E-08 | 0.001083409 | eQTLGen_trans_eQTLs | 9.18E-11 | rs6908626 |
| ENSG00000170873 | MTSS1 | 8 | 125563031 | 125740730 | 0.999823848 | 0 | 0 | 1 | 7.26E-06 | 0.044620036 | eQTLGen_trans_eQTLs | 4.84E-12 | rs1559810;rs7640550 |
| ENSG00000107372 | ZFAND5 | 9 | 74966341 | 74980163 | 0.912585067 | 0 | 0 | 1 | 4.48E-08 | 0.000618499 | eQTLGen_trans_eQTLs | 4.84E-12 | rs1559810;rs7640550 |
| ENSG00000099139 | PCSK5 | 9 | 78505560 | 78977255 | 0.000207779 | 0 | 0 | 1 | 5.65E-06 | 0.03648239 | eQTLGen_trans_eQTLs | 1.72E-196 | rs2476601 |
| ENSG00000119457 | SLC46A2 | 9 | 115641200 | 115653193 | 0.000292934 | 0 | 0 | 1 | 1.17E-07 | 0.001297215 | eQTLGen_trans_eQTLs | 4.84E-12 | rs1559810;rs7640550 |
| ENSG00000119522 | DENND1A | 9 | 126141933 | 126692431 | 0.904475401 | 0 | 0 | 1 | 1.01E-08 | 0.00016276 | eQTLGen_trans_eQTLs | 4.84E-12 | rs1559810;rs7640550 |
| ENSG00000119408 | NEK6 | 9 | 127019885 | 127115586 | 0.644137661 | 2 | 14.29 | 9 | 1.18E-62 | 0 | eQTLcatalogue/Fairfax_2014_IFN24:eQTLcatalogue/Fairfax_2014_naive:eQTLcatalogue/HipSci_ge_iPSC:eQTLcatalogue/Lepik_2017_ge_blood:eQTLcatalogue/Nedelec_2016_ge_macrophage_naive:eQTLcatalogue/Quach_2016_ge_monocyte_IAV:eQTLcatalogue/TwinsUK_ge_skin:PsychENCODE_eQTLs:eQTLGen_cis_eQTLs:BIOSQTL/BIOS_eQTL_geneLevel:GTEx/v8/Whole_Blood:GTEx/v8/Thyroid | 5.15E-11 | rs867610;rs34071757;rs2026191 |
| ENSG00000136930 | PSMB7 | 9 | 127115745 | 127177723 | 0.941261763 | 5 | 19.75 | 9 | 1.40E-27 | 0 | eQTLcatalogue/BLUEPRINT_ge_neutrophil:eQTLcatalogue/CEDAR_neutrophil_CD15:eQTLcatalogue/Quach_2016_ge_monocyte_LPS:eQTLcatalogue/Quach_2016_ge_monocyte_Pam3CSK4:eQTLcatalogue/Quach_2016_ge_monocyte_R848:eQTLcatalogue/TwinsUK_ge_fat:PsychENCODE_eQTLs:eQTLGen_cis_eQTLs:GTEx/v8/Thyroid | 5.15E-11 | rs867610;rs34071757;rs2026191 |
| ENSG00000148200 | NR6A1 | 9 | 127279554 | 127533589 | 0.992320028 | 0 | 0 | 1 | 0.000812327 | 0.048990997 | PsychENCODE_eQTLs | 6.48E-09 | rs867610 |
| ENSG00000119414 | PPP6C | 9 | 127908852 | 127952218 | 0.988609354 | 0 | 0 | 1 | 2.84E-06 | 0.0142191 | eQTLcatalogue/BLUEPRINT_ge_neutrophil | 3.38E-10 | rs867610 |
| ENSG00000136933 | RABEPK | 9 | 127962821 | 127996437 | 4.19E-06 | 0 | 0 | 8 | 5.05E-12 | 0 | eQTLGen_cis_eQTLs | 5.15E-11 | rs867610;rs34071757 |
| ENSG00000015171 | ZMYND11 | 10 | 180405 | 300577 | 0.999963586 | 0 | 0 | 1 | 1.86E-08 | 0.000246875 | eQTLGen_trans_eQTLs | 4.84E-12 | rs1559810;rs7640550 |
| ENSG00000173848 | NET1 | 10 | 5454514 | 5500426 | 0.066854195 | 0 | 0 | 1 | 2.48E-06 | 0.018345776 | eQTLGen_trans_eQTLs | 9.18E-11 | rs6908626 |
| ENSG00000057608 | GDI2 | 10 | 5807186 | 5884095 | 0.987154825 | 0 | 0 | 1 | 1.39E-05 | 0.038880341 | eQTLGen_cis_eQTLs | 6.34E-06 | rs7090504 |
| ENSG00000134460 | IL2RA | 10 | 6052652 | 6104288 | 0.88128872 | 1 | 12.94 | 2 | 1.19E-09 | 3.90E-05 | eQTLcatalogue/TwinsUK_ge_fat:eQTLGen_trans_eQTLs | 1.72E-196 | rs7090504;rs2476601 |
| ENSG00000134453 | RBM17 | 10 | 6130950 | 6159420 | 0.998602405 | 0 | 0 | 1 | 1.10E-09 | 1.31E-05 | eQTLGen_cis_eQTLs | 6.34E-06 | rs7090504 |
| ENSG00000012779 | ALOX5 | 10 | 45869661 | 45941561 | 0.001644393 | 0 | 0 | 1 | 2.12E-07 | 0.002085802 | eQTLGen_trans_eQTLs | 4.84E-12 | rs1559810;rs7640550 |
| ENSG00000150347 | ARID5B | 10 | 63661059 | 63856703 | 0.999889943 | 14 | 20.9 | 18 | 2.99E-68 | 0 | eQTLGen_cis_eQTLs:eQTLGen_trans_eQTLs:BIOSQTL/BIOS_eQTL_geneLevel | 1.72E-196 | rs71508903;rs68156080;rs10821945;rs10821948;rs10761603;rs10761620;rs2476601 |
| ENSG00000182010 | RTKN2 | 10 | 63942794 | 64028466 | 1.31E-14 | 2 | 15.54 | 12 | 3.27170000000001e-310 | 0 | eQTLcatalogue/BLUEPRINT_ge_T-cell:eQTLcatalogue/CEDAR_T-cell_CD4:eQTLcatalogue/GENCORD_ge_T-cell:eQTLcatalogue/Kasela_2017_T-cell_CD4:eQTLcatalogue/Lepik_2017_ge_blood:eQTLcatalogue/Quach_2016_ge_monocyte_IAV:eQTLcatalogue/Quach_2016_ge_monocyte_LPS:eQTLcatalogue/Quach_2016_ge_monocyte_naive:eQTLcatalogue/Quach_2016_ge_monocyte_Pam3CSK4:eQTLcatalogue/Quach_2016_ge_monocyte_R848:PsychENCODE_eQTLs:eQTLGen_cis_eQTLs:eQTLGen_trans_eQTLs:BIOSQTL/BIOS_eQTL_geneLevel:GTEx/v8/Whole_Blood:GTEx/v8/Spleen:GTEx/v8/Thyroid | 1.72E-196 | rs10761620;rs71508903;rs68156080;rs10821948;rs10761603;rs2476601;rs11102694 |
| ENSG00000138311 | ZNF365 | 10 | 64133951 | 64431771 | 3.62E-05 | 0 | 0 | 4 | 3.53E-12 | 1.76E-08 | eQTLcatalogue/Fairfax_2014_LPS24:eQTLcatalogue/Quach_2016_ge_monocyte_IAV:eQTLcatalogue/Quach_2016_ge_monocyte_LPS:eQTLcatalogue/Quach_2016_ge_monocyte_naive:eQTLcatalogue/Quach_2016_ge_monocyte_Pam3CSK4:eQTLcatalogue/Quach_2016_ge_monocyte_R848:eQTLGen_cis_eQTLs:GTEx/v8/Thyroid | 2.69E-11 | rs10761620;rs10821945 |
| ENSG00000181915 | ADO | 10 | 64564516 | 64568238 | 0.745072785 | 0 | 0 | 3 | 2.56E-06 | 0.007398206 | eQTLGen_cis_eQTLs | 4.72E-12 | rs10761603 |
| ENSG00000156510 | HKDC1 | 10 | 70980059 | 71027315 | 1.85E-12 | 0 | 0 | 1 | 1.85E-09 | 5.64E-05 | eQTLGen_trans_eQTLs | 9.18E-11 | rs6908626 |
| ENSG00000107742 | SPOCK2 | 10 | 73818793 | 73848790 | 0.881279639 | 0 | 0 | 1 | 3.96E-06 | 0.027218661 | eQTLGen_trans_eQTLs | 9.18E-11 | rs6908626 |
| ENSG00000133661 | SFTPD | 10 | 81697496 | 81742370 | 0.240356031 | 0 | 0 | 1 | 3.39E-10 | 2.16E-05 | eQTLGen_trans_eQTLs | 4.84E-12 | rs1559810;rs7640550 |
| ENSG00000198682 | PAPSS2 | 10 | 89419370 | 89507462 | 2.50E-07 | 0 | 0 | 1 | 1.03E-08 | 0.00016239 | eQTLGen_trans_eQTLs | 4.84E-12 | rs1559810;rs7640550 |
| ENSG00000185745 | IFIT1 | 10 | 91152303 | 91163745 | 0.0053482 | 0 | 0 | 2 | 1.40E-29 | 0 | eQTLGen_trans_eQTLs | 3.62E-08 | rs1990760;rs35667974 |
| ENSG00000156395 | SORCS3 | 10 | 106400859 | 107024993 | 0.325134264 | 0 | 0 | 1 | 4.06E-09 | 0.000105578 | eQTLGen_trans_eQTLs | 9.18E-11 | rs6908626 |
| ENSG00000151929 | BAG3 | 10 | 121410882 | 121437331 | 0.53203443 | 0 | 0 | 1 | 2.46E-10 | 0 | eQTLGen_trans_eQTLs | 9.18E-11 | rs6908626 |
| ENSG00000151640 | DPYSL4 | 10 | 134000404 | 134019280 | 5.86E-06 | 0 | 0 | 1 | 3.09E-06 | 0.022228493 | eQTLGen_trans_eQTLs | 9.18E-11 | rs6908626 |
| ENSG00000129757 | CDKN1C | 11 | 2904443 | 2907111 | 0.52282169 | 0 | 0 | 1 | 2.51E-09 | 7.35E-05 | eQTLGen_trans_eQTLs | 4.84E-12 | rs1559810;rs7640550 |
| ENSG00000179241 | LDLRAD3 | 11 | 35965531 | 36253686 | 0.02508931 | 0 | 0 | 1 | 3.74E-08 | 0.000500644 | eQTLGen_trans_eQTLs | 4.84E-12 | rs1559810;rs7640550 |
| ENSG00000110031 | LPXN | 11 | 58294344 | 58345693 | 0.017200443 | 0 | 0 | 1 | 6.33E-07 | 0.005560724 | eQTLGen_trans_eQTLs | 9.18E-11 | rs6908626 |
| ENSG00000013725 | CD6 | 11 | 60739115 | 60787849 | 0.004304658 | 0 | 0 | 3 | 9.61E-10 | 3.97E-05 | eQTLGen_trans_eQTLs | 1.72E-196 | rs2476601;rs244672;rs6908626 |
| ENSG00000110448 | CD5 | 11 | 60869867 | 60895324 | 2.73E-05 | 0 | 0 | 1 | 2.35E-13 | 0 | eQTLGen_trans_eQTLs | 1.72E-196 | rs2476601 |
| ENSG00000176485 | PLA2G16 | 11 | 63340667 | 63384355 | 1.19E-05 | 0 | 0 | 2 | 2.72E-07 | 0.000907001 | eQTLGen_cis_eQTLs | 1.11E-06 | rs663743 |
| ENSG00000110011 | DNAJC4 | 11 | 63997750 | 64001758 | 6.44E-06 | 0 | 0 | 3 | 3.25E-05 | 0.019737509 | BIOSQTL/BIOS_eQTL_geneLevel | 1.11E-06 | rs663743 |
| ENSG00000173511 | VEGFB | 11 | 64002010 | 64006259 | 1.52E-06 | 1 | 18.33 | 4 | 4.80E-18 | 0 | eQTLcatalogue/BLUEPRINT_ge_T-cell:eQTLGen_cis_eQTLs:BIOSQTL/BIOS_eQTL_geneLevel | 1.11E-06 | rs663743 |
| ENSG00000173486 | FKBP2 | 11 | 64008475 | 64011604 | 0.307159556 | 1 | 18.33 | 0 | NA | NA | NA | NA | rs663743 |
| ENSG00000173457 | PPP1R14B | 11 | 64011956 | 64014413 | 0.709204424 | 1 | 18.33 | 4 | 1.74E-17 | 0 | eQTLcatalogue/TwinsUK_ge_fat:PsychENCODE_eQTLs:eQTLGen_cis_eQTLs:BIOSQTL/BIOS_eQTL_geneLevel:GTEx/v8/Thyroid | 1.11E-06 | rs663743 |
| ENSG00000149782 | PLCB3 | 11 | 64018995 | 64036622 | 0.9683767 | 1 | 18.33 | 2 | 2.12E-08 | 0.000108474 | eQTLGen_cis_eQTLs | 3.82E-06 | rs663743 |
| ENSG00000002330 | BAD | 11 | 64037302 | 64052176 | 0.000414579 | 0 | 0 | 4 | 1.30E-09 | 5.97E-06 | eQTLGen_cis_eQTLs:BIOSQTL/BIOS_eQTL_geneLevel | 1.11E-06 | rs663743 |
| ENSG00000173264 | GPR137 | 11 | 64037534 | 64056972 | 0.48439827 | 0 | 0 | 4 | 1.07E-18 | 0 | eQTLGen_cis_eQTLs | 1.11E-06 | rs663743 |
| ENSG00000173153 | ESRRA | 11 | 64073044 | 64084215 | 0.527244157 | 0 | 0 | 4 | 1.69E-08 | 2.32E-05 | BIOSQTL/BIOS_eQTL_geneLevel | 1.11E-06 | rs663743 |
| ENSG00000173113 | TRMT112 | 11 | 64083932 | 64085556 | 0.141017363 | 0 | 0 | 4 | 1.50E-97 | 0 | eQTLGen_cis_eQTLs:BIOSQTL/BIOS_eQTL_geneLevel | 1.11E-06 | rs663743 |
| ENSG00000126432 | PRDX5 | 11 | 64085560 | 64089283 | 0.003140381 | 0 | 0 | 4 | 2.31E-86 | 0 | eQTLcatalogue/CEDAR_transverse_colon:eQTLcatalogue/Lepik_2017_ge_blood:eQTLcatalogue/TwinsUK_ge_LCL:eQTLcatalogue/TwinsUK_ge_skin:eQTLGen_cis_eQTLs:BIOSQTL/BIOS_eQTL_geneLevel | 1.11E-06 | rs663743 |
| ENSG00000168071 | CCDC88B | 11 | 64107695 | 64125006 | 0.000929045 | 1 | 13.15 | 4 | 3.27170000000001e-310 | 0 | eQTLcatalogue/BLUEPRINT_ge_monocyte:eQTLcatalogue/BLUEPRINT_ge_T-cell:eQTLcatalogue/BrainSeq_ge_brain:eQTLcatalogue/CEDAR_neutrophil_CD15:eQTLcatalogue/Fairfax_2012_B-cell_CD19:eQTLcatalogue/Fairfax_2014_IFN24:eQTLcatalogue/Fairfax_2014_LPS2:eQTLcatalogue/Fairfax_2014_naive:eQTLcatalogue/Naranbhai_2015_neutrophil_CD16:PsychENCODE_eQTLs:eQTLGen_cis_eQTLs:BIOSQTL/BIOS_eQTL_geneLevel:GTEx/v8/Whole_Blood:GTEx/v8/Spleen:GTEx/v8/Thyroid | 1.11E-06 | rs663743 |
| ENSG00000162302 | RPS6KA4 | 11 | 64126620 | 64139687 | 0.99947454 | 1 | 16.99 | 4 | 1.07E-21 | 0 | eQTLGen_cis_eQTLs:BIOSQTL/BIOS_eQTL_geneLevel | 1.11E-06 | rs663743 |
| ENSG00000084207 | GSTP1 | 11 | 67351066 | 67354131 | 0.115024063 | 0 | 0 | 1 | 8.24E-06 | 0.049565217 | eQTLGen_trans_eQTLs | 9.18E-11 | rs6908626 |
| ENSG00000137496 | IL18BP | 11 | 71709587 | 71716761 | 3.33E-06 | 0 | 0 | 1 | 1.07E-09 | 3.94E-05 | eQTLGen_trans_eQTLs | 4.84E-12 | rs1559810;rs7640550 |
| ENSG00000021300 | PLEKHB1 | 11 | 73357223 | 73373864 | 0.000703307 | 0 | 0 | 1 | 1.34E-08 | 0.000174465 | eQTLGen_trans_eQTLs | 9.18E-11 | rs6908626 |
| ENSG00000167257 | RNF214 | 11 | 117103341 | 117157161 | 0.830018245 | 0 | 0 | 1 | 7.95E-06 | 0.048342095 | eQTLGen_trans_eQTLs | 1.72E-196 | rs2476601 |
| ENSG00000110324 | IL10RA | 11 | 117857063 | 117872196 | 0.968345856 | 0 | 0 | 1 | 7.17E-07 | 0.006166193 | eQTLGen_trans_eQTLs | 1.72E-196 | rs2476601 |
| ENSG00000167286 | CD3D | 11 | 118209669 | 118213459 | 0.006627946 | 0 | 0 | 1 | 4.41E-08 | 0.000619369 | eQTLGen_trans_eQTLs | 2.93E-13 | rs11611029 |
| ENSG00000184232 | OAF | 11 | 120081475 | 120101041 | 0.006168883 | 0 | 0 | 1 | 1.72E-07 | 0.001701921 | eQTLGen_trans_eQTLs | 4.84E-12 | rs1559810;rs7640550 |
| ENSG00000111186 | WNT5B | 12 | 1639057 | 1756409 | 0.835340309 | 0 | 0 | 1 | 1.98E-24 | 0 | eQTLGen_trans_eQTLs | 4.84E-12 | rs1559810;rs7640550 |
| ENSG00000111728 | ST8SIA1 | 12 | 22216707 | 22589975 | 0.211574644 | 0 | 0 | 1 | 1.31E-09 | 5.82E-05 | eQTLGen_trans_eQTLs | 1.72E-196 | rs2476601 |
| ENSG00000060982 | BCAT1 | 12 | 24964295 | 25102393 | 7.05E-06 | 0 | 0 | 1 | 4.61E-06 | 0.03068787 | eQTLGen_trans_eQTLs | 4.84E-12 | rs1559810;rs7640550 |
| ENSG00000066084 | DIP2B | 12 | 50898768 | 51142450 | 0.999668382 | 0 | 0 | 1 | 4.37E-09 | 0.000104987 | eQTLGen_trans_eQTLs | 4.84E-12 | rs1559810;rs7640550 |
| ENSG00000139567 | ACVRL1 | 12 | 52300692 | 52317145 | 0.007941072 | 0 | 0 | 1 | 3.97E-12 | 0 | eQTLGen_trans_eQTLs | 4.84E-12 | rs1559810;rs7640550 |
| ENSG00000167779 | IGFBP6 | 12 | 53491220 | 53496129 | 0.092331337 | 0 | 0 | 1 | 1.99E-06 | 0.015345803 | eQTLGen_trans_eQTLs | 4.84E-12 | rs1559810;rs7640550 |
| ENSG00000135426 | TESPA1 | 12 | 55341802 | 55378530 | 1.92E-05 | 1 | 24.2 | 1 | 7.37E-06 | 1.33E-08 | BIOSQTL/BIOS_eQTL_geneLevel:GTEx/v8/Whole_Blood | 4.02E-08 | rs62623446 |
| ENSG00000258311 | RP11-644F5.10 | 12 | 56109820 | 56118487 | NA | 0 | 0 | 2 | 6.55E-18 | 0 | eQTLGen_cis_eQTLs | 2.93E-13 | rs11611029;rs2271194 |
| ENSG00000135437 | RDH5 | 12 | 56114151 | 56118489 | 3.11E-06 | 0 | 0 | 1 | 1.11E-05 | 0.031028337 | eQTLGen_cis_eQTLs | 2.93E-13 | rs11611029 |
| ENSG00000135414 | GDF11 | 12 | 56137064 | 56150911 | 0.963838119 | 0 | 0 | 1 | 5.91E-08 | 0.000246243 | eQTLGen_cis_eQTLs | 3.34E-12 | rs2271194 |
| ENSG00000170473 | WIBG | 12 | 56295197 | 56326402 | 0.862135667 | 0 | 0 | 1 | 5.34E-06 | 0.015366615 | eQTLGen_cis_eQTLs | 2.93E-13 | rs11611029 |
| ENSG00000185664 | PMEL | 12 | 56347889 | 56367101 | 1.12E-07 | 1 | 22.1 | 0 | NA | NA | NA | 2.93E-13 | rs11611029 |
| ENSG00000123374 | CDK2 | 12 | 56360553 | 56366568 | 0.958299189 | 1 | 22.1 | 0 | NA | NA | NA | 2.93E-13 | rs11611029 |
| ENSG00000111540 | RAB5B | 12 | 56367697 | 56388490 | 0.215698854 | 1 | 22.1 | 2 | 1.85E-07 | 0.000618016 | eQTLGen_cis_eQTLs:GTEx/v8/Thyroid | 2.93E-13 | rs11611029;rs2271194 |
| ENSG00000139531 | SUOX | 12 | 56390964 | 56400425 | 0.002084769 | 0 | 0 | 2 | 3.27170000000001e-310 | 0 | eQTLcatalogue/BLUEPRINT_ge_monocyte:eQTLcatalogue/BLUEPRINT_ge_T-cell:eQTLcatalogue/Fairfax_2012_B-cell_CD19:eQTLcatalogue/Fairfax_2014_LPS2:eQTLcatalogue/Fairfax_2014_naive:eQTLcatalogue/Kasela_2017_T-cell_CD4:eQTLcatalogue/Kasela_2017_T-cell_CD8:eQTLcatalogue/Lepik_2017_ge_blood:eQTLcatalogue/Quach_2016_ge_monocyte_IAV:eQTLcatalogue/TwinsUK_ge_blood:eQTLcatalogue/TwinsUK_ge_LCL:eQTLcatalogue/TwinsUK_ge_skin:DICE/Monocyte_classical:DICE/T_CD4_TH17:DICE/T_CD4_TH1_17:DICE/T_CD4_TH2:DICE/T_CD4_memory_TREG:DICE/T_CD4_naive_TREG:eQTLGen_cis_eQTLs:BIOSQTL/BIOS_eQTL_geneLevel:GTEx/v8/Whole_Blood:GTEx/v8/Spleen:GTEx/v8/Thyroid | 2.93E-13 | rs11611029;rs2271194 |
| ENSG00000123411 | IKZF4 | 12 | 56401443 | 56432219 | 0.953608121 | 0 | 0 | 1 | 1.45E-07 | 0.00187831 | eQTLcatalogue/TwinsUK_ge_fat:eQTLcatalogue/TwinsUK_ge_LCL:DICE/T_CD4_memory_TREG | 3.34E-12 | rs2271194 |
| ENSG00000197728 | RPS26 | 12 | 56435637 | 56438116 | 0.753342825 | 0 | 0 | 2 | 3.27167000000001e-310 | 0 | eQTLcatalogue/Alasoo_2018_ge_macrophage_IFNg_Salmonella:eQTLcatalogue/Alasoo_2018_ge_macrophage_IFNg:eQTLcatalogue/Alasoo_2018_ge_macrophage_naive:eQTLcatalogue/Alasoo_2018_ge_macrophage_Salmonella:eQTLcatalogue/BLUEPRINT_ge_monocyte:eQTLcatalogue/BLUEPRINT_ge_neutrophil:eQTLcatalogue/BLUEPRINT_ge_T-cell:eQTLcatalogue/BrainSeq_ge_brain:eQTLcatalogue/CEDAR_B-cell_CD19:eQTLcatalogue/CEDAR_ileum:eQTLcatalogue/CEDAR_monocyte_CD14:eQTLcatalogue/CEDAR_neutrophil_CD15:eQTLcatalogue/CEDAR_platelet:eQTLcatalogue/CEDAR_rectum:eQTLcatalogue/CEDAR_T-cell_CD4:eQTLcatalogue/CEDAR_T-cell_CD8:eQTLcatalogue/CEDAR_transverse_colon:eQTLcatalogue/Fairfax_2012_B-cell_CD19:eQTLcatalogue/Fairfax_2014_IFN24:eQTLcatalogue/Fairfax_2014_LPS24:eQTLcatalogue/Fairfax_2014_LPS2:eQTLcatalogue/Fairfax_2014_naive:eQTLcatalogue/GENCORD_ge_fibroblast:eQTLcatalogue/GENCORD_ge_LCL:eQTLcatalogue/GENCORD_ge_T-cell:eQTLcatalogue/GEUVADIS_ge_LCL:eQTLcatalogue/HipSci_ge_iPSC:eQTLcatalogue/Kasela_2017_T-cell_CD4:eQTLcatalogue/Kasela_2017_T-cell_CD8:eQTLcatalogue/Lepik_2017_ge_blood:eQTLcatalogue/Naranbhai_2015_neutrophil_CD16:eQTLcatalogue/Nedelec_2016_ge_macrophage_Listeria:eQTLcatalogue/Nedelec_2016_ge_macrophage_naive:eQTLcatalogue/Nedelec_2016_ge_macrophage_Salmonella:eQTLcatalogue/Quach_2016_ge_monocyte_IAV:eQTLcatalogue/Quach_2016_ge_monocyte_LPS:eQTLcatalogue/Quach_2016_ge_monocyte_naive:eQTLcatalogue/Quach_2016_ge_monocyte_Pam3CSK4:eQTLcatalogue/Quach_2016_ge_monocyte_R848:eQTLcatalogue/Schwartzentruber_2018_ge_sensory_neuron:eQTLcatalogue/TwinsUK_ge_blood:eQTLcatalogue/TwinsUK_ge_fat:eQTLcatalogue/TwinsUK_ge_LCL:eQTLcatalogue/TwinsUK_ge_skin:eQTLcatalogue/van_de_Bunt_2015_ge_pancreatic_islet:scRNA_eQTLs/B_cell:scRNA_eQTLs/DC:scRNA_eQTLs/NK:scRNA_eQTLs/Monocyte:scRNA_eQTLs/Classical_Monocyte:scRNA_eQTLs/Non_classical_Monocyte:scRNA_eQTLs/T_CD4:scRNA_eQTLs/T_CD8:scRNA_eQTLs/PBMC:DICE/T_CD8_naive_activated:eQTLGen_cis_eQTLs:BIOSQTL/BIOS_eQTL_geneLevel:GTEx/v8/Cells_EBV-transformed_lymphocytes:GTEx/v8/Whole_Blood:GTEx/v8/Spleen:GTEx/v8/Thyroid | 2.93E-13 | rs11611029;rs2271194 |
| ENSG00000065361 | ERBB3 | 12 | 56473641 | 56497289 | 4.36E-06 | 0 | 0 | 2 | 2.98E-77 | 0 | eQTLcatalogue/GENCORD_ge_T-cell:DICE/T_CD8_naive:DICE/T_CD4_TH1:DICE/T_CD4_TH1_17:DICE/T_CD4_naive_TREG:eQTLGen_cis_eQTLs:BIOSQTL/BIOS_eQTL_geneLevel | 2.93E-13 | rs2271194;rs11611029 |
| ENSG00000257411 | RP11-603J24.9 | 12 | 56495115 | 56503073 | NA | 0 | 0 | 2 | 3.84E-09 | 6.00E-06 | BIOSQTL/BIOS_eQTL_geneLevel | 2.93E-13 | rs11611029;rs2271194 |
| ENSG00000139641 | ESYT1 | 12 | 56512034 | 56538455 | 2.14E-07 | 0 | 0 | 2 | 2.25E-08 | 3.46E-05 | BIOSQTL/BIOS_eQTL_geneLevel | 2.93E-13 | rs11611029;rs2271194 |
| ENSG00000196465 | MYL6B | 12 | 56546040 | 56553431 | 0.003733811 | 0 | 0 | 2 | 2.29E-24 | 0 | eQTLGen_cis_eQTLs | 2.93E-13 | rs11611029;rs2271194 |
| ENSG00000092841 | MYL6 | 12 | 56551945 | 56557280 | 0.25661637 | 0 | 0 | 2 | 8.73E-07 | 0.002766192 | eQTLGen_cis_eQTLs:BIOSQTL/BIOS_eQTL_geneLevel | 2.93E-13 | rs11611029;rs2271194 |
| ENSG00000139613 | SMARCC2 | 12 | 56556767 | 56583351 | 0.999999989 | 0 | 0 | 2 | 4.76E-22 | 0 | eQTLGen_cis_eQTLs:BIOSQTL/BIOS_eQTL_geneLevel | 2.93E-13 | rs11611029;rs2271194 |
| ENSG00000170581 | STAT2 | 12 | 56735381 | 56753939 | 0.041094737 | 0 | 0 | 1 | 4.20E-11 | 0 | eQTLGen_cis_eQTLs | 3.34E-12 | rs2271194 |
| ENSG00000176422 | SPRYD4 | 12 | 56862301 | 56864763 | 0.000589212 | 0 | 0 | 1 | 1.54E-05 | 0.042414934 | eQTLGen_cis_eQTLs | 2.93E-13 | rs11611029 |
| ENSG00000076067 | RBMS2 | 12 | 56915713 | 56984745 | 0.002914798 | 0 | 0 | 1 | 2.01E-07 | 0.000654981 | eQTLGen_cis_eQTLs | 2.93E-13 | rs11611029 |
| ENSG00000166886 | NAB2 | 12 | 57482677 | 57489259 | 0.987535198 | 0 | 0 | 1 | 7.93E-06 | 0.048195859 | eQTLGen_trans_eQTLs | 4.84E-12 | rs1559810;rs7640550 |
| ENSG00000151135 | C12orf23 | 12 | 107349497 | 107372556 | 0.20050487 | 0 | 0 | 1 | 4.25E-07 | 0.003835289 | eQTLGen_trans_eQTLs | 9.18E-11 | rs6908626 |
| ENSG00000151136 | BTBD11 | 12 | 107712190 | 108053419 | 0.993432981 | 0 | 0 | 1 | 3.49E-07 | 0.00321898 | eQTLGen_trans_eQTLs | 9.18E-11 | rs6908626 |
| ENSG00000174600 | CMKLR1 | 12 | 108681821 | 108733118 | 0.068526963 | 0 | 0 | 1 | 1.19E-19 | 0 | eQTLGen_trans_eQTLs | 4.84E-12 | rs1559810;rs7640550 |
| ENSG00000174456 | C12orf76 | 12 | 110465872 | 110511491 | 0.000101301 | 0 | 0 | 1 | 1.35E-05 | 0.037628042 | eQTLGen_cis_eQTLs | 2.20E-14 | rs11065785 |
| ENSG00000111229 | ARPC3 | 12 | 110872630 | 110888227 | 0.931506661 | 0 | 0 | 2 | 1.07E-05 | 0.030100434 | eQTLGen_cis_eQTLs | 1.19E-13 | rs11065822 |
| ENSG00000111231 | GPN3 | 12 | 110890289 | 110907073 | 0.000100472 | 0 | 0 | 4 | 8.78E-108 | 0 | eQTLcatalogue/CEDAR_neutrophil_CD15:eQTLcatalogue/Fairfax_2014_IFN24:eQTLcatalogue/Fairfax_2014_LPS2:eQTLcatalogue/Fairfax_2014_naive:eQTLGen_cis_eQTLs:GTEx/v8/Whole_Blood | 2.20E-14 | rs11065822;rs11065785 |
| ENSG00000204856 | FAM216A | 12 | 110906169 | 110928190 | 0.001116299 | 0 | 0 | 4 | 2.93E-28 | 0 | eQTLGen_cis_eQTLs | 2.20E-14 | rs11065785;rs11065822 |
| ENSG00000111237 | VPS29 | 12 | 110928902 | 110939922 | 0.934744448 | 0 | 0 | 4 | 4.03E-13 | 0 | eQTLGen_cis_eQTLs | 2.20E-14 | rs11065785;rs11065822 |
| ENSG00000204852 | TCTN1 | 12 | 111051832 | 111087235 | 1.87E-09 | 0 | 0 | 5 | 1.05E-10 | 0 | eQTLGen_cis_eQTLs | 2.20E-14 | rs11065785;rs11065822;rs7398796 |
| ENSG00000111245 | MYL2 | 12 | 111348623 | 111358526 | 0.016813134 | 1 | 14.65 | 0 | NA | NA | NA | 2.20E-14 | rs11065785 |
| ENSG00000111249 | CUX2 | 12 | 111471828 | 111788358 | 0.999656385 | 3 | 17.49 | 0 | NA | NA | NA | 1.19E-13 | rs11065822;rs7398796 |
| ENSG00000198324 | FAM109A | 12 | 111798455 | 111806925 | 0.005149827 | 1 | 17.49 | 8 | 4.13E-41 | 0 | eQTLcatalogue/Fairfax_2012_B-cell_CD19:eQTLGen_cis_eQTLs:BIOSQTL/BIOS_eQTL_geneLevel | 2.20E-14 | rs7398796;rs11065785;rs11065822;rs2339718;rs117532831;rs7300285 |
| ENSG00000111252 | SH2B3 | 12 | 111843752 | 111889427 | 0.00592219 | 0 | 0 | 8 | 3.70E-58 | 0 | eQTLGen_cis_eQTLs:BIOSQTL/BIOS_eQTL_geneLevel | 2.20E-14 | rs11065785;rs11065822;rs2339718;rs7398796;rs11066152;rs7300285 |
| ENSG00000204842 | ATXN2 | 12 | 111890018 | 112037480 | 0.999862272 | 1 | 15.87 | 1 | 6.21E-18 | 0 | eQTLcatalogue/BLUEPRINT_ge_neutrophil:eQTLcatalogue/Naranbhai_2015_neutrophil_CD16:eQTLGen_cis_eQTLs:BIOSQTL/BIOS_eQTL_geneLevel | 9.27E-09 | rs117532831 |
| ENSG00000089234 | BRAP | 12 | 112079950 | 112123790 | 0.015703311 | 0 | 0 | 1 | 0.000375561 | 0.00574322 | GTEx/v8/Thyroid | 4.70E-06 | rs7398796 |
| ENSG00000111271 | ACAD10 | 12 | 112123857 | 112194903 | 4.01E-20 | 0 | 0 | 3 | 7.97E-19 | 0 | eQTLGen_cis_eQTLs | 4.70E-06 | rs2339718;rs7398796;rs7300285 |
| ENSG00000257767 | RP11-162P23.2 | 12 | 112191694 | 112229222 | NA | 0 | 0 | 1 | 1.68E-07 | 9.90E-05 | BIOSQTL/BIOS_eQTL_geneLevel | 7.77E-14 | rs11066152 |
| ENSG00000111275 | ALDH2 | 12 | 112204691 | 112247782 | 1.73E-06 | 0 | 0 | 9 | 3.00E-119 | 0 | eQTLcatalogue/BLUEPRINT_ge_neutrophil:eQTLcatalogue/TwinsUK_ge_skin:PsychENCODE_eQTLs:eQTLGen_cis_eQTLs:BIOSQTL/BIOS_eQTL_geneLevel:GTEx/v8/Whole_Blood:GTEx/v8/Thyroid | 7.77E-14 | rs7398796;rs7300285;rs11065785;rs11065822;rs2339718;rs117532831;rs11066152 |
| ENSG00000089022 | MAPKAPK5 | 12 | 112279782 | 112334343 | 0.023422603 | 0 | 0 | 4 | 7.25E-23 | 0 | eQTLcatalogue/BLUEPRINT_ge_T-cell:eQTLcatalogue/Fairfax_2014_naive:eQTLGen_cis_eQTLs | 4.70E-06 | rs7398796;rs7300285;rs2339718 |
| ENSG00000198270 | TMEM116 | 12 | 112369086 | 112450970 | 1.15E-09 | 0 | 0 | 7 | 3.30E-288 | 0 | eQTLcatalogue/BLUEPRINT_ge_monocyte:eQTLcatalogue/BLUEPRINT_ge_T-cell:eQTLcatalogue/Lepik_2017_ge_blood:eQTLcatalogue/Quach_2016_ge_monocyte_LPS:eQTLcatalogue/TwinsUK_ge_fat:DICE/T_CD4_naive:DICE/T_CD8_naive_activated:DICE/T_CD4_TH1:eQTLGen_cis_eQTLs:GTEx/v8/Whole_Blood:GTEx/v8/Thyroid | 1.19E-13 | rs7300285;rs7398796;rs11065822;rs2339718;rs117532831 |
| ENSG00000089248 | ERP29 | 12 | 112451120 | 112461255 | 0.470649236 | 0 | 0 | 1 | 1.08E-11 | 0 | eQTLcatalogue/Lepik_2017_ge_blood:eQTLGen_cis_eQTLs | NA | rs7300285 |
| ENSG00000111300 | NAA25 | 12 | 112464500 | 112546826 | 0.999989964 | 0 | 0 | 3 | 6.07E-24 | 0 | eQTLGen_cis_eQTLs:BIOSQTL/BIOS_eQTL_geneLevel | 4.70E-06 | rs2339718;rs7398796;rs7300285 |
| ENSG00000135148 | TRAFD1 | 12 | 112563305 | 112591407 | 1.83E-05 | 0 | 0 | 3 | 2.47E-17 | 0 | eQTLGen_cis_eQTLs:eQTLGen_trans_eQTLs | 7.77E-14 | rs117532831;rs11066152;rs1559810;rs7640550 |
| ENSG00000173064 | HECTD4 | 12 | 112597992 | 112819896 | 1 | 1 | 13.04 | 0 | NA | NA | NA | NA | rs7300285 |
| ENSG00000179295 | PTPN11 | 12 | 112856155 | 112947717 | 0.999877231 | 0 | 0 | 1 | 5.22E-07 | 0.001695961 | eQTLGen_cis_eQTLs | NA | rs7300285 |
| ENSG00000089127 | OAS1 | 12 | 113344582 | 113369990 | 0.001239241 | 0 | 0 | 1 | 9.32E-08 | 0.000333187 | eQTLGen_cis_eQTLs | NA | rs7300285 |
| ENSG00000111331 | OAS3 | 12 | 113376157 | 113411054 | 1.82E-23 | 0 | 0 | 2 | 5.89E-12 | 0 | eQTLGen_trans_eQTLs | 3.62E-08 | rs1990760;rs35667974 |
| ENSG00000111335 | OAS2 | 12 | 113416200 | 113449528 | 1.89E-14 | 0 | 0 | 1 | 1.03E-10 | 0 | eQTLGen_trans_eQTLs | 4.00E-08 | rs1990760 |
| ENSG00000139410 | SDSL | 12 | 113860042 | 113876081 | 2.99E-09 | 0 | 0 | 1 | 4.88E-28 | 0 | eQTLGen_trans_eQTLs | 4.84E-12 | rs1559810;rs7640550 |
| ENSG00000135114 | OASL | 12 | 121458095 | 121477045 | 5.07E-12 | 0 | 0 | 1 | 5.67E-08 | 0.000796703 | eQTLGen_trans_eQTLs | 4.00E-08 | rs1990760 |
| ENSG00000133106 | EPSTI1 | 13 | 43460524 | 43566407 | 1.35E-18 | 0 | 0 | 1 | 2.16E-08 | 0.000258202 | eQTLGen_trans_eQTLs | 4.00E-08 | rs1990760 |
| ENSG00000139679 | LPAR6 | 13 | 48963707 | 49018840 | 0.030436524 | 0 | 0 | 1 | 5.01E-10 | 2.09E-05 | eQTLGen_trans_eQTLs | 9.18E-11 | rs6908626 |
| ENSG00000139832 | RAB20 | 13 | 111175417 | 111214080 | 0.697708788 | 0 | 0 | 1 | 1.59E-07 | 0.001609337 | eQTLGen_trans_eQTLs | 4.84E-12 | rs1559810;rs7640550 |
| ENSG00000169413 | RNASE6 | 14 | 21249210 | 21250626 | 5.43E-08 | 0 | 0 | 1 | 4.75E-18 | 0 | eQTLGen_trans_eQTLs | 4.84E-12 | rs1559810;rs7640550 |
| ENSG00000165795 | NDRG2 | 14 | 21484922 | 21539031 | 0.126597034 | 0 | 0 | 1 | 1.56E-12 | 0 | eQTLGen_trans_eQTLs | 9.18E-11 | rs6908626 |
| ENSG00000092068 | SLC7A8 | 14 | 23594504 | 23652883 | 0.00016618 | 0 | 0 | 1 | 2.39E-07 | 0.00226715 | eQTLGen_trans_eQTLs | 9.18E-11 | rs6908626 |
| ENSG00000092096 | SLC22A17 | 14 | 23815515 | 23822121 | 0.772286751 | 0 | 0 | 1 | 1.24E-09 | 5.84E-05 | eQTLGen_trans_eQTLs | 9.18E-11 | rs6908626 |
| ENSG00000100453 | GZMB | 14 | 25100160 | 25103473 | 1.79E-06 | 0 | 0 | 1 | 6.22E-06 | 0.039509498 | eQTLGen_trans_eQTLs | 1.72E-196 | rs2476601 |
| ENSG00000100906 | NFKBIA | 14 | 35870717 | 35873955 | 0.979352026 | 0 | 0 | 1 | 6.84E-06 | 0.042583965 | eQTLGen_trans_eQTLs | 4.84E-12 | rs1559810;rs7640550 |
| ENSG00000131979 | GCH1 | 14 | 55308726 | 55369570 | 0.942485933 | 0 | 0 | 1 | 5.79E-06 | 0.037215317 | eQTLGen_trans_eQTLs | 4.84E-12 | rs1559810;rs7640550 |
| ENSG00000182185 | RAD51B | 14 | 68286496 | 69196935 | 1.46E-09 | 5 | 15.3 | 0 | NA | NA | NA | 3.44E-11 | rs10131490 |
| ENSG00000227051 | C14orf132 | 14 | 96505661 | 96560417 | NA | 0 | 0 | 1 | 9.37E-38 | 0 | eQTLGen_trans_eQTLs | 9.18E-11 | rs6908626 |
| ENSG00000166165 | CKB | 14 | 103985996 | 103989448 | 0.850845461 | 0 | 0 | 1 | 2.44E-35 | 0 | eQTLGen_trans_eQTLs | 4.84E-12 | rs1559810;rs7640550 |
| ENSG00000182809 | CRIP2 | 14 | 105939299 | 105946499 | 0.005070278 | 0 | 0 | 1 | 6.46E-06 | 0.01836356 | eQTLGen_cis_eQTLs | NA | rs12895622 |
| ENSG00000185347 | C14orf80 | 14 | 105956192 | 105965912 | 0.001222281 | 0 | 0 | 1 | 1.87E-11 | 0 | eQTLGen_cis_eQTLs:BIOSQTL/BIOS_eQTL_geneLevel | NA | rs12895622 |
| ENSG00000184986 | TMEM121 | 14 | 105992940 | 105996539 | 0.178807336 | 0 | 0 | 1 | 3.16E-60 | 0 | eQTLcatalogue/Lepik_2017_ge_blood:BIOSQTL/BIOS_eQTL_geneLevel:GTEx/v8/Whole_Blood | NA | rs12895622 |
| ENSG00000172575 | RASGRP1 | 15 | 38780304 | 38857776 | 0.18967697 | 0 | 0 | 1 | 2.77E-59 | 0 | eQTLcatalogue/GENCORD_ge_LCL:eQTLcatalogue/GEUVADIS_ge_LCL:eQTLcatalogue/TwinsUK_ge_LCL:eQTLcatalogue/TwinsUK_ge_skin:eQTLGen_cis_eQTLs:BIOSQTL/BIOS_eQTL_geneLevel:GTEx/v8/Cells_EBV-transformed_lymphocytes:GTEx/v8/Spleen | 1.44E-08 | rs6495979;rs56083426 |
| ENSG00000175779 | C15orf53 | 15 | 38988799 | 38992239 | 0.093609809 | 0 | 0 | 1 | 5.45E-06 | 0.015614877 | eQTLGen_cis_eQTLs | 1.44E-08 | rs6495979;rs56083426 |
| ENSG00000104043 | ATP8B4 | 15 | 50150435 | 50475014 | 2.42E-27 | 0 | 0 | 1 | 1.63E-14 | 0 | eQTLGen_trans_eQTLs | 4.84E-12 | rs1559810;rs7640550 |
| ENSG00000140577 | CRTC3 | 15 | 91073157 | 91188577 | 0.983929956 | 0 | 0 | 1 | 2.02E-06 | 0.015566939 | eQTLGen_trans_eQTLs | 4.84E-12 | rs1559810;rs7640550 |
| ENSG00000182511 | FES | 15 | 91426925 | 91439006 | 2.77E-05 | 0 | 0 | 1 | 4.27E-06 | 0.028983632 | eQTLGen_trans_eQTLs | 4.84E-12 | rs1559810;rs7640550 |
| ENSG00000005513 | SOX8 | 16 | 1031808 | 1036979 | 0.87370846 | 0 | 0 | 1 | 4.62E-06 | 0.030756542 | eQTLGen_trans_eQTLs | 4.55E-07 | rs244672 |
| ENSG00000089486 | CDIP1 | 16 | 4560676 | 4588829 | 0.734505012 | 0 | 0 | 1 | 3.17E-11 | 0 | eQTLGen_trans_eQTLs | 4.84E-12 | rs1559810;rs7640550 |
| ENSG00000103274 | NUBP1 | 16 | 10837643 | 10863208 | 6.66E-08 | 0 | 0 | 1 | 3.95E-06 | 0.027191966 | eQTLGen_trans_eQTLs | 9.83E-09 | rs1991797 |
| ENSG00000103528 | SYT17 | 16 | 19179293 | 19279652 | 0.02363635 | 0 | 0 | 1 | 3.02E-06 | 0.021688795 | eQTLGen_trans_eQTLs | 4.84E-12 | rs1559810;rs7640550 |
| ENSG00000140750 | ARHGAP17 | 16 | 24930706 | 25026987 | 0.990465511 | 0 | 0 | 1 | 6.13E-10 | 2.06E-05 | eQTLGen_trans_eQTLs | 4.84E-12 | rs1559810;rs7640550 |
| ENSG00000197272 | IL27 | 16 | 28510683 | 28523372 | 0.927792678 | 0 | 0 | 1 | 3.06E-08 | 0.000394679 | eQTLGen_trans_eQTLs | 4.84E-12 | rs1559810;rs7640550 |
| ENSG00000102934 | PLLP | 16 | 57290004 | 57318599 | 0.519581852 | 0 | 0 | 1 | 1.36E-06 | 0.010747318 | eQTLGen_trans_eQTLs | 9.18E-11 | rs6908626 |
| ENSG00000168404 | MLKL | 16 | 74705753 | 74734858 | 7.48E-14 | 0 | 0 | 1 | 3.40E-29 | 0 | eQTLGen_trans_eQTLs | 4.84E-12 | rs1559810;rs7640550 |
| ENSG00000178573 | MAF | 16 | 79619740 | 79634611 | 0.429001156 | 0 | 0 | 1 | 7.20E-06 | 0.044346509 | eQTLGen_trans_eQTLs | 1.72E-196 | rs2476601 |
| ENSG00000140948 | ZCCHC14 | 16 | 87439852 | 87525651 | 0.948835727 | 0 | 0 | 1 | 8.11E-06 | 0.049030266 | eQTLGen_trans_eQTLs | 9.18E-11 | rs6908626 |
| ENSG00000103335 | PIEZO1 | 16 | 88781751 | 88851619 | 0.53596901 | 0 | 0 | 1 | 2.12E-07 | 0.002085061 | eQTLGen_trans_eQTLs | 9.18E-11 | rs6908626 |
| ENSG00000182853 | VMO1 | 17 | 4688580 | 4689728 | 0.000437154 | 0 | 0 | 1 | 8.38E-24 | 0 | eQTLGen_trans_eQTLs | 4.84E-12 | rs1559810;rs7640550 |
| ENSG00000132514 | CLEC10A | 17 | 6977856 | 6983626 | 4.25E-08 | 0 | 0 | 1 | 1.83E-06 | 0.014137141 | eQTLGen_trans_eQTLs | 9.18E-11 | rs6908626 |
| ENSG00000161944 | ASGR2 | 17 | 7004641 | 7019019 | 6.64E-06 | 0 | 0 | 2 | 9.30E-19 | 0 | eQTLGen_cis_eQTLs:BIOSQTL/BIOS_eQTL_geneLevel | 4.52E-09 | rs61759532 |
| ENSG00000072778 | ACADVL | 17 | 7120444 | 7128592 | 1.31E-07 | 0 | 0 | 3 | 6.06E-18 | 0 | eQTLGen_cis_eQTLs:BIOSQTL/BIOS_eQTL_geneLevel | 4.52E-09 | rs61759532 |
| ENSG00000004975 | DVL2 | 17 | 7128660 | 7137864 | 0.011361464 | 0 | 0 | 1 | 1.02E-05 | 0.028561232 | eQTLGen_cis_eQTLs | 3.24E-07 | rs61759532 |
| ENSG00000262526 | CTD-2545G14.7 | 17 | 7143746 | 7147954 | NA | 0 | 0 | 3 | 6.98E-07 | 0.000587457 | BIOSQTL/BIOS_eQTL_geneLevel | 4.52E-09 | rs61759532 |
| ENSG00000175826 | CTDNEP1 | 17 | 7146910 | 7155810 | 0.972679931 | 0 | 0 | 3 | 1.37E-10 | 0 | BIOSQTL/BIOS_eQTL_geneLevel | 4.52E-09 | rs61759532 |
| ENSG00000262302 | RP1-4G17.5 | 17 | 7150148 | 7165408 | NA | 0 | 0 | 3 | 3.59E-09 | 6.01E-06 | BIOSQTL/BIOS_eQTL_geneLevel | 4.52E-09 | rs61759532 |
| ENSG00000170291 | ELP5 | 17 | 7154735 | 7163259 | 6.48E-06 | 0 | 0 | 3 | 1.43E-18 | 0 | eQTLGen_cis_eQTLs:BIOSQTL/BIOS_eQTL_geneLevel | 4.52E-09 | rs61759532 |
| ENSG00000181885 | CLDN7 | 17 | 7163222 | 7167302 | 0.041384735 | 0 | 0 | 3 | 1.43E-18 | 0 | BIOSQTL/BIOS_eQTL_geneLevel | 4.52E-09 | rs61759532 |
| ENSG00000006047 | YBX2 | 17 | 7191571 | 7197934 | 0.968725623 | 0 | 0 | 3 | 1.12E-10 | 0 | BIOSQTL/BIOS_eQTL_geneLevel:GTEx/v8/Thyroid | 4.52E-09 | rs61759532 |
| ENSG00000132507 | EIF5A | 17 | 7210318 | 7215774 | 0.890876904 | 0 | 0 | 3 | 6.17E-14 | 0 | eQTLcatalogue/BrainSeq_ge_brain:eQTLcatalogue/Fairfax_2014_IFN24:eQTLcatalogue/Fairfax_2014_naive:eQTLcatalogue/GENCORD_ge_LCL:eQTLcatalogue/GEUVADIS_ge_LCL:eQTLcatalogue/Lepik_2017_ge_blood:eQTLGen_cis_eQTLs:BIOSQTL/BIOS_eQTL_geneLevel:GTEx/v8/Thyroid | 4.52E-09 | rs61759532 |
| ENSG00000132522 | GPS2 | 17 | 7214643 | 7218883 | 0.590137502 | 1 | 12.62 | 0 | NA | NA | NA | 3.24E-07 | rs61759532 |
| ENSG00000261915 | RP11-542C16.2 | 17 | 7215980 | 7222493 | NA | 1 | 12.62 | 0 | NA | NA | NA | 3.24E-07 | rs61759532 |
| ENSG00000215041 | NEURL4 | 17 | 7218947 | 7232712 | 0.999976734 | 3 | 20.3 | 0 | NA | NA | NA | 4.52E-09 | rs61759532 |
| ENSG00000072818 | ACAP1 | 17 | 7239848 | 7254797 | 0.237755775 | 2 | 20.3 | 3 | 3.27170000000001e-310 | 0 | eQTLcatalogue/BLUEPRINT_ge_neutrophil:eQTLcatalogue/BLUEPRINT_ge_T-cell:eQTLcatalogue/CEDAR_neutrophil_CD15:eQTLcatalogue/CEDAR_T-cell_CD4:eQTLcatalogue/Fairfax_2012_B-cell_CD19:eQTLcatalogue/Fairfax_2014_LPS24:eQTLcatalogue/Fairfax_2014_naive:eQTLcatalogue/GENCORD_ge_T-cell:eQTLcatalogue/GEUVADIS_ge_LCL:eQTLcatalogue/HipSci_ge_iPSC:eQTLcatalogue/Lepik_2017_ge_blood:eQTLcatalogue/Naranbhai_2015_neutrophil_CD16:eQTLGen_cis_eQTLs:BIOSQTL/BIOS_eQTL_geneLevel:GTEx/v8/Whole_Blood:GTEx/v8/Spleen | 4.52E-09 | rs61759532 |
| ENSG00000213859 | KCTD11 | 17 | 7255208 | 7258258 | 0.447451322 | 0 | 0 | 4 | 2.49E-140 | 0 | eQTLcatalogue/BLUEPRINT_ge_T-cell:eQTLcatalogue/CEDAR_T-cell_CD4:eQTLcatalogue/CEDAR_T-cell_CD8:eQTLcatalogue/GENCORD_ge_T-cell:eQTLcatalogue/Lepik_2017_ge_blood:DICE/T_CD4_naive_activated:DICE/T_CD8_naive_activated:eQTLGen_cis_eQTLs:eQTLGen_trans_eQTLs:BIOSQTL/BIOS_eQTL_geneLevel | 2.93E-13 | rs61759532;rs11611029 |
| ENSG00000187838 | TMEM256-PLSCR3 | 17 | 7293046 | 7307416 | 0.245307294 | 0 | 0 | 2 | 1.18E-09 | 1.31E-05 | eQTLGen_cis_eQTLs | 4.52E-09 | rs61759532 |
| ENSG00000239697 | TNFSF12 | 17 | 7452208 | 7464925 | 0.997954015 | 0 | 0 | 3 | 2.00E-38 | 0 | eQTLGen_cis_eQTLs:BIOSQTL/BIOS_eQTL_geneLevel | 4.52E-09 | rs61759532 |
| ENSG00000248871 | TNFSF12-TNFSF13 | 17 | 7452416 | 7464918 | 0.997954015 | 0 | 0 | 3 | 5.39E-07 | 0.000442266 | BIOSQTL/BIOS_eQTL_geneLevel | 4.52E-09 | rs61759532 |
| ENSG00000141504 | SAT2 | 17 | 7529552 | 7531194 | 0.017616305 | 0 | 0 | 2 | 8.81E-08 | 0.000333438 | eQTLGen_cis_eQTLs | 4.52E-09 | rs61759532 |
| ENSG00000184060 | ADAP2 | 17 | 29233362 | 29286340 | 3.98E-05 | 0 | 0 | 1 | 5.56E-09 | 0.000119638 | eQTLGen_trans_eQTLs | 4.84E-12 | rs1559810;rs7640550 |
| ENSG00000172716 | SLFN11 | 17 | 33677324 | 33700720 | 7.42E-16 | 0 | 0 | 1 | 2.03E-24 | 0 | eQTLGen_trans_eQTLs | 4.84E-12 | rs1559810;rs7640550 |
| ENSG00000161381 | PLXDC1 | 17 | 37219556 | 37310647 | 0.000149406 | 0 | 0 | 1 | 2.22E-10 | 0 | eQTLGen_trans_eQTLs | 9.18E-11 | rs6908626 |
| ENSG00000173801 | JUP | 17 | 39775692 | 39943183 | 0.037534537 | 0 | 0 | 1 | 6.03E-10 | 2.06E-05 | eQTLGen_trans_eQTLs | 4.84E-12 | rs1559810;rs7640550 |
| ENSG00000005102 | MEOX1 | 17 | 41717756 | 41739322 | 0.065397404 | 0 | 0 | 1 | 2.55E-12 | 0 | eQTLGen_trans_eQTLs | 9.18E-11 | rs6908626 |
| ENSG00000168646 | AXIN2 | 17 | 63524681 | 63557765 | 0.119052193 | 0 | 0 | 2 | 8.20E-30 | 0 | eQTLGen_trans_eQTLs | 6.46E-17 | rs6908626;rs1561924 |
| ENSG00000154229 | PRKCA | 17 | 64298754 | 64806861 | 0.952135908 | 0 | 0 | 1 | 1.39E-07 | 0.001521197 | eQTLGen_trans_eQTLs | 9.18E-11 | rs6908626 |
| ENSG00000109066 | TMEM104 | 17 | 72772622 | 72835918 | 2.89E-05 | 0 | 0 | 1 | 6.80E-14 | 0 | eQTLGen_trans_eQTLs | 4.84E-12 | rs1559810;rs7640550 |
| ENSG00000141556 | TBCD | 17 | 80709940 | 80900724 | 6.98E-05 | 0 | 0 | 1 | 6.28E-11 | 0 | eQTLGen_trans_eQTLs | 9.18E-11 | rs6908626 |
| ENSG00000082397 | EPB41L3 | 18 | 5392383 | 5630699 | 0.029278388 | 0 | 0 | 1 | 4.45E-08 | 0.000618673 | eQTLGen_trans_eQTLs | 4.84E-12 | rs1559810;rs7640550 |
| ENSG00000101773 | RBBP8 | 18 | 20378224 | 20606451 | 0.000108379 | 0 | 0 | 1 | 8.18E-06 | 0.049310021 | eQTLGen_trans_eQTLs | 4.84E-12 | rs1559810;rs7640550 |
| ENSG00000168234 | TTC39C | 18 | 21572737 | 21715574 | 0.014646861 | 0 | 0 | 1 | 1.38E-13 | 0 | eQTLGen_trans_eQTLs | 9.18E-11 | rs6908626 |
| ENSG00000134046 | MBD2 | 18 | 51679079 | 51751158 | 0.989310018 | 0 | 0 | 1 | 1.11E-09 | 3.93E-05 | eQTLGen_trans_eQTLs | 4.84E-12 | rs1559810;rs7640550 |
| ENSG00000167670 | CHAF1A | 19 | 4402659 | 4445015 | 0.996226226 | 0 | 0 | 1 | 3.76E-06 | 0.026214953 | eQTLGen_trans_eQTLs | 4.84E-12 | rs1559810;rs7640550 |
| ENSG00000125730 | C3 | 19 | 6677715 | 6730573 | 0.999590151 | 0 | 0 | 1 | 1.98E-07 | 0.001993316 | eQTLGen_trans_eQTLs | 4.84E-12 | rs1559810;rs7640550 |
| ENSG00000130813 | C19orf66 | 19 | 10196798 | 10203928 | NA | 0 | 0 | 1 | 9.42E-05 | 2.08E-31 | GTEx/v8/Whole_Blood | NA | rs34536443 |
| ENSG00000105364 | MRPL4 | 19 | 10362577 | 10370721 | 1.13E-07 | 0 | 0 | 1 | 2.84E-08 | 0.000120919 | eQTLGen_cis_eQTLs | 2.65E-15 | rs11085727 |
| ENSG00000105376 | ICAM5 | 19 | 10400657 | 10407454 | NA | 0 | 0 | 1 | 6.65E-05 | 6.83E-11 | GTEx/v8/Thyroid | 2.65E-15 | rs11085727 |
| ENSG00000220201 | ZGLP1 | 19 | 10415479 | 10420556 | 0.437504524 | 1 | 20.8 | 2 | 1.63E-09 | 1.06E-16 | PsychENCODE_eQTLs:eQTLGen_cis_eQTLs:BIOSQTL/BIOS_eQTL_geneLevel:GTEx/v8/Thyroid | 2.65E-15 | rs34536443;rs11085727 |
| ENSG00000267673 | FDX1L | 19 | 10416103 | 10426691 | 0.003189351 | 1 | 20.8 | 1 | 5.51E-08 | 5.38E-10 | PsychENCODE_eQTLs:GTEx/v8/Thyroid | 2.65E-15 | rs34536443;rs11085727 |
| ENSG00000167807 | CTD-2369P2.10 | 19 | 10416103 | 10426685 | NA | 1 | 20.8 | 1 | 6.14E-06 | 0.004308469 | BIOSQTL/BIOS_eQTL_geneLevel | 2.65E-15 | rs34536443;rs11085727 |
| ENSG00000267303 | CTD-2369P2.12 | 19 | 10426147 | 10431354 | NA | 1 | 20.8 | 0 | NA | NA | NA | NA | rs34536443 |
| ENSG00000161847 | RAVER1 | 19 | 10426888 | 10444316 | 0.982340267 | 1 | 20.8 | 0 | NA | NA | NA | NA | rs34536443 |
| ENSG00000076662 | ICAM3 | 19 | 10444452 | 10450499 | 2.08E-22 | 0 | 0 | 1 | 5.21E-57 | 0 | eQTLcatalogue/Fairfax_2014_naive:eQTLGen_cis_eQTLs:BIOSQTL/BIOS_eQTL_geneLevel | 2.65E-15 | rs11085727 |
| ENSG00000105397 | TYK2 | 19 | 10461209 | 10491352 | 0.004314482 | 2 | 25.5 | 3 | 3.60E-164 | 0 | eQTLcatalogue/Fairfax_2014_naive:eQTLcatalogue/Lepik_2017_ge_blood:eQTLcatalogue/Quach_2016_ge_monocyte_IAV:eQTLcatalogue/TwinsUK_ge_blood:eQTLGen_cis_eQTLs:BIOSQTL/BIOS_eQTL_geneLevel:GTEx/v8/Whole_Blood | 1.62E-19 | rs34536443;rs11085727 |
| ENSG00000105401 | CDC37 | 19 | 10501810 | 10530797 | 0.962357896 | 0 | 0 | 1 | 7.44E-16 | 0 | eQTLGen_cis_eQTLs:BIOSQTL/BIOS_eQTL_geneLevel | 2.65E-15 | rs11085727 |
| ENSG00000065989 | PDE4A | 19 | 10527449 | 10580305 | 0.976417636 | 0 | 0 | 1 | 3.00E-09 | 2.60E-05 | eQTLcatalogue/Quach_2016_ge_monocyte_naive:eQTLGen_cis_eQTLs | 2.65E-15 | rs11085727 |
| ENSG00000079999 | KEAP1 | 19 | 10596796 | 10614417 | 0.251629058 | 0 | 0 | 2 | 8.99E-09 | 5.79E-05 | eQTLGen_cis_eQTLs | 1.62E-19 | rs34536443;rs11085727 |
| ENSG00000129354 | AP1M2 | 19 | 10683347 | 10697991 | 0.001345952 | 0 | 0 | 1 | 1.18E-08 | 2.10E-24 | eQTLGen_cis_eQTLs:BIOSQTL/BIOS_eQTL_geneLevel:GTEx/v8/Whole_Blood | 2.65E-15 | rs11085727 |
| ENSG00000129353 | SLC44A2 | 19 | 10713133 | 10755235 | 0.041464588 | 0 | 0 | 1 | 2.73E-15 | 0 | eQTLGen_cis_eQTLs | 2.65E-15 | rs11085727 |
| ENSG00000099203 | TMED1 | 19 | 10943114 | 10946994 | 0.000162417 | 0 | 0 | 1 | 1.73E-15 | 0 | eQTLGen_cis_eQTLs | 2.65E-15 | rs11085727 |
| ENSG00000104979 | C19orf53 | 19 | 13884982 | 13889276 | 0.236661552 | 0 | 0 | 1 | 5.44E-07 | 0.004784487 | eQTLGen_trans_eQTLs | 2.93E-13 | rs11611029 |
| ENSG00000105643 | ARRDC2 | 19 | 18111941 | 18124911 | 5.13E-05 | 0 | 0 | 1 | 3.61E-06 | 0.010440555 | eQTLGen_cis_eQTLs | 1.52E-10 | rs11666808 |
| ENSG00000096996 | IL12RB1 | 19 | 18169805 | 18209754 | 1.18E-07 | 0 | 0 | 5 | 1.69E-183 | 0 | eQTLcatalogue/BLUEPRINT_ge_neutrophil:eQTLcatalogue/GEUVADIS_ge_LCL:eQTLcatalogue/Lepik_2017_ge_blood:eQTLcatalogue/TwinsUK_ge_LCL:eQTLGen_cis_eQTLs:BIOSQTL/BIOS_eQTL_geneLevel:GTEx/v8/Whole_Blood:GTEx/v8/Spleen | 1.52E-10 | rs2271881;rs11666808;rs67482294 |
| ENSG00000099308 | MAST3 | 19 | 18208603 | 18262502 | 0.998925261 | 0 | 0 | 4 | 2.44E-81 | 0 | eQTLcatalogue/BLUEPRINT_ge_monocyte:eQTLcatalogue/BLUEPRINT_ge_neutrophil:eQTLcatalogue/Fairfax_2014_naive:eQTLcatalogue/TwinsUK_ge_LCL:eQTLGen_cis_eQTLs:BIOSQTL/BIOS_eQTL_geneLevel:GTEx/v8/Whole_Blood | 1.52E-10 | rs2271881;rs11666808 |
| ENSG00000105647 | PIK3R2 | 19 | 18263928 | 18281350 | 0.939626884 | 1 | 22.3 | 0 | NA | NA | NA | 3.19E-07 | rs2271881 |
| ENSG00000268173 | PIK3R2 | 19 | 18263968 | 18288927 | NA | 1 | 22.3 | 4 | 4.93E-34 | 0 | eQTLcatalogue/Fairfax_2014_IFN24:BIOSQTL/BIOS_eQTL_geneLevel | 1.52E-10 | rs2271881;rs11666808 |
| ENSG00000216490 | IFI30 | 19 | 18283972 | 18288927 | 2.60E-05 | 1 | 22.3 | 4 | 1.50E-38 | 0 | eQTLGen_cis_eQTLs:BIOSQTL/BIOS_eQTL_geneLevel | 1.52E-10 | rs2271881;rs11666808 |
| ENSG00000254858 | MPV17L2 | 19 | 18303992 | 18307758 | 1.67E-07 | 1 | 20.3 | 5 | 1.00E-65 | 0 | eQTLcatalogue/Alasoo_2018_ge_macrophage_Salmonella:eQTLcatalogue/BLUEPRINT_ge_T-cell:eQTLcatalogue/Fairfax_2012_B-cell_CD19:eQTLcatalogue/Fairfax_2014_LPS24:eQTLcatalogue/GEUVADIS_ge_LCL:eQTLcatalogue/Quach_2016_ge_monocyte_LPS:eQTLcatalogue/Quach_2016_ge_monocyte_naive:eQTLcatalogue/Quach_2016_ge_monocyte_R848:PsychENCODE_eQTLs:eQTLGen_cis_eQTLs:BIOSQTL/BIOS_eQTL_geneLevel:GTEx/v8/Whole_Blood:GTEx/v8/Thyroid | 1.52E-10 | rs2271881;rs11666808;rs67482294 |
| ENSG00000105649 | RAB3A | 19 | 18307594 | 18314884 | 0.870967555 | 1 | 20.3 | 5 | 1.10E-23 | 0 | BIOSQTL/BIOS_eQTL_geneLevel | 1.52E-10 | rs2271881;rs67482294;rs11666808 |
| ENSG00000105650 | PDE4C | 19 | 18318771 | 18366229 | 4.89E-05 | 0 | 0 | 1 | 0.00018998 | 0.014658931 | PsychENCODE_eQTLs | 1.52E-10 | rs11666808 |
| ENSG00000130518 | KIAA1683 | 19 | 18367908 | 18385319 | 8.28E-06 | 1 | 13.83 | 5 | 3.27170000000001e-310 | 0 | eQTLcatalogue/BLUEPRINT_ge_monocyte:eQTLcatalogue/BLUEPRINT_ge_neutrophil:eQTLcatalogue/BLUEPRINT_ge_T-cell:eQTLcatalogue/BrainSeq_ge_brain:eQTLcatalogue/CEDAR_B-cell_CD19:eQTLcatalogue/CEDAR_ileum:eQTLcatalogue/CEDAR_neutrophil_CD15:eQTLcatalogue/CEDAR_rectum:eQTLcatalogue/CEDAR_T-cell_CD4:eQTLcatalogue/CEDAR_T-cell_CD8:eQTLcatalogue/Fairfax_2012_B-cell_CD19:eQTLcatalogue/Fairfax_2014_IFN24:eQTLcatalogue/Fairfax_2014_LPS24:eQTLcatalogue/Fairfax_2014_naive:eQTLcatalogue/GENCORD_ge_T-cell:eQTLcatalogue/Kasela_2017_T-cell_CD4:eQTLcatalogue/Kasela_2017_T-cell_CD8:eQTLcatalogue/Lepik_2017_ge_blood:eQTLcatalogue/Nedelec_2016_ge_macrophage_naive:eQTLcatalogue/TwinsUK_ge_blood:eQTLcatalogue/TwinsUK_ge_fat:eQTLcatalogue/TwinsUK_ge_skin:PsychENCODE_eQTLs:DICE/T_CD4_naive:DICE/T_CD8_naive:DICE/Monocyte_non_classical:DICE/NK:DICE/T_CD4_TFH:DICE/T_CD4_TH1:DICE/T_CD4_TH17:DICE/T_CD4_TH1_17:DICE/T_CD4_TH2:DICE/T_CD4_memory_TREG:DICE/T_CD4_naive_TREG:eQTLGen_cis_eQTLs:BIOSQTL/BIOS_eQTL_geneLevel:GTEx/v8/Whole_Blood:GTEx/v8/Spleen:GTEx/v8/Thyroid | 1.52E-10 | rs11666808;rs2271881;rs67482294 |
| ENSG00000130522 | JUND | 19 | 18390563 | 18392432 | 0.560887374 | 1 | 13.83 | 2 | 1.56E-21 | 0 | eQTLcatalogue/BrainSeq_ge_brain:eQTLcatalogue/GENCORD_ge_LCL:eQTLcatalogue/TwinsUK_ge_LCL:eQTLcatalogue/TwinsUK_ge_skin:eQTLGen_cis_eQTLs:BIOSQTL/BIOS_eQTL_geneLevel | 1.52E-10 | rs11666808;rs67482294 |
| ENSG00000130520 | LSM4 | 19 | 18417040 | 18434084 | 0.667371394 | 0 | 0 | 1 | 8.93E-14 | 0 | eQTLGen_cis_eQTLs:BIOSQTL/BIOS_eQTL_geneLevel | 1.52E-10 | rs11666808 |
| ENSG00000130517 | PGPEP1 | 19 | 18451397 | 18480760 | 0.000353365 | 0 | 0 | 3 | 2.24E-10 | 0 | eQTLGen_cis_eQTLs:BIOSQTL/BIOS_eQTL_geneLevel | 1.52E-10 | rs11666808;rs2271881 |
| ENSG00000090920 | FCGBP | 19 | 40353963 | 40440533 | NA | 0 | 0 | 1 | 2.93E-09 | 9.07E-05 | eQTLGen_trans_eQTLs | 9.18E-11 | rs6908626 |
| ENSG00000213889 | PPM1N | 19 | 45992035 | 46005768 | 0.000104897 | 0 | 0 | 1 | 2.77E-07 | 0.002640946 | eQTLGen_trans_eQTLs | 4.84E-12 | rs1559810;rs7640550 |
| ENSG00000125743 | SNRPD2 | 19 | 46190712 | 46195827 | 0.747515256 | 0 | 0 | 1 | 4.63E-08 | 0.000631313 | eQTLGen_trans_eQTLs | 2.93E-13 | rs11611029 |
| ENSG00000187474 | FPR3 | 19 | 52298416 | 52329442 | 0.113913458 | 0 | 0 | 1 | 1.10E-07 | 0.00124359 | eQTLGen_trans_eQTLs | 4.84E-12 | rs1559810;rs7640550 |
| ENSG00000125898 | FAM110A | 20 | 814358 | 838106 | 0.747790603 | 0 | 0 | 1 | 4.61E-06 | 0.03068787 | eQTLGen_trans_eQTLs | 4.84E-12 | rs1559810;rs7640550 |
| ENSG00000089012 | SIRPG | 20 | 1609798 | 1638425 | 5.21E-08 | 0 | 0 | 1 | 4.94E-06 | 0.032636418 | eQTLGen_trans_eQTLs | 9.18E-11 | rs6908626 |
| ENSG00000126003 | PLAGL2 | 20 | 30780306 | 30795594 | 0.869795638 | 0 | 0 | 1 | 1.86E-15 | 0 | eQTLGen_trans_eQTLs | 4.84E-12 | rs1559810;rs7640550 |
| ENSG00000197122 | SRC | 20 | 35973088 | 36034453 | 0.988983228 | 0 | 0 | 1 | 1.24E-25 | 0 | eQTLGen_trans_eQTLs | 4.84E-12 | rs1559810;rs7640550 |
| ENSG00000204103 | MAFB | 20 | 39314488 | 39317880 | NA | 0 | 0 | 1 | 6.22E-26 | 0 | eQTLGen_trans_eQTLs | 4.84E-12 | rs1559810;rs7640550 |
| ENSG00000182463 | TSHZ2 | 20 | 51588946 | 52111869 | 0.406843773 | 0 | 0 | 1 | 6.66E-08 | 0.000853196 | eQTLGen_trans_eQTLs | 9.18E-11 | rs6908626 |
| ENSG00000124203 | ZNF831 | 20 | 57766075 | 57834168 | 7.65E-09 | 0 | 0 | 1 | 5.39E-06 | 0.034969012 | eQTLGen_trans_eQTLs | 1.72E-196 | rs2476601 |
| ENSG00000243927 | MRPS6 | 21 | 35445524 | 35515334 | 0.136243673 | 0 | 0 | 1 | 5.77E-06 | 0.037153127 | eQTLGen_trans_eQTLs | 9.18E-11 | rs6908626 |
| ENSG00000157601 | MX1 | 21 | 42792231 | 42831141 | 4.50E-09 | 0 | 0 | 2 | 3.07E-46 | 0 | eQTLGen_trans_eQTLs | 3.62E-08 | rs1990760;rs35667974 |
| ENSG00000160191 | PDE9A | 21 | 44073746 | 44195619 | 0.002017364 | 0 | 0 | 1 | 2.19E-06 | 0.016628076 | eQTLGen_trans_eQTLs | 9.18E-11 | rs6908626 |
| ENSG00000184979 | USP18 | 22 | 18632666 | 18660164 | 0.392598705 | 0 | 0 | 1 | 1.03E-08 | 0.000162443 | eQTLGen_trans_eQTLs | 4.00E-08 | rs1990760 |
| ENSG00000099917 | MED15 | 22 | 20850200 | 20941919 | 0.960808623 | 0 | 0 | 2 | 4.14E-08 | 0.000580654 | eQTLGen_trans_eQTLs | 1.72E-196 | rs2476601;rs6908626 |
| ENSG00000100292 | HMOX1 | 22 | 35776354 | 35790207 | 0.000641361 | 0 | 0 | 1 | 9.30E-40 | 0 | eQTLGen_trans_eQTLs | 4.84E-12 | rs1559810;rs7640550 |
| ENSG00000100350 | FOXRED2 | 22 | 36883237 | 36903148 | 0.006915467 | 0 | 0 | 2 | 5.72E-06 | 0.0286095 | eQTLcatalogue/Fairfax_2014_naive | 7.02E-11 | rs112578407 |
| ENSG00000133466 | C1QTNF6 | 22 | 37576207 | 37595425 | 0.023789385 | 2 | 17.88 | 4 | 3.27170000000001e-310 | 0 | eQTLcatalogue/BLUEPRINT_ge_neutrophil:eQTLcatalogue/BLUEPRINT_ge_T-cell:eQTLcatalogue/BrainSeq_ge_brain:eQTLcatalogue/CEDAR_rectum:eQTLcatalogue/Fairfax_2012_B-cell_CD19:eQTLcatalogue/Fairfax_2014_naive:eQTLcatalogue/GENCORD_ge_LCL:eQTLcatalogue/GEUVADIS_ge_LCL:eQTLcatalogue/Kasela_2017_T-cell_CD4:eQTLcatalogue/Lepik_2017_ge_blood:eQTLcatalogue/Quach_2016_ge_monocyte_IAV:eQTLcatalogue/TwinsUK_ge_blood:eQTLcatalogue/TwinsUK_ge_fat:eQTLcatalogue/TwinsUK_ge_LCL:DICE/NK:DICE/T_CD4_TFH:DICE/T_CD4_TH1_17:DICE/T_CD4_TH2:eQTLGen_cis_eQTLs:BIOSQTL/BIOS_eQTL_geneLevel:GTEx/v8/Whole_Blood:GTEx/v8/Spleen:GTEx/v8/Thyroid | 7.24E-12 | rs112578407;rs229527 |
| ENSG00000183473 | SSTR3 | 22 | 37600278 | 37608362 | 0.037224359 | 0 | 0 | 3 | 8.70E-30 | 0 | eQTLGen_cis_eQTLs:BIOSQTL/BIOS_eQTL_geneLevel | 7.24E-12 | rs112578407;rs229527 |
| ENSG00000128340 | RAC2 | 22 | 37621301 | 37640488 | 0.87309232 | 0 | 0 | 4 | 1.58E-81 | 0 | eQTLGen_cis_eQTLs:BIOSQTL/BIOS_eQTL_geneLevel:GTEx/v8/Whole_Blood | 7.24E-12 | rs112578407;rs229527 |
| ENSG00000100346 | CACNA1I | 22 | 39966758 | 40085742 | 0.999998894 | 0 | 0 | 1 | 3.41E-21 | 0 | eQTLGen_trans_eQTLs | 9.18E-11 | rs6908626 |
| ENSG00000049768 | FOXP3 | 23 | 49106897 | 49121288 | 0.948721768 | 0 | 0 | 1 | 1.17E-07 | 0.001296555 | eQTLGen_trans_eQTLs | 1.72E-196 | rs2476601 |
| ENSG00000158813 | EDA | 23 | 68835911 | 69259319 | 0.922511723 | 0 | 0 | 1 | 5.19E-08 | 0.000734988 | eQTLGen_trans_eQTLs | 9.18E-11 | rs6908626 |
| ENSG00000186462 | NAP1L2 | 23 | 72432135 | 72434684 | 0.791659154 | 0 | 0 | 1 | 3.36E-08 | 0.000434846 | eQTLGen_trans_eQTLs | 9.18E-11 | rs6908626 |

SNP: single nucleotide polymorphisms; The start and end described the base pair; chr: chromosome; CADD, Combined Annotation-Dependent depletion scores.

**Supplemental Table S7. 55 Pleiotropic Genomic Loci in Hypothyroidism and RA (discovery) Identified by FUMA Using PLACO Results.**

| **NO.** | **uniqID** | **rsID** | **CHR** | **BP** | ***P*** | **start** | **end** | **nSNPs** | **nGWASSNPs** | **nIndSigSNPs** | **IndSigSNPs** |
| --- | --- | --- | --- | --- | --- | --- | --- | --- | --- | --- | --- |
| 1 | 1:1151973:C:G | rs75972122 | 1 | 1151973 | 1.03E-08 | 1123434 | 1346703 | 43 | 29 | 2 | rs75972122;rs2649599 |
| 2 | 1:2516781:A:G | rs60733400 | 1 | 2516781 | 1.85E-10 | 2483961 | 2721149 | 158 | 92 | 1 | rs60733400 |
| 3 | 1:38635462:A:G | rs2045793 | 1 | 38635462 | 2.53E-12 | 38614867 | 38652792 | 14 | 14 | 1 | rs2045793 |
| 4 | 1:65455477:A:C | rs573741 | 1 | 65455477 | 9.80E-12 | 65219883 | 65528177 | 105 | 87 | 5 | rs573741;rs2935412;rs12143618;rs77560334;rs72922282 |
| 5 | 1:108346094:C:T | rs12563228 | 1 | 108346094 | 5.05E-10 | 108306557 | 108409665 | 128 | 105 | 1 | rs12563228 |
| 6 | 1:114377568:A:G | rs2476601 | 1 | 114377568 | 1.72E-196 | 113755018 | 114611487 | 836 | 583 | 42 | rs11811451;rs12128698;rs12061333;rs6537786;rs12063762;rs72685677;rs12029185;rs12126425;rs11102658;rs72691846;rs736202;rs2476601;rs2358816;rs11102694;rs2884603;rs3811019;rs2938319;rs12563513;rs114840167;rs6669008;rs1230666;rs1230686;rs61817618;rs55811970;rs1217401;rs7515189;rs7511816;rs11102678;rs1217422;rs1217390;rs3789599;rs36064592;rs1000528;rs12127377;rs11810241;rs4839348;rs4839349;rs2938323;rs872660;rs139269023;rs74714424;rs61819212 |
| 7 | 1:156784982:C:T | rs926103 | 1 | 156784982 | 1.27E-08 | 156745499 | 156806576 | 34 | 26 | 1 | rs926103 |
| 8 | 1:167426424:A:G | rs1214598 | 1 | 167426424 | 3.04E-12 | 167405132 | 167436320 | 32 | 25 | 2 | rs1214598;rs1723022 |
| 9 | 2:163124637:C:T | rs35667974 | 2 | 163124637 | 3.62E-08 | 162992004 | 163154363 | 7 | 6 | 2 | rs1990760;rs35667974 |
| 10 | 2:191958656:C:T | rs4274624 | 2 | 191958656 | 3.16E-33 | 191851997 | 191973563 | 66 | 53 | 10 | rs4274624;rs6752770;rs10199181;rs16833215;rs3024861;rs1517352;rs13389408;rs10168266;rs10207044;rs149218447 |
| 11 | 2:204738919:A:G | rs3087243 | 2 | 204738919 | 1.31E-50 | 204471201 | 204805387 | 184 | 139 | 10 | rs11676147;rs35988305;rs12693993;rs231779;rs12464033;rs117701653;rs3087243;rs10497873;rs58716662;rs77130284 |
| 12 | 3:12319716:A:G | rs62242118 | 3 | 12319716 | 1.09E-11 | 12273621 | 12439348 | 34 | 33 | 2 | rs62242118;rs12629337 |
| 13 | 3:105934953:G:T | rs13090803 | 3 | 105934953 | 2.82E-09 | 105916291 | 105975019 | 114 | 86 | 2 | rs13090803;rs1020364 |
| 14 | 3:108285518:A:T | rs2603121 | 3 | 108285518 | 1.36E-08 | 108195773 | 108514653 | 80 | 60 | 1 | rs2603121 |
| 15 | 3:188124354:A:C | rs1559810 | 3 | 188124354 | 8.93E-15 | 188072513 | 188135783 | 86 | 61 | 3 | rs1559810;rs7640550;rs9815073 |
| 16 | 4:26090862:A:T | rs932036 | 4 | 26090862 | 5.12E-15 | 26085480 | 26128710 | 28 | 24 | 1 | rs932036 |
| 17 | 5:102622453:G:T | rs1991797 | 5 | 102622453 | 2.70E-09 | 102595837 | 102686157 | 87 | 68 | 1 | rs1991797 |
| 18 | 5:133419283:C:T | rs244672 | 5 | 133419283 | 5.20E-12 | 133418739 | 133452495 | 28 | 22 | 1 | rs244672 |
| 19 | 6:21884440:A:G | rs952579 | 6 | 21884440 | 3.86E-11 | 21859731 | 21888181 | 6 | 6 | 1 | rs952579 |
| 20 | 6:26008260:C:T | rs9393681 | 6 | 26008260 | 1.23E-08 | 25499895 | 26176517 | 231 | 188 | 4 | rs2049967;rs1892252;rs9393681;rs72834698 |
| 21 | 6:26628773:C:T | rs62396171 | 6 | 26628773 | 6.16E-09 | 26428842 | 26669593 | 5 | 4 | 2 | rs62396171;rs1741740 |
| 22 | 6:28315657:A:C | rs72854533 | 6 | 28315657 | 1.19E-21 | 27262294 | 29611431 | 2108 | 1612 | 33 | rs2393923;rs1736894;rs57252182;rs2130357;rs1233704;rs9393925;rs6456795;rs12197514;rs72854533;rs733743;rs1016069;rs4711173;rs6456908;rs3129137;rs9468356;rs209165;rs1794588;rs4713186;rs17856167;rs112886535;rs3117343;rs12660111;rs9257158;rs116466121;rs116802478;rs9257837;rs34927823;rs1003582;rs1233387;rs378956;rs362520;rs29242;rs29232 |
| 23 | 6:33194717:C:T | rs9277946 | 6 | 33194717 | 2.49E-30 | 33173842 | 33811790 | 505 | 332 | 37 | rs383711;rs213208;rs411919;rs2854027;rs1704996;rs1704995;rs1704997;rs76766085;rs9277936;rs9277946;rs9277949;rs12190797;rs12197724;rs71565397;rs34859217;rs9296092;rs210157;rs9469527;rs78075721;rs4713624;rs2229637;rs34942621;rs4713653;rs9461896;rs34527391;rs4713669;rs12207382;rs12202730;rs28607030;rs35964955;rs1547669;rs10947440;rs10947417;rs1830873;rs13219530;rs10947433;rs12194518 |
| 24 | 6:35489775:A:G | rs749523 | 6 | 35489775 | 6.77E-09 | 35477152 | 35537964 | 15 | 15 | 1 | rs749523 |
| 25 | 6:91005743:G:T | rs6908626 | 6 | 91005743 | 3.44E-11 | 90880393 | 91005743 | 3 | 3 | 1 | rs6908626 |
| 26 | 6:135446826:C:G | rs12191243 | 6 | 135446826 | 3.05E-09 | 135406183 | 135477207 | 55 | 45 | 1 | rs12191243 |
| 27 | 6:167526096:C:T | rs968334 | 6 | 167526096 | 1.66E-19 | 167359558 | 167544278 | 479 | 351 | 7 | rs968334;rs2769343;rs3756838;rs2345568;rs415890;rs204295;rs150112 |
| 28 | 7:4785129:G:T | rs10277273 | 7 | 4785129 | 1.95E-08 | 4766383 | 4785515 | 15 | 11 | 1 | rs10277273 |
| 29 | 7:37382465:G:T | rs60600003 | 7 | 37382465 | 8.91E-13 | 37372614 | 37437919 | 65 | 60 | 2 | rs60600003;rs10279209 |
| 30 | 7:128579202:C:T | rs3807307 | 7 | 128579202 | 2.72E-15 | 128563333 | 128735438 | 136 | 106 | 6 | rs3807307;rs13246321;rs12674059;rs34644138;rs12540468;rs7801838 |
| 31 | 8:8094555:C:T | rs2945250 | 8 | 8094555 | 4.19E-09 | 8088230 | 8548117 | 103 | 67 | 2 | rs2945250;rs7826238 |
| 32 | 8:11440019:A:G | rs4841567 | 8 | 11440019 | 1.89E-09 | 10745469 | 11830150 | 522 | 415 | 6 | rs4841567;rs7004825;rs11250127;rs7836059;rs9792227;rs4841566 |
| 33 | 8:61395832:C:T | rs6992869 | 8 | 61395832 | 2.91E-10 | 61298005 | 61570627 | 183 | 123 | 1 | rs6992869 |
| 34 | 8:129569371:A:G | rs1561924 | 8 | 129569371 | 1.42E-17 | 129311446 | 129771358 | 268 | 233 | 19 | rs13281279;rs6470633;rs77156523;rs10095762;rs117697016;rs11785816;rs12682121;rs62528379;rs16903004;rs1561924;rs1476163;rs7825794;rs16903111;rs1516982;rs1561927;rs11780763;rs75725733;rs17805996;rs12543298 |
| 35 | 9:100522530:G:T | rs1512261 | 9 | 100522530 | 1.96E-09 | 100522530 | 100530899 | 5 | 5 | 2 | rs1512261;rs7357631 |
| 36 | 9:127086129:C:T | rs867610 | 9 | 127086129 | 1.49E-11 | 126999153 | 127184067 | 158 | 126 | 3 | rs867610;rs34071757;rs2026191 |
| 37 | 10:6098949:C:T | rs706778 | 10 | 6098949 | 1.33E-20 | 6071453 | 6182251 | 81 | 66 | 10 | rs706778;rs3118469;rs10905716;rs35285258;rs4747846;rs11256442;rs7090504;rs1924138;rs706779;rs41260244 |
| 38 | 10:63779871:C:T | rs71508903 | 10 | 63779871 | 3.71E-22 | 63779871 | 64060240 | 106 | 85 | 6 | rs71508903;rs68156080;rs10821945;rs10821948;rs10761603;rs10761620 |
| 39 | 11:64107735:A:G | rs663743 | 11 | 64107735 | 3.47E-09 | 64012910 | 64141771 | 47 | 33 | 1 | rs663743 |
| 40 | 11:95311422:C:T | rs4409785 | 11 | 95311422 | 5.72E-10 | 95298828 | 95320808 | 4 | 4 | 1 | rs4409785 |
| 41 | 12:55368291:C:T | rs62623446 | 12 | 55368291 | 4.02E-08 | 55358844 | 55368291 | 2 | 2 | 1 | rs62623446 |
| 42 | 12:56477694:A:T | rs2271194 | 12 | 56477694 | 2.46E-14 | 56354272 | 56609885 | 60 | 36 | 4 | rs2271194;rs7960225;rs10876863;rs11611029 |
| 43 | 12:111884608:C:T | rs3184504 | 12 | 111884608 | 8.04E-57 | 111359712 | 113235274 | 356 | 257 | 20 | rs3184504;rs10744774;rs4766897;rs11066152;rs7300285;rs11065785;rs11065822;rs4766453;rs2339718;rs1265565;rs3809272;rs7398796;rs117532831;rs11066320;rs233724;rs233722;rs55847700;rs232924;rs2891403;rs976702 |
| 44 | 13:24775072:G:T | rs1923908 | 13 | 24775072 | 3.03E-08 | 24772780 | 24788031 | 36 | 28 | 1 | rs1923908 |
| 45 | 14:68743307:A:G | rs10131490 | 14 | 68743307 | 3.79E-12 | 68727506 | 68815261 | 77 | 63 | 1 | rs10131490 |
| 46 | 14:106127786:A:G | rs12895622 | 14 | 106127786 | 6.93E-13 | 106073059 | 106201807 | 158 | 25 | 1 | rs12895622 |
| 47 | 15:38847359:C:T | rs6495979 | 15 | 38847359 | 1.67E-11 | 38820606 | 38925195 | 62 | 52 | 2 | rs6495979;rs56083426 |
| 48 | 17:7240391:C:T | rs61759532 | 17 | 7240391 | 4.52E-09 | 7226957 | 7240391 | 6 | 6 | 1 | rs61759532 |
| 49 | 18:12775821:A:C | rs2847259 | 18 | 12775821 | 7.51E-11 | 12775821 | 12886441 | 23 | 18 | 2 | rs2847259;rs12967678 |
| 50 | 19:4838056:A:G | rs59129932 | 19 | 4838056 | 8.15E-10 | 4833410 | 4838056 | 15 | 8 | 1 | rs59129932 |
| 51 | 19:10463118:C:G | rs34536443 | 19 | 10463118 | 1.62E-19 | 10427721 | 10586018 | 10 | 8 | 3 | rs34536443;rs11085727;rs539820608 |
| 52 | 19:18383506:C:T | rs11666808 | 19 | 18383506 | 2.82E-11 | 18282940 | 18408519 | 60 | 45 | 3 | rs11666808;rs67482294;rs2271881 |
| 53 | 19:55763262:A:G | rs73068668 | 19 | 55763262 | 2.37E-08 | 55739048 | 55763262 | 2 | 2 | 1 | rs73068668 |
| 54 | 22:37581485:A:C | rs229527 | 22 | 37581485 | 7.24E-12 | 37573712 | 37609342 | 29 | 25 | 2 | rs229527;rs112578407 |
| 55 | 22:39747780:A:G | rs2069235 | 22 | 39747780 | 1.49E-08 | 39739187 | 39756650 | 11 | 11 | 1 | rs2069235 |

No.: the numerical order of pleiotropic genomic risk locus; CHR: chromosome; BP: base pair SNP: single nucleotide polymorphisms.

**Supplemental Table S8. 66 Pleiotropic Genomic Loci in Hypothyroidism and RA (replication) Identified by FUMA Using PLACO Results.**

| **NO.** | **uniqID** | **rsID** | **CHR** | **BP** | ***P*** | **start** | **end** | **nSNPs** | **nGWASSNPs** | **nIndSigSNPs** | **IndSigSNPs** |
| --- | --- | --- | --- | --- | --- | --- | --- | --- | --- | --- | --- |
| 1 | 1:2516781:A:G | rs60733400 | 1 | 2516781 | 2.31E-13 | 2483961 | 2721576 | 218 | 125 | 4 | rs28734787;rs61765774;rs60733400;rs881640 |
| 2 | 1:38635462:A:G | rs2045793 | 1 | 38635462 | 5.19E-12 | 38226776 | 38652792 | 110 | 78 | 7 | rs36084352;rs28469609;rs12022363;rs28411352;rs3748682;rs2127647;rs2045793 |
| 3 | 1:67798445:C:T | rs72678531 | 1 | 67798445 | 1.52E-10 | 67787691 | 67825399 | 18 | 16 | 1 | rs72678531 |
| 4 | 1:114377568:A:G | rs2476601 | 1 | 114377568 | 6.38E-261 | 113810083 | 114647963 | 867 | 617 | 47 | rs11811451;rs773566;rs6680300;rs4839318;rs12061333;rs6537786;rs12063762;rs773557;rs72685677;rs72685682;rs12128454;rs72691846;rs736202;rs4839324;rs2797414;rs1237682;rs2476601;rs11102694;rs1217392;rs3811019;rs2938319;rs10494164;rs72687906;rs6669008;rs1230666;rs115699904;rs61817618;rs55811970;rs1217401;rs10858006;rs7511816;rs10858015;rs12144309;rs1217422;rs1217390;rs66782936;rs3789599;rs36064592;rs1217441;rs7545300;rs11810241;rs4839349;rs4348719;rs139269023;rs74714424;rs61819212;rs34817006 |
| 5 | 1:117262791:C:T | rs958434 | 1 | 117262791 | 5.72E-09 | 117257144 | 117287101 | 12 | 9 | 3 | rs958434;rs624988;rs12137270 |
| 6 | 1:157668390:C:T | rs7522061 | 1 | 157668390 | 3.10E-09 | 157615218 | 157802877 | 92 | 78 | 1 | rs7522061 |
| 7 | 2:98373006:C:T | rs5865 | 2 | 98373006 | 5.72E-11 | 98013814 | 98373006 | 191 | 13 | 2 | rs5865;rs11123861 |
| 8 | 2:100811903:A:C | rs7580200 | 2 | 100811903 | 5.60E-11 | 100640986 | 100871361 | 203 | 166 | 3 | rs7580200;rs4851253;rs11691869 |
| 9 | 2:191966452:C:G | rs7568275 | 2 | 191966452 | 4.23E-33 | 191520845 | 191994936 | 172 | 140 | 16 | rs1155060;rs11894064;rs2356121;rs11687659;rs1033249;rs34997637;rs10199181;rs16833177;rs7568275;rs6752770;rs4341966;rs16833215;rs13017460;rs13426947;rs13389408;rs10207044 |
| 10 | 2:204738919:A:G | rs3087243 | 2 | 204738919 | 4.98E-64 | 204570092 | 204812044 | 274 | 213 | 14 | rs35988305;rs12693993;rs58038423;rs2882970;rs117701653;rs13429019;rs231839;rs12464033;rs3087243;rs231724;rs10497873;rs79570815;rs138585195;rs77130284 |
| 11 | 3:12316326:A:G | rs62242116 | 3 | 12316326 | 1.44E-09 | 12026709 | 12439348 | 66 | 55 | 2 | rs62242116;rs310746 |
| 12 | 3:58318477:A:G | rs185407974 | 3 | 58318477 | 3.02E-10 | 57931931 | 58502708 | 85 | 69 | 3 | rs185407974;rs4681852;rs114584537 |
| 13 | 3:105934953:G:T | rs13090803 | 3 | 105934953 | 4.92E-15 | 105916291 | 105975019 | 115 | 89 | 2 | rs13090803;rs1373740 |
| 14 | 4:10716939:C:G | rs4293777 | 4 | 10716939 | 6.95E-15 | 10701970 | 10814525 | 104 | 85 | 3 | rs4293777;rs55688771;rs56001653 |
| 15 | 4:26090375:A:G | rs7441808 | 4 | 26090375 | 1.68E-25 | 25995272 | 26128710 | 94 | 82 | 5 | rs7441808;rs1877386;rs10939090;rs4692059;rs56147366 |
| 16 | 4:38677227:A:C | rs34089598 | 4 | 38677227 | 3.88E-08 | 38613107 | 38703970 | 23 | 14 | 1 | rs34089598 |
| 17 | 4:80905833:C:T | rs28534734 | 4 | 80905833 | 4.62E-09 | 80805741 | 80967671 | 88 | 63 | 1 | rs28534734 |
| 18 | 4:123554790:C:T | rs10518402 | 4 | 123554790 | 3.84E-09 | 123023206 | 123555178 | 37 | 31 | 1 | rs10518402 |
| 19 | 4:149665602:C:T | rs11732089 | 4 | 149665602 | 1.73E-10 | 149568408 | 149728841 | 128 | 102 | 1 | rs11732089 |
| 20 | 5:102595892:C:G | rs28158 | 5 | 102595892 | 1.94E-16 | 102191715 | 102686157 | 559 | 423 | 4 | rs28158;rs168820;rs454275;rs26431 |
| 21 | 5:133422816:A:G | rs244689 | 5 | 133422816 | 3.39E-13 | 133418739 | 133514972 | 48 | 38 | 2 | rs244689;rs244660 |
| 22 | 6:412802:A:G | rs9392504 | 6 | 412802 | 2.42E-13 | 382559 | 450033 | 52 | 43 | 7 | rs9392504;rs9378807;rs6916402;rs6930468;rs56306240;rs9392525;rs2671427 |
| 23 | 6:29557642:A:C | rs115503606 | 6 | 29557642 | 8.01E-25 | 25935591 | 29611788 | 2285 | 1569 | 45 | rs35173303;rs67575965;rs77377107;rs116756717;rs2232430;rs3116836;rs276362;rs2394102;rs6901724;rs9468413;rs79141865;rs3135329;rs6933672;rs9468487;rs116508100;rs148466862;rs238883;rs55969931;rs73396583;rs6905368;rs9468358;rs13215804;rs61289879;rs73407126;rs1233478;rs59068970;rs73407222;rs3129137;rs3129146;rs9257802;rs3130725;rs2745401;rs12527959;rs1233400;rs3130248;rs1233372;rs12524535;rs28986304;rs28749531;rs148589791;rs115503606;rs17351888;rs362520;rs79208750;rs29232 |
| 24 | 6:33182895:A:C | rs1704996 | 6 | 33182895 | 2.48E-87 | 33173842 | 33860194 | 999 | 742 | 54 | rs421446;rs213210;rs1705001;rs9277946;rs213197;rs1704996;rs1704995;rs1704998;rs2282850;rs9277976;rs11756767;rs62405860;rs113451798;rs112528998;rs139333827;rs115516845;rs2854027;rs34859217;rs211468;rs114542428;rs145325967;rs73408233;rs11967839;rs75254945;rs9277949;rs12197724;rs28892580;rs120626;rs1014779;rs211453;rs3130276;rs9461856;rs12190797;rs2274195;rs34942621;rs210181;rs6911745;rs210194;rs210132;rs62407633;rs2229637;rs4713653;rs9368772;rs4713669;rs12207382;rs9380376;rs35964955;rs1547669;rs13214793;rs1535950;rs1830873;rs7761859;rs78861422;rs4713711 |
| 25 | 6:91005743:G:T | rs6908626 | 6 | 91005743 | 3.11E-18 | 90809560 | 91037287 | 109 | 92 | 5 | rs6908626;rs1504215;rs927297;rs72928086;rs755178 |
| 26 | 6:138003822:C:G | rs11757201 | 6 | 138003822 | 1.84E-13 | 137959235 | 138262773 | 53 | 48 | 4 | rs11757201;rs694069;rs12525643;rs17781283 |
| 27 | 6:159489791:A:G | rs212389 | 6 | 159489791 | 2.96E-10 | 159465977 | 159515309 | 43 | 39 | 3 | rs212389;rs212405;rs212392 |
| 28 | 6:167541258:C:G | rs3093017 | 6 | 167541258 | 6.62E-26 | 167359558 | 167547442 | 472 | 358 | 10 | rs3093017;rs9366093;rs2769343;rs2236312;rs2236313;rs2757053;rs239936;rs204295;rs6941355;rs6905876 |
| 29 | 7:37382465:G:T | rs60600003 | 7 | 37382465 | 2.10E-09 | 37372614 | 37437919 | 64 | 59 | 1 | rs60600003 |
| 30 | 7:128579202:C:T | rs3807307 | 7 | 128579202 | 1.17E-15 | 128563333 | 128735438 | 111 | 91 | 4 | rs3807307;rs17424602;rs34591253;rs7801838 |
| 31 | 8:8164318:A:G | rs60672704 | 8 | 8164318 | 3.16E-08 | 8160717 | 8181518 | 5 | 3 | 1 | rs60672704 |
| 32 | 8:11080014:C:T | rs6601571 | 8 | 11080014 | 4.55E-08 | 10758213 | 11450422 | 167 | 135 | 1 | rs6601571 |
| 33 | 8:129569371:A:G | rs1561924 | 8 | 129569371 | 6.95E-14 | 129492753 | 129592699 | 118 | 102 | 6 | rs1561924;rs7825794;rs77156523;rs1356332;rs10089519;rs1516982 |
| 34 | 8:134212652:C:T | rs13250295 | 8 | 134212652 | 3.51E-09 | 134191256 | 134219818 | 18 | 16 | 2 | rs13250295;rs3739262 |
| 35 | 9:5438435:A:C | rs911760 | 9 | 5438435 | 7.68E-09 | 5438435 | 5438435 | 1 | 1 | 1 | rs911760 |
| 36 | 9:100615914:A:G | rs1867277 | 9 | 100615914 | 7.00E-19 | 100533317 | 100670272 | 232 | 194 | 5 | rs1867277;rs4743140;rs723227;rs4273946;rs10759960 |
| 37 | 9:123691237:G:T | rs1930785 | 9 | 123691237 | 2.01E-09 | 123636121 | 123723351 | 134 | 106 | 1 | rs1930785 |
| 38 | 10:6098949:C:T | rs706778 | 10 | 6098949 | 7.84E-23 | 6074451 | 6542361 | 108 | 86 | 18 | rs7893467;rs706778;rs3118469;rs11597367;rs7090530;rs10905716;rs7916878;rs1983890;rs11593331;rs4747846;rs11256442;rs11256516;rs2387397;rs2181622;rs10906340;rs72781765;rs4750528;rs622998 |
| 39 | 10:9049253:C:T | rs12413578 | 10 | 9049253 | 1.95E-08 | 9043457 | 9049253 | 3 | 3 | 1 | rs12413578 |
| 40 | 10:63779871:C:T | rs71508903 | 10 | 63779871 | 6.08E-32 | 63742015 | 64057202 | 109 | 89 | 9 | rs71508903;rs68156080;rs12761779;rs10821945;rs76027217;rs3740355;rs10761604;rs75180516;rs4529854 |
| 41 | 10:124195485:A:G | rs7072204 | 10 | 124195485 | 1.88E-09 | 124119446 | 124198585 | 42 | 30 | 1 | rs7072204 |
| 42 | 11:35266944:A:G | rs736374 | 11 | 35266944 | 4.56E-09 | 35244058 | 35329809 | 67 | 53 | 1 | rs736374 |
| 43 | 11:61581656:A:G | rs174559 | 11 | 61581656 | 1.20E-10 | 61542006 | 61623140 | 64 | 52 | 2 | rs174559;rs968567 |
| 44 | 11:64107477:C:T | rs479777 | 11 | 64107477 | 1.07E-09 | 64012910 | 64141771 | 45 | 37 | 1 | rs479777 |
| 45 | 11:95311422:C:T | rs4409785 | 11 | 95311422 | 9.49E-20 | 95298828 | 95320808 | 4 | 4 | 1 | rs4409785 |
| 46 | 12:56384804:A:G | rs705699 | 12 | 56384804 | 1.91E-15 | 56368078 | 56609885 | 73 | 52 | 4 | rs705699;rs11611029;rs705696;rs7960225 |
| 47 | 12:103923154:A:T | rs117818633 | 12 | 103923154 | 3.37E-08 | 103923154 | 103926966 | 2 | 2 | 1 | rs117818633 |
| 48 | 12:111884608:C:T | rs3184504 | 12 | 111884608 | 5.01E-44 | 111426615 | 113218868 | 553 | 414 | 28 | rs2301658;rs6490048;rs2879564;rs7299183;rs6490061;rs7398796;rs11065884;rs648997;rs695871;rs10744775;rs1265564;rs7963641;rs3184504;rs4766897;rs4767078;rs7300285;rs11065822;rs61507607;rs3809272;rs11066320;rs7974266;rs233701;rs11066353;rs233724;rs233721;rs494273;rs2891403;rs232935 |
| 49 | 13:24782080:C:T | rs7329958 | 13 | 24782080 | 4.43E-13 | 24773157 | 24788197 | 33 | 23 | 1 | rs7329958 |
| 50 | 13:42999179:C:T | rs4566053 | 13 | 42999179 | 3.48E-12 | 42945821 | 43096532 | 180 | 135 | 2 | rs4566053;rs1853573 |
| 51 | 14:68749927:A:G | rs3784099 | 14 | 68749927 | 2.23E-13 | 68727506 | 68815261 | 82 | 67 | 1 | rs3784099 |
| 52 | 14:98641284:G:T | rs76381532 | 14 | 98641284 | 8.08E-09 | 98531606 | 98668778 | 124 | 105 | 1 | rs76381532 |
| 53 | 15:38846738:C:G | rs4924273 | 15 | 38846738 | 6.38E-22 | 38817150 | 38925195 | 66 | 56 | 5 | rs4924273;rs16967103;rs10520096;rs4583194;rs11855910 |
| 54 | 15:69990787:A:G | rs919053 | 15 | 69990787 | 8.12E-13 | 69972912 | 70048984 | 53 | 48 | 3 | rs919053;rs17374222;rs11857740 |
| 55 | 16:11826013:C:T | rs11075010 | 16 | 11826013 | 1.78E-08 | 11764082 | 11841539 | 86 | 69 | 1 | rs11075010 |
| 56 | 17:7226957:A:G | rs35776863 | 17 | 7226957 | 1.23E-08 | 7226957 | 7240391 | 6 | 6 | 1 | rs35776863 |
| 57 | 17:40289412:C:T | rs12325861 | 17 | 40289412 | 1.47E-09 | 40271970 | 40300126 | 46 | 38 | 1 | rs12325861 |
| 58 | 18:12797694:A:G | rs2847297 | 18 | 12797694 | 3.55E-15 | 12745889 | 12924171 | 162 | 126 | 10 | rs2847297;rs80262450;rs2847281;rs8091566;rs2222138;rs12966224;rs2847262;rs7237497;rs34452117;rs62099360 |
| 59 | 19:4837487:A:G | rs10425559 | 19 | 4837487 | 1.98E-11 | 4802438 | 4837557 | 24 | 16 | 1 | rs10425559 |
| 60 | 19:10463118:C:G | rs34536443 | 19 | 10463118 | 2.43E-22 | 10423338 | 10591276 | 67 | 49 | 5 | rs34536443;rs11085727;rs12720270;rs74179925;rs280519 |
| 61 | 19:50196992:A:G | rs12980063 | 19 | 50196992 | 2.25E-08 | 50189156 | 50207165 | 9 | 9 | 1 | rs12980063 |
| 62 | 21:35911003:A:G | rs74679034 | 21 | 35911003 | 8.83E-09 | 35906671 | 35938968 | 156 | 137 | 1 | rs74679034 |
| 63 | 21:43855067:A:C | rs1893592 | 21 | 43855067 | 5.64E-11 | 43820573 | 43855067 | 29 | 28 | 4 | rs80054410;rs12482396;rs12482947;rs1893592 |
| 64 | 22:30545149:A:G | rs4823077 | 22 | 30545149 | 3.40E-11 | 30223888 | 30592487 | 40 | 24 | 1 | rs4823077 |
| 65 | 22:37593091:A:G | rs6000603 | 22 | 37593091 | 3.41E-11 | 37573712 | 37609342 | 29 | 26 | 2 | rs6000603;rs229540 |
| 66 | 22:39747671:A:T | rs909685 | 22 | 39747671 | 7.25E-13 | 39659487 | 39756650 | 65 | 49 | 3 | rs909685;rs5757600;rs137688 |

No.: the numerical order of pleiotropic genomic risk locus; CHR: chromosome; BP: base pair SNP: single nucleotide polymorphisms.

**Supplemental Table S9. Thirteen Colocalized Loci in Hypothyroidism and RA (replication) Identified through Colocalization Analysis on Pleiotropic Loci.**

| **Top SNV** | **Locus boundary^a^** | **Region** | **Nearest gene** | **Best causal** | **SNP.PP.H4** |
| --- | --- | --- | --- | --- | --- |
| rs5865 | 2:98013814-98373006 | 2q11.2 | *TMEM131, ZAP70* | rs5865 | 1.0 |
| rs7568275 | 2:191520845-191994936 | 2q32.2-q32.3 | *STAT4* | rs13389408 | 1.0 |
| rs185407974 | 3:57931931-58502708 | 3p14.3 | *PXK* | rs35677470 | 0.825 |
| rs11732089 | 4:149568408-149728841 | 4q31.23 | *LOC107986195* | rs72735938 | 0.992 |
| rs115503606 | 6:25935591-29611788 | 6p22.2-p22.1 | *OR2H2* | rs4713242 | 0.811 |
| rs1704996 | 6:33173842-33860194 | 6p21.32-p21.31 | / | rs3128947 | 1.0 |
| rs6908626 | 6:90809560-91037287 | 6q15 | *BACH2, LOC105377891, LOC124901361* | rs6454802 | 0.875 |
| rs3093017 | 6:167359558-167547442 | 6q27 | *CCR6, LOC107986672* | rs11575078 | 0.999 |
| rs12413578 | 10:9043457-9049253 | 10p14 | / | rs12413578 | 0.842 |
| rs4409785 | 11:95298828-95320808 | 11q21 | / | rs34536806 | 0.960 |
| rs7329958 | 13:24773157-24788197 | 13q12.12 | *SPATA13* | rs1220603 | 0.782 |
| rs34536443 | 19:10423338-10591276 | 19p13.2 | *TYK2* | rs34536443 | 0.957 |
| rs6000603 | 22:37573712-37609342 | 22q12.3-q13.1 | *C1QTNF6* | rs229544 | 0.807 |

^a^ Locus boundary of each pleiotropic genomic risk locus was denoted as “chromosome: start-end” defined by FUMA.

**Supplemental Table S10. 39 single nucleotide variants between hypothyroidism and RA (discovery) identified by MTAG**

| **SNP** | **CHR** | **BP** | **A1** | **A2** | **Z** | **FRQ** | **mtag_beta_**  **hypothyroidism** | **mtag_se_**  **hypothyroidism** | **mtag_z_**  **hypothyroidism** | **mtag_pval_**  **hypothyroidism** | **mtag_beta_**  **RA (discovery)** | **mtag_se_**  **RA (discovery)** | **mtag_z_**  **RA (discovery)** | **mtag_pval_**  **RA (discovery)** |
| --- | --- | --- | --- | --- | --- | --- | --- | --- | --- | --- | --- | --- | --- | --- |
| rs2045793 | 1 | 38635462 | A | G | -5.867733465 | 0.260524 | -0.015011467 | 0.002425143 | -6.189931694 | 6.02E-10 | -0.019676184 | 0.003374624 | -5.830630019 | 5.52E-09 |
| rs573741 | 1 | 65455477 | C | A | 5.05444857 | 0.89527 | 0.018992531 | 0.003476247 | 5.463516004 | 4.67E-08 | 0.028565192 | 0.00458168 | 6.234654663 | 4.53E-10 |
| rs12086096 | 1 | 113856853 | G | A | 8.254740934 | 0.220118 | 0.021629065 | 0.0025691 | 8.418927824 | 3.80E-17 | 0.020652373 | 0.003728992 | 5.538326412 | 3.05E-08 |
| rs1237290 | 1 | 114135147 | C | A | -7.016030911 | 0.338601 | -0.016532536 | 0.0022493 | -7.350081795 | 1.98E-13 | -0.020235132 | 0.003113195 | -6.499796021 | 8.04E-11 |
| rs1217403 | 1 | 114388804 | T | C | -8.244506907 | 0.253612 | -0.020768598 | 0.00244656 | -8.488896349 | 2.09E-17 | -0.021521941 | 0.003431163 | -6.272492441 | 3.55E-10 |
| rs34382796 | 1 | 114564892 | G | T | 5.356505997 | 0.086191 | 0.021569916 | 0.00379284 | 5.687009495 | 1.29E-08 | 0.031519059 | 0.005570816 | 5.657888647 | 1.53E-08 |
| rs16833215 | 2 | 191913799 | A | G | 7.578818168 | 0.291161 | 0.01825288 | 0.002343057 | 7.790200138 | 6.69E-15 | 0.017686792 | 0.003133906 | 5.643689815 | 1.66E-08 |
| rs117701653 | 2 | 204628795 | A | C | -5.647275073 | 0.045318 | -0.030623935 | 0.005117507 | -5.984150977 | 2.18E-09 | -0.035948675 | 0.00613626 | -5.858401745 | 4.67E-09 |
| rs7441808 | 4 | 26090375 | A | G | 8.115827012 | 0.30075 | 0.019242166 | 0.002321155 | 8.289910365 | 1.13E-16 | 0.017858328 | 0.003210892 | 5.561796478 | 2.67E-08 |
| rs3117343 | 6 | 29205333 | C | T | -5.917867221 | 0.550994 | -0.013827978 | 0.002140049 | -6.461524587 | 1.04E-10 | -0.023443542 | 0.002969014 | -7.896070083 | 2.88E-15 |
| rs29232 | 6 | 29611431 | C | T | -6.865546455 | 0.352325 | -0.015919295 | 0.002228296 | -7.144156436 | 9.06E-13 | -0.019418378 | 0.003282645 | -5.915467084 | 3.31E-09 |
| rs9260313 | 6 | 29916885 | T | C | -7.712759898 | 0.282455 | -0.019072535 | 0.002364416 | -8.066487107 | 7.23E-16 | -0.022782197 | 0.003244924 | -7.020872996 | 2.20E-12 |
| rs3130396 | 6 | 30223490 | T | C | 7.538241582 | 0.433903 | 0.017568736 | 0.002147739 | 8.180108939 | 2.84E-16 | 0.02794669 | 0.002913751 | 9.591308858 | 8.70E-22 |
| rs41315800 | 6 | 30706631 | T | C | -7.430545748 | 0.062339 | -0.033782707 | 0.004402713 | -7.673157381 | 1.68E-14 | -0.055821232 | 0.009527099 | -5.859205823 | 4.65E-09 |
| rs2844670 | 6 | 31005726 | G | A | 7.196925695 | 0.832928 | 0.023176021 | 0.002853433 | 8.122154884 | 4.58E-16 | 0.047979285 | 0.003986191 | 12.03637368 | 2.29E-33 |
| rs28752924 | 6 | 31303922 | T | C | -12.58975909 | 0.445799 | -0.026873821 | 0.002141509 | -12.549009 | 4.03E-36 | -0.016671142 | 0.00289257 | -5.763435568 | 8.24E-09 |
| rs2857283 | 6 | 31381162 | C | T | -8.051796104 | 0.129249 | -0.027448996 | 0.003172945 | -8.650951474 | 5.11E-18 | -0.05144192 | 0.005444691 | -9.448088506 | 3.45E-21 |
| rs115128935 | 6 | 31571971 | T | C | -7.551315171 | 0.017116 | -0.064817489 | 0.008206744 | -7.898075711 | 2.83E-15 | -0.083853995 | 0.012191724 | -6.877943974 | 6.07E-12 |
| rs28732147 | 6 | 31572634 | G | A | -6.316117403 | 0.050101 | -0.032221135 | 0.004879342 | -6.60358189 | 4.01E-11 | -0.067657613 | 0.011809343 | -5.729159771 | 1.01E-08 |
| rs35609644 | 6 | 31638848 | C | T | -6.700379549 | 0.015468 | -0.060127457 | 0.008625637 | -6.970784436 | 3.15E-12 | -0.056745446 | 0.009852821 | -5.759309596 | 8.45E-09 |
| rs28366162 | 6 | 31695590 | T | C | -7.364950174 | 0.074681 | -0.031139054 | 0.004049229 | -7.69011854 | 1.47E-14 | -0.100072545 | 0.015189957 | -6.588072982 | 4.46E-11 |
| rs143928576 | 6 | 31871281 | C | T | -8.191171925 | 0.017636 | -0.070839389 | 0.00808699 | -8.759672922 | 1.96E-18 | -0.077415057 | 0.008384118 | -9.233536282 | 2.62E-20 |
| rs116793716 | 6 | 31892310 | T | G | -7.337164972 | 0.016499 | -0.066134031 | 0.008356164 | -7.914400576 | 2.48E-15 | -0.091027155 | 0.010230522 | -8.897606297 | 5.71E-19 |
| rs140776878 | 6 | 32134828 | C | T | -8.230613652 | 0.016776 | -0.074311728 | 0.008288057 | -8.966121596 | 3.07E-19 | -0.094940716 | 0.008797385 | -10.79192482 | 3.76E-27 |
| rs6906662 | 6 | 32266506 | G | A | -7.93038869 | 0.061225 | -0.041158218 | 0.004439949 | -9.269974361 | 1.86E-20 | -0.088932097 | 0.005485307 | -16.21278245 | 4.10E-59 |
| rs28366319 | 6 | 32561495 | G | A | 8.285304799 | 0.349149 | 0.024222056 | 0.00223294 | 10.84760871 | 2.05E-27 | 0.093153888 | 0.003368482 | 27.65456374 | 2.46E-168 |
| rs17211881 | 6 | 32637197 | T | G | 7.663600256 | 0.089831 | 0.031807214 | 0.003722623 | 8.544302001 | 1.29E-17 | 0.054692967 | 0.004614053 | 11.85356235 | 2.06E-32 |
| rs2856997 | 6 | 32781776 | C | A | -7.554593835 | 0.388477 | -0.013288879 | 0.002183907 | -6.084910704 | 1.17E-09 | 0.028555274 | 0.002895773 | 9.861019847 | 6.14E-23 |
| rs13213265 | 6 | 33039755 | T | C | -11.73244941 | 0.1778 | -0.034374577 | 0.002783996 | -12.34720882 | 5.04E-35 | -0.052680869 | 0.004626547 | -11.38664998 | 4.87E-30 |
| rs926419 | 6 | 33111408 | G | A | 7.779775878 | 0.364222 | 0.019003753 | 0.002212011 | 8.591164501 | 8.61E-18 | 0.033382442 | 0.002961691 | 11.27141363 | 1.82E-29 |
| rs2229637 | 6 | 33643558 | G | A | -4.791781662 | 0.290989 | -0.012888806 | 0.002343465 | -5.499893359 | 3.80E-08 | -0.034072575 | 0.003844534 | -8.862601307 | 7.82E-19 |
| rs7774396 | 6 | 167519786 | G | A | 7.613348879 | 0.453213 | 0.016709552 | 0.002138272 | 7.814512494 | 5.52E-15 | 0.016312349 | 0.002930527 | 5.566354024 | 2.60E-08 |
| rs60600003 | 7 | 37382465 | T | G | 5.998657747 | 0.100194 | 0.022425201 | 0.003545095 | 6.325698184 | 2.52E-10 | 0.028249159 | 0.004756473 | 5.939097425 | 2.87E-09 |
| rs3807307 | 7 | 128579202 | T | C | 5.930949612 | 0.464579 | 0.013639466 | 0.002134252 | 6.390747952 | 1.65E-10 | 0.020821063 | 0.002920371 | 7.129594465 | 1.01E-12 |
| rs1561924 | 8 | 129569371 | G | A | -7.079931245 | 0.128538 | -0.023780031 | 0.003180411 | -7.4770319 | 7.60E-14 | -0.032197691 | 0.004527216 | -7.112029219 | 1.14E-12 |
| rs10986319 | 9 | 127093823 | C | T | -5.6597095 | 0.626215 | -0.013111857 | 0.002200141 | -5.959552971 | 2.53E-09 | -0.016473532 | 0.002982623 | -5.523169657 | 3.33E-08 |
| rs3118470 | 10 | 6101713 | T | C | 7.560887317 | 0.317566 | 0.018110276 | 0.002286524 | 7.920438114 | 2.37E-15 | 0.020382514 | 0.002911585 | 7.000488065 | 2.55E-12 |
| rs10821948 | 10 | 63809624 | C | A | 8.077892991 | 0.324524 | 0.018976238 | 0.002273499 | 8.34670963 | 7.02E-17 | 0.01876191 | 0.002924068 | 6.41637248 | 1.40E-10 |
| rs11085727 | 19 | 10466123 | C | T | -7.05564624 | 0.292428 | -0.017228114 | 0.002340068 | -7.362229313 | 1.81E-13 | -0.020556646 | 0.003280682 | -6.265966539 | 3.71E-10 |

CHR：chromosome; BP: base pair SNP: single nucleotide polymorphisms; RA: rheumatoid arthritis.**Supplemental Table S11. 53 single nucleotide variants between hypothyroidism and RA (replication) identified by MTAG.**

| **SNP** | **CHR** | **BP** | **A1** | **A2** | **Z** | **FRQ** | **mtag_beta_** | **mtag_se_** | **mtag_z_** | **mtag_pval_** | **mtag_beta_** | **mtag_se_** | **mtag_z_** | **mtag_pval_** |
| --- | --- | --- | --- | --- | --- | --- | --- | --- | --- | --- | --- | --- | --- | --- |
|  |  |  |  |  |  |  | **hypothyroidism** | **hypothyroidism** | **hypothyroidism** | **hypothyroidism** | **RA (replicatiom)** | **RA (replicatiom)** | **RA (replicatiom)** | **RA (replicatiom)** |
| rs1061801 | 6 | 33282338 | G | A | 8.030399291 | 0.190763 | 0.023591398 | 0.002676527 | 8.814181777 | 1.21E-18 | 0.06312138 | 0.007636705 | 8.26552591 | 1.39E-16 |
| rs10821948 | 10 | 63809624 | C | A | 8.077892991 | 0.324524 | 0.019186604 | 0.002246096 | 8.542202874 | 1.32E-17 | 0.041048783 | 0.006407687 | 6.406177454 | 1.49E-10 |
| rs11085727 | 19 | 10466123 | C | T | -7.05564624 | 0.292428 | -0.017866347 | 0.002311861 | -7.72812188 | 1.09E-14 | -0.047147588 | 0.006578395 | -7.167034825 | 7.66E-13 |
| rs115503606 | 6 | 29557642 | C | A | 7.174402097 | 0.046191 | 0.041357727 | 0.00501011 | 8.254854837 | 1.52E-16 | 0.135618348 | 0.014093075 | 9.623048921 | 6.39E-22 |
| rs116282089 | 6 | 29823616 | A | G | 7.864513282 | 0.041569 | 0.047792948 | 0.005268553 | 9.071360521 | 1.18E-19 | 0.158029218 | 0.014795409 | 10.68096279 | 1.25E-26 |
| rs12086096 | 1 | 113856853 | G | A | 8.254740934 | 0.220118 | 0.023241845 | 0.002538133 | 9.157063287 | 5.33E-20 | 0.065536078 | 0.007229222 | 9.065439742 | 1.24E-19 |
| rs1217403 | 1 | 114388804 | T | C | -8.244506907 | 0.253612 | -0.021856538 | 0.002417071 | -9.04257227 | 1.53E-19 | -0.058164759 | 0.006885882 | -8.446959037 | 2.99E-17 |
| rs1237290 | 1 | 114135147 | C | A | -7.016030911 | 0.338601 | -0.01685453 | 0.002222188 | -7.584656641 | 3.33E-14 | -0.04136471 | 0.006327208 | -6.537593116 | 6.25E-11 |
| rs1266079 | 6 | 31504774 | A | G | -6.551993243 | 0.2242 | -0.023144309 | 0.002521529 | -9.178681457 | 4.36E-20 | -0.132859852 | 0.007203511 | -18.44376342 | 5.85E-76 |
| rs13200022 | 6 | 31098957 | C | T | -8.017165213 | 0.152798 | -0.026717776 | 0.002922835 | -9.141048638 | 6.18E-20 | -0.08582934 | 0.008363882 | -10.26190224 | 1.05E-24 |
| rs13389408 | 2 | 191933283 | T | C | 8.113807803 | 0.117537 | 0.028660695 | 0.003265285 | 8.777395362 | 1.67E-18 | 0.070665375 | 0.00930324 | 7.595781419 | 3.06E-14 |
| rs140545087 | 6 | 32960494 | G | A | -7.692093006 | 0.019577 | -0.065488662 | 0.007590612 | -8.627586564 | 6.27E-18 | -0.192620742 | 0.021390561 | -9.004940965 | 2.16E-19 |
| rs14597 | 6 | 31440014 | C | A | 7.108164053 | 0.433212 | 0.018475864 | 0.002122248 | 8.705800334 | 3.15E-18 | 0.076318877 | 0.006038876 | 12.63792702 | 1.30E-36 |
| rs148466862 | 6 | 29317177 | C | T | -4.86850309 | 0.045478 | -0.02822722 | 0.005047345 | -5.592489132 | 2.24E-08 | -0.092768792 | 0.014325104 | -6.475959596 | 9.42E-11 |
| rs1561924 | 8 | 129569371 | G | A | -7.079931245 | 0.128538 | -0.023555877 | 0.003142075 | -7.496916483 | 6.53E-14 | -0.050740427 | 0.008942873 | -5.673839274 | 1.40E-08 |
| rs16901846 | 6 | 28254988 | T | C | -4.794016233 | 0.096137 | -0.020476867 | 0.003567467 | -5.739888902 | 9.47E-09 | -0.078988618 | 0.010194073 | -7.748484623 | 9.30E-15 |
| rs17179234 | 6 | 29821340 | C | A | -5.239732751 | 0.076954 | -0.023623293 | 0.003945748 | -5.987025287 | 2.14E-09 | -0.076502793 | 0.011280347 | -6.781953619 | 1.19E-11 |
| rs17404424 | 6 | 29275298 | G | A | 5.820303786 | 0.041765 | 0.035850065 | 0.005256714 | 6.819862073 | 9.11E-12 | 0.126504617 | 0.014828551 | 8.531151824 | 1.45E-17 |
| rs2045793 | 1 | 38635462 | A | G | -5.867733465 | 0.260524 | -0.015257129 | 0.002395911 | -6.367986275 | 1.92E-10 | -0.038254846 | 0.006815422 | -5.612982456 | 1.99E-08 |
| rs2069235 | 22 | 39747780 | G | A | 5.119526878 | 0.301712 | 0.013423971 | 0.002291095 | 5.859195195 | 4.65E-09 | 0.043635911 | 0.006529961 | 6.682415235 | 2.35E-11 |
| rs2073045 | 6 | 32339548 | G | A | 8.285363628 | 0.372703 | 0.027233852 | 0.002174896 | 12.52190838 | 5.67E-36 | 0.177186234 | 0.00617157 | 28.71007386 | 2.86E-181 |
| rs2394102 | 6 | 28512017 | A | G | -5.438038341 | 0.118136 | -0.020479191 | 0.003258102 | -6.285619927 | 3.27E-10 | -0.069451788 | 0.009306677 | -7.462576272 | 8.48E-14 |
| rs2621417 | 6 | 32853607 | G | A | 7.905832046 | 0.579799 | 0.019038193 | 0.002130538 | 8.935860657 | 4.04E-19 | 0.058592919 | 0.006066334 | 9.658702713 | 4.52E-22 |
| rs28157 | 5 | 102595837 | G | T | -6.913979823 | 0.315166 | -0.017012705 | 0.002263572 | -7.51586735 | 5.65E-14 | -0.043077345 | 0.006441924 | -6.687030795 | 2.28E-11 |
| rs2847297 | 18 | 12797694 | A | G | 6.986720859 | 0.344087 | 0.016590334 | 0.002213601 | 7.494726183 | 6.64E-14 | 0.038844524 | 0.006298408 | 6.167356442 | 6.94E-10 |
| rs2857698 | 6 | 31585084 | C | T | 7.768128999 | 0.286267 | 0.02506918 | 0.0023265 | 10.77549074 | 4.49E-27 | 0.141026461 | 0.006640311 | 21.23793025 | 4.26E-100 |
| rs28894081 | 6 | 30482476 | G | A | -7.061685179 | 0.066686 | -0.032922277 | 0.00421527 | -7.810241798 | 5.71E-15 | -0.091624295 | 0.012027788 | -7.617717991 | 2.58E-14 |
| rs3095351 | 6 | 30806518 | C | T | 8.196103908 | 0.583377 | 0.020229785 | 0.002133096 | 9.483768037 | 2.45E-21 | 0.068701326 | 0.006075711 | 11.30753626 | 1.20E-29 |
| rs3118470 | 10 | 6101713 | T | C | 7.560887317 | 0.317566 | 0.018446216 | 0.002258964 | 8.165786086 | 3.19E-16 | 0.698959759 | 0.09986757 | 6.99886619 | 2.58E-12 |
| rs3130396 | 6 | 30223490 | T | C | 7.538241582 | 0.433903 | 0.020031464 | 0.002121851 | 9.440561499 | 3.71E-21 | 0.088318892 | 0.006037939 | 14.62732354 | 1.88E-48 |
| rs34382796 | 1 | 114564892 | G | T | 5.356505997 | 0.086191 | 0.022285329 | 0.003747123 | 5.947317331 | 2.73E-09 | 0.06290272 | 0.010636712 | 5.913737241 | 3.34E-09 |
| rs35677470 | 3 | 58183636 | G | A | 5.345132831 | 0.080833 | 0.022763139 | 0.003858025 | 5.900204892 | 3.63E-09 | 0.062126072 | 0.010902856 | 5.698146525 | 1.21E-08 |
| rs36101795 | 6 | 33690691 | C | T | -5.260038205 | 0.019236 | -0.046121923 | 0.007656265 | -6.024075912 | 1.70E-09 | -0.149215568 | 0.021657539 | -6.889774768 | 5.59E-12 |
| rs3784099 | 14 | 68749927 | G | A | -6.547066874 | 0.28185 | -0.016416833 | 0.002337437 | -7.023431832 | 2.16E-12 | -0.038533479 | 0.006665322 | -5.781187511 | 7.42E-09 |
| rs3807307 | 7 | 128579202 | T | C | 5.930949612 | 0.464579 | 0.014104445 | 0.002108527 | 6.689241551 | 2.24E-11 | 0.043006217 | 0.006005645 | 7.160965014 | 8.01E-13 |
| rs3823403 | 6 | 31081746 | C | T | 8.052527762 | 0.189926 | 0.023324235 | 0.002681032 | 8.699721467 | 3.33E-18 | 0.056919526 | 0.007618321 | 7.471400255 | 7.93E-14 |
| rs3873230 | 6 | 29718052 | G | A | -5.187301101 | 0.017244 | -0.047491996 | 0.008078203 | -5.879029989 | 4.13E-09 | -0.148979319 | 0.02316589 | -6.430977464 | 1.27E-10 |
| rs56082284 | 6 | 29984592 | C | T | -5.128103837 | 0.016684 | -0.047162187 | 0.008210318 | -5.744258445 | 9.23E-09 | -0.140507799 | 0.023578655 | -5.959109958 | 2.54E-09 |
| rs60733400 | 1 | 2516781 | G | A | -5.542547986 | 0.340825 | -0.013804708 | 0.002218659 | -6.222095959 | 4.91E-10 | -0.04119937 | 0.006318178 | -6.520767965 | 6.99E-11 |
| rs614549 | 6 | 31840625 | A | G | 8.102321238 | 0.343033 | 0.026344996 | 0.00221522 | 11.89272185 | 1.29E-32 | 0.164390269 | 0.006322674 | 26.00011901 | 4.94E-149 |
| rs62407567 | 6 | 33537213 | A | G | 7.739578797 | 0.268409 | 0.019678574 | 0.002373143 | 8.292199148 | 1.11E-16 | 0.045735364 | 0.00675329 | 6.772308777 | 1.27E-11 |
| rs6456151 | 6 | 167471851 | A | C | 10.03193017 | 0.408866 | 0.021931712 | 0.002139061 | 10.25296314 | 1.15E-24 | 0.03570328 | 0.006090401 | 5.862221887 | 4.57E-09 |
| rs6495979 | 15 | 38847359 | C | T | 8.282842393 | 0.257035 | 0.021522722 | 0.002406447 | 8.94377557 | 3.76E-19 | 0.052548243 | 0.00686277 | 7.657002276 | 1.90E-14 |
| rs6831973 | 4 | 10709366 | T | C | 7.393175716 | 0.544519 | 0.01636261 | 0.002111616 | 7.748856759 | 9.27E-15 | 0.032803413 | 0.006013112 | 5.455313695 | 4.89E-08 |
| rs6906128 | 6 | 32206304 | A | C | -7.722489862 | 0.264519 | -0.026778602 | 0.002384199 | -11.23169882 | 2.85E-29 | -0.164700875 | 0.006813739 | -24.17187856 | 4.40E-129 |
| rs6933672 | 6 | 28941688 | G | A | -4.484536301 | 0.109336 | -0.018525885 | 0.003369908 | -5.497444929 | 3.85E-08 | -0.07714838 | 0.009640573 | -8.002468274 | 1.22E-15 |
| rs7441808 | 4 | 26090375 | A | G | 8.115827012 | 0.30075 | 0.020645783 | 0.002293177 | 9.003135898 | 2.19E-19 | 0.058185342 | 0.006527491 | 8.913890785 | 4.93E-19 |
| rs76573413 | 6 | 29638474 | T | C | -6.57214848 | 0.026862 | -0.04823347 | 0.006504295 | -7.415633976 | 1.21E-13 | -0.148302492 | 0.018644868 | -7.954065226 | 1.80E-15 |
| rs9271776 | 6 | 32594341 | T | C | 8.274286083 | 0.357769 | 0.029138445 | 0.002193864 | 13.28179416 | 2.95E-40 | 0.20708379 | 0.006229171 | 33.24419468 | 2.48E-242 |
| rs9277000 | 6 | 32989436 | C | T | 8.230345897 | 0.134437 | 0.032015689 | 0.003082818 | 10.38520309 | 2.90E-25 | 0.144018289 | 0.008766194 | 16.42882721 | 1.19E-60 |
| rs9392504 | 6 | 412802 | G | A | 7.080211636 | 0.545074 | 0.015761361 | 0.002111828 | 7.463374233 | 8.43E-14 | 0.032921363 | 0.00601322 | 5.474831103 | 4.38E-08 |
| rs9469237 | 6 | 32689067 | T | C | 5.913673498 | 0.265009 | 0.017361149 | 0.002382787 | 7.286067214 | 3.19E-13 | 0.0738089 | 0.006854007 | 10.76872288 | 4.84E-27 |
| rs947474 | 10 | 6390450 | G | A | 6.373515068 | 0.815687 | 0.018953142 | 0.00271217 | 6.988184511 | 2.78E-12 | 0.050352853 | 0.007726892 | 6.516572391 | 7.19E-11 |

CHR: chromosome; BP: base pair SNP: single nucleotide polymorphisms; RA: rheumatoid arthritis.

**Supplemental Table S12. MR analysis of hypothyroidism and RA (discovery).**

| **exposure** | **outcome** | **method** | **nSNP** | **β** | **SE** | **pval** | **lo_ci** | **up_ci** | **or** | **or_lci95** | **or_uci95** |
| --- | --- | --- | --- | --- | --- | --- | --- | --- | --- | --- | --- |
| hypothyroidism | RA | MR Egger | 121 | 1.361669 | 3.4672936 | 6.95E-01 | -5.4342262 | 8.157565 | 3.902703 | 0.004364611 | 3489.6782 |
| hypothyroidism | RA | Weighted median | 121 | 4.108512 | 0.6892062 | 2.50E-09 | 2.7576677 | 5.459356 | 60.856085 | 15.76303534 | 234.9461 |
| hypothyroidism | RA | Inverse variance weighted | 121 | 4.030483 | 1.0280261 | 8.83E-05 | 2.0155517 | 6.045414 | 56.288088 | 7.504866562 | 422.1726 |
| hypothyroidism | RA | Simple mode | 121 | 3.164074 | 1.864089 | 9.22E-02 | -0.4895403 | 6.817689 | 23.666823 | 0.612908067 | 913.8703 |
| hypothyroidism | RA | Weighted mode | 121 | 3.164074 | 1.5323615 | 4.11E-02 | 0.1606456 | 6.167503 | 23.666823 | 1.17426879 | 476.9934 |
|  | | | | | | | | | | | |
| **exposure** | **outcome** | **method** | **nSNP** | **β** | **SE** | **pval** | **lo_ci** | **up_ci** | **or** | **or_lci95** | **or_uci95** |
| RA | hypothyroidism | MR Egger | 21 | -0.003408275 | 0.00878929 | 0.7024919 | -0.020635283 | 0.013818732 | 0.9965975 | 0.9795762 | 1.013915 |
| RA | hypothyroidism | Weighted median | 21 | -0.002339873 | 0.001840798 | 0.2036863 | -0.005947836 | 0.00126809 | 0.9976629 | 0.9940698 | 1.001269 |
| RA | hypothyroidism | Inverse variance weighted | 21 | 0.010413687 | 0.0057156 | 0.06845858 | -0.000788888 | 0.021616263 | 1.0104681 | 0.9992114 | 1.021852 |
| RA | hypothyroidism | Simple mode | 21 | 0.006058073 | 0.003859822 | 0.132213 | -0.001507177 | 0.013623323 | 1.0060765 | 0.998494 | 1.013717 |
| RA | hypothyroidism | Weighted mode | 21 | -0.005324003 | 0.000947949 | 1.70E-05 | -0.007181983 | -0.003466023 | 0.9946901 | 0.9928437 | 0.996540 |

SE: standard error; SNP: single nucleotide polymorphisms; RA: rheumatoid arthritis.

**Supplemental Table S13. MR analysis of the causal effect of hypothyroidism on RA (replication).**

| **exposure** | **outcome** | **method** | **nSNP** | **β** | **SE** | **pval** |
| --- | --- | --- | --- | --- | --- | --- |
| hypothyroidism | RA | MR Egger | 92 | 11.583737 | 3.6844812 | 2.26E-03 |
| hypothyroidism | RA | Weighted median | 92 | 9.694234 | 1.1068956 | 1.99E-18 |
| hypothyroidism | RA | Inverse variance weighted | 92 | 11.294389 | 0.9645791 | 1.14E-31 |
| hypothyroidism | RA | Simple mode | 92 | 7.927740 | 3.7513568 | 3.73E-02 |
| hypothyroidism | RA | Weighted mode | 92 | 6.666100 | 2.7481470 | 1.73E-03 |

SE: standard error; SNP: single nucleotide polymorphisms; RA: rheumatoid arthritis.

**Supplemental Table S14. the top ten noteworthy GO or KEGG pathways**

| **subgroup** | **GOterm** | ***P*-value** | **Count** |
| --- | --- | --- | --- |
| Biological process | type I interferon signaling pathway | 1.58E-12 | 18 |
| Biological process | cellular response to type I interferon | 1.58E-12 | 18 |
| Biological process | response to type I interferon | 3.30E-12 | 18 |
| Biological process | defense response to virus | 6.80E-11 | 26 |
| Biological process | T cell activation | 9.25E-11 | 36 |
| Biological process | response to virus | 1.21E-10 | 30 |
| Biological process | regulation of T cell activation | 7.89E-10 | 28 |
| Biological process | positive regulation of T cell activation | 1.40E-09 | 22 |
| Biological process | positive regulation of leukocyte cell-cell adhesion | 1.55E-09 | 23 |
| Biological process | positive regulation of cell-cell adhesion | 1.89E-09 | 25 |
| Cellular component | external side of plasma membrane | 7.12E-05 | 22 |
| Cellular component | lateral plasma membrane | 2.32E-03 | 6 |
| Cellular component | clathrin adaptor complex | 1.47E-02 | 3 |
| Cellular component | endocytic vesicle | 1.48E-02 | 13 |
| Cellular component | ribosomal subunit | 1.69E-02 | 9 |
| Cellular component | cytoplasmic side of membrane | 1.74E-02 | 9 |
| Cellular component | unconventional myosin complex | 1.75E-02 | 2 |
| Cellular component | endocytic vesicle membrane | 2.20E-02 | 8 |
| Cellular component | podosome | 2.20E-02 | 3 |
| Cellular component | melanosome | 2.27E-02 | 6 |
| Molecular function | ubiquitin-like protein ligase binding | 2.25E-04 | 18 |
| Molecular function | adenylyltransferase activity | 5.23E-04 | 5 |
| Molecular function | ubiquitin protein ligase binding | 9.03E-04 | 16 |
| Molecular function | tumor necrosis factor-activated receptor activity | 1.12E-03 | 3 |
| Molecular function | cytokine receptor activity | 1.31E-03 | 8 |
| Molecular function | 3',5'-cyclic-nucleotide phosphodiesterase activity | 1.44E-03 | 4 |
| Molecular function | GTP-dependent protein binding | 1.44E-03 | 4 |
| Molecular function | sodium:phosphate symporter activity | 1.51E-03 | 3 |
| Molecular function | hormone receptor binding | 1.85E-03 | 11 |
| Molecular function | 3',5'-cyclic-AMP phosphodiesterase activity | 1.98E-03 | 3 |
| KEGG | Measles | 1.39E-09 | 19 |
| KEGG | Hepatitis C | 3.48E-07 | 17 |
| KEGG | Coronavirus disease - COVID-19 | 5.12E-06 | 19 |
| KEGG | Th17 cell differentiation | 1.50E-05 | 12 |
| KEGG | PD-L1 expression and PD-1 checkpoint pathway in cancer | 7.09E-05 | 10 |
| KEGG | Influenza A | 9.37E-05 | 14 |
| KEGG | Epstein-Barr virus infection | 1.59E-04 | 15 |
| KEGG | Hepatocellular carcinoma | 2.92E-04 | 13 |
| KEGG | Th1 and Th2 cell differentiation | 4.69E-04 | 9 |
| KEGG | EGFR tyrosine kinase inhibitor resistance | 7.68E-04 | 8 |

**Supplemental Table S15. Candidate genes in blood associated with hypothyroidism using TWAS.**

| **ID** | **CHR** | **Start** | **End** | **HSQ** | **BEST.GWAS.ID** | **BEST.GWAS.Z** | **EQTL.ID** | **EQTL.R2** | **EQTL.Z** | **EQTL.GWAS.Z** | **NSNP** | **NWGT** | **MODEL** | **MODELCV.R2** | **MODELCV.PV** | **TWAS.Z** | **TWAS.P** | **padj** |
| --- | --- | --- | --- | --- | --- | --- | --- | --- | --- | --- | --- | --- | --- | --- | --- | --- | --- | --- |
| ENSG00000240409.1 | 1 | 633534 | 633535 | -0.00804 | rs1891910 | -3.342 | rs1891910 | 0.0117 | 1.66 | -3.34194 | 80 | 1 | top1 | 0.012 | 0.006 | -3.34 | 8.32E-04 | 2.88E-02 |
| ENSG00000228327.3 | 1 | 778625 | 778626 | 0.025298 | rs6697886 | 5.169 | rs1891910 | 0.00506 | 3.91 | -3.34194 | 137 | 1 | lasso | 0.0058 | 0.04 | -3.34 | 8.32E-04 | 2.88E-02 |
| ENSG00000188157.14 | 1 | 1020122 | 1020123 | 0.049343 | rs6697886 | 5.169 | rs1320565 | 0.00302 | -3.67 | 3.45294 | 187 | 1 | top1 | 0.003 | 0.1 | -3.45 | 5.55E-04 | 2.13E-02 |
| ENSG00000186891.13 | 1 | 1206690 | 1206691 | 0.1756 | rs6697886 | 5.169 | rs12036216 | 0.111 | -7.94 | 3.51304 | 228 | 228 | susie | 0.11 | 3.30E-16 | -3.72 | 1.98E-04 | 9.97E-03 |
| ENSG00000078808.16 | 1 | 1232030 | 1232031 | 0.052847 | rs6697886 | 5.169 | rs7515488 | 0.0389 | -5.28 | 4.96505 | 229 | 1 | top1 | 0.039 | 1.60E-06 | -4.97 | 6.87E-07 | 1.02E-04 |
| ENSG00000176022.4 | 1 | 1232264 | 1232265 | 0.110002 | rs6697886 | 5.169 | rs12036216 | 0.0961 | -7.49 | 3.51304 | 229 | 1 | top1 | 0.096 | 4.10E-14 | -3.51 | 4.43E-04 | 1.79E-02 |
| ENSG00000240731.1 | 1 | 1318677 | 1318678 | 0.01656 | rs6697886 | 5.169 | rs11721 | 0.00888 | 2.63 | 3.2007 | 253 | 1 | top1 | 0.0089 | 0.015 | 3.2 | 1.37E-03 | 3.96E-02 |
| ENSG00000224870.6 | 1 | 1399521 | 1399522 | 0.013977 | rs6697886 | 5.169 | rs12093154 | -0.0018 | 3.39 | 4.78545 | 275 | 275 | susie | -0.0012 | 0.56 | 5.73 | 1.00E-08 | 2.73E-06 |
| ENSG00000242485.5 | 1 | 1407312 | 1407313 | 0.080426 | rs6697886 | 5.169 | rs1240708 | 0.0365 | 5.13 | 4.60364 | 276 | 1 | top1 | 0.036 | 3.30E-06 | 4.6 | 4.15E-06 | 4.29E-04 |
| ENSG00000272455.1 | 1 | 1409095 | 1409096 | 0.052035 | rs6697886 | 5.169 | rs12089560 | 0.0249 | -4.51 | 3.23412 | 276 | 1 | top1 | 0.025 | 0.00011 | -3.23 | 1.22E-03 | 3.69E-02 |
| ENSG00000160072.19 | 1 | 1471768 | 1471769 | 0.257556 | rs6697886 | 5.169 | rs2031709 | 0.079 | -7.09 | 1.61575 | 285 | 59 | enet | 0.098 | 1.90E-14 | -3.27 | 1.08E-03 | 3.38E-02 |
| ENSG00000197785.13 | 1 | 1512150 | 1512151 | 0.088923 | rs6697886 | 5.169 | rs3128342 | 0.00344 | -4.14 | 0.99708 | 279 | 279 | susie | 0.022 | 0.00028 | -3.87 | 1.08E-04 | 6.22E-03 |
| ENSG00000157881.13 | 1 | 2526627 | 2526628 | 0.111004 | rs2234167 | -6.545 | rs4648559 | 0.0126 | 5.33 | -1.00523 | 357 | 13 | enet | 0.05 | 6.30E-08 | -3.19 | 1.43E-03 | 4.08E-02 |
| ENSG00000157873.17 | 1 | 2556696 | 2556697 | 0.040055 | rs2234167 | -6.545 | rs1886730 | -0.000858 | 4.03 | 1.78683 | 364 | 26 | enet | 0.011 | 0.0074 | -3.39 | 6.96E-04 | 2.49E-02 |
| ENSG00000157870.14 | 1 | 2586490 | 2586491 | 0.133851 | rs2234167 | -6.545 | rs4445406 | 0.105 | -9.05 | 5.29999 | 366 | 24 | enet | 0.16 | 8.60E-24 | -4.28 | 1.89E-05 | 1.55E-03 |
| ENSG00000142606.15 | 1 | 2632989 | 2632990 | 0.163611 | rs2234167 | -6.545 | rs3748816 | 0.126 | -9.37 | 5.47822 | 356 | 32 | enet | 0.19 | 4.80E-27 | -4.24 | 2.28E-05 | 1.78E-03 |
| ENSG00000215912.12 | 1 | 2801716 | 2801717 | 0.067302 | rs2234167 | -6.545 | rs4648356 | 0.0699 | 6.88 | 5.02104 | 369 | 369 | susie | 0.073 | 5.40E-11 | 5.33 | 9.97E-08 | 2.09E-05 |
| ENSG00000232912.5 | 1 | 8424644 | 8424645 | 0.034095 | rs301805 | -4.913 | rs301805 | 0.0273 | -4.42 | -4.91289 | 303 | 1 | top1 | 0.027 | 5.20E-05 | 4.91 | 8.97E-07 | 1.22E-04 |
| ENSG00000142599.17 | 1 | 8817642 | 8817643 | 0.227115 | rs301805 | -4.913 | rs301806 | 0.24 | -11.95 | -4.76595 | 374 | 18 | enet | 0.24 | 8.60E-36 | 4.68 | 2.88E-06 | 3.13E-04 |
| ENSG00000233929.1 | 1 | 16241397 | 16241398 | 0.080659 | rs6698317 | -3.459 | rs6698317 | 0.0653 | 6.14 | -3.45868 | 316 | 1 | top1 | 0.065 | 5.40E-10 | -3.46 | 5.43E-04 | 2.11E-02 |
| ENSG00000127481.14 | 1 | 19210275 | 19210276 | 0.030591 | rs2473808 | -6.589 | rs4912151 | -0.00169 | -3.02 | 0.55658 | 510 | 510 | susie | 0.0019 | 0.15 | -3.21 | 1.31E-03 | 3.87E-02 |
| ENSG00000053371.12 | 1 | 19312145 | 19312146 | 0.145277 | rs2473808 | -6.589 | rs859218 | 0.0453 | -5.49 | -6.26854 | 487 | 487 | susie | 0.086 | 8.70E-13 | 3.62 | 2.94E-04 | 1.32E-02 |
| ENSG00000040487.12 | 1 | 19312325 | 19312326 | 0.158795 | rs2473808 | -6.589 | rs214336 | 0.132 | -9.38 | -5.65383 | 487 | 4 | lasso | 0.16 | 1.60E-22 | 6.35 | 2.12E-10 | 8.95E-08 |
| ENSG00000011007.12 | 1 | 23743154 | 23743155 | 0.142485 | rs586173 | -5.07 | rs2999566 | 0.125 | -8.59 | -5.03528 | 342 | 1 | top1 | 0.12 | 4.80E-18 | 5.04 | 4.77E-07 | 7.61E-05 |
| ENSG00000236810.5 | 1 | 23778286 | 23778287 | 0.045858 | rs586173 | -5.07 | rs2999566 | 0.0273 | -4.77 | -5.03528 | 354 | 354 | susie | 0.032 | 1.30E-05 | 4.2 | 2.68E-05 | 1.99E-03 |
| ENSG00000011009.10 | 1 | 23790969 | 23790970 | 0.022238 | rs586173 | -5.07 | rs10917430 | -0.00161 | -3.15 | -4.88087 | 359 | 3 | lasso | 0.0054 | 0.046 | 4.81 | 1.47E-06 | 1.83E-04 |
| ENSG00000117308.14 | 1 | 23800803 | 23800804 | 0.009395 | rs586173 | -5.07 | rs7514394 | -0.000644 | -3.06 | -4.08821 | 360 | 360 | susie | 0.0058 | 0.04 | 4.14 | 3.41E-05 | 2.43E-03 |
| ENSG00000117305.14 | 1 | 23838619 | 23838620 | 0.04995 | rs586173 | -5.07 | rs6424115 | 0.0351 | -5 | -4.13485 | 366 | 366 | susie | 0.037 | 2.50E-06 | 4.01 | 6.11E-05 | 3.97E-03 |
| ENSG00000117713.18 | 1 | 26696031 | 26696032 | 0.082789 | rs17261915 | -3.408 | rs17162311 | 0.000715 | -3.84 | -2.05268 | 322 | 56 | enet | 0.013 | 0.004 | 3.25 | 1.17E-03 | 3.61E-02 |
| ENSG00000126698.10 | 1 | 28233024 | 28233025 | 0.169317 | rs905908 | 4.569 | rs490633 | 0.149 | -9.3 | 3.98889 | 253 | 1 | top1 | 0.15 | 1.60E-21 | -3.99 | 6.64E-05 | 4.25E-03 |
| ENSG00000162522.10 | 1 | 32741884 | 32741885 | 0.132249 | rs16835227 | 3.151 | rs2184931 | 0.0721 | -6.59 | -3.10943 | 238 | 6 | lasso | 0.073 | 5.10E-11 | 3.15 | 1.61E-03 | 4.41E-02 |
| ENSG00000204084.12 | 1 | 37947056 | 37947057 | 0.495929 | rs36084352 | 6.236 | rs36084352 | 0.417 | -15.31 | 6.23591 | 459 | 8 | lasso | 0.46 | 8.20E-76 | -6.79 | 1.11E-11 | 6.17E-09 |
| ENSG00000183431.11 | 1 | 37990920 | 37990921 | 0.589154 | rs36084352 | 6.236 | rs4634868 | 0.249 | 12.2 | -3.09108 | 468 | 21 | enet | 0.47 | 7.70E-79 | -5.3 | 1.16E-07 | 2.31E-05 |
| ENSG00000183386.9 | 1 | 38005605 | 38005606 | 0.597659 | rs36084352 | 6.236 | rs4634868 | 0.436 | 15.63 | -3.09108 | 464 | 7 | lasso | 0.5 | 3.30E-86 | -3.86 | 1.13E-04 | 6.37E-03 |
| ENSG00000183520.11 | 1 | 38009257 | 38009258 | 0.067334 | rs36084352 | 6.236 | rs12138115 | 0.0424 | 5.83 | -4.77104 | 462 | 1 | top1 | 0.042 | 5.50E-07 | -4.77 | 1.83E-06 | 2.20E-04 |
| ENSG00000243970.1 | 1 | 39558706 | 39558707 | 0.065945 | rs7542907 | -3.725 | rs11206378 | 0.02 | 4.54 | 3.1798 | 381 | 1 | top1 | 0.02 | 0.00047 | 3.18 | 1.47E-03 | 4.13E-02 |
| ENSG00000186409.14 | 1 | 42463329 | 42463330 | 0.08163 | rs11590814 | -4.548 | rs1002701 | 0.0155 | 5.17 | -3.28154 | 340 | 340 | susie | 0.033 | 9.80E-06 | -3.15 | 1.65E-03 | 4.47E-02 |
| ENSG00000065978.18 | 1 | 42682426 | 42682427 | 0.00626 | rs11590814 | -4.548 | rs11581921 | 0.00926 | -2.62 | -4.41914 | 375 | 1 | top1 | 0.0093 | 0.013 | 4.42 | 9.91E-06 | 9.08E-04 |
| ENSG00000117385.15 | 1 | 42767083 | 42767084 | 0.075177 | rs11590814 | -4.548 | rs10890213 | 0.0399 | 5.29 | -3.32984 | 415 | 1 | top1 | 0.04 | 1.20E-06 | -3.33 | 8.69E-04 | 2.94E-02 |
| ENSG00000198198.15 | 1 | 43389881 | 43389882 | 0.136841 | rs11210860 | -3.396 | rs2782648 | 0.0617 | 6.41 | -3.26964 | 457 | 457 | susie | 0.066 | 4.00E-10 | -3.22 | 1.27E-03 | 3.79E-02 |
| ENSG00000234329.1 | 1 | 45651825 | 45651826 | 0.029456 | rs1707302 | 3.488 | rs11211248 | 0.0147 | 4.59 | 3.19477 | 287 | 287 | susie | 0.028 | 4.10E-05 | 3.28 | 1.04E-03 | 3.30E-02 |
| ENSG00000230896.1 | 1 | 45697057 | 45697058 | 0.003601 | rs1707302 | 3.488 | rs11211248 | -0.000421 | -2.32 | 3.19477 | 294 | 1 | top1 | -0.00042 | 0.38 | -3.19 | 1.40E-03 | 4.02E-02 |
| ENSG00000157184.6 | 1 | 53196428 | 53196429 | 0.075429 | rs5174 | 4.195 | rs7554022 | 0.000317 | 3.37 | -1.84919 | 490 | 490 | susie | 0.013 | 0.0043 | -3.18 | 1.47E-03 | 4.13E-02 |
| ENSG00000162384.13 | 1 | 53220616 | 53220617 | 0.118128 | rs5174 | 4.195 | rs2297656 | 0.105 | -8.41 | 2.53646 | 487 | 4 | lasso | 0.11 | 1.60E-16 | -3.11 | 1.87E-03 | 4.90E-02 |
| ENSG00000162600.11 | 1 | 58546801 | 58546802 | 0.166084 | rs12139475 | -4.289 | rs6690139 | 0.131 | 9.12 | -3.6973 | 548 | 23 | enet | 0.14 | 1.40E-20 | -3.64 | 2.75E-04 | 1.27E-02 |
| ENSG00000184292.6 | 1 | 58577772 | 58577773 | 0.360703 | rs12139475 | -4.289 | rs6690139 | 0.379 | 14.73 | -3.6973 | 539 | 5 | lasso | 0.38 | 1.00E-60 | -3.69 | 2.23E-04 | 1.09E-02 |
| ENSG00000283445.1 | 1 | 58715608 | 58715609 | 0.383532 | rs12139475 | -4.289 | rs6690139 | 0.384 | 14.63 | -3.6973 | 561 | 26 | enet | 0.38 | 1.10E-60 | -3.64 | 2.68E-04 | 1.26E-02 |
| ENSG00000081985.10 | 1 | 67307363 | 67307364 | 0.270184 | rs17129794 | -5.726 | rs17129794 | 0.191 | 10.42 | -5.72632 | 498 | 498 | susie | 0.19 | 8.10E-28 | -5.59 | 2.31E-08 | 5.89E-06 |
| ENSG00000117500.12 | 1 | 93163209 | 93163210 | 0.082477 | rs10782959 | -3.33 | rs546 | 0.0712 | -6.61 | -2.93519 | 267 | 267 | susie | 0.077 | 1.40E-11 | 3.21 | 1.30E-03 | 3.85E-02 |
| ENSG00000230489.1 | 1 | 107964442 | 107964443 | 0.12519 | rs4915076 | -15.09 | rs12564085 | 0.0754 | 6.62 | 3.15946 | 460 | 4 | lasso | 0.12 | 1.10E-17 | 3.82 | 1.33E-04 | 7.29E-03 |
| ENSG00000134215.15 | 1 | 107965143 | 107965144 | 0.102644 | rs4915076 | -15.09 | rs17020055 | 0.0911 | 7.21 | -13.70851 | 460 | 3 | lasso | 0.093 | 9.50E-14 | -13.5 | 1.52E-41 | 2.45E-37 |
| ENSG00000116337.15 | 1 | 109619503 | 109619504 | 0.013256 | rs484959 | 6.28 | rs12039964 | 0.00471 | -3.58 | -3.18702 | 443 | 1 | top1 | 0.0047 | 0.057 | 3.19 | 1.44E-03 | 4.08E-02 |
| ENSG00000081026.18 | 1 | 113390748 | 113390749 | 0.015169 | rs2476601 | -27.76 | rs12723762 | 0.00875 | -3.26 | 3.15464 | 367 | 1 | top1 | 0.0088 | 0.015 | -3.15 | 1.61E-03 | 4.41E-02 |
| ENSG00000134242.15 | 1 | 113871758 | 113871759 | 0.042669 | rs2476601 | -27.76 | rs2279380 | -0.00116 | 3.6 | -2.17264 | 483 | 15 | enet | 0.008 | 0.02 | -3.56 | 3.78E-04 | 1.59E-02 |
| ENSG00000118655.4 | 1 | 113905201 | 113905202 | 0.010875 | rs2476601 | -27.76 | rs4462141 | -0.00152 | -3.13 | -1.02519 | 469 | 469 | susie | -9.00E-04 | 0.48 | 11 | 2.37E-28 | 6.37E-25 |
| ENSG00000163349.21 | 1 | 113929318 | 113929319 | 0.029586 | rs2476601 | -27.76 | rs2358996 | 0.00652 | 3.82 | 0.01296 | 472 | 17 | enet | 0.019 | 0.00068 | -3.95 | 7.69E-05 | 4.78E-03 |
| ENSG00000233154.5 | 1 | 116478841 | 116478842 | 0.014741 | rs11582506 | 5.004 | rs624988 | 0.0043 | 2.56 | -3.51553 | 394 | 1 | top1 | 0.0043 | 0.065 | -3.52 | 4.39E-04 | 1.79E-02 |
| ENSG00000134256.12 | 1 | 117001749 | 117001750 | 0.201504 | rs11582506 | 5.004 | rs12131886 | 0.118 | -8.29 | -2.94321 | 413 | 413 | susie | 0.15 | 3.00E-22 | 3.63 | 2.85E-04 | 1.29E-02 |
| ENSG00000272196.2 | 1 | 149851060 | 149851061 | 0.014125 | rs1868992 | 3.186 | rs1868992 | -0.00171 | -3.04 | 3.18577 | 119 | 1 | enet | -0.0013 | 0.58 | -3.19 | 1.44E-03 | 4.08E-02 |
| ENSG00000163154.5 | 1 | 151156628 | 151156629 | 0.003841 | rs6702842 | -4.438 | rs12097169 | 0.00478 | -3.19 | 3.96784 | 315 | 1 | top1 | 0.0048 | 0.056 | -3.97 | 7.25E-05 | 4.56E-03 |
| ENSG00000163156.11 | 1 | 151166021 | 151166022 | 0.036262 | rs6702842 | -4.438 | rs11799964 | -0.000414 | 3.92 | 1.2173 | 318 | 318 | susie | 0.0082 | 0.018 | 4.14 | 3.54E-05 | 2.51E-03 |
| ENSG00000143416.20 | 1 | 151372732 | 151372733 | 0.003254 | rs4845606 | 4.468 | rs12097169 | -0.000691 | -2.97 | 3.96784 | 334 | 1 | top1 | -0.00069 | 0.43 | -3.97 | 7.25E-05 | 4.56E-03 |
| ENSG00000159377.10 | 1 | 151399533 | 151399534 | 0.028581 | rs4845606 | 4.468 | rs7172 | 0.0015 | -3.54 | 3.65292 | 335 | 335 | susie | 0.0075 | 0.023 | -3.88 | 1.05E-04 | 6.15E-03 |
| ENSG00000143442.21 | 1 | 151459464 | 151459465 | 0.193624 | rs4845606 | 4.468 | rs11204819 | 0.198 | 10.56 | 3.87756 | 345 | 1 | top1 | 0.2 | 1.00E-28 | 3.88 | 1.06E-04 | 6.15E-03 |
| ENSG00000143376.12 | 1 | 151612399 | 151612400 | 0.015809 | rs4845606 | 4.468 | rs17641780 | -0.000256 | 3.42 | -4.28742 | 333 | 1 | top1 | -0.00026 | 0.35 | -4.29 | 1.81E-05 | 1.50E-03 |
| ENSG00000225556.1 | 1 | 151840556 | 151840557 | 0.0037 | rs4845606 | 4.468 | rs17646946 | -0.00127 | -2.74 | 0.48917 | 339 | 339 | susie | -0.001 | 0.51 | -3.15 | 1.64E-03 | 4.45E-02 |
| ENSG00000160767.20 | 1 | 155255482 | 155255483 | 0.02934 | rs3753639 | -3.717 | rs11465205 | 0.0143 | 3.79 | -2.17233 | 302 | 14 | enet | 0.015 | 0.002 | -3.56 | 3.69E-04 | 1.57E-02 |
| ENSG00000027644.4 | 1 | 156859017 | 156859018 | 0.025465 | rs926103 | -5.375 | rs2735657 | 0.000185 | 3.37 | -3.70535 | 452 | 1 | top1 | 0.00018 | 0.29 | -3.71 | 2.11E-04 | 1.05E-02 |
| ENSG00000160856.20 | 1 | 157700856 | 157700857 | 0.174602 | rs6681271 | -5.105 | rs3761959 | 0.263 | 12.18 | -5.01975 | 537 | 1 | top1 | 0.26 | 6.50E-39 | -5.02 | 5.17E-07 | 8.09E-05 |
| ENSG00000132704.15 | 1 | 157777131 | 157777132 | 0.048331 | rs6681271 | -5.105 | rs12143912 | 0.00881 | -4.12 | -1.86965 | 522 | 522 | susie | 0.014 | 0.003 | 3.29 | 9.95E-04 | 3.23E-02 |
| ENSG00000163534.14 | 1 | 157820104 | 157820105 | 0.135579 | rs6681271 | -5.105 | rs6689427 | 0.115 | -8.28 | -3.55196 | 500 | 5 | lasso | 0.13 | 2.50E-19 | 3.49 | 4.89E-04 | 1.95E-02 |
| ENSG00000228863.8 | 1 | 160670979 | 160670980 | -0.006002 | rs11265410 | 5.122 | rs10489635 | 0.00531 | 3.5 | -3.46808 | 435 | 2 | lasso | 0.0095 | 0.012 | -3.47 | 5.24E-04 | 2.05E-02 |
| ENSG00000198821.10 | 1 | 167518609 | 167518610 | 0.046103 | rs1214598 | -5.902 | rs7523907 | 0.00381 | 3.91 | -3.86184 | 608 | 6 | enet | 0.0063 | 0.034 | -3.56 | 3.76E-04 | 1.59E-02 |
| ENSG00000094975.13 | 1 | 172532348 | 172532349 | 0.05641 | rs972100 | -3.869 | rs12021702 | 0.0344 | 5.04 | -3.1199 | 392 | 1 | top1 | 0.034 | 6.10E-06 | -3.12 | 1.81E-03 | 4.82E-02 |
| ENSG00000279838.1 | 1 | 185294371 | 185294372 | 0.01046 | rs6684708 | 3.554 | rs10911707 | 0.000255 | -2.71 | -3.38521 | 389 | 1 | top1 | 0.00026 | 0.29 | 3.39 | 7.11E-04 | 2.51E-02 |
| ENSG00000213045.3 | 1 | 200329160 | 200329161 | 0.049328 | rs6660197 | -6.359 | rs2790119 | 0.0503 | 5.59 | -3.35174 | 494 | 1 | top1 | 0.05 | 5.20E-08 | -3.35 | 8.03E-04 | 2.81E-02 |
| ENSG00000118197.13 | 1 | 200669968 | 200669969 | 0.122436 | rs12742404 | -6.386 | rs12126676 | 0.0395 | -6.22 | -4.53684 | 540 | 22 | enet | 0.065 | 5.60E-10 | 4.91 | 8.91E-07 | 1.22E-04 |
| ENSG00000162892.15 | 1 | 206897442 | 206897443 | 0.046218 | rs1150258 | -4.813 | rs12022348 | 0.00838 | 4.32 | -4.70075 | 446 | 446 | susie | 0.013 | 0.0038 | -4.8 | 1.61E-06 | 1.97E-04 |
| ENSG00000271680.1 | 1 | 206905927 | 206905928 | 0.089366 | rs1150258 | -4.813 | rs1150255 | 0.0382 | 5.77 | -4.76969 | 450 | 450 | susie | 0.049 | 6.70E-08 | -4.92 | 8.68E-07 | 1.21E-04 |
| ENSG00000123685.8 | 1 | 212699984 | 212699985 | 0.169429 | rs1532951 | 4.252 | rs2221593 | 0.083 | -7.1 | 2.56922 | 433 | 9 | lasso | 0.092 | 1.40E-13 | -3.75 | 1.80E-04 | 9.27E-03 |
| ENSG00000203705.10 | 1 | 212791827 | 212791828 | 0.108333 | rs1532951 | 4.252 | rs12753524 | 0.032 | 6.06 | -3.85129 | 415 | 21 | enet | 0.051 | 3.80E-08 | -3.18 | 1.46E-03 | 4.12E-02 |
| ENSG00000258082.1 | 1 | 234980803 | 234980804 | 0.041565 | rs2891026 | 4.624 | rs16844292 | -0.00131 | -3.37 | 0.71084 | 437 | 437 | susie | 0.004 | 0.072 | 3.41 | 6.51E-04 | 2.38E-02 |
| ENSG00000173726.10 | 1 | 235128935 | 235128936 | 0.005405 | rs2891026 | 4.624 | rs9662936 | -0.00155 | 2.67 | 3.10251 | 434 | 434 | susie | 7.00E-04 | 0.24 | 3.8 | 1.45E-04 | 7.74E-03 |
| ENSG00000188739.14 | 1 | 235160247 | 235160248 | 0.025983 | rs2891026 | 4.624 | rs9662936 | 0.0117 | 4.07 | 3.10251 | 415 | 1 | top1 | 0.012 | 0.006 | 3.1 | 1.92E-03 | 4.98E-02 |
| ENSG00000116957.12 | 1 | 235367359 | 235367360 | 0.072748 | rs2891026 | 4.624 | rs2055127 | 0.0795 | 7.13 | 3.53838 | 351 | 351 | susie | 0.084 | 1.80E-12 | 3.44 | 5.85E-04 | 2.21E-02 |
| ENSG00000077585.13 | 1 | 236142504 | 236142505 | 0.259314 | rs10924341 | 4.38 | rs7543562 | 0.12 | 8.54 | 3.43585 | 517 | 517 | susie | 0.18 | 9.70E-27 | 3.19 | 1.41E-03 | 4.04E-02 |
| ENSG00000229692.3 | 2 | 38993856 | 38993857 | -0.0026 | rs4670264 | 4.49 | rs4670265 | 0.0127 | -2.01 | 3.23 | 288 | 1 | top1 | 0.013 | 0.0045 | -3.228115 | 1.25E-03 | 3.74E-02 |
| ENSG00000234936.1 | 2 | 43229572 | 43229573 | 0.111837 | rs13026309 | 4.14 | rs13407351 | 0.023 | 5 | 3.64 | 496 | 45 | enet | 0.035 | 5.50E-06 | 3.659803 | 2.52E-04 | 1.20E-02 |
| ENSG00000085760.14 | 2 | 55269346 | 55269347 | 0.066308 | rs10496044 | -5.67 | rs12622380 | 0.0236 | 4.93 | -4.03 | 480 | 2 | lasso | 0.025 | 0.00012 | -4.694093 | 2.68E-06 | 2.98E-04 |
| ENSG00000232713.2 | 2 | 60938603 | 60938604 | 0.01315 | rs13003464 | -4.55 | rs4643526 | 0.0164 | -3.84 | -3.53 | 350 | 1 | top1 | 0.016 | 0.0014 | 3.53429 | 4.09E-04 | 1.68E-02 |
| ENSG00000170340.10 | 2 | 62196112 | 62196113 | 0.489343 | rs10195033 | 5.95 | rs4672495 | 0.323 | -13.64 | 5.12 | 357 | 9 | lasso | 0.35 | 4.40E-55 | -5.55789 | 2.73E-08 | 6.77E-06 |
| ENSG00000198380.12 | 2 | 69387253 | 69387254 | 0.082598 | rs11690738 | 4.04 | rs4346430 | 0.113 | 8.3 | 3.62 | 404 | 1 | top1 | 0.11 | 1.70E-16 | 3.621735 | 2.93E-04 | 1.32E-02 |
| ENSG00000242766.1 | 2 | 90082634 | 90082635 | 0.325579 | rs17620193 | -2.71 | rs2556012 | 0.0823 | 8.4 | -2.44 | 13 | 8 | enet | 0.23 | 1.90E-33 | -3.455974 | 5.48E-04 | 2.12E-02 |
| ENSG00000172005.10 | 2 | 95025676 | 95025677 | 0.032587 | rs4854239 | -4.12 | rs4854244 | 0.0165 | 4.72 | -2.86 | 107 | 107 | susie | 0.021 | 4.00E-04 | -3.104021 | 1.91E-03 | 4.97E-02 |
| ENSG00000233850.1 | 2 | 95026708 | 95026709 | 0.007344 | rs4854239 | -4.12 | rs3105099 | -0.000786 | 2.74 | -3.19 | 107 | 107 | susie | 0.0023 | 0.13 | -3.16218 | 1.57E-03 | 4.35E-02 |
| ENSG00000144199.11 | 2 | 97094881 | 97094882 | 0.033186 | rs6718109 | -5.27 | rs1148591 | 0.0222 | -4.72 | -4.52 | 125 | 125 | susie | 0.025 | 0.00011 | 4.23334 | 2.30E-05 | 1.78E-03 |
| ENSG00000135976.17 | 2 | 97113495 | 97113496 | 0.053884 | rs6718109 | -5.27 | rs3906948 | 0.0307 | -4.87 | -5.2 | 121 | 3 | lasso | 0.038 | 1.90E-06 | 4.783076 | 1.73E-06 | 2.10E-04 |
| ENSG00000196912.12 | 2 | 97589964 | 97589965 | 0.080068 | rs5865 | -6.58 | rs3906948 | 0.099 | 7.76 | -5.2 | 118 | 118 | susie | 0.1 | 6.10E-15 | -5.324213 | 1.01E-07 | 2.09E-05 |
| ENSG00000135940.6 | 2 | 97646039 | 97646040 | 0.059687 | rs5865 | -6.58 | rs6745653 | 0.0123 | -4.35 | -3.7 | 133 | 14 | enet | 0.033 | 9.70E-06 | 4.910904 | 9.07E-07 | 1.22E-04 |
| ENSG00000228486.10 | 2 | 97664216 | 97664217 | 0.11669 | rs5865 | -6.58 | rs298913 | 0.0135 | 4.22 | -2.53 | 143 | 40 | enet | 0.04 | 1.10E-06 | -3.871581 | 1.08E-04 | 6.22E-03 |
| ENSG00000115085.13 | 2 | 97713559 | 97713560 | 0.0294 | rs5865 | -6.58 | rs746004 | 0.000326 | 3.56 | -4.58 | 166 | 1 | top1 | 0.00033 | 0.28 | -4.578675 | 4.68E-06 | 4.71E-04 |
| ENSG00000075568.16 | 2 | 97995890 | 97995891 | 0.063651 | rs5865 | -6.58 | rs11687510 | 0.0119 | -5.04 | 3.15 | 251 | 251 | susie | 0.026 | 7.20E-05 | -3.716224 | 2.02E-04 | 1.01E-02 |
| ENSG00000273306.1 | 2 | 99405217 | 99405218 | 0.033038 | rs901596 | -3.6 | rs13013984 | -0.000348 | -3.75 | 3.34 | 325 | 325 | susie | 0.014 | 0.0031 | -3.326278 | 8.80E-04 | 2.96E-02 |
| ENSG00000135945.9 | 2 | 99490034 | 99490035 | 0.078549 | rs901596 | -3.6 | rs1011633 | 0.0679 | -6.75 | -3.14 | 324 | 1 | top1 | 0.068 | 2.50E-10 | 3.137385 | 1.70E-03 | 4.57E-02 |
| ENSG00000144218.18 | 2 | 100142738 | 100142739 | 0.070765 | rs11692215 | 4.9 | rs2309811 | 0.0154 | 5.35 | -3.05 | 456 | 456 | susie | 0.037 | 3.30E-06 | -3.413786 | 6.41E-04 | 2.35E-02 |
| ENSG00000123636.17 | 2 | 159615559 | 159615560 | 0.213372 | rs2114625 | -5.5 | rs4665083 | 0.209 | 10.95 | -5.29 | 484 | 18 | enet | 0.22 | 7.90E-33 | -4.831813 | 1.35E-06 | 1.71E-04 |
| ENSG00000136560.13 | 2 | 161136907 | 161136908 | 0.036189 | rs10930013 | -5.97 | rs4664398 | -0.000547 | -3.48 | -3.83 | 365 | 365 | susie | 0.0015 | 0.18 | 5.446079 | 5.15E-08 | 1.17E-05 |
| ENSG00000227403.1 | 2 | 161244738 | 161244739 | 0.280779 | rs10930013 | -5.97 | rs10930013 | 0.193 | -10.47 | -5.97 | 385 | 25 | enet | 0.22 | 1.20E-31 | 4.935891 | 7.98E-07 | 1.16E-04 |
| ENSG00000224638.1 | 2 | 173899427 | 173899428 | 0.128519 | rs1047640 | 4.44 | rs16866 | 0.0611 | -6.48 | -3.69 | 445 | 445 | susie | 0.071 | 1.10E-10 | 3.33315 | 8.59E-04 | 2.93E-02 |
| ENSG00000271151.1 | 2 | 173968350 | 173968351 | 0.021127 | rs1047640 | 4.44 | rs4325816 | 0.0265 | -4.24 | -3.74 | 427 | 1 | top1 | 0.027 | 6.50E-05 | 3.735586 | 1.87E-04 | 9.54E-03 |
| ENSG00000170035.15 | 2 | 180967247 | 180967248 | 0.174639 | rs10200577 | -5.02 | rs10184846 | 0.185 | 10.32 | -4.63 | 389 | 389 | susie | 0.19 | 1.60E-27 | -4.606757 | 4.09E-06 | 4.25E-04 |
| ENSG00000115232.13 | 2 | 181457206 | 181457207 | 0.329349 | rs10200577 | -5.02 | rs2124440 | 0.226 | 11.44 | 3.63 | 364 | 22 | enet | 0.24 | 9.00E-36 | 3.605763 | 3.11E-04 | 1.37E-02 |
| ENSG00000188452.13 | 2 | 181657115 | 181657116 | 0.32926 | rs10200577 | -5.02 | rs1449263 | 0.224 | 11.47 | 3.39 | 387 | 6 | lasso | 0.23 | 3.50E-33 | 3.483067 | 4.96E-04 | 1.96E-02 |
| ENSG00000198130.15 | 2 | 190320044 | 190320045 | 0.133632 | rs1155060 | -7.25 | rs291449 | 0.154 | -9.51 | -3.39 | 338 | 1 | top1 | 0.15 | 3.00E-22 | 3.39489 | 6.87E-04 | 2.47E-02 |
| ENSG00000272979.1 | 2 | 190454520 | 190454521 | 0.036155 | rs1155060 | -7.25 | rs7602459 | 0.0108 | -4.1 | 3.62 | 339 | 1 | top1 | 0.011 | 0.0081 | -3.61943 | 2.95E-04 | 1.32E-02 |
| ENSG00000115419.12 | 2 | 190895151 | 190895152 | 0.048531 | rs4274624 | -12.71 | rs13395505 | 0.035 | 5.51 | -3.99 | 352 | 352 | susie | 0.039 | 1.50E-06 | -3.113449 | 1.85E-03 | 4.87E-02 |
| ENSG00000115415.18 | 2 | 191020959 | 191020960 | -0.021071 | rs4274624 | -12.71 | rs2066802 | 0.00398 | 2.93 | -3.71 | 355 | 1 | lasso | 0.0071 | 0.026 | -3.711522 | 2.06E-04 | 1.03E-02 |
| ENSG00000231858.5 | 2 | 191021525 | 191021526 | 0.00575 | rs4274624 | -12.71 | rs10497709 | 6.50E-05 | -2.84 | 3.44 | 354 | 1 | top1 | 6.50E-05 | 0.31 | -3.439759 | 5.82E-04 | 2.21E-02 |
| ENSG00000178074.5 | 2 | 199911255 | 199911256 | 0.034805 | rs1347551 | -3.99 | rs769949 | 0.0274 | 4.94 | -2.65 | 342 | 342 | susie | 0.038 | 2.00E-06 | -3.109039 | 1.88E-03 | 4.91E-02 |
| ENSG00000119004.15 | 2 | 203238939 | 203238940 | -0.005499 | rs11681040 | -7.3 | rs10932017 | 0.0015 | -3.28 | -5.99 | 297 | 1 | top1 | 0.0015 | 0.18 | 5.990368 | 2.09E-09 | 6.24E-07 |
| ENSG00000127831.10 | 2 | 218419091 | 218419092 | 0.059333 | rs4674338 | -3.78 | rs4672891 | 0.0999 | -7.57 | -3.77 | 379 | 3 | lasso | 0.1 | 7.90E-15 | 3.807459 | 1.40E-04 | 7.55E-03 |
| ENSG00000135913.10 | 2 | 218568360 | 218568361 | 0.045064 | rs4674338 | -3.78 | rs591573 | 0.0376 | 5.11 | -3.25 | 364 | 364 | susie | 0.038 | 2.30E-06 | -3.201542 | 1.37E-03 | 3.96E-02 |
| ENSG00000144580.13 | 2 | 218568579 | 218568580 | 0.119528 | rs4674338 | -3.78 | rs6720403 | 0.228 | -11.5 | -3.21 | 364 | 364 | susie | 0.23 | 8.90E-34 | 3.195391 | 1.40E-03 | 4.02E-02 |
| ENSG00000135929.8 | 2 | 218781755 | 218781756 | 0.126216 | rs4674338 | -3.78 | rs6704575 | 0.151 | 10.22 | -3.5 | 380 | 30 | enet | 0.18 | 1.70E-25 | -3.139821 | 1.69E-03 | 4.56E-02 |
| ENSG00000272555.1 | 2 | 218818689 | 218818690 | 0.055242 | rs4674338 | -3.78 | rs2556388 | 0.0642 | 6.7 | -3.08 | 377 | 377 | susie | 0.065 | 5.20E-10 | -3.237928 | 1.20E-03 | 3.68E-02 |
| ENSG00000115596.3 | 2 | 218859820 | 218859821 | 0.014031 | rs4674338 | -3.78 | rs4674338 | 0.0109 | 3.18 | -3.78 | 380 | 1 | top1 | 0.011 | 0.0078 | -3.778527 | 1.58E-04 | 8.32E-03 |
| ENSG00000135905.18 | 2 | 225042444 | 225042445 | 0.302592 | rs17199382 | 4.82 | rs6750856 | 0.236 | 11.56 | 2.96 | 393 | 33 | enet | 0.27 | 1.30E-39 | 3.434587 | 5.93E-04 | 2.23E-02 |
| ENSG00000115009.11 | 2 | 227813841 | 227813842 | 0.20189 | rs11694155 | -4.77 | rs13034664 | 0.162 | -9.59 | -4.48 | 490 | 2 | enet | 0.16 | 1.10E-23 | 5.004745 | 5.59E-07 | 8.66E-05 |
| ENSG00000263941.2 | 2 | 233205198 | 233205199 | -0.008126 | rs4430948 | -4.57 | rs13031194 | 0.00917 | -3.9 | 2.65 | 511 | 3 | lasso | 0.011 | 0.0069 | -3.634657 | 2.78E-04 | 1.28E-02 |
| ENSG00000178623.11 | 2 | 240605430 | 240605431 | 0.314602 | rs2975781 | 4.25 | rs2953158 | 0.0809 | -7.06 | -0.429 | 499 | 499 | susie | 0.2 | 1.20E-28 | 3.797648 | 1.46E-04 | 7.77E-03 |
| ENSG00000226321.5 | 2 | 240906329 | 240906330 | 0.031019 | rs10153800 | -4.99 | rs4312490 | 0.0114 | 3.82 | 3.23 | 472 | 1 | top1 | 0.011 | 0.0066 | 3.234327 | 1.22E-03 | 3.69E-02 |
| ENSG00000168385.17 | 2 | 241315099 | 241315100 | 0.617375 | rs7596240 | -5.81 | rs11681497 | 0.42 | 15.4 | 3.67 | 369 | 11 | lasso | 0.5 | 1.00E-86 | 5.138811 | 2.76E-07 | 4.78E-05 |
| ENSG00000223374.1 | 2 | 241351714 | 241351715 | 0.036975 | rs7596240 | -5.81 | rs4675812 | 0.00582 | 3.53 | 4.44 | 366 | 366 | susie | 0.018 | 0.00097 | 6.698952 | 2.10E-11 | 1.08E-08 |
| ENSG00000006607.13 | 2 | 241356242 | 241356243 | 0.160233 | rs7596240 | -5.81 | rs2018761 | 0.104 | -8.52 | 2.23 | 365 | 365 | susie | 0.16 | 1.60E-23 | -4.169793 | 3.05E-05 | 2.23E-03 |
| ENSG00000176720.4 | 2 | 241558720 | 241558721 | 0.403388 | rs7596240 | -5.81 | rs6716290 | 0.249 | -12.07 | -4.26 | 316 | 4 | lasso | 0.28 | 2.10E-41 | 4.296543 | 1.73E-05 | 1.45E-03 |
| ENSG00000168393.12 | 2 | 241686990 | 241686991 | 0.040348 | rs7596240 | -5.81 | rs34761249 | -0.00175 | -3.22 | -2.83 | 242 | 242 | susie | 0.01 | 0.01 | 4.501525 | 6.75E-06 | 6.43E-04 |
| ENSG00000180902.17 | 2 | 241734578 | 241734579 | 0.485539 | rs7596240 | -5.81 | rs6733823 | 0.228 | 11.63 | -4.08 | 221 | 14 | enet | 0.28 | 5.20E-42 | -3.771314 | 1.62E-04 | 8.48E-03 |
| ENSG00000188011.5 | 2 | 241869599 | 241869600 | 0.044049 | rs7596240 | -5.81 | rs3749155 | 0.00161 | 3.32 | -0.485 | 166 | 18 | enet | 0.0025 | 0.12 | 4.480663 | 7.44E-06 | 6.97E-04 |
| ENSG00000226423.1 | 2 | 242026175 | 242026176 | -0.008836 | rs4675929 | -4.29 | rs7425592 | -0.000898 | -2.56 | 3.54 | 113 | 1 | lasso | -0.00022 | 0.35 | -3.535028 | 4.08E-04 | 1.68E-02 |
| ENSG00000075975.15 | 3 | 12557013 | 12557014 | 0.125815 | rs6798713 | -7.49 | rs11710163 | 0.09 | 7.17 | -3.299669 | 466 | 1 | top1 | 0.09 | 2.70E-13 | -3.29967 | 9.68E-04 | 3.16E-02 |
| ENSG00000144713.12 | 3 | 12841587 | 12841588 | 0.006892 | rs895752 | -4.13 | rs9855622 | 0.000927 | -2.88 | -3.151538 | 514 | 1 | top1 | 0.00093 | 0.22 | 3.15154 | 1.62E-03 | 4.43E-02 |
| ENSG00000228956.8 | 3 | 18445023 | 18445024 | 0.021228 | rs7618405 | 3.9 | rs9820501 | -0.0017 | -2.83 | -3.624659 | 363 | 2 | lasso | -0.00044 | 0.39 | 3.22281 | 1.27E-03 | 3.79E-02 |
| ENSG00000281100.1 | 3 | 36825157 | 36825158 | 0.025503 | rs7649344 | 5.88 | rs4441609 | 0.012 | 4.05 | -4.521571 | 401 | 6 | enet | 0.018 | 0.00095 | -5.3089 | 1.10E-07 | 2.24E-05 |
| ENSG00000272334.1 | 3 | 36973671 | 36973672 | 0.016008 | rs7649344 | 5.88 | rs9311149 | -0.00111 | 3.27 | -4.948367 | 389 | 389 | susie | 0.0027 | 0.11 | -5.68019 | 1.35E-08 | 3.51E-06 |
| ENSG00000093167.17 | 3 | 37176531 | 37176532 | 0.06257 | rs7649344 | 5.88 | rs11129752 | 0.0914 | -8.07 | 5.279512 | 426 | 426 | susie | 0.1 | 9.70E-15 | -5.71398 | 1.10E-08 | 2.95E-06 |
| ENSG00000271993.1 | 3 | 37182106 | 37182107 | 0.015868 | rs7649344 | 5.88 | rs11129752 | 0.0054 | -3.54 | 5.279512 | 426 | 1 | top1 | 0.0054 | 0.045 | -5.27951 | 1.30E-07 | 2.56E-05 |
| ENSG00000198590.11 | 3 | 37386268 | 37386269 | 0.015288 | rs7649344 | 5.88 | rs6809976 | 0.00657 | 3.43 | -3.617384 | 400 | 1 | top1 | 0.0066 | 0.031 | -3.61738 | 2.98E-04 | 1.32E-02 |
| ENSG00000144668.11 | 3 | 37452114 | 37452115 | 0.119979 | rs7649344 | 5.88 | rs4452278 | 0.116 | -8.36 | 3.341079 | 405 | 405 | susie | 0.14 | 7.40E-21 | -3.13535 | 1.72E-03 | 4.61E-02 |
| ENSG00000235257.8 | 3 | 37861779 | 37861780 | 0.008386 | rs2162683 | 4.73 | rs197766 | 0.00227 | 3.19 | -3.248289 | 422 | 1 | top1 | 0.0023 | 0.13 | -3.24829 | 1.16E-03 | 3.60E-02 |
| ENSG00000228168.1 | 3 | 39334978 | 39334979 | 0.000626 | rs9825782 | -6.56 | rs12632500 | 0.00689 | 3.33 | -4.242 | 427 | 1 | top1 | 0.0069 | 0.028 | -4.242 | 2.22E-05 | 1.75E-03 |
| ENSG00000173578.7 | 3 | 46027741 | 46027742 | 0.146593 | rs2373148 | 4.9 | rs9311384 | 0.0478 | -5.88 | 4.7419 | 449 | 25 | enet | 0.09 | 2.40E-13 | -4.66056 | 3.15E-06 | 3.36E-04 |
| ENSG00000164048.13 | 3 | 48241099 | 48241100 | 0.365026 | rs6442130 | -4.44 | rs6787500 | 0.392 | 14.87 | 4.158168 | 244 | 1 | top1 | 0.39 | 3.30E-62 | 4.15817 | 3.21E-05 | 2.30E-03 |
| ENSG00000229759.1 | 3 | 48256585 | 48256586 | 0.168906 | rs7616815 | -4.5 | rs6796490 | 0.196 | 10.62 | 4.2835 | 248 | 1 | top1 | 0.2 | 1.80E-28 | 4.2835 | 1.84E-05 | 1.51E-03 |
| ENSG00000228638.1 | 3 | 48291374 | 48291375 | 0.099469 | rs7616815 | -4.5 | rs936426 | 0.103 | -8.19 | 3.879325 | 252 | 252 | susie | 0.11 | 6.00E-16 | -3.87734 | 1.06E-04 | 6.15E-03 |
| ENSG00000172113.8 | 3 | 48301684 | 48301685 | 0.215741 | rs7616815 | -4.5 | rs3197223 | 0.19 | -10.4 | 3.824769 | 255 | 32 | enet | 0.21 | 4.10E-30 | -3.86727 | 1.10E-04 | 6.29E-03 |
| ENSG00000164054.15 | 3 | 48504825 | 48504826 | 0.032587 | rs9682444 | -4.5 | rs4558783 | 0.00674 | -3.97 | 2.886682 | 251 | 251 | susie | 0.018 | 9.00E-04 | -4.16867 | 3.06E-05 | 2.23E-03 |
| ENSG00000213672.7 | 3 | 48686363 | 48686364 | 0.084029 | rs9682444 | -4.5 | rs12493578 | 0.135 | -9.34 | -3.917623 | 261 | 261 | susie | 0.14 | 1.70E-20 | 3.9142 | 9.07E-05 | 5.48E-03 |
| ENSG00000178467.17 | 3 | 48989988 | 48989989 | 0.03722 | rs9682444 | -4.5 | rs6766238 | 0.0344 | -5.36 | -3.179031 | 259 | 3 | lasso | 0.04 | 1.00E-06 | 3.19045 | 1.42E-03 | 4.06E-02 |
| ENSG00000178252.17 | 3 | 49007154 | 49007155 | 0.232401 | rs9682444 | -4.5 | rs9850134 | 0.388 | 14.85 | -4.39762 | 260 | 260 | susie | 0.4 | 8.70E-64 | -4.3213 | 1.55E-05 | 1.32E-03 |
| ENSG00000172037.13 | 3 | 49133117 | 49133118 | 0.019059 | rs9682444 | -4.5 | rs12631989 | 0.0225 | 3.93 | -3.334077 | 253 | 1 | top1 | 0.023 | 0.00022 | -3.33408 | 8.56E-04 | 2.93E-02 |
| ENSG00000177352.9 | 3 | 49166320 | 49166321 | 0.03921 | rs9682444 | -4.5 | rs3212 | 0.0309 | 5.09 | -3.993054 | 259 | 1 | top1 | 0.031 | 1.80E-05 | -3.99305 | 6.52E-05 | 4.20E-03 |
| ENSG00000114316.12 | 3 | 49340711 | 49340712 | 0.004138 | rs9840684 | -4.49 | rs7100 | -0.0018 | 2.24 | -3.166642 | 278 | 2 | lasso | -0.00037 | 0.37 | -3.16664 | 1.54E-03 | 4.29E-02 |
| ENSG00000163684.11 | 3 | 58306246 | 58306247 | 0.073712 | rs11130633 | -4.67 | rs1554125 | 0.0649 | 6.61 | 3.838457 | 459 | 4 | lasso | 0.079 | 9.40E-12 | 3.66009 | 2.52E-04 | 1.20E-02 |
| ENSG00000168297.15 | 3 | 58332879 | 58332880 | 0.169725 | rs11130633 | -4.67 | rs11130633 | 0.0865 | 7.48 | -4.670569 | 457 | 25 | enet | 0.15 | 2.10E-21 | -4.33733 | 1.44E-05 | 1.25E-03 |
| ENSG00000272182.1 | 3 | 58428254 | 58428255 | 0.021655 | rs11130633 | -4.67 | rs7633553 | -0.00179 | 3.2 | -3.640379 | 438 | 438 | susie | 0.0042 | 0.069 | -4.25194 | 2.12E-05 | 1.68E-03 |
| ENSG00000168291.12 | 3 | 58433856 | 58433857 | 0.131807 | rs11130633 | -4.67 | rs4390943 | 0.0995 | -7.65 | -3.780599 | 433 | 433 | susie | 0.1 | 1.20E-14 | 3.81505 | 1.36E-04 | 7.41E-03 |
| ENSG00000114423.18 | 3 | 105869551 | 105869552 | 0.014505 | rs13090803 | -8.87 | rs13060223 | 0.0141 | -3.22 | 4.037532 | 373 | 1 | lasso | 0.021 | 4.00E-04 | -4.03753 | 5.40E-05 | 3.57E-03 |
| ENSG00000163507.13 | 3 | 108589643 | 108589644 | 0.144883 | rs3957557 | -5.89 | rs1377843 | 0.101 | 7.84 | 4.433578 | 441 | 441 | susie | 0.11 | 2.20E-15 | 4.5486 | 5.40E-06 | 5.27E-04 |
| ENSG00000163389.10 | 3 | 119468937 | 119468938 | 0.370333 | rs1599796 | -6.98 | rs17203132 | 0.229 | 11.36 | 2.842732 | 509 | 3 | lasso | 0.26 | 3.00E-38 | 3.39087 | 6.97E-04 | 2.49E-02 |
| ENSG00000138495.6 | 3 | 119677453 | 119677454 | 0.003709 | rs1599796 | -6.98 | rs4234661 | 0.00557 | -3.53 | -4.230091 | 516 | 1 | lasso | 0.0097 | 0.011 | 4.23009 | 2.34E-05 | 1.80E-03 |
| ENSG00000243544.3 | 3 | 121654295 | 121654296 | 0.05233 | rs17203439 | -6.19 | rs11719458 | 0.0482 | 5.76 | 3.894807 | 340 | 340 | susie | 0.048 | 9.40E-08 | 3.82109 | 1.33E-04 | 7.29E-03 |
| ENSG00000145088.8 | 3 | 121835182 | 121835183 | 0.014449 | rs17203439 | -6.19 | rs1492177 | -0.00158 | -2.7 | -2.298359 | 420 | 420 | susie | -0.0013 | 0.61 | 4.53094 | 5.87E-06 | 5.70E-04 |
| ENSG00000160124.9 | 3 | 122383230 | 122383231 | 0.014519 | rs17203439 | -6.19 | rs9818482 | 0.00112 | 3.02 | -4.422143 | 472 | 1 | top1 | 0.0011 | 0.2 | -4.42214 | 9.77E-06 | 9.05E-04 |
| ENSG00000168779.19 | 3 | 158106502 | 158106503 | 0.047737 | rs4234321 | -3.86 | rs6774911 | 0.0544 | -5.69 | -3.720911 | 319 | 1 | top1 | 0.054 | 1.50E-08 | 3.72091 | 1.99E-04 | 9.99E-03 |
| ENSG00000213186.7 | 3 | 160485772 | 160485773 | 0.051632 | rs564799 | 4.24 | rs2152425 | 0.0479 | -5.93 | -2.444592 | 361 | 13 | enet | 0.056 | 9.90E-09 | 3.21706 | 1.30E-03 | 3.85E-02 |
| ENSG00000184378.2 | 3 | 169769647 | 169769648 | 0.047543 | rs12630450 | -4.8 | rs11709840 | 0.00787 | -3.95 | -3.899596 | 415 | 18 | enet | 0.025 | 1.00E-04 | 3.5496 | 3.86E-04 | 1.60E-02 |
| ENSG00000171757.15 | 3 | 169812985 | 169812986 | 0.000397 | rs12630450 | -4.8 | rs10513677 | 0.000686 | -2.4 | 0.596599 | 413 | 413 | susie | 0.0038 | 0.076 | 3.69182 | 2.23E-04 | 1.09E-02 |
| ENSG00000078070.12 | 3 | 183116074 | 183116075 | 0.09587 | rs4859148 | 4.02 | rs7640612 | 0.0452 | -5.82 | 3.380476 | 399 | 399 | susie | 0.05 | 5.10E-08 | -3.20901 | 1.33E-03 | 3.88E-02 |
| ENSG00000114859.15 | 3 | 184361650 | 184361651 | 0.069689 | rs2239856 | 3.62 | rs4912539 | 0.0125 | -4.32 | 1.407616 | 510 | 20 | enet | 0.02 | 0.00043 | -3.11568 | 1.84E-03 | 4.87E-02 |
| ENSG00000185798.7 | 3 | 196568609 | 196568610 | 0.029052 | rs11707537 | 3.53 | rs9311 | 0.00323 | 3.62 | 3.334174 | 408 | 2 | lasso | 0.0068 | 0.028 | 3.19921 | 1.38E-03 | 3.98E-02 |
| ENSG00000275426.1 | 4 | 149737 | 149738 | 0.207401 | rs17802159 | 4.02 | rs28716466 | 0.11 | -8.09 | 3.98327 | 158 | 158 | susie | 0.13 | 9.00E-19 | -3.43824 | 5.86E-04 | 2.21E-02 |
| ENSG00000250892.1 | 4 | 205008 | 205009 | 0.016988 | rs17802159 | 4.02 | rs10027536 | 0.0115 | -3.6 | 3.11577 | 180 | 1 | top1 | 0.012 | 0.0063 | -3.11577 | 1.83E-03 | 4.85E-02 |
| ENSG00000240005.5 | 4 | 26860598 | 26860599 | 0.255065 | rs4505809 | -3.42 | rs9790789 | 0.285 | 12.71 | -3.38819 | 371 | 1 | top1 | 0.29 | 1.10E-42 | -3.38819 | 7.04E-04 | 2.49E-02 |
| ENSG00000231160.9 | 4 | 38664882 | 38664883 | 0.225817 | rs337638 | 5.22 | rs9992667 | 0.202 | 10.96 | -2.90045 | 586 | 17 | enet | 0.22 | 2.00E-31 | -3.63194 | 2.81E-04 | 1.29E-02 |
| ENSG00000174123.10 | 4 | 38782989 | 38782990 | 0.052784 | rs337638 | 5.22 | rs5743595 | 0.033 | -4.54 | -3.76294 | 530 | 3 | lasso | 0.035 | 5.70E-06 | 3.73392 | 1.89E-04 | 9.61E-03 |
| ENSG00000197712.11 | 4 | 38867676 | 38867677 | 0.048903 | rs337638 | 5.22 | rs17582830 | 0.0412 | 5.23 | -3.84126 | 508 | 1 | top1 | 0.041 | 8.00E-07 | -3.84126 | 1.22E-04 | 6.85E-03 |
| ENSG00000035720.7 | 4 | 67558727 | 67558728 | 0.033046 | rs10008670 | -3.21 | rs7700004 | -0.000881 | 3.65 | -2.99323 | 410 | 11 | lasso | 0.0041 | 0.069 | -3.20858 | 1.33E-03 | 3.88E-02 |
| ENSG00000163633.10 | 4 | 86936201 | 86936202 | 0.072459 | rs13120839 | -5.87 | rs7657530 | 0.0202 | -5 | 3.19396 | 321 | 11 | enet | 0.022 | 0.00026 | -4.03466 | 5.47E-05 | 3.60E-03 |
| ENSG00000109270.12 | 4 | 99894489 | 99894490 | 0.019996 | rs6816635 | 4.46 | rs6813794 | 0.0196 | -3.97 | 3.7505 | 420 | 1 | top1 | 0.02 | 0.00053 | -3.7505 | 1.76E-04 | 9.09E-03 |
| ENSG00000164032.11 | 4 | 99950387 | 99950388 | 0.034571 | rs6816635 | 4.46 | rs2070738 | 0.0259 | -4.85 | 3.42627 | 414 | 1 | top1 | 0.026 | 8.00E-05 | -3.42627 | 6.12E-04 | 2.27E-02 |
| ENSG00000164089.8 | 4 | 108763053 | 108763054 | -0.007187 | rs219500 | 3.56 | rs219500 | 0.00179 | 2.76 | 3.55527 | 433 | 1 | top1 | 0.0018 | 0.16 | 3.55527 | 3.78E-04 | 1.59E-02 |
| ENSG00000249806.1 | 4 | 142566018 | 142566019 | 0.038116 | rs978361 | 3.54 | rs1373036 | 0.037 | -4.82 | 3.34891 | 344 | 1 | top1 | 0.037 | 2.80E-06 | -3.34891 | 8.11E-04 | 2.82E-02 |
| ENSG00000151623.14 | 4 | 148444697 | 148444698 | 0.008471 | rs17025117 | 11.79 | rs10050229 | -0.000936 | -3.13 | 3.40444 | 395 | 395 | susie | 0.0016 | 0.17 | -4.99437 | 5.90E-07 | 8.97E-05 |
| ENSG00000049656.13 | 5 | 1345098 | 1345099 | -0.001499 | rs2736100 | 4.86 | rs4975612 | 0.00339 | 2.52 | -3.7736 | 474 | 1 | top1 | 0.0034 | 0.089 | -3.7736 | 1.61E-04 | 8.45E-03 |
| ENSG00000062194.15 | 5 | 57173947 | 57173948 | 0.014507 | rs831652 | 4.61 | rs831821 | 0.00539 | 3.48 | 2.92976 | 452 | 2 | lasso | 0.007 | 0.027 | 3.66311 | 2.49E-04 | 1.20E-02 |
| ENSG00000183474.15 | 5 | 69560207 | 69560208 | 0.198933 | rs34221525 | -4.86 | rs34221525 | 0.129 | 8.63 | -4.86009 | 150 | 1 | top1 | 0.13 | 1.30E-18 | -4.86009 | 1.17E-06 | 1.51E-04 |
| ENSG00000248477.6 | 5 | 69607098 | 69607099 | 0.056151 | rs34221525 | -4.86 | rs34221525 | 0.00488 | -4 | -4.86009 | 138 | 4 | lasso | 0.012 | 0.0056 | 4.66849 | 3.03E-06 | 3.26E-04 |
| ENSG00000198237.8 | 5 | 69875270 | 69875271 | 0.056239 | rs34221525 | -4.86 | rs28431145 | -0.00131 | 2.92 | -4.05709 | 41 | 8 | enet | 0.0066 | 0.031 | -3.53609 | 4.06E-04 | 1.67E-02 |
| ENSG00000083312.17 | 5 | 72816565 | 72816566 | 0.020965 | rs4703854 | 5.6 | rs513130 | 0.0138 | -3.71 | -2.0164 | 356 | 356 | susie | 0.014 | 0.0027 | 3.67861 | 2.35E-04 | 1.14E-02 |
| ENSG00000157107.13 | 5 | 72955980 | 72955981 | 0.199086 | rs34958 | -5.45 | rs7713398 | 0.195 | 10.55 | -5.08267 | 350 | 350 | susie | 0.21 | 3.90E-31 | -5.58466 | 2.34E-08 | 5.89E-06 |
| ENSG00000164307.12 | 5 | 96808099 | 96808100 | 0.63684 | rs1421911 | 3.96 | rs1057569 | 0.374 | -15.42 | -2.93479 | 494 | 494 | susie | 0.71 | 2.50E-150 | 3.29177 | 9.96E-04 | 3.23E-02 |
| ENSG00000145723.16 | 5 | 103120147 | 103120148 | 0.031243 | rs26232 | 6.72 | rs26262 | 0.0129 | -3.97 | 4.67418 | 318 | 318 | susie | 0.021 | 0.00031 | -4.5789 | 4.67E-06 | 4.71E-04 |
| ENSG00000145725.19 | 5 | 103120151 | 103120152 | 0.263805 | rs26232 | 6.72 | rs1011454 | 0.386 | -15.03 | 4.43791 | 318 | 318 | susie | 0.42 | 5.90E-68 | -4.3343 | 1.46E-05 | 1.26E-03 |
| ENSG00000155329.11 | 5 | 133026603 | 133026604 | -0.011571 | rs803137 | 3.7 | rs2706338 | 0.000432 | 2.67 | 3.52481 | 405 | 1 | top1 | 0.00043 | 0.27 | 3.52481 | 4.24E-04 | 1.73E-02 |
| ENSG00000279691.1 | 5 | 133052768 | 133052769 | 0.004674 | rs803137 | 3.7 | rs25736 | -0.000168 | 2.47 | -0.20003 | 411 | 411 | susie | 0.0057 | 0.041 | -3.78687 | 1.53E-04 | 8.09E-03 |
| ENSG00000248559.1 | 5 | 134399494 | 134399495 | 0.008482 | rs244673 | -7.3 | rs1476095 | 0.00293 | -2.81 | -3.59322 | 291 | 1 | top1 | 0.0029 | 0.11 | 3.59322 | 3.27E-04 | 1.42E-02 |
| ENSG00000184584.12 | 5 | 139482789 | 139482790 | 0.043415 | rs13153461 | 4.92 | rs7448031 | 0.0238 | -4.4 | 3.48116 | 267 | 1 | top1 | 0.024 | 0.00015 | -3.48116 | 4.99E-04 | 1.97E-02 |
| ENSG00000272112.1 | 5 | 151725355 | 151725356 | 0.001553 | rs4958490 | -3.72 | rs2033466 | 0.000942 | 2.9 | -3.19876 | 564 | 1 | lasso | 0.0046 | 0.058 | -3.19876 | 1.38E-03 | 3.98E-02 |
| ENSG00000113249.12 | 5 | 157059118 | 157059119 | 0.152103 | rs2434971 | -5.54 | rs919745 | 0.105 | -8.37 | -3.409 | 470 | 470 | susie | 0.11 | 1.10E-16 | 4.00877 | 6.10E-05 | 3.97E-03 |
| ENSG00000135077.8 | 5 | 157109713 | 157109714 | 0.064662 | rs2434971 | -5.54 | rs3087616 | 0.0433 | 5.31 | -3.56385 | 474 | 1 | top1 | 0.043 | 4.20E-07 | -3.56385 | 3.65E-04 | 1.56E-02 |
| ENSG00000137265.14 | 6 | 391738 | 391739 | 0.061777 | rs1050976 | -7.3 | rs12203592 | 0.0811 | 6.91 | -4.9264 | 420 | 1 | top1 | 0.081 | 4.30E-12 | -4.92641 | 8.38E-07 | 1.17E-04 |
| ENSG00000021355.12 | 6 | 2842005 | 2842006 | 0.082807 | rs409346 | -3.7 | rs316346 | 0.0441 | -5.27 | -3.2838 | 657 | 1 | top1 | 0.044 | 3.30E-07 | 3.28385 | 1.02E-03 | 3.27E-02 |
| ENSG00000272462.2 | 6 | 25992661 | 25992662 | 0.075633 | rs6923139 | -8.89 | rs13212534 | 0.0432 | 6.24 | -7.0122 | 679 | 14 | enet | 0.081 | 4.10E-12 | -7.48021 | 7.42E-14 | 5.98E-11 |
| ENSG00000124562.9 | 6 | 34757405 | 34757406 | 0.038391 | rs2744961 | -5.39 | rs2744961 | 0.048 | -5.32 | -5.3879 | 354 | 1 | top1 | 0.048 | 1.00E-07 | 5.38791 | 7.13E-08 | 1.51E-05 |
| ENSG00000065060.16 | 6 | 34792014 | 34792015 | 0.254838 | rs2744961 | -5.39 | rs11759151 | 0.395 | 15.2 | -4.1917 | 356 | 356 | susie | 0.41 | 3.40E-66 | -4.25434 | 2.10E-05 | 1.68E-03 |
| ENSG00000064995.16 | 6 | 34888088 | 34888089 | 0.033246 | rs2744961 | -5.39 | rs1888822 | 0.00772 | 3.88 | 3.1071 | 374 | 374 | susie | 0.0091 | 0.014 | 3.22956 | 1.24E-03 | 3.73E-02 |
| ENSG00000065029.14 | 6 | 35258981 | 35258982 | 0.095712 | rs3800373 | 6.46 | rs3800385 | 0.118 | -8.96 | 3.0851 | 430 | 4 | lasso | 0.13 | 2.60E-19 | -3.23421 | 1.22E-03 | 3.69E-02 |
| ENSG00000023892.10 | 6 | 35297851 | 35297852 | 0.283858 | rs3800373 | 6.46 | rs10755687 | 0.26 | -12.16 | 3.2092 | 436 | 1 | top1 | 0.26 | 1.70E-38 | -3.20922 | 1.33E-03 | 3.88E-02 |
| ENSG00000096060.14 | 6 | 35728582 | 35728583 | 0.021886 | rs3800373 | 6.46 | rs10755687 | 0.00578 | -3.61 | 3.2092 | 412 | 1 | top1 | 0.0058 | 0.04 | -3.20922 | 1.33E-03 | 3.88E-02 |
| ENSG00000237719.1 | 6 | 36092645 | 36092646 | -0.014907 | rs7751598 | 5.96 | rs9380529 | -0.00122 | 2.89 | -4.9109 | 376 | 1 | top1 | -0.0012 | 0.57 | -4.91086 | 9.07E-07 | 1.22E-04 |
| ENSG00000137168.7 | 6 | 36875023 | 36875024 | 0.057049 | rs1724088 | -5.63 | rs6912602 | 0.0268 | -5.14 | -3.1615 | 529 | 529 | susie | 0.033 | 8.40E-06 | 3.14357 | 1.67E-03 | 4.52E-02 |
| ENSG00000146192.14 | 6 | 37005646 | 37005647 | 0.16875 | rs1724088 | -5.63 | rs831512 | 0.0786 | -7.44 | -3.6849 | 529 | 3 | lasso | 0.091 | 2.20E-13 | 3.55398 | 3.79E-04 | 1.59E-02 |
| ENSG00000153291.15 | 6 | 46652914 | 46652915 | 0.025241 | rs974670 | -3.58 | rs974670 | 0.018 | -3.89 | -3.579 | 499 | 1 | top1 | 0.018 | 0.00086 | 3.57901 | 3.45E-04 | 1.48E-02 |
| ENSG00000188994.12 | 6 | 87155285 | 87155286 | 0.033025 | rs242267 | 4.06 | rs373646 | 0.0274 | 4.97 | 4.0335 | 382 | 382 | susie | 0.031 | 1.50E-05 | 3.95303 | 7.72E-05 | 4.78E-03 |
| ENSG00000111850.10 | 6 | 87322674 | 87322675 | 0.096915 | rs242267 | 4.06 | rs9359743 | 0.0674 | 6.85 | -3.3525 | 406 | 406 | susie | 0.074 | 3.70E-11 | -3.4369 | 5.88E-04 | 2.21E-02 |
| ENSG00000164414.17 | 6 | 87470622 | 87470623 | 0.17146 | rs242267 | 4.06 | rs2268993 | 0.249 | -11.88 | 3.1263 | 445 | 445 | susie | 0.25 | 6.80E-37 | -3.27586 | 1.05E-03 | 3.32E-02 |
| ENSG00000146281.5 | 6 | 89146049 | 89146050 | 0.084794 | rs393203 | -3.9 | rs6921997 | 0.0367 | -4.95 | -3.6283 | 406 | 1 | top1 | 0.037 | 3.10E-06 | 3.62835 | 2.85E-04 | 1.29E-02 |
| ENSG00000132423.11 | 6 | 99394203 | 99394204 | 0.014147 | rs4839737 | 3.36 | rs4839737 | -0.00118 | 2.97 | 3.356 | 472 | 1 | top1 | -0.0012 | 0.56 | 3.35604 | 7.91E-04 | 2.77E-02 |
| ENSG00000057657.15 | 6 | 106086319 | 106086320 | 0.016034 | rs9386512 | -4.68 | rs10499050 | 0.0217 | 3.81 | -3.1482 | 574 | 1 | top1 | 0.022 | 0.00028 | -3.14821 | 1.64E-03 | 4.45E-02 |
| ENSG00000269919.1 | 6 | 106100139 | 106100140 | -0.020762 | rs9386512 | -4.68 | rs10499050 | -0.00174 | 2.99 | -3.1482 | 568 | 1 | lasso | 0.0082 | 0.018 | -3.14821 | 1.64E-03 | 4.45E-02 |
| ENSG00000178409.13 | 6 | 107115268 | 107115269 | 0.004745 | rs4946810 | -3.93 | rs4946810 | 0.0021 | -3.65 | -3.9293 | 377 | 2 | lasso | 0.013 | 0.0038 | 3.86995 | 1.09E-04 | 6.25E-03 |
| ENSG00000272476.1 | 6 | 107957412 | 107957413 | 0.029927 | rs9320236 | -3 | rs17528372 | -0.00102 | -2.98 | -2.587 | 419 | 3 | lasso | -0.00053 | 0.4 | 3.24987 | 1.15E-03 | 3.58E-02 |
| ENSG00000203801.8 | 6 | 108751653 | 108751654 | 0.047323 | rs12208578 | -4.66 | rs12208908 | 0.00849 | -3.49 | -0.3523 | 347 | 347 | susie | 0.018 | 0.00085 | 3.95228 | 7.74E-05 | 4.78E-03 |
| ENSG00000271789.1 | 6 | 111297125 | 111297126 | 0.010223 | rs9384805 | -5.04 | rs17679731 | -0.000529 | -2.97 | -1.9738 | 393 | 393 | susie | -0.00015 | 0.34 | 3.68765 | 2.26E-04 | 1.10E-02 |
| ENSG00000112769.18 | 6 | 112254938 | 112254939 | 0.008335 | rs9384805 | -5.04 | rs2012071 | -0.00156 | -2.82 | 4.1695 | 450 | 1 | lasso | 0.0048 | 0.056 | -4.16951 | 3.05E-05 | 2.23E-03 |
| ENSG00000146373.16 | 6 | 124962544 | 124962545 | 0.047338 | rs1413595 | -3.39 | rs1413595 | 0.0352 | 4.73 | -3.3881 | 493 | 1 | lasso | 0.039 | 1.70E-06 | -3.3881 | 7.04E-04 | 2.49E-02 |
| ENSG00000118520.14 | 6 | 131573143 | 131573144 | 0.265199 | rs3756784 | -4.3 | rs2246012 | 0.366 | -14.29 | -4.08 | 398 | 1 | top1 | 0.37 | 4.00E-57 | 4.08004 | 4.50E-05 | 3.07E-03 |
| ENSG00000112282.17 | 6 | 131628228 | 131628229 | 0.132855 | rs3756784 | -4.3 | rs2245133 | 0.12 | -8.42 | -3.9423 | 395 | 1 | top1 | 0.12 | 2.10E-17 | 3.94231 | 8.07E-05 | 4.95E-03 |
| ENSG00000146409.10 | 6 | 132798552 | 132798553 | 0.375041 | rs7751879 | 4.3 | rs12204740 | 0.336 | -13.77 | 3.2751 | 534 | 534 | susie | 0.34 | 3.10E-52 | -3.28345 | 1.03E-03 | 3.29E-02 |
| ENSG00000135541.20 | 6 | 135497775 | 135497776 | 0.171603 | rs6925090 | -6.09 | rs6908428 | 0.238 | -11.76 | -5.6949 | 358 | 358 | susie | 0.24 | 1.70E-35 | 5.6985 | 1.21E-08 | 3.20E-06 |
| ENSG00000171408.13 | 6 | 135851695 | 135851696 | 0.026404 | rs6925090 | -6.09 | rs9399176 | -0.0018 | -2.89 | 0.0662 | 311 | 311 | susie | 0.0084 | 0.017 | 5.23193 | 1.68E-07 | 3.19E-05 |
| ENSG00000029639.10 | 6 | 155314492 | 155314493 | 0.004722 | rs9480081 | 3.35 | rs7742211 | 0.00285 | -3.45 | 3.1857 | 516 | 1 | top1 | 0.0029 | 0.11 | -3.18573 | 1.44E-03 | 4.08E-02 |
| ENSG00000112096.16 | 6 | 159762493 | 159762494 | -0.004486 | rs7855 | 3.81 | rs7855 | 0.000951 | 3.33 | 3.8092 | 544 | 1 | top1 | 0.00095 | 0.22 | 3.80916 | 1.39E-04 | 7.52E-03 |
| ENSG00000071242.11 | 6 | 166906450 | 166906451 | 0.22939 | rs933243 | 11.98 | rs2281144 | 0.137 | -8.86 | -1.1003 | 690 | 690 | susie | 0.2 | 1.70E-28 | 7.21298 | 5.47E-13 | 3.53E-10 |
| ENSG00000026297.15 | 6 | 166957183 | 166957184 | 0.517484 | rs933243 | 11.98 | rs429083 | 0.485 | 16.46 | 10.871 | 684 | 25 | enet | 0.49 | 1.90E-84 | 11.23399 | 2.78E-29 | 8.96E-26 |
| ENSG00000227598.1 | 6 | 166999064 | 166999065 | 0.053483 | rs933243 | 11.98 | rs239935 | 0.0281 | 4.76 | 10.7646 | 631 | 1 | top1 | 0.028 | 4.00E-05 | 10.76457 | 5.06E-27 | 9.06E-24 |
| ENSG00000213066.11 | 6 | 166999181 | 166999182 | 0.146132 | rs933243 | 11.98 | rs9459874 | 0.0813 | 6.85 | -8.8966 | 631 | 14 | enet | 0.11 | 6.00E-16 | -7.23533 | 4.64E-13 | 3.12E-10 |
| ENSG00000112486.15 | 6 | 167111806 | 167111807 | 0.072269 | rs933243 | 11.98 | rs3093026 | 0.0543 | -6.08 | 6.2012 | 541 | 541 | susie | 0.056 | 8.70E-09 | -6.88166 | 5.92E-12 | 3.41E-09 |
| ENSG00000164916.10 | 7 | 4682308 | 4682309 | 0.068747 | rs7784748 | -5.78 | rs7784748 | 0.0466 | 5.62 | -5.7798 | 449 | 9 | enet | 0.05 | 5.50E-08 | -5.08316 | 3.71E-07 | 6.16E-05 |
| ENSG00000178397.12 | 7 | 6348980 | 6348981 | 0.134448 | rs2042524 | -4.3 | rs2042524 | 0.127 | 8.47 | -4.3045 | 297 | 1 | top1 | 0.13 | 2.30E-18 | -4.3045 | 1.67E-05 | 1.41E-03 |
| ENSG00000136238.17 | 7 | 6374522 | 6374523 | 0.272327 | rs2042524 | -4.3 | rs836549 | 0.238 | 11.79 | -3.3721 | 295 | 9 | lasso | 0.25 | 5.70E-37 | -3.86004 | 1.13E-04 | 6.37E-03 |
| ENSG00000215045.8 | 7 | 6551435 | 6551436 | 0.006455 | rs2042524 | -4.3 | rs17198191 | -0.00138 | -3.01 | 0.2433 | 283 | 283 | susie | -0.0012 | 0.55 | -3.45251 | 5.55E-04 | 2.13E-02 |
| ENSG00000050344.8 | 7 | 26152239 | 26152240 | 0.132678 | rs2057763 | 4.93 | rs2057763 | 0.0977 | -7.47 | 4.928 | 528 | 1 | top1 | 0.098 | 2.50E-14 | -4.92804 | 8.31E-07 | 1.17E-04 |
| ENSG00000222004.7 | 7 | 26637870 | 26637871 | 0.042044 | rs7777206 | -4.29 | rs10247053 | 0.00751 | -4.33 | -3.9146 | 450 | 15 | enet | 0.027 | 6.40E-05 | 3.76795 | 1.65E-04 | 8.61E-03 |
| ENSG00000106066.14 | 7 | 29195450 | 29195451 | 0.360302 | rs245897 | 3.41 | rs245883 | 0.223 | 11.3 | 3.3878 | 692 | 38 | enet | 0.27 | 3.90E-40 | 3.58703 | 3.34E-04 | 1.44E-02 |
| ENSG00000136271.10 | 7 | 44575050 | 44575051 | 0.107789 | rs3735477 | -5.03 | rs217378 | 0.0527 | 6.2 | -4.0852 | 361 | 1 | top1 | 0.053 | 2.50E-08 | -4.08517 | 4.40E-05 | 3.02E-03 |
| ENSG00000158604.14 | 7 | 44582286 | 44582287 | 0.06762 | rs3735477 | -5.03 | rs217378 | 0.0754 | 6.72 | -4.0852 | 363 | 1 | top1 | 0.075 | 2.50E-11 | -4.08517 | 4.40E-05 | 3.02E-03 |
| ENSG00000105953.14 | 7 | 44606571 | 44606572 | 0.073306 | rs3735477 | -5.03 | rs3735477 | 0.0191 | -4.69 | -5.0312 | 364 | 3 | lasso | 0.038 | 2.20E-06 | 4.28682 | 1.81E-05 | 1.50E-03 |
| ENSG00000135211.5 | 7 | 77798579 | 77798580 | 0.120613 | rs17156320 | -5.3 | rs1544457 | 0.116 | 8.47 | 4.4067 | 518 | 518 | susie | 0.12 | 7.30E-17 | 3.83777 | 1.24E-04 | 6.94E-03 |
| ENSG00000160813.6 | 7 | 100436564 | 100436565 | 0.026743 | rs221774 | 6.3 | rs2075672 | -0.000127 | 3.56 | 1.6976 | 310 | 310 | susie | 0.0025 | 0.12 | 3.32145 | 8.96E-04 | 3.01E-02 |
| ENSG00000106351.12 | 7 | 100539210 | 100539211 | -0.004892 | rs221774 | 6.3 | rs7457868 | 0.00331 | -1.76 | 3.6162 | 305 | 1 | top1 | 0.0033 | 0.092 | -3.61615 | 2.99E-04 | 1.32E-02 |
| ENSG00000146830.9 | 7 | 100689447 | 100689448 | 0.203322 | rs221774 | 6.3 | rs564449 | 0.123 | 8.78 | 6.0019 | 320 | 320 | susie | 0.18 | 9.90E-27 | 5.43779 | 5.39E-08 | 1.21E-05 |
| ENSG00000128563.13 | 7 | 102363871 | 102363872 | 0.07547 | rs803118 | -3.52 | rs803118 | 0.0481 | 5.99 | -3.5232 | 250 | 250 | susie | 0.048 | 8.90E-08 | -3.40412 | 6.64E-04 | 2.42E-02 |
| ENSG00000161057.11 | 7 | 103344253 | 103344254 | 0.016358 | rs6975450 | -3.48 | rs6975450 | 0.00484 | 3.56 | -3.4807 | 392 | 1 | top1 | 0.0048 | 0.055 | -3.48071 | 5.00E-04 | 1.97E-02 |
| ENSG00000128604.19 | 7 | 128937611 | 128937612 | 0.448716 | rs17340542 | -5.33 | rs4728142 | 0.339 | 14.3 | -4.4158 | 332 | 332 | susie | 0.43 | 5.00E-70 | -3.33644 | 8.49E-04 | 2.91E-02 |
| ENSG00000230359.5 | 7 | 129055222 | 129055223 | 0.034969 | rs17340542 | -5.33 | rs13227095 | 0.0276 | -4.31 | -4.1189 | 351 | 1 | top1 | 0.028 | 4.80E-05 | 4.11886 | 3.81E-05 | 2.68E-03 |
| ENSG00000229677.1 | 7 | 139050005 | 139050006 | 0.065597 | rs1604912 | 3.13 | rs5022943 | 0.0021 | 3.38 | 2.6467 | 416 | 416 | susie | 0.0047 | 0.057 | 3.12886 | 1.75E-03 | 4.67E-02 |
| ENSG00000197362.14 | 7 | 149090781 | 149090782 | 0.101498 | rs4727027 | -3.73 | rs1405124 | 0.0713 | -6.49 | -3.3378 | 378 | 1 | top1 | 0.071 | 8.70E-11 | 3.33782 | 8.44E-04 | 2.90E-02 |
| ENSG00000197024.8 | 7 | 149126415 | 149126416 | 0.041234 | rs4727027 | -3.73 | rs4727019 | 0.0217 | -4.42 | -3.5372 | 375 | 375 | susie | 0.025 | 9.40E-05 | 3.57179 | 3.55E-04 | 1.52E-02 |
| ENSG00000170265.11 | 7 | 149195484 | 149195485 | 0.238841 | rs4727027 | -3.73 | rs13225884 | 0.316 | -13.47 | -3.5646 | 377 | 377 | susie | 0.32 | 2.90E-48 | 3.62066 | 2.94E-04 | 1.32E-02 |
| ENSG00000253981.5 | 8 | 8244039 | 8244040 | 0.058224 | rs2979179 | 10.2 | rs10087493 | 0.0617 | 6.07 | 7.9004 | 342 | 1 | top1 | 0.062 | 1.70E-09 | 7.9004 | 2.78E-15 | 2.80E-12 |
| ENSG00000275342.4 | 8 | 8386497 | 8386498 | 0.011358 | rs2979179 | 10.2 | rs17149208 | -0.00175 | 3.41 | 4.41975 | 438 | 2 | lasso | 0.0019 | 0.15 | 6.34517 | 2.22E-10 | 8.95E-08 |
| ENSG00000147324.10 | 8 | 8893644 | 8893645 | 0.100299 | rs2979179 | 10.2 | rs11781985 | 0.033 | -5.72 | -4.0758 | 702 | 8 | lasso | 0.034 | 7.40E-06 | 4.71505 | 2.42E-06 | 2.73E-04 |
| ENSG00000233609.3 | 8 | 8961199 | 8961200 | 0.181166 | rs2979179 | 10.2 | rs6981573 | 0.162 | 9.66 | 4.30425 | 716 | 716 | susie | 0.17 | 4.90E-24 | 3.92093 | 8.82E-05 | 5.38E-03 |
| ENSG00000173273.15 | 8 | 9556520 | 9556521 | -0.006107 | rs719409 | 7.12 | rs329985 | 0.00357 | 2.44 | -4.01778 | 590 | 1 | top1 | 0.0036 | 0.084 | -4.01778 | 5.87E-05 | 3.85E-03 |
| ENSG00000253230.8 | 8 | 9905365 | 9905366 | 0.010376 | rs9329221 | 6.78 | rs12680442 | -0.00176 | -3.05 | 1.44258 | 612 | 612 | susie | -0.00036 | 0.37 | -4.27035 | 1.95E-05 | 1.59E-03 |
| ENSG00000261451.1 | 8 | 10433671 | 10433672 | 0.038041 | rs7831557 | 7.16 | rs1484648 | 0.00359 | 4.12 | 3.98966 | 708 | 708 | susie | 0.0077 | 0.021 | 4.75562 | 1.98E-06 | 2.36E-04 |
| ENSG00000253641.5 | 8 | 10474564 | 10474565 | 0.029648 | rs7831557 | 7.16 | rs6601450 | -0.00166 | 4.02 | 6.50422 | 708 | 3 | lasso | 0.0033 | 0.092 | 7.08892 | 1.35E-12 | 8.37E-10 |
| ENSG00000272505.1 | 8 | 10486806 | 10486807 | 0.168036 | rs7831557 | 7.16 | rs4448276 | 0.0177 | 5.92 | 4.07741 | 709 | 25 | enet | 0.11 | 6.60E-16 | 4.73359 | 2.21E-06 | 2.54E-04 |
| ENSG00000269918.1 | 8 | 11106703 | 11106704 | 0.150771 | rs2618443 | 7.55 | rs6601563 | 0.128 | -8.69 | -4.87275 | 626 | 7 | lasso | 0.14 | 5.50E-20 | 5.20551 | 1.93E-07 | 3.58E-05 |
| ENSG00000255310.2 | 8 | 11109725 | 11109726 | 0.085301 | rs2618443 | 7.55 | rs7832722 | 0.0768 | -7.29 | -4.85667 | 625 | 625 | susie | 0.081 | 4.30E-12 | 4.89572 | 9.79E-07 | 1.27E-04 |
| ENSG00000171044.10 | 8 | 11201365 | 11201366 | 0.007889 | rs2618443 | 7.55 | rs11991118 | -0.00138 | 3.11 | -5.25417 | 637 | 637 | susie | -0.0011 | 0.53 | -6.32692 | 2.50E-10 | 9.83E-08 |
| ENSG00000177710.5 | 8 | 11330887 | 11330888 | 0.096651 | rs2618443 | 7.55 | rs3779891 | 0.0822 | -7.05 | 4.74824 | 638 | 1 | top1 | 0.082 | 3.00E-12 | -4.74824 | 2.05E-06 | 2.41E-04 |
| ENSG00000154316.16 | 8 | 11339636 | 11339637 | 0.006375 | rs2618443 | 7.55 | rs11250164 | 0.00314 | 2.69 | -4.39505 | 637 | 1 | top1 | 0.0031 | 0.097 | -4.39505 | 1.11E-05 | 9.88E-04 |
| ENSG00000154328.15 | 8 | 11769638 | 11769639 | 0.209486 | rs2618443 | 7.55 | rs2686187 | 0.0813 | 7.83 | 4.07735 | 475 | 14 | enet | 0.12 | 1.60E-17 | 3.59695 | 3.22E-04 | 1.41E-02 |
| ENSG00000079459.12 | 8 | 11795572 | 11795573 | 0.123225 | rs2618443 | 7.55 | rs7001819 | 0.0723 | -6.71 | -4.65217 | 468 | 15 | enet | 0.086 | 1.00E-12 | 4.92734 | 8.34E-07 | 1.17E-04 |
| ENSG00000269899.1 | 8 | 11846390 | 11846391 | 0.020338 | rs2618443 | 7.55 | rs1298295 | -0.0018 | 3.57 | 4.93103 | 439 | 5 | enet | -0.0013 | 0.61 | 4.90309 | 9.43E-07 | 1.24E-04 |
| ENSG00000253483.1 | 8 | 23483533 | 23483534 | 0.081201 | rs9314268 | 5.02 | rs17698981 | -0.00103 | -3.67 | 3.75506 | 585 | 5 | lasso | -4.00E-04 | 0.38 | -4.48795 | 7.19E-06 | 6.78E-04 |
| ENSG00000147454.13 | 8 | 23528804 | 23528805 | 0.090495 | rs9314268 | 5.02 | rs6557679 | 0.00543 | -4.1 | -2.43991 | 579 | 579 | susie | 0.018 | 0.00085 | 3.63014 | 2.83E-04 | 1.29E-02 |
| ENSG00000271869.1 | 8 | 30156231 | 30156232 | 0.164353 | rs7845816 | -4.08 | rs4733396 | 0.149 | 9.67 | 3.37423 | 368 | 368 | susie | 0.16 | 1.90E-22 | 3.26858 | 1.08E-03 | 3.38E-02 |
| ENSG00000175324.9 | 8 | 38176531 | 38176532 | 0.000463 | rs7845911 | 3.85 | rs35296192 | -0.00106 | 2.88 | 0.28668 | 253 | 253 | susie | 0.0084 | 0.017 | 3.41515 | 6.37E-04 | 2.34E-02 |
| ENSG00000156735.10 | 8 | 38176730 | 38176731 | 0.056841 | rs7845911 | 3.85 | rs2280847 | 0.038 | 5.36 | 3.31951 | 253 | 253 | susie | 0.039 | 1.50E-06 | 3.54967 | 3.86E-04 | 1.60E-02 |
| ENSG00000085788.13 | 8 | 38225217 | 38225218 | 0.01926 | rs7845911 | 3.85 | rs2306899 | 0.000118 | 3.19 | 3.75796 | 275 | 275 | susie | 0.0027 | 0.11 | 3.72954 | 1.92E-04 | 9.70E-03 |
| ENSG00000272092.1 | 8 | 38382363 | 38382364 | 0.039565 | rs7845911 | 3.85 | rs6981405 | 0.0183 | -4.12 | 3.76171 | 270 | 270 | susie | 0.023 | 0.00017 | -3.67956 | 2.34E-04 | 1.14E-02 |
| ENSG00000104388.14 | 8 | 60516856 | 60516857 | 0.103798 | rs4354350 | -6.25 | rs671275 | 0.0976 | 7.6 | -4.3914 | 401 | 1 | top1 | 0.098 | 2.60E-14 | -4.3914 | 1.13E-05 | 1.00E-03 |
| ENSG00000104450.12 | 8 | 100157905 | 100157906 | 0.184357 | rs17425247 | 4.56 | rs2514680 | 0.19 | -11.19 | 1.91313 | 371 | 21 | enet | 0.27 | 7.50E-40 | -3.11015 | 1.87E-03 | 4.90E-02 |
| ENSG00000189376.11 | 8 | 123241397 | 123241398 | 0.077319 | rs6987216 | 3.43 | rs16898127 | 0.00257 | -4.06 | -0.08256 | 545 | 24 | enet | 0.0052 | 0.049 | 3.11767 | 1.82E-03 | 4.83E-02 |
| ENSG00000136997.16 | 8 | 127735433 | 127735434 | -0.028781 | rs1121946 | 7.03 | rs10492294 | -0.00113 | -3 | 4.90518 | 623 | 1 | top1 | -0.0011 | 0.54 | -4.90518 | 9.33E-07 | 1.24E-04 |
| ENSG00000249859.9 | 8 | 127794532 | 127794533 | 0.198361 | rs1121946 | 7.03 | rs16902510 | 0.124 | 8.43 | -5.14706 | 619 | 3 | lasso | 0.13 | 7.30E-19 | -4.20906 | 2.56E-05 | 1.93E-03 |
| ENSG00000129295.8 | 8 | 132675616 | 132675617 | 0.600112 | rs1810396 | -8.12 | rs1048490 | 0.53 | -17.2 | -4.64405 | 605 | 54 | enet | 0.59 | 7.10E-111 | 3.90836 | 9.29E-05 | 5.55E-03 |
| ENSG00000270137.1 | 8 | 132826178 | 132826179 | 0.009443 | rs1810396 | -8.12 | rs4520144 | 0.000457 | -2.66 | -3.26234 | 664 | 1 | top1 | 0.00046 | 0.26 | 3.26234 | 1.10E-03 | 3.44E-02 |
| ENSG00000042832.11 | 8 | 132866957 | 132866958 | 0.025586 | rs1810396 | -8.12 | rs10092556 | -0.000725 | -3.15 | 6.29686 | 663 | 663 | susie | -0.00029 | 0.36 | -7.33826 | 2.16E-13 | 1.58E-10 |
| ENSG00000104472.9 | 8 | 140511297 | 140511298 | 0.033294 | rs10088596 | -5.98 | rs1568607 | 0.00523 | -3.4 | -0.84611 | 379 | 379 | susie | 0.0058 | 0.039 | 3.22802 | 1.25E-03 | 3.74E-02 |
| ENSG00000105339.10 | 8 | 141117277 | 141117278 | 0.082607 | rs10088596 | -5.98 | rs1045303 | 0.0209 | -5.27 | 3.31514 | 427 | 11 | enet | 0.043 | 4.90E-07 | -3.21204 | 1.32E-03 | 3.88E-02 |
| ENSG00000198642.6 | 9 | 21335379 | 21335380 | -0.002589 | rs970987 | -8.304 | rs871024 | -0.00105 | -2.799 | -3.90032 | 482 | 2 | lasso | -3.70E-05 | 0.32 | 3.90033 | 9.61E-05 | 5.67E-03 |
| ENSG00000099810.18 | 9 | 21802542 | 21802543 | 0.069541 | rs970987 | -8.304 | rs1341866 | 0.0184 | -4.737 | -3.61089 | 445 | 445 | susie | 0.022 | 0.00024 | 3.66554 | 2.47E-04 | 1.19E-02 |
| ENSG00000147889.17 | 9 | 21995300 | 21995301 | 0.020769 | rs970987 | -8.304 | rs3731217 | 0.0353 | 4.992 | 3.15558 | 447 | 1 | top1 | 0.035 | 4.70E-06 | 3.15558 | 1.60E-03 | 4.40E-02 |
| ENSG00000165030.3 | 9 | 91423861 | 91423862 | 0.118783 | rs7020893 | -3.86 | rs10991853 | 0.073 | 7.114 | -3.82359 | 522 | 522 | susie | 0.077 | 1.80E-11 | -3.90535 | 9.41E-05 | 5.58E-03 |
| ENSG00000136842.13 | 9 | 97501179 | 97501180 | 0.015676 | rs7850258 | 21.059 | rs913912 | 0.00851 | -3.535 | -7.05162 | 372 | 1 | top1 | 0.0085 | 0.017 | 7.05162 | 1.77E-12 | 1.06E-09 |
| ENSG00000136937.12 | 9 | 97633625 | 97633626 | 0.046412 | rs7850258 | 21.059 | rs10983035 | 0.000676 | 3.223 | -5.43322 | 400 | 24 | enet | 0.0097 | 0.011 | -5.40413 | 6.51E-08 | 1.42E-05 |
| ENSG00000136936.10 | 9 | 97697356 | 97697357 | 0.026584 | rs7850258 | 21.059 | rs4743112 | -0.00073 | 2.962 | -3.69238 | 437 | 437 | susie | 0.0029 | 0.11 | -8.42653 | 3.56E-17 | 5.74E-14 |
| ENSG00000136932.13 | 9 | 97922569 | 97922570 | 0.073892 | rs7850258 | 21.059 | rs907577 | 0.0245 | -5.151 | 16.67775 | 498 | 21 | enet | 0.055 | 1.40E-08 | -13.20461 | 8.25E-40 | 6.65E-36 |
| ENSG00000136938.8 | 9 | 97983360 | 97983361 | 0.034026 | rs7850258 | 21.059 | rs1010777 | 0.0105 | 4.312 | 7.78896 | 519 | 1 | top1 | 0.011 | 0.0087 | 7.78896 | 6.76E-15 | 6.05E-12 |
| ENSG00000106785.14 | 9 | 98119211 | 98119212 | 0.325083 | rs7850258 | 21.059 | rs942165 | 0.181 | -10.113 | -1.65086 | 575 | 13 | enet | 0.24 | 6.20E-35 | 3.46593 | 5.28E-04 | 2.06E-02 |
| ENSG00000119514.6 | 9 | 98807698 | 98807699 | 0.005588 | rs3780620 | -4.534 | rs2779523 | 0.00124 | 2.908 | 0.00231 | 585 | 585 | susie | 0.0036 | 0.083 | -3.46866 | 5.23E-04 | 2.05E-02 |
| ENSG00000106799.12 | 9 | 99104037 | 99104038 | 0.179942 | rs3780620 | -4.534 | rs334367 | 0.126 | -8.692 | -2.59433 | 452 | 8 | lasso | 0.13 | 4.20E-19 | 3.24601 | 1.17E-03 | 3.61E-02 |
| ENSG00000136874.10 | 9 | 99906632 | 99906633 | 0.091174 | rs3813712 | 4.004 | rs2416935 | 0.0738 | 7.232 | -2.89489 | 270 | 20 | enet | 0.081 | 4.40E-12 | -3.31744 | 9.08E-04 | 3.03E-02 |
| ENSG00000226752.9 | 9 | 120824827 | 120824828 | 0.829111 | rs1060817 | -4.769 | rs12343516 | 0.788 | 21.061 | -4.75149 | 354 | 354 | susie | 0.81 | 3.60E-204 | -4.73656 | 2.17E-06 | 2.52E-04 |
| ENSG00000095261.13 | 9 | 120842983 | 120842984 | 0.04301 | rs1060817 | -4.769 | rs7037673 | 0.0133 | -4.709 | 3.53442 | 368 | 8 | enet | 0.027 | 5.10E-05 | -5.04105 | 4.63E-07 | 7.54E-05 |
| ENSG00000119403.13 | 9 | 120894895 | 120894896 | 0.026065 | rs1060817 | -4.769 | rs11794516 | 0.0128 | -4.211 | -3.83711 | 357 | 10 | enet | 0.02 | 0.00046 | 4.84564 | 1.26E-06 | 1.61E-04 |
| ENSG00000119397.16 | 9 | 121074862 | 121074863 | 0.050438 | rs1060817 | -4.769 | rs2209076 | 0.0612 | 6.066 | 2.7366 | 380 | 9 | lasso | 0.063 | 1.10E-09 | 3.27513 | 1.06E-03 | 3.34E-02 |
| ENSG00000119396.10 | 9 | 121223013 | 121223014 | 0.006743 | rs1060817 | -4.769 | rs11794516 | 0.0054 | 2.843 | -3.83711 | 417 | 3 | lasso | 0.012 | 0.0048 | -4.25416 | 2.10E-05 | 1.68E-03 |
| ENSG00000197694.15 | 9 | 128552557 | 128552558 | 0.0211 | rs9697210 | 5.848 | rs2417126 | 0.00156 | 2.672 | 5.30843 | 276 | 1 | top1 | 0.0016 | 0.17 | 5.30843 | 1.11E-07 | 2.24E-05 |
| ENSG00000160447.6 | 9 | 128702522 | 128702523 | 0.646805 | rs9697210 | 5.848 | rs13289095 | 0.352 | 14.091 | 5.81863 | 275 | 27 | enet | 0.37 | 3.50E-57 | 5.48815 | 4.06E-08 | 9.62E-06 |
| ENSG00000167136.6 | 9 | 128818473 | 128818474 | 0.088942 | rs9697210 | 5.848 | rs2759009 | 0.05 | -5.935 | 4.33022 | 313 | 313 | susie | 0.054 | 1.50E-08 | -4.41529 | 1.01E-05 | 9.20E-04 |
| ENSG00000198917.12 | 9 | 128829820 | 128829821 | 0.090688 | rs9697210 | 5.848 | rs2997922 | 0.0649 | -6.41 | 4.56247 | 318 | 1 | top1 | 0.065 | 6.10E-10 | -4.56247 | 5.06E-06 | 5.07E-04 |
| ENSG00000165699.13 | 9 | 132944562 | 132944563 | 0.01658 | rs1073123 | 3.288 | rs6597586 | -0.00151 | -3.229 | 3.27986 | 543 | 1 | lasso | 0.0036 | 0.084 | -3.27986 | 1.04E-03 | 3.30E-02 |
| ENSG00000065675.14 | 10 | 6580300 | 6580301 | 0.027983 | rs744254 | 7.23 | rs755627 | 0.00283 | -3.85 | 3.2366 | 782 | 1 | top1 | 0.0028 | 0.11 | -3.2366 | 1.21E-03 | 3.69E-02 |
| ENSG00000230322.1 | 10 | 11171375 | 11171376 | -0.028639 | rs4114417 | -3.19 | rs4114417 | 0.00547 | -2.7 | -3.18977 | 587 | 1 | top1 | 0.0055 | 0.044 | 3.18977 | 1.42E-03 | 4.06E-02 |
| ENSG00000260314.2 | 10 | 17809343 | 17809344 | 0.061855 | rs2272146 | 5.15 | rs12570539 | -0.000235 | 3.31 | -0.865 | 536 | 30 | enet | 0.0083 | 0.018 | -3.62593 | 2.88E-04 | 1.30E-02 |
| ENSG00000148450.12 | 10 | 23095505 | 23095506 | 0.086637 | rs4237360 | 3.29 | rs6482253 | 0.0179 | -4.77 | -1.5388 | 359 | 25 | enet | 0.029 | 3.00E-05 | 3.25611 | 1.13E-03 | 3.52E-02 |
| ENSG00000182010.10 | 10 | 62268706 | 62268707 | 0.143301 | rs4948293 | -10.89 | rs7092005 | 0.079 | 7.63 | -4.99684 | 452 | 30 | enet | 0.097 | 3.60E-14 | -5.74586 | 9.15E-09 | 2.54E-06 |
| ENSG00000214688.5 | 10 | 71737823 | 71737824 | 0.098563 | rs9415996 | 4.15 | rs3747868 | 0.0298 | -4.34 | -3.86059 | 628 | 1 | top1 | 0.03 | 2.50E-05 | 3.86059 | 1.13E-04 | 6.37E-03 |
| ENSG00000197746.13 | 10 | 71851374 | 71851375 | 0.053701 | rs9415996 | 4.15 | rs7869 | 0.0439 | 5.36 | -3.31671 | 577 | 1 | top1 | 0.044 | 3.60E-07 | -3.31671 | 9.11E-04 | 3.03E-02 |
| ENSG00000165644.10 | 10 | 75236029 | 75236030 | 0.024614 | rs6480771 | -3.65 | rs1259498 | 0.00372 | 3.56 | -2.69435 | 341 | 341 | susie | 0.0061 | 0.036 | -3.63382 | 2.79E-04 | 1.28E-02 |
| ENSG00000148606.12 | 10 | 78029544 | 78029545 | -0.016828 | rs12415679 | 3.36 | rs4979982 | -0.000426 | -2.88 | 3.30165 | 521 | 1 | lasso | 0.0017 | 0.16 | -3.30165 | 9.61E-04 | 3.15E-02 |
| ENSG00000108179.13 | 10 | 79347468 | 79347469 | 0.067412 | rs1250550 | -5.33 | rs1250557 | 0.00471 | -4.33 | 2.91595 | 395 | 395 | susie | 0.02 | 0.00044 | -3.22866 | 1.24E-03 | 3.73E-02 |
| ENSG00000122852.14 | 10 | 79610938 | 79610939 | 0.011322 | rs1250550 | -5.33 | rs2802362 | -0.00176 | -2.78 | -3.6958 | 337 | 1 | lasso | 0.009 | 0.014 | 3.6958 | 2.19E-04 | 1.08E-02 |
| ENSG00000026103.21 | 10 | 88990581 | 88990582 | 0.139604 | rs4406737 | -4.76 | rs3740286 | 0.0803 | 7.08 | 3.90877 | 566 | 1 | top1 | 0.08 | 5.50E-12 | 3.90877 | 9.28E-05 | 5.55E-03 |
| ENSG00000107796.13 | 10 | 88990958 | 88990959 | 0.357199 | rs4406737 | -4.76 | rs7097572 | 0.262 | -12.19 | -4.5798 | 566 | 1 | top1 | 0.26 | 8.30E-39 | 4.5798 | 4.65E-06 | 4.71E-04 |
| ENSG00000107679.14 | 10 | 122374695 | 122374696 | 0.130052 | rs3850765 | 6.96 | rs11816578 | 0.0674 | 6.64 | 3.16295 | 614 | 614 | susie | 0.077 | 1.60E-11 | 3.44206 | 5.77E-04 | 2.20E-02 |
| ENSG00000166033.11 | 10 | 122461524 | 122461525 | 0.059841 | rs3850765 | 6.96 | rs4627016 | 0.00448 | -3.43 | 3.7436 | 614 | 614 | susie | 0.0066 | 0.031 | -4.23622 | 2.27E-05 | 1.78E-03 |
| ENSG00000230724.9 | 11 | 139611 | 139612 | 0.010772 | rs7936397 | 5.9 | rs11821341 | -0.00172 | 2.77 | 4.81 | 244 | 1 | lasso | 0.0032 | 0.095 | 4.814433 | 1.48E-06 | 1.83E-04 |
| ENSG00000254910.1 | 11 | 311140 | 311141 | 0.558902 | rs7936397 | 5.9 | rs3809112 | 0.189 | -10.33 | 4.89 | 324 | 13 | lasso | 0.27 | 8.10E-41 | -4.594582 | 4.34E-06 | 4.46E-04 |
| ENSG00000251661.3 | 11 | 318639 | 318640 | 0.523392 | rs7936397 | 5.9 | rs3809112 | 0.183 | 10.15 | 4.89 | 329 | 39 | enet | 0.26 | 1.10E-38 | 4.80155 | 1.57E-06 | 1.93E-04 |
| ENSG00000142089.15 | 11 | 321339 | 321340 | 0.648328 | rs7936397 | 5.9 | rs7102856 | 0.177 | 10.78 | 0.263 | 329 | 329 | susie | 0.41 | 4.40E-65 | 3.639692 | 2.73E-04 | 1.27E-02 |
| ENSG00000255328.1 | 11 | 327170 | 327171 | 0.833693 | rs7936397 | 5.9 | rs7395319 | 0.165 | -10.56 | -2.81 | 332 | 31 | enet | 0.55 | 2.00E-97 | 3.416755 | 6.34E-04 | 2.34E-02 |
| ENSG00000185101.12 | 11 | 442010 | 442011 | 0.115827 | rs7936397 | 5.9 | rs6421975 | 0.0558 | 7.04 | 2.62 | 382 | 24 | enet | 0.11 | 2.10E-16 | 3.280879 | 1.03E-03 | 3.29E-02 |
| ENSG00000174915.11 | 11 | 448267 | 448268 | 0.055563 | rs7936397 | 5.9 | rs12806062 | 0.0404 | -5.28 | 3.04 | 387 | 387 | susie | 0.042 | 6.10E-07 | -3.23588 | 1.21E-03 | 3.69E-02 |
| ENSG00000254815.5 | 11 | 557640 | 557641 | 0.043477 | rs7936397 | 5.9 | rs2246614 | 0.0107 | 3.88 | 4.48 | 436 | 6 | enet | 0.019 | 0.00065 | 4.305776 | 1.66E-05 | 1.41E-03 |
| ENSG00000099849.14 | 11 | 560403 | 560404 | 0.076247 | rs7936397 | 5.9 | rs12277611 | 0.00297 | 4.27 | 3.61 | 436 | 4 | enet | 0.0072 | 0.025 | 3.908377 | 9.29E-05 | 5.55E-03 |
| ENSG00000185507.19 | 11 | 615998 | 615999 | 0.023209 | rs7936397 | 5.9 | rs6598008 | 0.0091 | -3.79 | 4.7 | 464 | 2 | lasso | 0.021 | 0.00037 | -4.831498 | 1.36E-06 | 1.71E-04 |
| ENSG00000069696.6 | 11 | 637292 | 637293 | 0.087244 | rs7936397 | 5.9 | rs2246614 | 0.00448 | -3.67 | 4.48 | 470 | 9 | enet | 0.01 | 0.0092 | -3.384717 | 7.13E-04 | 2.51E-02 |
| ENSG00000177042.14 | 11 | 695532 | 695533 | 0.379908 | rs7936397 | 5.9 | rs11246262 | 0.209 | -10.85 | -2.58 | 479 | 33 | enet | 0.27 | 3.90E-40 | 3.830568 | 1.28E-04 | 7.14E-03 |
| ENSG00000026508.17 | 11 | 35138869 | 35138870 | 0.067591 | rs736374 | -8.85 | rs41352148 | 0.0209 | 5.62 | 6.5 | 674 | 13 | lasso | 0.031 | 1.70E-05 | 6.348472 | 2.17E-10 | 8.95E-08 |
| ENSG00000180423.4 | 11 | 46617908 | 46617909 | 0.005545 | rs10838610 | -3.44 | rs35619591 | 0.0013 | 2.5 | 2.25 | 235 | 235 | susie | 0.0036 | 0.084 | -3.73634 | 1.87E-04 | 9.54E-03 |
| ENSG00000134571.10 | 11 | 47352701 | 47352702 | 0.342118 | rs11605672 | -5.11 | rs10838698 | 0.143 | -9.07 | -4.85 | 296 | 296 | susie | 0.21 | 7.30E-30 | 4.946271 | 7.56E-07 | 1.11E-04 |
| ENSG00000172247.3 | 11 | 47594658 | 47594659 | 0.137111 | rs11605672 | -5.11 | rs4752856 | 0.191 | -10.36 | -4.29 | 286 | 1 | top1 | 0.19 | 1.40E-27 | 4.286244 | 1.82E-05 | 1.50E-03 |
| ENSG00000240371.1 | 11 | 57577079 | 57577080 | 0.010354 | rs4477456 | 3.88 | rs96894 | 0.0156 | 3.45 | -3.45 | 380 | 1 | top1 | 0.016 | 0.0018 | -3.448898 | 5.63E-04 | 2.16E-02 |
| ENSG00000240823.3 | 11 | 61444148 | 61444149 | 0.005868 | rs174574 | 6.47 | rs968567 | 0.00265 | -2.85 | 4.68 | 352 | 1 | lasso | 0.014 | 0.0027 | -4.678773 | 2.89E-06 | 3.13E-04 |
| ENSG00000134825.15 | 11 | 61792635 | 61792636 | 0.043163 | rs174574 | 6.47 | rs174538 | 0.0358 | 5.53 | 6.16 | 401 | 6 | enet | 0.044 | 3.70E-07 | 6.064884 | 1.32E-09 | 4.17E-07 |
| ENSG00000134824.13 | 11 | 61816255 | 61816256 | 0.703445 | rs174574 | 6.47 | rs968567 | 0.619 | 18.57 | 4.68 | 414 | 6 | lasso | 0.63 | 5.70E-121 | 5.135758 | 2.81E-07 | 4.80E-05 |
| ENSG00000149485.18 | 11 | 61829317 | 61829318 | 0.050543 | rs174574 | 6.47 | rs102275 | 0.0502 | 5.61 | 6.31 | 421 | 3 | lasso | 0.06 | 2.90E-09 | 6.270907 | 3.59E-10 | 1.35E-07 |
| ENSG00000149761.8 | 11 | 64225940 | 64225941 | 0.01133 | rs479777 | 6.4 | rs4930155 | 0.0023 | 3.31 | 5.81 | 343 | 1 | top1 | 0.0023 | 0.13 | 5.810576 | 6.23E-09 | 1.79E-06 |
| ENSG00000149782.11 | 11 | 64251522 | 64251523 | 0.076561 | rs479777 | 6.4 | rs11231740 | 0.00184 | -3.98 | -3.01 | 342 | 342 | susie | 0.015 | 0.0025 | 5.187614 | 2.13E-07 | 3.81E-05 |
| ENSG00000173264.14 | 11 | 64270337 | 64270338 | 0.059436 | rs479777 | 6.4 | rs3782101 | 0.0141 | 4.21 | -5.26 | 349 | 1 | top1 | 0.014 | 0.0029 | -5.260809 | 1.43E-07 | 2.78E-05 |
| ENSG00000173113.6 | 11 | 64318083 | 64318084 | 0.181641 | rs479777 | 6.4 | rs28395880 | 0.118 | -8.8 | -3.37 | 358 | 358 | susie | 0.13 | 3.10E-18 | 3.364634 | 7.66E-04 | 2.69E-02 |
| ENSG00000236935.1 | 11 | 64329503 | 64329504 | 0.491178 | rs479777 | 6.4 | rs479777 | 0.421 | 15.65 | 6.4 | 353 | 16 | enet | 0.47 | 4.20E-78 | 6.704832 | 2.02E-11 | 1.08E-08 |
| ENSG00000168071.21 | 11 | 64340222 | 64340223 | 0.049638 | rs479777 | 6.4 | rs645078 | 0.0203 | -4.71 | 6.01 | 353 | 353 | susie | 0.03 | 2.20E-05 | -6.348045 | 2.18E-10 | 8.95E-08 |
| ENSG00000173825.6 | 11 | 65354766 | 65354767 | 0.071554 | rs7107912 | 3.58 | rs2904980 | 0.0301 | 4.67 | 3.11 | 341 | 1 | top1 | 0.03 | 2.30E-05 | 3.106971 | 1.89E-03 | 4.93E-02 |
| ENSG00000172732.11 | 11 | 65860061 | 65860062 | 0.062637 | rs552130 | 4.17 | rs659857 | 0.0306 | -5.16 | 3.31 | 351 | 351 | susie | 0.043 | 4.10E-07 | -3.330464 | 8.67E-04 | 2.94E-02 |
| ENSG00000172757.12 | 11 | 65861945 | 65861946 | 0.040562 | rs552130 | 4.17 | rs13817 | 0.0141 | -4.65 | 3.33 | 351 | 1 | top1 | 0.014 | 0.0029 | -3.329025 | 8.72E-04 | 2.94E-02 |
| ENSG00000175334.7 | 11 | 66002176 | 66002177 | 0.104081 | rs552130 | 4.17 | rs747526 | 0.056 | 6.68 | -3.9 | 347 | 21 | enet | 0.086 | 1.00E-12 | -4.45337 | 8.45E-06 | 7.87E-04 |
| ENSG00000166435.15 | 11 | 74949199 | 74949200 | 0.682858 | rs10899060 | -3.56 | rs2119028 | 0.695 | 19.69 | -3.5 | 456 | 1 | top1 | 0.69 | 1.20E-145 | -3.504737 | 4.57E-04 | 1.84E-02 |
| ENSG00000118363.11 | 11 | 74949246 | 74949247 | 0.043926 | rs10899060 | -3.56 | rs1433771 | 0.0147 | 4.71 | -2.78 | 456 | 456 | susie | 0.016 | 0.0016 | -3.431811 | 6.00E-04 | 2.24E-02 |
| ENSG00000254810.1 | 11 | 76656711 | 76656712 | 0.206012 | rs4944115 | 4.29 | rs11236839 | 0.129 | -8.68 | 4.07 | 476 | 2 | lasso | 0.13 | 1.00E-18 | -3.905178 | 9.42E-05 | 5.58E-03 |
| ENSG00000137507.11 | 11 | 76670746 | 76670747 | 0.094733 | rs4944115 | 4.29 | rs11236839 | 0.0218 | -4.71 | 4.07 | 480 | 28 | enet | 0.03 | 2.40E-05 | -3.958547 | 7.54E-05 | 4.73E-03 |
| ENSG00000227376.1 | 11 | 77735025 | 77735026 | 0.018003 | rs606732 | -3.34 | rs530588 | 0.00447 | -2.9 | -3.28 | 352 | 1 | lasso | 0.0052 | 0.049 | 3.284011 | 1.02E-03 | 3.27E-02 |
| ENSG00000279696.1 | 11 | 93729804 | 93729805 | 0.028457 | rs12793348 | 5.93 | rs6483293 | 0.0142 | -3.67 | 5.03 | 412 | 1 | top1 | 0.014 | 0.0027 | -5.030831 | 4.88E-07 | 7.71E-05 |
| ENSG00000110218.8 | 11 | 94128927 | 94128928 | 0.229581 | rs12793348 | 5.93 | rs4073612 | 0.178 | -10.39 | 4.13 | 418 | 21 | enet | 0.22 | 9.50E-33 | -5.46595 | 4.60E-08 | 1.06E-05 |
| ENSG00000245552.6 | 11 | 95150538 | 95150539 | -0.032492 | rs4409785 | -11.6 | rs10466336 | 0.00615 | 2.39 | -4.4 | 518 | 1 | top1 | 0.0061 | 0.035 | -4.395455 | 1.11E-05 | 9.88E-04 |
| ENSG00000235505.7 | 11 | 104919072 | 104919073 | 0.088928 | rs1834631 | -3.82 | rs528641 | 0.0357 | -5.42 | 2.28 | 335 | 10 | lasso | 0.062 | 1.40E-09 | -3.320431 | 8.99E-04 | 3.01E-02 |
| ENSG00000150764.13 | 11 | 111927143 | 111927144 | 0.139359 | rs11214125 | -3.2 | rs623491 | 0.0147 | -4.75 | 2.82 | 338 | 33 | enet | 0.094 | 8.80E-14 | -3.308171 | 9.39E-04 | 3.11E-02 |
| ENSG00000160588.9 | 11 | 118252349 | 118252350 | 0.167617 | rs494459 | 4.16 | rs10790248 | 0.141 | -9.53 | 3.11 | 538 | 1 | top1 | 0.14 | 2.50E-20 | -3.111072 | 1.86E-03 | 4.89E-02 |
| ENSG00000149573.8 | 11 | 118264535 | 118264536 | 0.136666 | rs494459 | 4.16 | rs1056562 | 0.149 | -9.19 | 3.1 | 536 | 1 | top1 | 0.15 | 1.80E-21 | -3.102 | 1.92E-03 | 4.98E-02 |
| ENSG00000019144.18 | 11 | 118606439 | 118606440 | 0.008648 | rs494459 | 4.16 | rs489126 | -0.000592 | 2.89 | 3.94 | 427 | 2 | lasso | 0.0078 | 0.021 | 3.942609 | 8.06E-05 | 4.95E-03 |
| ENSG00000255422.1 | 11 | 118704606 | 118704607 | 0.516158 | rs494459 | 4.16 | rs573905 | 0.426 | 15.43 | -2.82 | 381 | 381 | susie | 0.5 | 6.40E-86 | -3.632929 | 2.80E-04 | 1.29E-02 |
| ENSG00000278376.1 | 11 | 118791253 | 118791254 | 0.144058 | rs494459 | 4.16 | rs636736 | 0.0555 | 6.95 | -2.5 | 363 | 16 | enet | 0.094 | 7.10E-14 | -3.657281 | 2.55E-04 | 1.21E-02 |
| ENSG00000186174.12 | 11 | 118925607 | 118925608 | 0.011199 | rs494459 | 4.16 | rs10892301 | -0.00145 | 3.07 | -3.51 | 370 | 2 | lasso | -0.0014 | 0.62 | -3.693915 | 2.21E-04 | 1.09E-02 |
| ENSG00000256673.1 | 12 | 9414850 | 9414851 | -0.007089 | rs7313141 | -3.64 | rs7313141 | 0.00367 | -2.45 | -3.64 | 32 | 1 | top1 | 0.0037 | 0.081 | 3.64 | 2.75E-04 | 1.27E-02 |
| ENSG00000151233.10 | 12 | 42144878 | 42144879 | 0.098885 | rs712119 | -3.98 | rs2215454 | 0.036 | 5.5 | -3.03 | 407 | 407 | susie | 0.04 | 1.10E-06 | -3.32 | 9.16E-04 | 3.04E-02 |
| ENSG00000139168.7 | 12 | 42326117 | 42326118 | 0.019538 | rs712119 | -3.98 | rs1059360 | -0.000637 | -3.18 | 3.46 | 427 | 427 | susie | 0.00021 | 0.29 | -3.76 | 1.70E-04 | 8.84E-03 |
| ENSG00000167535.7 | 12 | 48814431 | 48814432 | 0.295618 | rs12369114 | -3.52 | rs12369114 | 0.209 | 10.94 | -3.52 | 345 | 1 | top1 | 0.21 | 2.20E-30 | -3.52 | 4.30E-04 | 1.75E-02 |
| ENSG00000257913.2 | 12 | 48998666 | 48998667 | 0.023528 | rs12369114 | -3.52 | rs11614738 | -0.000621 | 3.41 | -3.34 | 323 | 323 | susie | 0.00086 | 0.22 | -3.35 | 8.05E-04 | 2.81E-02 |
| ENSG00000178449.8 | 12 | 50111978 | 50111979 | 0.083911 | rs11169493 | 3.41 | rs7972465 | 0.0487 | 6.09 | -2.23 | 308 | 13 | enet | 0.071 | 8.70E-11 | -3.11 | 1.85E-03 | 4.87E-02 |
| ENSG00000161813.21 | 12 | 50392382 | 50392383 | 0.010965 | rs17210898 | 3.65 | rs4768914 | -0.000137 | -3.18 | 3.33 | 308 | 1 | top1 | -0.00014 | 0.34 | -3.33 | 8.69E-04 | 2.94E-02 |
| ENSG00000139651.10 | 12 | 53180699 | 53180700 | 0.11867 | rs2280446 | 4.63 | rs12581102 | 0.0594 | 6.43 | 2.78 | 476 | 12 | enet | 0.072 | 6.10E-11 | 3.24 | 1.19E-03 | 3.65E-02 |
| ENSG00000135414.9 | 12 | 55743279 | 55743280 | -0.000552 | rs705702 | -7.66 | rs11171747 | -0.00119 | 2.74 | 5.28 | 308 | 2 | lasso | 0.0017 | 0.16 | 5.9 | 3.59E-09 | 1.05E-06 |
| ENSG00000185664.14 | 12 | 55973316 | 55973317 | 0.021862 | rs705702 | -7.66 | rs10876862 | 0.00141 | 2.93 | 3.92 | 292 | 1 | top1 | 0.0014 | 0.18 | 3.92 | 8.99E-05 | 5.46E-03 |
| ENSG00000139531.12 | 12 | 55997179 | 55997180 | 0.216061 | rs705702 | -7.66 | rs705700 | 0.179 | -10.23 | -7.29 | 293 | 45 | enet | 0.19 | 6.20E-27 | 7.34 | 2.09E-13 | 1.58E-10 |
| ENSG00000197728.9 | 12 | 56041852 | 56041853 | 0.597246 | rs705702 | -7.66 | rs10876864 | 0.669 | 19.31 | -7.14 | 290 | 17 | enet | 0.68 | 3.40E-141 | -7.66 | 1.80E-14 | 1.53E-11 |
| ENSG00000065361.14 | 12 | 56079856 | 56079857 | 0.023703 | rs705702 | -7.66 | rs705699 | -0.0014 | -3.2 | -7.33 | 292 | 292 | susie | -0.00056 | 0.41 | 4.52 | 6.14E-06 | 5.93E-04 |
| ENSG00000170515.13 | 12 | 56104318 | 56104319 | 0.016935 | rs705702 | -7.66 | rs2069391 | -0.000759 | 3.01 | -1.78 | 290 | 6 | enet | -0.00011 | 0.33 | -4.69 | 2.76E-06 | 3.03E-04 |
| ENSG00000135482.6 | 12 | 56118158 | 56118159 | 0.03218 | rs705702 | -7.66 | rs10876840 | 0.00751 | 3.38 | 0.0399 | 295 | 3 | lasso | 0.011 | 0.0074 | 4.62 | 3.87E-06 | 4.05E-04 |
| ENSG00000139641.12 | 12 | 56128055 | 56128056 | 0.041872 | rs705702 | -7.66 | rs11171710 | -0.00167 | -2.78 | 6.69 | 299 | 299 | susie | 0.0075 | 0.023 | -4.75 | 2.06E-06 | 2.41E-04 |
| ENSG00000110958.15 | 12 | 56688407 | 56688408 | 0.024378 | rs4759017 | -4.22 | rs2950387 | 0.0164 | 4.23 | -3.21 | 320 | 1 | top1 | 0.016 | 0.0014 | -3.21 | 1.32E-03 | 3.88E-02 |
| ENSG00000182199.10 | 12 | 57229326 | 57229327 | 0.00748 | rs11172254 | 4.03 | rs7313599 | 0.00326 | 2.72 | -3.3 | 340 | 1 | top1 | 0.0033 | 0.094 | -3.3 | 9.67E-04 | 3.16E-02 |
| ENSG00000185482.7 | 12 | 57251192 | 57251193 | 0.023331 | rs11172254 | 4.03 | rs10876968 | 0.0143 | -4.06 | 3.47 | 343 | 1 | lasso | 0.02 | 0.00041 | -3.47 | 5.15E-04 | 2.02E-02 |
| ENSG00000139269.2 | 12 | 57452322 | 57452323 | 0.021853 | rs11172254 | 4.03 | rs11609986 | -0.000301 | -2.99 | 2.95 | 335 | 335 | susie | 0.00083 | 0.23 | -3.22 | 1.29E-03 | 3.84E-02 |
| ENSG00000166908.17 | 12 | 57591173 | 57591174 | 0.041016 | rs11172254 | 4.03 | rs7313599 | 0.0409 | 5.01 | -3.3 | 338 | 7 | enet | 0.048 | 1.00E-07 | -3.3 | 9.54E-04 | 3.14E-02 |
| ENSG00000184575.11 | 12 | 64404349 | 64404350 | 0.014439 | rs12319677 | 3.63 | rs6581561 | 0.000581 | -3.35 | 3.56 | 307 | 1 | top1 | 0.00058 | 0.25 | -3.56 | 3.76E-04 | 1.59E-02 |
| ENSG00000257802.1 | 12 | 71850427 | 71850428 | 0.007266 | rs1292516 | -3.43 | rs1292516 | 0.000853 | 2.78 | -3.43 | 417 | 1 | lasso | 0.0022 | 0.14 | -3.43 | 6.04E-04 | 2.25E-02 |
| ENSG00000258035.1 | 12 | 94186043 | 94186044 | 0.006954 | rs3847800 | 5.56 | rs11107181 | -0.0018 | -2.92 | -1.61 | 616 | 616 | susie | 1.00E-05 | 0.32 | 3.66 | 2.55E-04 | 1.21E-02 |
| ENSG00000076513.16 | 12 | 109999185 | 109999186 | 0.047905 | rs11064860 | 5.29 | rs11829777 | 0.00281 | -3.84 | 4.46 | 300 | 300 | susie | 0.015 | 0.0021 | -3.39 | 6.96E-04 | 2.49E-02 |
| ENSG00000196510.12 | 12 | 110403729 | 110403730 | 0.058207 | rs11064860 | 5.29 | rs13313219 | -0.00158 | -3.26 | 3.25 | 228 | 228 | susie | 0.0052 | 0.049 | -4.66 | 3.19E-06 | 3.38E-04 |
| ENSG00000196850.5 | 12 | 110583319 | 110583320 | 0.000398 | rs4378452 | -11.73 | rs1011300 | -0.0018 | -3.07 | 3.52 | 212 | 212 | susie | 0.0013 | 0.19 | -4.56 | 5.12E-06 | 5.09E-04 |
| ENSG00000122986.13 | 12 | 110704949 | 110704950 | 0.040928 | rs11065822 | -13 | rs6606683 | 0.0199 | -4.74 | 4 | 208 | 208 | susie | 0.02 | 0.00046 | -4.07 | 4.80E-05 | 3.22E-03 |
| ENSG00000198324.13 | 12 | 111369120 | 111369121 | 0.020315 | rs3184504 | -22.92 | rs3847953 | 0.00317 | 3.76 | 10.8 | 233 | 233 | susie | 0.0042 | 0.069 | 11 | 3.55E-28 | 8.17E-25 |
| ENSG00000257595.2 | 12 | 111369281 | 111369282 | 0.001118 | rs3184504 | -22.92 | rs3742004 | -0.00177 | 2.19 | 8.23 | 233 | 233 | susie | -0.00097 | 0.5 | 12.6 | 2.71E-36 | 1.46E-32 |
| ENSG00000111252.10 | 12 | 111405947 | 111405948 | 0.006092 | rs3184504 | -22.92 | rs4766453 | -0.00179 | -3.03 | 7.91 | 241 | 1 | lasso | 7.30E-05 | 0.31 | -7.91 | 2.52E-15 | 2.71E-12 |
| ENSG00000111275.12 | 12 | 111766886 | 111766887 | 0.137107 | rs3184504 | -22.92 | rs2238151 | 0.0467 | 5.43 | 12.4 | 222 | 222 | susie | 0.049 | 7.00E-08 | 10.9 | 1.26E-27 | 2.54E-24 |
| ENSG00000234608.7 | 12 | 111842901 | 111842902 | 0.044293 | rs3184504 | -22.92 | rs7314870 | 0.0625 | 6.24 | 8.25 | 204 | 1 | top1 | 0.063 | 1.30E-09 | 8.25 | 1.57E-16 | 1.95E-13 |
| ENSG00000089022.13 | 12 | 111843102 | 111843103 | 0.027482 | rs3184504 | -22.92 | rs7114 | 0.00731 | -3.93 | 4.5 | 204 | 1 | top1 | 0.0073 | 0.024 | -4.5 | 6.78E-06 | 6.43E-04 |
| ENSG00000226469.1 | 12 | 111927017 | 111927018 | 0.020002 | rs3184504 | -22.92 | rs16941724 | 0.00575 | 3.33 | 4.07 | 191 | 1 | top1 | 0.0058 | 0.04 | 4.07 | 4.65E-05 | 3.16E-03 |
| ENSG00000198270.12 | 12 | 112013184 | 112013185 | 0.098459 | rs653178 | -22.75 | rs4766764 | 0.0903 | 7.4 | 4.5 | 196 | 1 | top1 | 0.09 | 2.50E-13 | 4.5 | 6.75E-06 | 6.43E-04 |
| ENSG00000111300.9 | 12 | 112109021 | 112109022 | 0.021739 | rs11065987 | -20.44 | rs11066118 | 0.013 | 3.88 | 4.58 | 203 | 203 | susie | 0.015 | 0.0019 | 6.2 | 5.71E-10 | 2.00E-07 |
| ENSG00000135148.11 | 12 | 112125500 | 112125501 | 0.177247 | rs11065987 | -20.44 | rs11611847 | 0.00789 | 3.7 | 0.135 | 209 | 9 | enet | 0.028 | 4.10E-05 | -6.7 | 2.15E-11 | 1.08E-08 |
| ENSG00000173064.12 | 12 | 112382438 | 112382439 | 0.008519 | rs17630235 | -19.68 | rs7315519 | 0.00388 | -2.99 | -8.41 | 278 | 1 | top1 | 0.0039 | 0.075 | 8.41 | 4.16E-17 | 6.10E-14 |
| ENSG00000089009.15 | 12 | 112411441 | 112411442 | -0.005706 | rs17630235 | -19.68 | rs232925 | -0.000891 | -2.18 | -8.39 | 283 | 1 | top1 | -0.00089 | 0.48 | 8.39 | 4.88E-17 | 6.55E-14 |
| ENSG00000179295.17 | 12 | 112418350 | 112418351 | 0.018415 | rs17630235 | -19.68 | rs12422941 | 0.00067 | 3.05 | 4.06 | 284 | 1 | lasso | 0.012 | 0.0054 | 4.06 | 4.97E-05 | 3.32E-03 |
| ENSG00000089127.12 | 12 | 112906776 | 112906777 | 0.123162 | rs11066320 | -19 | rs4766662 | 0.0875 | 7.41 | -3.64 | 412 | 412 | susie | 0.089 | 3.50E-13 | -3.64 | 2.71E-04 | 1.27E-02 |
| ENSG00000111335.12 | 12 | 112978394 | 112978395 | -0.005918 | rs2891403 | -12.08 | rs11066323 | 0.00506 | 3.14 | 6.38 | 418 | 1 | lasso | 0.0061 | 0.036 | 6.38 | 1.72E-10 | 7.70E-08 |
| ENSG00000123064.12 | 12 | 113185478 | 113185479 | 0.003143 | rs2891403 | -12.08 | rs2239193 | -0.000508 | -2.95 | -1.32 | 481 | 481 | susie | -0.00038 | 0.37 | -6.21 | 5.37E-10 | 1.97E-07 |
| ENSG00000167272.10 | 12 | 120581397 | 120581398 | 0.059568 | rs3213566 | -4.71 | rs12369178 | 0.0181 | -4.96 | -4.41 | 411 | 411 | susie | 0.034 | 6.60E-06 | 4.35 | 1.39E-05 | 1.22E-03 |
| ENSG00000122971.8 | 12 | 120725734 | 120725735 | 0.087359 | rs3213566 | -4.71 | rs3850521 | 0.0215 | -4.32 | 3.81 | 448 | 1 | top1 | 0.021 | 3.00E-04 | -3.81 | 1.41E-04 | 7.58E-03 |
| ENSG00000135124.14 | 12 | 121209856 | 121209857 | 0.36671 | rs3213566 | -4.71 | rs7298368 | 0.192 | 10.43 | 2.29 | 413 | 34 | enet | 0.29 | 3.20E-43 | 3.28 | 1.03E-03 | 3.29E-02 |
| ENSG00000256742.1 | 12 | 121581015 | 121581016 | -0.009611 | rs1169091 | 4.39 | rs3892756 | -0.000601 | 2.3 | -3.23 | 393 | 1 | top1 | -6.00E-04 | 0.41 | -3.23 | 1.22E-03 | 3.69E-02 |
| ENSG00000110801.13 | 12 | 121888730 | 121888731 | 0.057662 | rs11611988 | -4.63 | rs7309105 | 0.0308 | -5.73 | 3.91 | 344 | 8 | lasso | 0.04 | 1.20E-06 | -3.49 | 4.78E-04 | 1.92E-02 |
| ENSG00000158023.9 | 12 | 121918297 | 121918298 | 0.03304 | rs11611988 | -4.63 | rs1169072 | 0.0123 | 4.1 | 3.19 | 343 | 2 | lasso | 0.013 | 0.0036 | 3.2 | 1.36E-03 | 3.95E-02 |
| ENSG00000158113.12 | 12 | 122167737 | 122167738 | 0.07452 | rs11611988 | -4.63 | rs7487608 | 0.0407 | 5.74 | -3.99 | 337 | 12 | enet | 0.064 | 8.70E-10 | -3.79 | 1.50E-04 | 7.95E-03 |
| ENSG00000130779.19 | 12 | 122422631 | 122422632 | 0.01774 | rs11611988 | -4.63 | rs10770186 | -0.00173 | 2.89 | 3.61 | 337 | 337 | susie | 0.0033 | 0.093 | 4.9 | 9.42E-07 | 1.24E-04 |
| ENSG00000111325.16 | 12 | 122974682 | 122974683 | 0.020841 | rs28533432 | -3.87 | rs7135296 | 3.00E-04 | 3.07 | -3.57 | 320 | 320 | susie | 0.0056 | 0.043 | -3.2 | 1.35E-03 | 3.93E-02 |
| ENSG00000182196.13 | 12 | 122981649 | 122981650 | 0.044933 | rs28533432 | -3.87 | rs4275659 | 0.0507 | -5.79 | -3.4 | 318 | 1 | top1 | 0.051 | 4.50E-08 | 3.4 | 6.81E-04 | 2.45E-02 |
| ENSG00000280120.1 | 12 | 123153376 | 123153377 | 0.007377 | rs28533432 | -3.87 | rs1568427 | 0.0063 | 2.8 | -3.39 | 330 | 1 | top1 | 0.0063 | 0.034 | -3.39 | 7.02E-04 | 2.49E-02 |
| ENSG00000183955.12 | 12 | 123383772 | 123383773 | 0.102736 | rs28533432 | -3.87 | rs11057276 | 0.0789 | 7.63 | -3.75 | 346 | 16 | enet | 0.097 | 3.00E-14 | -3.92 | 9.01E-05 | 5.46E-03 |
| ENSG00000133116.7 | 13 | 33016432 | 33016433 | 0.056665 | rs9595795 | 4.6 | rs9527023 | -0.00167 | -3.57 | 2.33912 | 529 | 18 | enet | 0.0098 | 0.011 | -3.7034 | 2.13E-04 | 1.06E-02 |
| ENSG00000150907.7 | 13 | 40666596 | 40666597 | 0.045825 | rs12865518 | -4.3 | rs2721047 | 0.000103 | 3.59 | 3.64876 | 438 | 438 | susie | 0.0066 | 0.031 | 3.20883 | 1.33E-03 | 3.88E-02 |
| ENSG00000168852.12 | 13 | 40921748 | 40921749 | 0.001798 | rs12865518 | -4.3 | rs9603758 | -0.000385 | 2.77 | -0.68413 | 325 | 325 | susie | 0.0044 | 0.063 | 3.15433 | 1.61E-03 | 4.41E-02 |
| ENSG00000118922.17 | 13 | 73995048 | 73995049 | 0.048389 | rs4885151 | 4.96 | rs4885151 | 0.00607 | -4.44 | 4.95587 | 585 | 18 | enet | 0.016 | 0.0014 | -5.51838 | 3.42E-08 | 8.23E-06 |
| ENSG00000226677.3 | 14 | 34939323 | 34939324 | 0.065331 | rs4982208 | 3.77 | rs1967723 | 0.0201 | -4.88 | 3.57115 | 378 | 13 | enet | 0.029 | 2.70E-05 | -3.59896 | 3.19E-04 | 1.40E-02 |
| ENSG00000174373.16 | 14 | 35809303 | 35809304 | 0.126605 | rs2415317 | -5.16 | rs10134646 | 0.172 | 9.98 | 3.26077 | 400 | 400 | susie | 0.18 | 2.50E-25 | 3.209 | 1.33E-03 | 3.88E-02 |
| ENSG00000176435.6 | 14 | 38256368 | 38256369 | 0.018441 | rs12434337 | 3.75 | rs10133926 | -0.00124 | 2.78 | -2.17648 | 390 | 390 | susie | 0.0017 | 0.16 | -3.69795 | 2.17E-04 | 1.07E-02 |
| ENSG00000178974.9 | 14 | 55271308 | 55271309 | 0.019743 | rs9323285 | -3.81 | rs11622636 | 0.00611 | 3.7 | -3.64614 | 376 | 1 | top1 | 0.0061 | 0.036 | -3.64614 | 2.66E-04 | 1.25E-02 |
| ENSG00000126775.8 | 14 | 55411857 | 55411858 | 0.013537 | rs9323285 | -3.81 | rs17128145 | -0.000545 | 3.3 | -2.70209 | 434 | 434 | susie | 0.00086 | 0.22 | -3.75093 | 1.76E-04 | 9.09E-03 |
| ENSG00000182185.18 | 14 | 67819778 | 67819779 | 0.001748 | rs3784099 | 6.55 | rs4902562 | -0.00156 | 2.95 | -4.27243 | 367 | 9 | enet | 0.00082 | 0.23 | -3.28068 | 1.04E-03 | 3.30E-02 |
| ENSG00000198208.11 | 14 | 74923395 | 74923396 | 0.094547 | rs10483863 | -4.33 | rs2159906 | 0.0646 | 6.45 | -3.56117 | 377 | 3 | lasso | 0.072 | 7.10E-11 | -3.50857 | 4.51E-04 | 1.82E-02 |
| ENSG00000119718.10 | 14 | 75002910 | 75002911 | 0.163887 | rs10483863 | -4.33 | rs175040 | 0.202 | -10.92 | 3.48212 | 413 | 413 | susie | 0.21 | 1.70E-30 | -3.39996 | 6.74E-04 | 2.43E-02 |
| ENSG00000119638.12 | 14 | 75127151 | 75127152 | 0.063568 | rs10483863 | -4.33 | rs175479 | 0.0567 | -6.03 | 3.31771 | 416 | 6 | lasso | 0.06 | 2.90E-09 | -3.38284 | 7.17E-04 | 2.52E-02 |
| ENSG00000165409.16 | 14 | 80954988 | 80954989 | 0.035357 | rs741651 | -4.38 | rs12437092 | 0.00489 | -3.72 | -3.65636 | 419 | 3 | lasso | 0.0077 | 0.021 | 3.80087 | 1.44E-04 | 7.71E-03 |
| ENSG00000184990.12 | 14 | 104753099 | 104753100 | 0.080025 | rs4465542 | -3.47 | rs4983386 | 0.0377 | -5.47 | 3.26032 | 368 | 368 | susie | 0.041 | 8.10E-07 | -3.29141 | 9.97E-04 | 3.23E-02 |
| ENSG00000166428.12 | 14 | 104924815 | 104924816 | 0.097599 | rs11620688 | 3.51 | rs10083490 | 0.048 | 6.14 | -3.40211 | 320 | 17 | enet | 0.059 | 3.20E-09 | -3.82794 | 1.29E-04 | 7.17E-03 |
| ENSG00000185567.6 | 14 | 104978356 | 104978357 | 0.00524 | rs11620688 | 3.77 | rs10083490 | -0.00155 | 2.8 | -3.40211 | 316 | 6 | lasso | 0.014 | 0.0034 | -3.4021 | 6.69E-04 | 2.42E-02 |
| ENSG00000185347.17 | 14 | 105489854 | 105489855 | 0.008811 | rs35590487 | 4.09 | rs35590487 | 0.000877 | -2.9 | 4.08525 | 152 | 1 | top1 | 0.00088 | 0.22 | -4.08525 | 4.40E-05 | 3.02E-03 |
| ENSG00000184986.10 | 14 | 105526602 | 105526603 | 0.170261 | rs35590487 | 4.09 | rs11624007 | 0.213 | -11.18 | 3.89275 | 129 | 129 | susie | 0.22 | 1.30E-31 | -3.87546 | 1.06E-04 | 6.15E-03 |
| ENSG00000253364.2 | 14 | 105649056 | 105649057 | 0.200806 | rs35590487 | 4.09 | rs35590487 | 0.0633 | -6.6 | 4.08525 | 90 | 90 | susie | 0.14 | 1.10E-19 | -3.59537 | 3.24E-04 | 1.41E-02 |
| ENSG00000211897.8 | 14 | 105771404 | 105771405 | 0.06631 | rs35590487 | 4.09 | rs10134526 | 0.0179 | -3.85 | -2.93388 | 42 | 8 | enet | 0.025 | 0.00011 | 4.11042 | 3.95E-05 | 2.77E-03 |
| ENSG00000211935.3 | 14 | 106005573 | 106005574 | 0.089403 | rs35590487 | 4.09 | rs10151805 | 0.0133 | -3.33 | 3.82109 | 21 | 21 | susie | 0.033 | 1.10E-05 | -3.95449 | 7.67E-05 | 4.78E-03 |
| ENSG00000128965.11 | 15 | 40952961 | 40952962 | 0.032206 | rs8042729 | 3.85 | rs17747351 | 0.00238 | 3.24 | 3.18586 | 268 | 268 | susie | 0.0084 | 0.017 | 3.424903 | 6.15E-04 | 2.27E-02 |
| ENSG00000137818.11 | 15 | 69452783 | 69452784 | 0.015171 | rs7176072 | -4.42 | rs17315418 | 0.0096 | 2.52 | -3.65069 | 453 | 1 | top1 | 0.0096 | 0.012 | -3.650689 | 2.62E-04 | 1.23E-02 |
| ENSG00000159556.9 | 15 | 76336723 | 76336724 | 0.34032 | rs2930681 | 3.39 | rs12591100 | 0.204 | 10.77 | -2.98502 | 270 | 270 | susie | 0.26 | 4.00E-38 | -3.233382 | 1.22E-03 | 3.69E-02 |
| ENSG00000259514.1 | 15 | 76340274 | 76340275 | 0.060806 | rs2930681 | 3.39 | rs12591100 | 0.0114 | 4.42 | -2.98502 | 269 | 269 | susie | 0.032 | 1.10E-05 | -3.431775 | 6.00E-04 | 2.24E-02 |
| ENSG00000140386.12 | 15 | 76905443 | 76905444 | 0.222946 | rs907593 | -3.52 | rs11072636 | 0.357 | 14.61 | -3.29503 | 232 | 9 | lasso | 0.38 | 8.20E-61 | -3.109778 | 1.87E-03 | 4.90E-02 |
| ENSG00000166965.12 | 15 | 90955795 | 90955796 | 0.131935 | rs3803563 | -3.39 | rs2290202 | 0.0937 | 7.71 | -3.23617 | 474 | 474 | susie | 0.098 | 2.20E-14 | -3.30299 | 9.57E-04 | 3.15E-02 |
| ENSG00000068305.17 | 15 | 99565416 | 99565417 | 0.006872 | rs4483828 | 3.19 | rs8026614 | 0.00534 | -4.18 | 2.85124 | 513 | 513 | susie | 0.012 | 0.0049 | -3.170845 | 1.52E-03 | 4.25E-02 |
| ENSG00000102854.15 | 16 | 760761 | 760762 | 0.033912 | rs9927150 | -3.86 | rs3764246 | 0.0188 | 4.18 | -2.1254 | 457 | 457 | susie | 0.019 | 0.00068 | -3.483 | 4.96E-04 | 1.96E-02 |
| ENSG00000127586.16 | 16 | 788045 | 788046 | 0.033074 | rs9927150 | -3.86 | rs3765264 | 0.00392 | -4.25 | -3.03527 | 451 | 3 | lasso | 0.012 | 0.0051 | 3.1865 | 1.44E-03 | 4.08E-02 |
| ENSG00000131634.13 | 16 | 1528687 | 1528688 | 0.463805 | rs1057610 | -3.84 | rs8048206 | 0.454 | 15.94 | -2.78177 | 488 | 488 | susie | 0.47 | 1.60E-79 | -3.2436 | 1.18E-03 | 3.63E-02 |
| ENSG00000007545.15 | 16 | 1612324 | 1612325 | 0.071265 | rs1057610 | -3.84 | rs9940288 | 0.0439 | 5.79 | -3.32083 | 471 | 471 | susie | 0.051 | 4.10E-08 | -3.1557 | 1.60E-03 | 4.40E-02 |
| ENSG00000140990.14 | 16 | 1959507 | 1959508 | 0.040487 | rs1057610 | -3.84 | rs397435 | 0.0475 | -5.68 | 3.54984 | 440 | 1 | top1 | 0.047 | 1.20E-07 | -3.5498 | 3.85E-04 | 1.60E-02 |
| ENSG00000140988.15 | 16 | 1964859 | 1964860 | 0.323657 | rs1057610 | -3.84 | rs397435 | 0.125 | 8.41 | 3.54984 | 439 | 439 | susie | 0.16 | 2.20E-22 | 3.5532 | 3.81E-04 | 1.59E-02 |
| ENSG00000167978.16 | 16 | 2752328 | 2752329 | 0.018577 | rs11077336 | -4.38 | rs3112726 | -0.00162 | 2.92 | -4.25452 | 313 | 16 | enet | 0.0033 | 0.093 | -4.0653 | 4.80E-05 | 3.22E-03 |
| ENSG00000162076.12 | 16 | 2883185 | 2883186 | 0.371391 | rs11077336 | -4.38 | rs9673618 | 0.29 | 12.74 | 4.01958 | 329 | 13 | enet | 0.31 | 1.40E-47 | 3.8179 | 1.35E-04 | 7.38E-03 |
| ENSG00000277369.1 | 16 | 11797467 | 11797468 | 0.021858 | rs7203803 | 3.94 | rs7199847 | -0.00179 | -3.38 | -2.68765 | 466 | 466 | susie | 0.0039 | 0.074 | 3.3053 | 9.49E-04 | 3.13E-02 |
| ENSG00000260592.1 | 16 | 19487898 | 19487899 | 0.055732 | rs7200119 | 4.41 | rs7200119 | 0.0423 | -5.02 | 4.41024 | 457 | 1 | top1 | 0.042 | 5.80E-07 | -4.4102 | 1.03E-05 | 9.33E-04 |
| ENSG00000188603.18 | 16 | 28495097 | 28495098 | 0.157786 | rs151228 | -4.2 | rs151228 | 0.107 | -7.79 | -4.20143 | 214 | 1 | top1 | 0.11 | 1.40E-15 | 4.2014 | 2.65E-05 | 1.98E-03 |
| ENSG00000184730.10 | 16 | 28495575 | 28495576 | 0.024324 | rs151228 | -4.2 | rs480400 | 0.00826 | -3.44 | -4.12925 | 213 | 1 | top1 | 0.0083 | 0.018 | 4.1293 | 3.64E-05 | 2.57E-03 |
| ENSG00000176476.8 | 16 | 28581054 | 28581055 | 0.241879 | rs151228 | -4.2 | rs151228 | 0.197 | -10.57 | -4.20143 | 187 | 187 | susie | 0.2 | 1.80E-29 | 4.329 | 1.50E-05 | 1.29E-03 |
| ENSG00000196502.11 | 16 | 28623624 | 28623625 | 0.519822 | rs151228 | -4.2 | rs480400 | 0.132 | -8.78 | -4.12925 | 182 | 40 | enet | 0.28 | 2.30E-41 | 3.9896 | 6.62E-05 | 4.25E-03 |
| ENSG00000251417.2 | 16 | 28802742 | 28802743 | 0.040373 | rs151228 | -4.2 | rs151227 | 0.00129 | 3.09 | -3.44281 | 182 | 1 | top1 | 0.0013 | 0.19 | -3.4428 | 5.76E-04 | 2.20E-02 |
| ENSG00000177548.12 | 16 | 28936510 | 28936511 | 0.02164 | rs151228 | -4.2 | rs181209 | 0.00926 | 3.55 | -3.73212 | 184 | 1 | top1 | 0.0093 | 0.013 | -3.7321 | 1.90E-04 | 9.63E-03 |
| ENSG00000183604.14 | 16 | 30335373 | 30335374 | 0.117498 | rs7204270 | 3.32 | rs9924308 | 0.107 | -8.18 | 3.2706 | 209 | 209 | susie | 0.11 | 9.30E-16 | -3.2155 | 1.30E-03 | 3.85E-02 |
| ENSG00000273724.1 | 16 | 30336399 | 30336400 | 0.068641 | rs7204270 | 3.32 | rs13331817 | 0.0355 | -5.43 | 3.06753 | 209 | 209 | susie | 0.04 | 1.20E-06 | -3.1496 | 1.63E-03 | 4.45E-02 |
| ENSG00000179965.11 | 16 | 30407296 | 30407297 | 0.031303 | rs7204270 | 3.32 | rs4787495 | 0.00582 | -3.58 | 2.32006 | 202 | 202 | susie | 0.0074 | 0.024 | -3.3298 | 8.69E-04 | 2.94E-02 |
| ENSG00000121281.12 | 16 | 50246136 | 50246137 | 0.269234 | rs1872691 | 6.35 | rs7184802 | 0.191 | 10.82 | 6.33086 | 332 | 27 | enet | 0.2 | 3.60E-29 | 6.3911 | 1.65E-10 | 7.60E-08 |
| ENSG00000166164.15 | 16 | 50368933 | 50368934 | 0.429449 | rs1872691 | 6.35 | rs11644386 | 0.346 | 14.13 | 6.02895 | 344 | 22 | enet | 0.37 | 1.20E-57 | 6.3931 | 1.63E-10 | 7.60E-08 |
| ENSG00000083799.17 | 16 | 50742049 | 50742050 | 0.003875 | rs1872691 | 6.35 | rs11644386 | -0.00174 | 3.11 | 6.02895 | 432 | 2 | lasso | 0.0016 | 0.17 | 6.0288 | 1.65E-09 | 5.02E-07 |
| ENSG00000234337.4 | 16 | 52655306 | 52655307 | -0.000982 | rs6499311 | 3.4 | rs6499311 | -0.00149 | 2.99 | 3.40034 | 457 | 1 | lasso | 0.016 | 0.0019 | 3.4003 | 6.73E-04 | 2.43E-02 |
| ENSG00000135736.5 | 16 | 57536598 | 57536599 | 0.071442 | rs9925562 | 4.83 | rs6499882 | 0.0286 | -4.48 | -3.66077 | 551 | 1 | top1 | 0.029 | 3.50E-05 | 3.6608 | 2.51E-04 | 1.20E-02 |
| ENSG00000217555.12 | 16 | 66552562 | 66552563 | -0.016873 | rs8051710 | -4.34 | rs37178 | 0.0169 | -3.57 | -3.87975 | 428 | 1 | top1 | 0.017 | 0.0012 | 3.8798 | 1.05E-04 | 6.15E-03 |
| ENSG00000140931.19 | 16 | 66603873 | 66603874 | 0.154019 | rs8051710 | -4.34 | rs8051710 | 0.163 | -9.6 | -4.33837 | 409 | 3 | lasso | 0.17 | 9.40E-25 | 4.1657 | 3.10E-05 | 2.25E-03 |
| ENSG00000172828.12 | 16 | 66961236 | 66961237 | -0.002261 | rs8056260 | -6.32 | rs28473376 | 0.00133 | 1.88 | -6.20439 | 306 | 1 | top1 | 0.0013 | 0.19 | -6.2044 | 5.49E-10 | 1.97E-07 |
| ENSG00000237172.3 | 16 | 67151213 | 67151214 | 0.032765 | rs8056260 | -6.32 | rs9033 | 0.0303 | 4.45 | -3.24617 | 261 | 1 | top1 | 0.03 | 2.10E-05 | -3.2462 | 1.17E-03 | 3.61E-02 |
| ENSG00000205250.8 | 16 | 67192168 | 67192169 | 0.02313 | rs8056260 | -6.32 | rs35356834 | 0.00152 | 3.42 | -5.18984 | 251 | 1 | top1 | 0.0015 | 0.17 | -5.1898 | 2.10E-07 | 3.81E-05 |
| ENSG00000159713.10 | 16 | 67393534 | 67393535 | 0.007166 | rs8056260 | -6.32 | rs8063771 | -0.000718 | 2.81 | -4.21491 | 228 | 3 | lasso | 0.0083 | 0.018 | -5.1341 | 2.83E-07 | 4.80E-05 |
| ENSG00000159714.10 | 16 | 67416473 | 67416474 | 0.023483 | rs8056260 | -6.32 | rs5030980 | 0.00162 | 3.56 | -6.05064 | 224 | 224 | susie | 0.003 | 0.1 | -6.0829 | 1.18E-09 | 3.80E-07 |
| ENSG00000159720.11 | 16 | 67481236 | 67481237 | 0.115744 | rs8056260 | -6.32 | rs2089402 | 0.0944 | -7.86 | -5.86171 | 213 | 213 | susie | 0.1 | 6.40E-15 | 6.1071 | 1.01E-09 | 3.32E-07 |
| ENSG00000270049.2 | 16 | 67481313 | 67481314 | 0.002325 | rs8056260 | -6.32 | rs3785098 | -0.00136 | 2.16 | -4.34318 | 213 | 1 | top1 | -0.0014 | 0.62 | -4.3432 | 1.40E-05 | 1.22E-03 |
| ENSG00000159723.4 | 16 | 67483812 | 67483813 | 0.003531 | rs8056260 | -6.32 | rs35356834 | -0.000349 | 2.56 | -5.18984 | 214 | 3 | lasso | 0.0019 | 0.15 | -5.1897 | 2.11E-07 | 3.81E-05 |
| ENSG00000261386.2 | 16 | 67563957 | 67563958 | -0.006184 | rs8056260 | -6.32 | rs11075664 | 0.0137 | 3.13 | 3.17741 | 218 | 1 | top1 | 0.014 | 0.0032 | 3.1774 | 1.49E-03 | 4.18E-02 |
| ENSG00000124074.11 | 16 | 67666763 | 67666764 | 2.90E-05 | rs8056260 | -6.32 | rs34607252 | -0.00168 | -2.12 | -0.48635 | 219 | 219 | susie | 0.00039 | 0.27 | -6.0599 | 1.36E-09 | 4.22E-07 |
| ENSG00000159761.14 | 16 | 67666815 | 67666816 | 0.045987 | rs8056260 | -6.32 | rs35356834 | 0.0464 | -5.36 | -5.18984 | 219 | 8 | lasso | 0.049 | 8.10E-08 | 5.7798 | 7.48E-09 | 2.12E-06 |
| ENSG00000102898.11 | 16 | 67846731 | 67846732 | 0.00538 | rs8056260 | -6.32 | rs28711261 | -0.00108 | 2.44 | -4.18977 | 231 | 231 | susie | -0.00079 | 0.45 | -6.1087 | 1.00E-09 | 3.32E-07 |
| ENSG00000038358.14 | 16 | 67873022 | 67873023 | 0.056386 | rs8056260 | -6.32 | rs8061122 | 0.0221 | 4.68 | -5.07782 | 237 | 23 | enet | 0.025 | 9.60E-05 | -4.2164 | 2.48E-05 | 1.89E-03 |
| ENSG00000167264.17 | 16 | 67987760 | 67987761 | 0.186295 | rs9933102 | -6.27 | rs9932414 | 0.181 | 11.03 | -5.10437 | 241 | 241 | susie | 0.28 | 3.70E-42 | -6.2788 | 3.41E-10 | 1.31E-07 |
| ENSG00000072736.18 | 16 | 68084750 | 68084751 | 0.120688 | rs7187202 | -5.42 | rs3743733 | 0.0851 | -7.16 | -2.12217 | 261 | 20 | enet | 0.098 | 2.40E-14 | 3.3398 | 8.38E-04 | 2.89E-02 |
| ENSG00000260891.1 | 16 | 68221670 | 68221671 | 0.017083 | rs7187202 | -5.42 | rs5923 | 0.0181 | -3.64 | -5.3902 | 289 | 1 | top1 | 0.018 | 0.00086 | 5.3902 | 7.04E-08 | 1.51E-05 |
| ENSG00000103067.13 | 16 | 68238101 | 68238102 | 0.003376 | rs7187202 | -5.42 | rs1073632 | -0.00132 | -2.61 | -4.96934 | 290 | 1 | lasso | -0.00027 | 0.36 | 4.9693 | 6.72E-07 | 1.00E-04 |
| ENSG00000132600.16 | 16 | 68310973 | 68310974 | 0.189917 | rs7187202 | -5.42 | rs9928605 | 0.12 | 8.38 | -2.94963 | 304 | 18 | enet | 0.16 | 5.70E-23 | -3.8636 | 1.12E-04 | 6.37E-03 |
| ENSG00000184939.15 | 16 | 68530089 | 68530090 | 0.526297 | rs7187202 | -5.42 | rs1170445 | 0.522 | -17.13 | 3.18496 | 308 | 7 | lasso | 0.53 | 7.10E-94 | -3.2752 | 1.06E-03 | 3.34E-02 |
| ENSG00000215041.9 | 17 | 7329392 | 7329393 | 0.124087 | rs35776863 | -7.24 | rs2047803 | 0.0548 | -6.16 | 4.28476 | 456 | 456 | susie | 0.055 | 1.30E-08 | -3.402064 | 6.69E-04 | 2.42E-02 |
| ENSG00000072818.11 | 17 | 7336528 | 7336529 | 0.282355 | rs35776863 | -7.24 | rs35776863 | 0.226 | -11.33 | -7.23577 | 454 | 1 | top1 | 0.23 | 5.90E-33 | 7.235771 | 4.63E-13 | 3.12E-10 |
| ENSG00000161958.10 | 17 | 7438272 | 7438273 | 0.011852 | rs35776863 | -7.24 | rs17805277 | -0.00179 | -2.54 | -4.20511 | 475 | 1 | top1 | -0.0018 | 0.95 | 4.20511 | 2.61E-05 | 1.96E-03 |
| ENSG00000161956.12 | 17 | 7561874 | 7561875 | -0.021419 | rs35776863 | -7.24 | rs2047803 | -0.00166 | -3.19 | 4.28476 | 473 | 14 | enet | -0.00053 | 0.4 | -4.639416 | 3.49E-06 | 3.68E-04 |
| ENSG00000178999.12 | 17 | 8210597 | 8210598 | 0.027234 | rs12051691 | 3.6 | rs7503353 | 0.00797 | -3.75 | 3.34505 | 438 | 1 | top1 | 0.008 | 0.02 | -3.345051 | 8.23E-04 | 2.86E-02 |
| ENSG00000232859.9 | 17 | 27893382 | 27893383 | 0.141117 | rs4794976 | -3.59 | rs11653617 | 0.0387 | 6.35 | 3.3299 | 309 | 11 | enet | 0.11 | 2.10E-16 | 3.450108 | 5.60E-04 | 2.15E-02 |
| ENSG00000087095.12 | 17 | 28041736 | 28041737 | 0.009972 | rs4794976 | -3.59 | rs11653617 | 0.0245 | 3.86 | 3.3299 | 306 | 1 | top1 | 0.024 | 0.00012 | 3.329897 | 8.69E-04 | 2.94E-02 |
| ENSG00000109107.13 | 17 | 28577263 | 28577264 | 0.000193 | rs11545699 | -3.73 | rs11545699 | -0.0018 | 2.87 | -3.72549 | 283 | 2 | lasso | 0.0041 | 0.07 | -3.813492 | 1.37E-04 | 7.43E-03 |
| ENSG00000240531.1 | 17 | 29716684 | 29716685 | 0.004774 | rs13723 | 4.77 | rs11544945 | -0.00171 | -2.69 | -4.1631 | 318 | 2 | lasso | 0.0016 | 0.17 | 5.170497 | 2.33E-07 | 4.13E-05 |
| ENSG00000108576.9 | 17 | 30236001 | 30236002 | -0.00428 | rs11544945 | -4.16 | rs11544945 | 0.000318 | -2.58 | -4.1631 | 305 | 1 | top1 | 0.00032 | 0.28 | 4.163098 | 3.14E-05 | 2.26E-03 |
| ENSG00000172716.16 | 17 | 35373700 | 35373701 | 0.048899 | rs2586514 | 2.88 | rs2671833 | 0.0118 | 3.67 | 2.81773 | 369 | 369 | susie | 0.014 | 0.0029 | 3.427896 | 6.08E-04 | 2.26E-02 |
| ENSG00000141696.12 | 17 | 41812603 | 41812604 | -0.001956 | rs12325861 | -6.99 | rs11079035 | -0.000671 | -2.85 | -6.98013 | 415 | 15 | enet | 0.002 | 0.15 | 4.347453 | 1.38E-05 | 1.22E-03 |
| ENSG00000108771.12 | 17 | 42112732 | 42112733 | 0.267535 | rs12325861 | -6.99 | rs2074166 | 0.198 | -10.68 | -2.85889 | 317 | 42 | enet | 0.22 | 3.10E-32 | 3.591076 | 3.29E-04 | 1.43E-02 |
| ENSG00000108773.10 | 17 | 42121357 | 42121358 | 0.01167 | rs12325861 | -6.99 | rs2074166 | 0.00582 | -3.64 | -2.85889 | 321 | 321 | susie | 0.012 | 0.0058 | 3.986692 | 6.70E-05 | 4.27E-03 |
| ENSG00000260325.1 | 17 | 42121430 | 42121431 | 0.083787 | rs12325861 | -6.99 | rs11079035 | 0.00176 | -3.25 | -6.98013 | 321 | 321 | susie | 0.0076 | 0.022 | 5.114038 | 3.15E-07 | 5.29E-05 |
| ENSG00000033627.16 | 17 | 42458843 | 42458844 | 0.069211 | rs12325861 | -6.99 | rs3760387 | 0.0133 | 4.54 | -2.71688 | 252 | 252 | susie | 0.024 | 0.00015 | -3.298749 | 9.71E-04 | 3.17E-02 |
| ENSG00000267185.1 | 17 | 42533531 | 42533532 | -0.004213 | rs12325861 | -6.99 | rs2883456 | -0.000339 | -2.98 | -4.21093 | 248 | 1 | lasso | 0.0032 | 0.097 | 4.210926 | 2.54E-05 | 1.92E-03 |
| ENSG00000108799.12 | 17 | 42745048 | 42745049 | 0.032857 | rs7211777 | -4.34 | rs7359598 | 0.0495 | -5.48 | 3.27543 | 265 | 1 | top1 | 0.05 | 6.50E-08 | -3.275426 | 1.06E-03 | 3.34E-02 |
| ENSG00000131477.10 | 17 | 42761257 | 42761258 | -0.002677 | rs7211777 | -4.34 | rs963987 | 0.00199 | -2.04 | -4.05136 | 258 | 1 | top1 | 0.002 | 0.15 | 4.051362 | 5.09E-05 | 3.38E-03 |
| ENSG00000225190.10 | 17 | 45490748 | 45490749 | 0.061945 | rs17630064 | 3.62 | rs16940665 | 0.00401 | -4.34 | -2.3475 | 158 | 158 | susie | 0.012 | 0.0059 | 3.579623 | 3.44E-04 | 1.48E-02 |
| ENSG00000228782.7 | 17 | 47492491 | 47492492 | 0.070364 | rs11079786 | -7.85 | rs2175290 | 0.0779 | -7.4 | -2.87167 | 358 | 358 | susie | 0.082 | 3.40E-12 | 3.112684 | 1.85E-03 | 4.87E-02 |
| ENSG00000141279.15 | 17 | 47522941 | 47522942 | 0.043644 | rs11079786 | -7.85 | rs11652097 | 0.00247 | 4.12 | -5.6612 | 366 | 366 | susie | 0.0097 | 0.011 | -7.867736 | 3.61E-15 | 3.42E-12 |
| ENSG00000198933.9 | 17 | 47694080 | 47694081 | 0.253284 | rs11079786 | -7.85 | rs9913503 | 0.322 | 13.49 | -4.09503 | 412 | 412 | susie | 0.32 | 3.70E-49 | -4.211861 | 2.53E-05 | 1.92E-03 |
| ENSG00000159111.12 | 17 | 47831533 | 47831534 | 0.192228 | rs11079786 | -7.85 | rs17774272 | 0.0397 | -6.67 | -3.96674 | 384 | 29 | enet | 0.084 | 1.80E-12 | 4.401169 | 1.08E-05 | 9.72E-04 |
| ENSG00000264920.1 | 17 | 47895811 | 47895812 | 0.145447 | rs11079786 | -7.85 | rs1130932 | 0.0302 | 5.68 | -1.37064 | 380 | 14 | enet | 0.062 | 1.40E-09 | -3.604697 | 3.13E-04 | 1.37E-02 |
| ENSG00000234494.7 | 17 | 47941409 | 47941410 | 0.0174 | rs11079786 | -7.85 | rs12451815 | -0.00172 | 2.67 | -6.59072 | 376 | 376 | susie | 0.00077 | 0.23 | -3.820298 | 1.33E-04 | 7.29E-03 |
| ENSG00000132470.13 | 17 | 75721327 | 75721328 | 0.020146 | rs11868566 | -5.07 | rs17581498 | 0.00501 | -2.68 | -4.2216 | 356 | 1 | top1 | 0.005 | 0.052 | 4.221604 | 2.43E-05 | 1.87E-03 |
| ENSG00000108479.11 | 17 | 75765710 | 75765711 | 0.077584 | rs11868566 | -5.07 | rs8076052 | 0.0433 | -5.91 | -3.02518 | 357 | 6 | lasso | 0.062 | 1.60E-09 | 4.056049 | 4.99E-05 | 3.32E-03 |
| ENSG00000132471.11 | 17 | 75856506 | 75856507 | 0.044554 | rs11868566 | -5.07 | rs17581498 | 0.00776 | -4.2 | -4.2216 | 381 | 9 | enet | 0.0097 | 0.011 | 4.728753 | 2.26E-06 | 2.58E-04 |
| ENSG00000267801.1 | 17 | 75876492 | 75876493 | 0.048835 | rs11868566 | -5.07 | rs9908862 | 0.0315 | 5.1 | -4.7206 | 382 | 1 | top1 | 0.032 | 1.50E-05 | -4.720597 | 2.35E-06 | 2.67E-04 |
| ENSG00000267249.1 | 18 | 12671144 | 12671145 | 0.022674 | rs2847297 | -6.99 | rs9945801 | -0.000487 | 3.34 | -2.49515 | 372 | 5 | lasso | 0.015 | 0.0021 | -4.08751 | 4.36E-05 | 3.02E-03 |
| ENSG00000267654.1 | 18 | 12749420 | 12749421 | 0.388328 | rs2847297 | -6.99 | rs2542157 | 0.163 | -10.13 | 4.24346 | 394 | 11 | lasso | 0.22 | 9.50E-33 | -5.21634 | 1.82E-07 | 3.41E-05 |
| ENSG00000260302.1 | 18 | 12775922 | 12775923 | 0.289907 | rs2847297 | -6.99 | rs2542157 | 0.181 | -10.08 | 4.24346 | 385 | 30 | enet | 0.19 | 6.20E-28 | -4.42088 | 9.83E-06 | 9.05E-04 |
| ENSG00000134504.12 | 18 | 26657400 | 26657401 | 0.000458 | rs11083166 | -3.81 | rs1530599 | 0.00137 | -2.69 | 3.43303 | 554 | 1 | top1 | 0.0014 | 0.18 | -3.43303 | 5.97E-04 | 2.24E-02 |
| ENSG00000206052.10 | 18 | 69401054 | 69401055 | 0.132127 | rs727088 | -6.17 | rs1790588 | 0.0889 | 7.22 | -6.0986 | 550 | 550 | susie | 0.094 | 8.00E-14 | -5.40528 | 6.47E-08 | 1.42E-05 |
| ENSG00000150637.8 | 18 | 69961802 | 69961803 | 0.048376 | rs727088 | -6.17 | rs763361 | -0.000759 | -3.92 | -6.01488 | 527 | 527 | susie | 0.001 | 0.21 | 6.14777 | 7.86E-10 | 2.70E-07 |
| ENSG00000131196.17 | 18 | 79395855 | 79395856 | 0.117972 | rs1562722 | -5.8 | rs2958431 | 0.00915 | -4.22 | 1.2623 | 412 | 7 | lasso | 0.041 | 8.20E-07 | -3.99845 | 6.38E-05 | 4.13E-03 |
| ENSG00000248015.6 | 19 | 1396466 | 1396467 | 0.014612 | rs12459067 | 4.91 | rs3752243 | 0.0127 | -3.3 | 3.402002 | 401 | 1 | top1 | 0.013 | 0.0044 | -3.402002 | 6.69E-04 | 2.42E-02 |
| ENSG00000130270.16 | 19 | 1812275 | 1812276 | 0.138835 | rs2289286 | 2.82 | rs4807143 | 0.0874 | -7.64 | -2.228261 | 373 | 16 | enet | 0.14 | 3.30E-20 | 3.142518 | 1.68E-03 | 4.54E-02 |
| ENSG00000269318.1 | 19 | 4358447 | 4358448 | -0.01466 | rs10424978 | -9.77 | rs11085094 | -0.00178 | -2.58 | 3.184353 | 377 | 1 | top1 | -0.0018 | 0.93 | -3.184353 | 1.45E-03 | 4.10E-02 |
| ENSG00000127666.9 | 19 | 4831703 | 4831704 | 0.07539 | rs10424978 | -9.77 | rs7255265 | 0.0279 | 5.17 | -8.020326 | 427 | 1 | top1 | 0.028 | 4.40E-05 | -8.020326 | 1.05E-15 | 1.21E-12 |
| ENSG00000105397.13 | 19 | 10380675 | 10380676 | 0.13208 | rs2304256 | 6.66 | rs2304256 | 0.0553 | 6.83 | 6.6624 | 375 | 14 | enet | 0.088 | 4.80E-13 | 6.452587 | 1.10E-10 | 5.37E-08 |
| ENSG00000234773.7 | 19 | 12195014 | 12195015 | -0.009727 | rs8113106 | 3.62 | rs8113106 | 0.00483 | 2.3 | 3.620722 | 293 | 1 | top1 | 0.0048 | 0.055 | 3.620722 | 2.94E-04 | 1.32E-02 |
| ENSG00000141854.9 | 19 | 14072535 | 14072536 | 0.041494 | rs10404803 | 3.42 | rs2306190 | 0.0123 | 4.18 | 2.998581 | 288 | 10 | enet | 0.028 | 3.80E-05 | 3.246782 | 1.17E-03 | 3.61E-02 |
| ENSG00000099308.10 | 19 | 18097792 | 18097793 | 0.068191 | rs2384992 | -7.07 | rs273506 | 0.076 | -6.62 | 3.188746 | 419 | 2 | lasso | 0.078 | 1.20E-11 | -3.51413 | 4.41E-04 | 1.79E-02 |
| ENSG00000254858.9 | 19 | 18193181 | 18193182 | 0.073492 | rs2384992 | -7.07 | rs2271881 | 0.0341 | 5.61 | 4.750216 | 420 | 1 | top1 | 0.034 | 6.80E-06 | 4.750216 | 2.03E-06 | 2.41E-04 |
| ENSG00000130518.16 | 19 | 18274508 | 18274509 | 0.233699 | rs2384992 | -7.07 | rs12610691 | 0.164 | 10.16 | 6.563474 | 411 | 411 | susie | 0.23 | 3.20E-33 | 5.161827 | 2.45E-07 | 4.29E-05 |
| ENSG00000105701.15 | 19 | 18544076 | 18544077 | 0.012096 | rs2384992 | -7.07 | rs11667487 | 0.00261 | 2.7 | 4.553977 | 379 | 1 | top1 | 0.0026 | 0.12 | 4.553977 | 5.26E-06 | 5.17E-04 |
| ENSG00000005007.12 | 19 | 18831937 | 18831938 | 0.007512 | rs2040562 | -3.58 | rs3826994 | 0.000152 | -2.82 | -1.137649 | 351 | 351 | susie | 0.0013 | 0.19 | 3.172186 | 1.51E-03 | 4.23E-02 |
| ENSG00000167491.17 | 19 | 19385825 | 19385826 | 0.091922 | rs2040562 | -3.58 | rs4808200 | 0.145 | 9.28 | -3.311487 | 344 | 344 | susie | 0.15 | 6.10E-21 | -3.290307 | 1.00E-03 | 3.24E-02 |
| ENSG00000186010.18 | 19 | 19515735 | 19515736 | 0.03581 | rs7258519 | 3.95 | rs11667828 | 0.00102 | 4.07 | -2.574707 | 356 | 356 | susie | 0.0092 | 0.013 | -3.287522 | 1.01E-03 | 3.26E-02 |
| ENSG00000160161.9 | 19 | 19538247 | 19538248 | 0.089798 | rs7258519 | 3.95 | rs16995922 | -0.00179 | -3.16 | -3.350714 | 355 | 355 | susie | 0.0033 | 0.091 | 3.823015 | 1.32E-04 | 7.29E-03 |
| ENSG00000240522.1 | 19 | 21150437 | 21150438 | 0.008989 | rs2292986 | 4.5 | rs3951363 | 0.0146 | 4.07 | 3.113064 | 280 | 280 | susie | 0.016 | 0.0016 | 3.616389 | 2.99E-04 | 1.32E-02 |
| ENSG00000268081.1 | 19 | 21569236 | 21569237 | 0.138632 | rs2292986 | 4.5 | rs8110814 | 0.196 | 10.61 | -3.741744 | 305 | 1 | top1 | 0.2 | 1.90E-28 | -3.741744 | 1.83E-04 | 9.39E-03 |
| ENSG00000221946.7 | 19 | 35143249 | 35143250 | 0.169213 | rs12110 | -4.37 | rs4805110 | 0.109 | 8.24 | -3.593092 | 488 | 488 | susie | 0.11 | 2.80E-16 | -3.659985 | 2.52E-04 | 1.20E-02 |
| ENSG00000105287.12 | 19 | 46717126 | 46717127 | 0.325999 | rs12973505 | 4.08 | rs3786705 | 0.216 | 11.53 | 2.781487 | 351 | 17 | enet | 0.33 | 5.10E-51 | 3.138426 | 1.70E-03 | 4.57E-02 |
| ENSG00000118162.13 | 19 | 47484267 | 47484268 | 0.081224 | rs12973505 | 4.08 | rs4804044 | -0.00101 | -3.13 | 1.677803 | 358 | 358 | susie | 0.023 | 0.00017 | -3.338602 | 8.42E-04 | 2.90E-02 |
| ENSG00000232871.8 | 19 | 48638144 | 48638145 | 0.152126 | rs838144 | -5.19 | rs550455 | 0.0838 | -7.06 | 3.157402 | 462 | 1 | top1 | 0.084 | 1.90E-12 | -3.157402 | 1.59E-03 | 4.39E-02 |
| ENSG00000063180.8 | 19 | 48646311 | 48646312 | 0.03075 | rs838144 | -5.19 | rs550455 | 0.0189 | 4.26 | 3.157402 | 461 | 1 | top1 | 0.019 | 0.00066 | 3.157402 | 1.59E-03 | 4.39E-02 |
| ENSG00000142233.11 | 19 | 48673080 | 48673081 | 0.116777 | rs838144 | -5.19 | rs516246 | 0.0596 | 5.97 | -5.036448 | 459 | 18 | enet | 0.068 | 2.70E-10 | -4.554837 | 5.24E-06 | 5.17E-04 |
| ENSG00000105538.9 | 19 | 48740720 | 48740721 | 0.026722 | rs838144 | -5.19 | rs12975781 | 0.0172 | -3.89 | 3.461354 | 436 | 1 | lasso | 0.021 | 0.00032 | -3.461354 | 5.37E-04 | 2.09E-02 |
| ENSG00000104894.11 | 19 | 49335170 | 49335171 | 0.084289 | rs12981033 | 6.61 | rs8107548 | 0.0474 | -6.36 | -3.98307 | 377 | 377 | susie | 0.052 | 2.90E-08 | 3.974271 | 7.06E-05 | 4.48E-03 |
| ENSG00000161618.9 | 19 | 49453168 | 49453169 | 0.403124 | rs12981033 | 6.61 | rs2293013 | 0.419 | 15.38 | -5.004542 | 371 | 371 | susie | 0.42 | 1.40E-68 | -5.001492 | 5.69E-07 | 8.73E-05 |
| ENSG00000104870.12 | 19 | 49512278 | 49512279 | 0.074612 | rs12981033 | 6.61 | rs4560020 | 0.0627 | -6.4 | 3.082434 | 366 | 366 | susie | 0.065 | 6.70E-10 | -3.129086 | 1.75E-03 | 4.67E-02 |
| ENSG00000142552.7 | 19 | 49527617 | 49527618 | 0.188897 | rs12981033 | 6.61 | rs7256116 | 0.152 | -9.72 | 3.064982 | 369 | 4 | lasso | 0.16 | 2.00E-23 | -3.234094 | 1.22E-03 | 3.69E-02 |
| ENSG00000126453.9 | 19 | 49665875 | 49665876 | 0.001727 | rs12981033 | 6.61 | rs2946848 | -0.00112 | 2.91 | 0.403291 | 324 | 324 | susie | -1.00E-04 | 0.33 | 5.528375 | 3.23E-08 | 7.89E-06 |
| ENSG00000268006.1 | 19 | 49851675 | 49851676 | 0.03739 | rs12981033 | 6.61 | rs1290652 | 0.00216 | 3.53 | -0.014928 | 346 | 346 | susie | 0.011 | 0.0066 | 4.251679 | 2.12E-05 | 1.68E-03 |
| ENSG00000129450.8 | 19 | 51124907 | 51124908 | 0.02187 | rs12983058 | 3.49 | rs12983058 | 0.00587 | 4.24 | 3.49155 | 622 | 1 | top1 | 0.0059 | 0.039 | 3.49155 | 4.80E-04 | 1.92E-02 |
| ENSG00000168995.13 | 19 | 51142298 | 51142299 | 0.043632 | rs12983058 | 3.49 | rs12983058 | 0.0605 | 5.97 | 3.49155 | 615 | 1 | top1 | 0.061 | 2.30E-09 | 3.49155 | 4.80E-04 | 1.92E-02 |
| ENSG00000267649.1 | 19 | 55216659 | 55216660 | -0.015093 | rs3826884 | -4.87 | rs10419308 | 0.00786 | 3.33 | -4.708268 | 436 | 1 | top1 | 0.0079 | 0.02 | -4.708268 | 2.50E-06 | 2.80E-04 |
| ENSG00000105063.18 | 19 | 55258994 | 55258995 | 0.041835 | rs3826884 | -4.87 | rs4806656 | 0.0118 | 4.57 | 2.683447 | 456 | 11 | enet | 0.014 | 0.0034 | 3.318222 | 9.06E-04 | 3.03E-02 |
| ENSG00000125991.19 | 20 | 35542020 | 35542021 | 0.017005 | rs6060450 | 2.86 | rs1033798 | 0.00248 | -2.52 | 1.53205 | 316 | 21 | enet | 0.004 | 0.072 | -3.41205 | 6.45E-04 | 2.36E-02 |
| ENSG00000196756.11 | 20 | 38435352 | 38435353 | 0.574305 | rs788348 | 3.38 | rs1780636 | 0.574 | 17.98 | 3.311217 | 477 | 33 | enet | 0.58 | 1.20E-107 | 3.43856 | 5.85E-04 | 2.21E-02 |
| ENSG00000166913.12 | 20 | 44885675 | 44885676 | 0.111374 | rs16989481 | 3.22 | rs12481468 | 0.091 | -7.37 | 3.143466 | 430 | 430 | susie | 0.093 | 1.30E-13 | -3.17008 | 1.52E-03 | 4.25E-02 |
| ENSG00000258366.7 | 20 | 63657809 | 63657810 | 0.067302 | rs816935 | 3.88 | rs879471 | 0.0408 | -5.53 | -3.171887 | 429 | 1 | top1 | 0.041 | 9.00E-07 | 3.17189 | 1.52E-03 | 4.24E-02 |
| ENSG00000171703.16 | 20 | 64049835 | 64049836 | 0.059004 | rs816935 | 3.88 | rs8121509 | -0.0017 | 3.42 | 2.503813 | 330 | 3 | lasso | 0.023 | 0.00019 | 3.16673 | 1.54E-03 | 4.29E-02 |
| ENSG00000160185.14 | 21 | 42403446 | 42403447 | 0.333831 | rs12482947 | 5.83 | rs1893592 | 0.265 | 12.19 | 4.692 | 759 | 1 | top1 | 0.27 | 2.60E-39 | 4.69 | 2.71E-06 | 2.99E-04 |
| ENSG00000185651.14 | 22 | 21567546 | 21567547 | 0.110376 | rs2876981 | 3.88 | rs4821130 | 0.107 | 8.48 | -3.44791 | 286 | 286 | susie | 0.12 | 5.40E-17 | -3.54536 | 3.92E-04 | 1.62E-02 |
| ENSG00000224086.5 | 22 | 21938584 | 21938585 | 0.18046 | rs2876981 | 3.88 | rs240066 | 0.144 | -10.18 | 2.27018 | 416 | 416 | susie | 0.23 | 1.70E-33 | -3.45373 | 5.53E-04 | 2.13E-02 |
| ENSG00000100034.13 | 22 | 21940656 | 21940657 | 0.311318 | rs2876981 | 3.88 | rs3788333 | 0.293 | -12.93 | 3.47139 | 416 | 416 | susie | 0.32 | 3.30E-49 | -3.55529 | 3.78E-04 | 1.59E-02 |
| ENSG00000100038.19 | 22 | 21982815 | 21982816 | 0.034539 | rs2876981 | 3.88 | rs9610608 | 0.0135 | -3.9 | 3.48747 | 413 | 11 | enet | 0.016 | 0.0017 | -4.08989 | 4.32E-05 | 3.01E-03 |
| ENSG00000100314.3 | 22 | 29720083 | 29720084 | 0.01328 | rs5997579 | 6.55 | rs9614158 | -0.00169 | 3.16 | 3.55456 | 376 | 2 | lasso | -0.00031 | 0.36 | 3.60663 | 3.10E-04 | 1.37E-02 |
| ENSG00000184076.13 | 22 | 29767368 | 29767369 | 0.04382 | rs5997579 | 6.55 | rs737787 | 0.005 | -4.56 | -0.82731 | 371 | 5 | enet | 0.023 | 0.00018 | 3.31445 | 9.18E-04 | 3.04E-02 |
| ENSG00000100330.15 | 22 | 29883154 | 29883155 | 0.130662 | rs5997579 | 6.55 | rs713718 | 0.108 | -8.16 | 2.45646 | 391 | 391 | susie | 0.11 | 1.90E-16 | -3.55591 | 3.77E-04 | 1.59E-02 |
| ENSG00000128342.4 | 22 | 30244932 | 30244933 | 0.024383 | rs5997579 | 6.55 | rs41172 | 0.0122 | 3.74 | 4.06936 | 430 | 1 | top1 | 0.012 | 0.0052 | 4.06936 | 4.71E-05 | 3.19E-03 |
| ENSG00000268812.3 | 22 | 30246204 | 30246205 | 0.015086 | rs5997579 | 6.55 | rs11090598 | 0.00187 | 3.46 | 5.05786 | 431 | 7 | lasso | 0.0086 | 0.016 | 4.93339 | 8.08E-07 | 1.16E-04 |
| ENSG00000253352.8 | 22 | 30970676 | 30970677 | -0.014302 | rs12485048 | 4.1 | rs740234 | -0.00158 | -3 | -2.99386 | 378 | 2 | enet | -0.0011 | 0.52 | 3.11789 | 1.82E-03 | 4.83E-02 |
| ENSG00000100379.17 | 22 | 37051735 | 37051736 | 0.038252 | rs229540 | -11.3 | rs229540 | -0.00172 | -3.18 | -11.30328 | 675 | 2 | lasso | -9.00E-04 | 0.48 | 11.30325 | 1.26E-29 | 5.08E-26 |
| ENSG00000133466.13 | 22 | 37199384 | 37199385 | 0.340369 | rs229540 | -11.3 | rs9610669 | 0.198 | 10.72 | -1.98032 | 648 | 15 | enet | 0.24 | 3.30E-35 | -5.23692 | 1.63E-07 | 3.13E-05 |
| ENSG00000128340.14 | 22 | 37244447 | 37244448 | 0.093565 | rs229540 | -11.3 | rs9798725 | 0.0583 | -6.06 | 3.51013 | 634 | 14 | enet | 0.061 | 1.80E-09 | -5.03848 | 4.69E-07 | 7.56E-05 |
| ENSG00000100129.17 | 22 | 37849327 | 37849328 | 0.006004 | rs16998847 | -3.16 | rs2267368 | 0.00947 | -3.45 | -2.08318 | 406 | 5 | lasso | 0.023 | 0.00021 | 3.4891 | 4.85E-04 | 1.93E-02 |
| ENSG00000100316.15 | 22 | 39320388 | 39320389 | 0.030634 | rs2069235 | -5.12 | rs5757582 | -0.000882 | -3.52 | -3.90735 | 395 | 2 | lasso | 0.0012 | 0.19 | 4.97926 | 6.38E-07 | 9.61E-05 |
| ENSG00000100321.14 | 22 | 39349924 | 39349925 | 0.284668 | rs2069235 | -5.12 | rs2069235 | 0.18 | 10.96 | -5.11953 | 385 | 15 | enet | 0.23 | 5.50E-34 | -5.47769 | 4.31E-08 | 1.01E-05 |
| ENSG00000100335.13 | 22 | 39499431 | 39499432 | 0.015114 | rs2069235 | -5.12 | rs137985 | -0.00155 | 3.12 | -3.28678 | 394 | 1 | lasso | 0.0024 | 0.13 | -3.28678 | 1.01E-03 | 3.26E-02 |
| ENSG00000230071.2 | 22 | 40588002 | 40588003 | 0.028492 | rs11913132 | 2.67 | rs4507196 | 0.00163 | 3.09 | -0.85401 | 277 | 23 | enet | 0.0048 | 0.056 | -4.24373 | 2.20E-05 | 1.74E-03 |
| ENSG00000100401.19 | 22 | 41286250 | 41286251 | 0.044755 | rs4820437 | 5.46 | rs139507 | 0.0277 | 4.32 | -4.16366 | 263 | 1 | top1 | 0.028 | 4.60E-05 | -4.16366 | 3.13E-05 | 2.26E-03 |
| ENSG00000167074.14 | 22 | 41367332 | 41367333 | 0.174946 | rs4820437 | 5.46 | rs2234059 | 0.201 | 11.08 | 5.15827 | 262 | 24 | enet | 0.22 | 7.80E-32 | 5.05497 | 4.30E-07 | 7.07E-05 |
| ENSG00000172346.14 | 22 | 41560762 | 41560763 | 0.021417 | rs4820437 | 5.46 | rs5758397 | 0.0167 | 3.77 | 4.25599 | 305 | 1 | top1 | 0.017 | 0.0013 | 4.25599 | 2.08E-05 | 1.68E-03 |
| ENSG00000100417.11 | 22 | 41589889 | 41589890 | 0.193201 | rs4820437 | 5.46 | rs4820446 | 0.0743 | -6.93 | 4.2239 | 309 | 46 | enet | 0.097 | 3.40E-14 | -3.26878 | 1.08E-03 | 3.38E-02 |
| ENSG00000167077.12 | 22 | 41699498 | 41699499 | 0.24445 | rs4820437 | 5.46 | rs4820444 | 0.321 | 13.39 | 4.17666 | 307 | 1 | top1 | 0.32 | 7.40E-49 | 4.17666 | 2.96E-05 | 2.19E-03 |
| ENSG00000183066.14 | 22 | 41998724 | 41998725 | 0.054233 | rs139559 | 4.41 | rs134904 | 0.00282 | 3.64 | -1.69351 | 353 | 353 | susie | 0.0034 | 0.089 | -3.12931 | 1.75E-03 | 4.67E-02 |
| ENSG00000100241.20 | 22 | 50475023 | 50475024 | 0.046109 | rs738683 | -5.43 | rs5771039 | 0.0127 | 3.95 | -5.27421 | 338 | 12 | enet | 0.019 | 7.00E-04 | -3.16412 | 1.56E-03 | 4.33E-02 |

**Supplemental Table S16. Candidate genes in blood associated with RA (discovery) using TWAS.**

| **ID** | **CHR** | **Start** | **End** | **HSQ** | **BEST.GWAS.ID** | **BEST.GWAS.Z** | **EQTL.ID** | **EQTL.R2** | **EQTL.Z** | **EQTL.GWAS.Z** | **NSNP** | **NWGT** | **MODEL** | **MODELCV.R2** | **MODELCV.PV** | **TWAS.Z** | **TWAS.P** | **padj** |
| --- | --- | --- | --- | --- | --- | --- | --- | --- | --- | --- | --- | --- | --- | --- | --- | --- | --- | --- |
| ENSG00000224870.6 | 1 | 1399521 | 1399522 | 0.013977 | rs1240708 | -4.0517 | rs12093154 | -0.0018 | 3.389 | -2.906774 | 275 | 275 | susie | -0.0012 | 0.56 | -3.587957 | 3.33E-04 | 4.32E-02 |
| ENSG00000242485.5 | 1 | 1407312 | 1407313 | 0.080426 | rs1240708 | -4.0517 | rs1240708 | 0.0365 | 5.13 | -4.051671 | 276 | 1 | top1 | 0.036 | 3.30E-06 | -4.051671 | 5.09E-05 | 1.32E-02 |
| ENSG00000142606.15 | 1 | 2632989 | 2632990 | 0.163611 | rs2843401 | 4.5644 | rs3748816 | 0.126 | -9.371 | 4.497766 | 356 | 32 | enet | 0.19 | 4.80E-27 | -3.578072 | 3.46E-04 | 4.42E-02 |
| ENSG00000215912.12 | 1 | 2801716 | 2801717 | 0.067302 | rs2843401 | 4.5644 | rs4648356 | 0.0699 | 6.883 | 4.343338 | 369 | 369 | susie | 0.073 | 5.40E-11 | 4.461257 | 8.15E-06 | 4.93E-03 |
| ENSG00000186715.10 | 1 | 16770236 | 16770237 | 0.20926 | rs732679 | 3.6441 | rs9435734 | 0.142 | 9.235 | 3.065639 | 291 | 291 | susie | 0.15 | 2.60E-21 | 3.617806 | 2.97E-04 | 4.05E-02 |
| ENSG00000117118.9 | 1 | 17054169 | 17054170 | 0.001074 | rs732679 | 3.6441 | rs1635577 | -0.000412 | -2.304 | -1.190755 | 360 | 360 | susie | 0.0094 | 0.012 | 3.77051 | 1.63E-04 | 2.97E-02 |
| ENSG00000229162.1 | 1 | 24961344 | 24961345 | 0.016401 | rs4478762 | -3.6129 | rs4478762 | 0.0118 | 3.75 | -3.612891 | 350 | 1 | top1 | 0.012 | 0.0059 | -3.612891 | 3.03E-04 | 4.10E-02 |
| ENSG00000261349.1 | 1 | 25267135 | 25267136 | 0.621188 | rs4478762 | -3.6129 | rs3091242 | 0.303 | 13.186 | -3.124343 | 350 | 350 | susie | 0.55 | 2.90E-99 | -4.402029 | 1.07E-05 | 5.18E-03 |
| ENSG00000187010.19 | 1 | 25272392 | 25272393 | 0.752991 | rs4478762 | -3.6129 | rs3091242 | 0.332 | 13.635 | -3.124343 | 351 | 351 | susie | 0.68 | 2.40E-141 | -4.398083 | 1.09E-05 | 5.18E-03 |
| ENSG00000224183.1 | 1 | 25294642 | 25294643 | 0.567311 | rs4478762 | -3.6129 | rs3091242 | 0.222 | 11.202 | -3.124343 | 364 | 364 | susie | 0.48 | 3.30E-80 | -4.335742 | 1.45E-05 | 6.28E-03 |
| ENSG00000183726.10 | 1 | 25338213 | 25338214 | 0.421536 | rs4478762 | -3.6129 | rs3091242 | 0.111 | 7.917 | -3.124343 | 376 | 376 | susie | 0.31 | 2.20E-47 | -4.23572 | 2.28E-05 | 8.22E-03 |
| ENSG00000126698.10 | 1 | 28233024 | 28233025 | 0.169317 | rs510379 | 3.6947 | rs490633 | 0.149 | -9.303 | 3.61748 | 253 | 1 | top1 | 0.15 | 1.60E-21 | -3.61748 | 2.97E-04 | 4.05E-02 |
| ENSG00000162437.14 | 1 | 64745094 | 64745095 | 0.08356 | rs6676160 | 5.7735 | rs1874406 | -0.0012 | 4.145 | -1.23646 | 452 | 49 | enet | 0.011 | 0.0087 | -4.104706 | 4.05E-05 | 1.15E-02 |
| ENSG00000162434.11 | 1 | 64966503 | 64966504 | 0.080168 | rs6676160 | 5.7735 | rs6696692 | 0.0158 | -4.474 | 4.117286 | 438 | 4 | lasso | 0.017 | 0.001 | -3.755876 | 1.73E-04 | 3.05E-02 |
| ENSG00000226891.7 | 1 | 65002488 | 65002489 | 0.0618 | rs6676160 | 5.7735 | rs7549445 | 0.048 | -5.882 | 4.142791 | 451 | 7 | lasso | 0.051 | 4.60E-08 | -3.563809 | 3.66E-04 | 4.57E-02 |
| ENSG00000197852.9 | 1 | 111755243 | 111755244 | 0.092615 | rs669088 | -4.2108 | rs535317 | 0.0182 | 5.471 | 3.044006 | 683 | 11 | enet | 0.06 | 2.50E-09 | 4.028208 | 5.62E-05 | 1.34E-02 |
| ENSG00000226419.7 | 1 | 112956414 | 112956415 | 0.102138 | rs11811051 | -11.2968 | rs7524567 | 0.0422 | -6.287 | -4.393506 | 328 | 16 | enet | 0.086 | 8.90E-13 | 4.912611 | 8.99E-07 | 1.17E-03 |
| ENSG00000232450.1 | 1 | 113699630 | 113699631 | 0.091181 | rs2476601 | -20.348 | rs6669008 | 0.0224 | -5.217 | 6.227911 | 486 | 2 | lasso | 0.034 | 6.90E-06 | -5.763197 | 8.25E-09 | 3.35E-05 |
| ENSG00000134242.15 | 1 | 113871758 | 113871759 | 0.042669 | rs2476601 | -20.348 | rs2279380 | -0.00116 | 3.603 | -3.408318 | 483 | 15 | enet | 0.008 | 0.02 | -6.852639 | 7.25E-12 | 3.92E-08 |
| ENSG00000118655.4 | 1 | 113905201 | 113905202 | 0.010875 | rs2476601 | -20.348 | rs4462141 | -0.00152 | -3.126 | -2.806377 | 469 | 469 | susie | -9.00E-04 | 0.48 | 12.679203 | 7.71E-37 | 1.25E-32 |
| ENSG00000163207.6 | 1 | 152908544 | 152908545 | 0.002215 | rs7517755 | 3.6899 | rs7517755 | 0.00055 | -3.071 | 3.689945 | 516 | 1 | lasso | 0.0043 | 0.065 | -3.689945 | 2.24E-04 | 3.40E-02 |
| ENSG00000198821.10 | 1 | 167518609 | 167518610 | 0.046103 | rs2056626 | 5.2707 | rs7523907 | 0.00381 | 3.911 | 5.054979 | 607 | 6 | enet | 0.0063 | 0.034 | 4.97029 | 6.69E-07 | 1.17E-03 |
| ENSG00000144218.18 | 2 | 100142738 | 100142739 | 0.070765 | rs11887597 | 4.578 | rs2309811 | 0.0154 | 5.35 | -3.60164 | 456 | 456 | susie | 0.037 | 3.30E-06 | -4.049503 | 5.13E-05 | 1.32E-02 |
| ENSG00000003393.14 | 2 | 201781188 | 201781189 | 0.198017 | rs12693945 | 4.716 | rs10211223 | 0.214 | -10.94 | 3.73821 | 422 | 26 | enet | 0.22 | 9.80E-32 | -3.946158 | 7.94E-05 | 1.74E-02 |
| ENSG00000135913.10 | 2 | 218568360 | 218568361 | 0.045064 | rs4674332 | -4.077 | rs591573 | 0.0376 | 5.11 | -3.5738 | 364 | 364 | susie | 0.038 | 2.30E-06 | -3.660588 | 2.52E-04 | 3.65E-02 |
| ENSG00000144580.13 | 2 | 218568579 | 218568580 | 0.119528 | rs4674332 | -4.077 | rs6720403 | 0.228 | -11.5 | -3.74985 | 364 | 364 | susie | 0.23 | 8.90E-34 | 3.679535 | 2.34E-04 | 3.45E-02 |
| ENSG00000115596.3 | 2 | 218859820 | 218859821 | 0.014031 | rs4674332 | -4.077 | rs4674338 | 0.0109 | 3.18 | -4.06559 | 380 | 1 | top1 | 0.011 | 0.0078 | -4.065593 | 4.79E-05 | 1.27E-02 |
| ENSG00000173585.15 | 3 | 45886503 | 45886504 | 0.046959 | rs2131091 | 3.97 | rs17713054 | 0.00277 | 3.97 | -2.11725 | 445 | 445 | susie | 0.0092 | 0.013 | -3.54428 | 3.94E-04 | 4.72E-02 |
| ENSG00000183625.14 | 3 | 46163603 | 46163604 | 0.279076 | rs2131091 | 3.97 | rs7616215 | 0.147 | -9.62 | -2.43424 | 458 | 28 | enet | 0.23 | 5.00E-34 | 3.67884 | 2.34E-04 | 3.45E-02 |
| ENSG00000277855.1 | 3 | 71627640 | 71627641 | -0.030797 | rs12633797 | 4.79 | rs13085841 | 0.00322 | 2.67 | 3.77782 | 530 | 1 | top1 | 0.0032 | 0.095 | 3.77782 | 1.58E-04 | 2.95E-02 |
| ENSG00000170837.2 | 3 | 71754049 | 71754050 | 0.043814 | rs13085841 | 3.78 | rs7644362 | 0.0271 | 4.1 | 3.55523 | 558 | 1 | top1 | 0.027 | 5.60E-05 | 3.55523 | 3.78E-04 | 4.61E-02 |
| ENSG00000163833.7 | 3 | 121593118 | 121593119 | 0.090673 | rs2332056 | -4.76 | rs1492177 | 0.0591 | 6.37 | -4.00313 | 342 | 342 | susie | 0.063 | 9.60E-10 | -4.10152 | 4.10E-05 | 1.15E-02 |
| ENSG00000222057.1 | 3 | 121655603 | 121655604 | 0.010581 | rs2332056 | -4.76 | rs2331963 | -0.00113 | -3.09 | -4.00176 | 341 | 8 | enet | 0.0018 | 0.16 | 3.7017 | 2.14E-04 | 3.35E-02 |
| ENSG00000223117.1 | 3 | 190642162 | 190642163 | -0.022283 | rs6764286 | 4.06 | rs2241343 | -0.000487 | 2.93 | 4.04252 | 519 | 1 | lasso | 0.0015 | 0.18 | 4.04252 | 5.29E-05 | 1.33E-02 |
| ENSG00000168769.13 | 4 | 105145874 | 105145875 | 0.027976 | rs12498981 | -5.21 | rs2636729 | 0.000989 | -3.71 | 1.500535 | 381 | 381 | susie | 0.011 | 0.0084 | -3.591754 | 3.28E-04 | 4.29E-02 |
| ENSG00000134352.19 | 5 | 55994992 | 55994993 | 0.010139 | rs13328207 | 5.48 | rs182572 | 0.031 | 4.92 | 4.38987 | 415 | 1 | top1 | 0.031 | 1.70E-05 | 4.38987 | 1.13E-05 | 5.18E-03 |
| ENSG00000111846.16 | 6 | 10492222 | 10492223 | 0.158104 | rs504117 | -4.27 | rs504117 | 0.19 | -10.43 | -4.27112 | 526 | 526 | susie | 0.19 | 1.50E-27 | 4.2711 | 1.94E-05 | 7.32E-03 |
| ENSG00000124532.14 | 6 | 24402907 | 24402908 | 0.185706 | rs1054899 | -3.9 | rs793680 | 0.0938 | 8.17 | -3.16305 | 613 | 9 | enet | 0.1 | 1.20E-14 | -3.7894 | 1.51E-04 | 2.92E-02 |
| ENSG00000217083.1 | 6 | 24947879 | 24947880 | 0.029504 | rs12211739 | 5.94 | rs716797 | -0.00179 | -3.19 | -4.72271 | 653 | 1 | lasso | 5.00E-04 | 0.26 | 4.7227 | 2.33E-06 | 2.36E-03 |
| ENSG00000214975.4 | 6 | 24976418 | 24976419 | 0.163966 | rs12211739 | 5.94 | rs9461082 | 0.106 | 7.77 | 4.10298 | 639 | 5 | lasso | 0.15 | 5.90E-21 | 4.6209 | 3.82E-06 | 3.26E-03 |
| ENSG00000186577.12 | 6 | 34249469 | 34249470 | 0.011908 | rs10947435 | -13.03 | rs9368783 | -0.000431 | -3.53 | -7.32031 | 455 | 2 | lasso | 0.0044 | 0.064 | 7.3211 | 2.46E-13 | 2.00E-09 |
| ENSG00000007866.19 | 6 | 35497075 | 35497076 | 0.022674 | rs7744287 | -4.71 | rs9470056 | 0.0174 | -4.07 | -4.03348 | 430 | 1 | top1 | 0.017 | 0.0011 | 4.0335 | 5.50E-05 | 1.33E-02 |
| ENSG00000124496.12 | 6 | 42452050 | 42452051 | 0.026465 | rs4714591 | -3.75 | rs4714591 | -0.00124 | -3.06 | -3.74791 | 395 | 2 | lasso | 0.0087 | 0.016 | 3.7478 | 1.78E-04 | 3.11E-02 |
| ENSG00000183137.14 | 6 | 109095109 | 109095110 | -0.007162 | rs2768557 | 4.04 | rs2768557 | 0.0089 | -2.29 | 4.03881 | 411 | 1 | top1 | 0.0089 | 0.015 | -4.0388 | 5.37E-05 | 1.33E-02 |
| ENSG00000233231.1 | 6 | 166334567 | 166334568 | 0.100379 | rs9356478 | 4.5 | rs9356478 | 0.0342 | 5.05 | 4.50055 | 728 | 1 | top1 | 0.034 | 6.50E-06 | 4.5005 | 6.78E-06 | 4.78E-03 |
| ENSG00000198818.9 | 6 | 166342590 | 166342591 | 0.42471 | rs9356478 | 4.5 | rs9356478 | 0.268 | -12.29 | 4.50055 | 726 | 726 | susie | 0.35 | 1.80E-54 | -3.9119 | 9.16E-05 | 1.91E-02 |
| ENSG00000026297.15 | 6 | 166957183 | 166957184 | 0.517484 | rs3093023 | -5.88 | rs429083 | 0.485 | 16.46 | 4.55174 | 684 | 25 | enet | 0.49 | 1.90E-84 | 4.4501 | 8.58E-06 | 4.93E-03 |
| ENSG00000227598.1 | 6 | 166999064 | 166999065 | 0.053483 | rs3093023 | -5.88 | rs239935 | 0.0281 | 4.76 | 4.58804 | 631 | 1 | top1 | 0.028 | 4.00E-05 | 4.588 | 4.47E-06 | 3.45E-03 |
| ENSG00000112486.15 | 6 | 167111806 | 167111807 | 0.072269 | rs3093023 | -5.88 | rs3093026 | 0.0543 | -6.08 | 4.33948 | 541 | 541 | susie | 0.056 | 8.70E-09 | -4.5916 | 4.40E-06 | 3.45E-03 |
| ENSG00000185811.16 | 7 | 50304123 | 50304124 | 0.099335 | rs2886554 | -4.27 | rs9886239 | 0.079 | -6.88 | 3.71977 | 485 | 1 | top1 | 0.079 | 8.20E-12 | -3.71978 | 1.99E-04 | 3.28E-02 |
| ENSG00000157259.6 | 7 | 92447452 | 92447453 | -0.005972 | rs8179 | -4.12 | rs4272 | 0.00129 | -3.02 | -3.71003 | 307 | 1 | top1 | 0.0013 | 0.19 | 3.71003 | 2.07E-04 | 3.29E-02 |
| ENSG00000106244.12 | 7 | 99408639 | 99408640 | 0.030127 | rs6957987 | -3.88 | rs6955490 | -0.000171 | -3.53 | -3.37465 | 308 | 308 | susie | 0.0033 | 0.093 | 3.75869 | 1.71E-04 | 3.05E-02 |
| ENSG00000244219.6 | 7 | 99598065 | 99598066 | 0.028296 | rs6957987 | -3.88 | rs17161652 | 0.000436 | 2.77 | 2.0864 | 311 | 311 | susie | 0.011 | 0.0077 | 3.71424 | 2.04E-04 | 3.28E-02 |
| ENSG00000128604.19 | 7 | 128937611 | 128937612 | 0.448716 | rs13239597 | -6.51 | rs4728142 | 0.339 | 14.3 | -5.76 | 332 | 332 | susie | 0.43 | 5.00E-70 | -5.54246 | 2.98E-08 | 9.67E-05 |
| ENSG00000275106.1 | 7 | 128953315 | 128953316 | 0.257236 | rs13239597 | -6.51 | rs10239340 | 0.243 | 11.71 | 4.21475 | 330 | 1 | top1 | 0.24 | 8.70E-36 | 4.21475 | 2.50E-05 | 8.28E-03 |
| ENSG00000064419.13 | 7 | 129055172 | 129055173 | 0.434691 | rs13239597 | -6.51 | rs13227095 | 0.294 | -12.98 | -5.41477 | 351 | 351 | susie | 0.37 | 3.60E-57 | 3.98175 | 6.84E-05 | 1.56E-02 |
| ENSG00000230359.5 | 7 | 129055222 | 129055223 | 0.034969 | rs13239597 | -6.51 | rs13227095 | 0.0276 | -4.31 | -5.41477 | 351 | 1 | top1 | 0.028 | 4.80E-05 | 5.41477 | 6.14E-08 | 1.42E-04 |
| ENSG00000240204.2 | 7 | 129502478 | 129502479 | 0.015877 | rs13239597 | -6.51 | rs6960994 | -0.00176 | 3.03 | 3.12826 | 331 | 2 | lasso | 0.0023 | 0.13 | 3.56826 | 3.59E-04 | 4.55E-02 |
| ENSG00000154319.14 | 8 | 11474714 | 11474715 | 0.343094 | rs4840568 | -4.35 | rs4840568 | 0.28 | 12.6 | -4.34768 | 624 | 7 | lasso | 0.28 | 1.50E-42 | -4.32321 | 1.54E-05 | 6.41E-03 |
| ENSG00000136573.12 | 8 | 11494000 | 11494001 | 0.210441 | rs4840568 | -4.35 | rs998683 | 0.143 | -9.51 | -4.14572 | 629 | 18 | enet | 0.16 | 1.20E-22 | 4.1979 | 2.69E-05 | 8.52E-03 |
| ENSG00000255518.1 | 8 | 11557960 | 11557961 | 0.042366 | rs4840568 | -4.35 | rs2736340 | 0.0379 | -5.08 | -4.2253 | 602 | 1 | top1 | 0.038 | 2.20E-06 | 4.2253 | 2.39E-05 | 8.25E-03 |
| ENSG00000255354.1 | 8 | 11560019 | 11560020 | 0.14218 | rs4840568 | -4.35 | rs4840568 | 0.053 | -6.21 | -4.34768 | 601 | 601 | susie | 0.062 | 1.40E-09 | 4.42573 | 9.61E-06 | 5.03E-03 |
| ENSG00000137100.15 | 9 | 34620522 | 34620523 | 0.037483 | rs3793472 | -4.959 | rs3763615 | -0.000879 | -3.335 | 3.48259 | 364 | 364 | susie | 0.00049 | 0.26 | -4.21611 | 2.49E-05 | 8.28E-03 |
| ENSG00000165282.13 | 9 | 35096600 | 35096601 | 0.012389 | rs7024727 | 4.59 | rs3136658 | -0.000568 | -2.783 | 4.44593 | 337 | 1 | lasso | -0.00019 | 0.34 | -4.445934 | 8.75E-06 | 4.93E-03 |
| ENSG00000095261.13 | 9 | 120842983 | 120842984 | 0.04301 | rs10733648 | -4.601 | rs7037673 | 0.0133 | -4.709 | 3.41685 | 367 | 8 | enet | 0.027 | 5.10E-05 | -3.886924 | 1.02E-04 | 2.04E-02 |
| ENSG00000119403.13 | 9 | 120894895 | 120894896 | 0.026065 | rs10733648 | -4.601 | rs11794516 | 0.0128 | -4.211 | -3.86322 | 356 | 10 | enet | 0.02 | 0.00046 | 3.893766 | 9.87E-05 | 2.03E-02 |
| ENSG00000119396.10 | 9 | 121223013 | 121223014 | 0.006743 | rs10733648 | -4.601 | rs11794516 | 0.0054 | 2.843 | -3.86322 | 415 | 3 | lasso | 0.012 | 0.0048 | -3.82285 | 1.32E-04 | 2.61E-02 |
| ENSG00000235865.2 | 9 | 121285529 | 121285530 | 0.016512 | rs10733648 | -4.601 | rs2777317 | 0.00433 | -3.875 | -3.14805 | 406 | 406 | susie | 0.0061 | 0.036 | 4.066701 | 4.77E-05 | 1.27E-02 |
| ENSG00000204136.10 | 9 | 121500026 | 121500027 | 0.339242 | rs767769 | 4.128 | rs10985287 | 0.498 | -16.711 | -3.89886 | 438 | 11 | lasso | 0.5 | 1.40E-85 | 3.955035 | 7.65E-05 | 1.70E-02 |
| ENSG00000186193.8 | 9 | 137070587 | 137070588 | 0.415366 | rs4880201 | -3.74 | rs10870177 | 0.43 | -15.515 | -3.55493 | 365 | 1 | top1 | 0.43 | 4.50E-70 | 3.554929 | 3.78E-04 | 4.61E-02 |
| ENSG00000197355.10 | 9 | 137077500 | 137077501 | 0.511446 | rs4880201 | -3.74 | rs10870177 | 0.54 | -17.381 | -3.55493 | 367 | 1 | top1 | 0.54 | 4.20E-96 | 3.554929 | 3.78E-04 | 4.61E-02 |
| ENSG00000177239.14 | 9 | 137086926 | 137086927 | 0.33275 | rs4880201 | -3.74 | rs10870177 | 0.32 | -13.386 | -3.55493 | 368 | 368 | susie | 0.36 | 1.60E-55 | 3.60059 | 3.17E-04 | 4.18E-02 |
| ENSG00000183621.15 | 10 | 31031936 | 31031937 | 0.077705 | rs793108 | -4.79 | rs2256726 | 0.0428 | -6.25 | -2.714061 | 417 | 11 | enet | 0.072 | 7.40E-11 | 3.54244 | 3.96E-04 | 4.72E-02 |
| ENSG00000228403.1 | 10 | 48878021 | 48878022 | 0.080828 | rs17836435 | -4.18 | rs2663052 | 0.0245 | -5.28 | 4.153647 | 645 | 24 | enet | 0.036 | 3.80E-06 | -4.38667 | 1.15E-05 | 5.18E-03 |
| ENSG00000165806.19 | 10 | 113679161 | 113679162 | 0.347448 | rs7088038 | -3.95 | rs4353229 | 0.179 | -10.1 | -2.429744 | 598 | 598 | susie | 0.29 | 3.30E-43 | 3.93412 | 8.35E-05 | 1.77E-02 |
| ENSG00000148985.19 | 11 | 3797818 | 3797819 | 0.118246 | rs1451722 | 4.67 | rs1451722 | 0.0787 | 6.77 | 4.667 | 350 | 1 | top1 | 0.079 | 9.00E-12 | 4.667 | 3.06E-06 | 2.76E-03 |
| ENSG00000177105.9 | 11 | 3840982 | 3840983 | 0.23102 | rs1451722 | 4.67 | rs1451722 | 0.164 | 9.66 | 4.667 | 351 | 1 | top1 | 0.16 | 1.00E-23 | 4.667 | 3.06E-06 | 2.76E-03 |
| ENSG00000175213.2 | 11 | 46700817 | 46700818 | 0.004987 | rs4752927 | -4.37 | rs4606447 | -0.00168 | -2.69 | -3.71462 | 238 | 1 | lasso | 0.003 | 0.1 | 3.71462 | 2.04E-04 | 3.28E-02 |
| ENSG00000149179.13 | 11 | 46936688 | 46936689 | 0.041253 | rs4752927 | -4.37 | rs7128102 | -0.000415 | -3.31 | 3.15633 | 281 | 16 | enet | 0.0016 | 0.17 | -4.11276 | 3.91E-05 | 1.15E-02 |
| ENSG00000025434.18 | 11 | 47248299 | 47248300 | 0.059719 | rs2167079 | -4.36 | rs326222 | 0.016 | -4.04 | -4.30012 | 296 | 3 | enet | 0.031 | 1.80E-05 | 4.30162 | 1.70E-05 | 6.73E-03 |
| ENSG00000134575.9 | 11 | 47248905 | 47248906 | 0.169239 | rs2167079 | -4.36 | rs326222 | 0.167 | -9.85 | -4.30012 | 297 | 16 | enet | 0.18 | 2.60E-25 | 4.22666 | 2.37E-05 | 8.25E-03 |
| ENSG00000229719.5 | 11 | 64893448 | 64893449 | 0.003343 | rs547484 | -4.38 | rs6591858 | 0.00148 | -2.91 | -3.53207 | 338 | 1 | top1 | 0.0015 | 0.18 | 3.53207 | 4.12E-04 | 4.88E-02 |
| ENSG00000167283.7 | 11 | 118401153 | 118401154 | 0.032592 | rs548877 | -5.67 | rs2239695 | 0.0293 | 4.22 | -4.92121 | 482 | 1 | top1 | 0.029 | 2.90E-05 | -4.92121 | 8.60E-07 | 1.17E-03 |
| ENSG00000135414.9 | 12 | 55743279 | 55743280 | -0.000552 | rs10876870 | 4.78 | rs11171747 | -0.00119 | 2.74 | -4.35 | 308 | 2 | lasso | 0.0017 | 0.16 | -3.94 | 8.17E-05 | 1.77E-02 |
| ENSG00000123342.15 | 12 | 55842965 | 55842966 | 0.009638 | rs10876870 | 4.78 | rs12815387 | 0.00277 | 2.89 | 3.53 | 300 | 1 | top1 | 0.0028 | 0.11 | 3.53 | 4.18E-04 | 4.91E-02 |
| ENSG00000139531.12 | 12 | 55997179 | 55997180 | 0.216061 | rs10876870 | 4.78 | rs705700 | 0.179 | -10.23 | 3.92 | 293 | 45 | enet | 0.19 | 6.20E-27 | -4.11 | 3.98E-05 | 1.15E-02 |
| ENSG00000197728.9 | 12 | 56041852 | 56041853 | 0.597246 | rs10876870 | 4.78 | rs10876864 | 0.669 | 19.31 | 3.3 | 290 | 17 | enet | 0.68 | 3.40E-141 | 3.68 | 2.36E-04 | 3.45E-02 |
| ENSG00000170515.13 | 12 | 56104318 | 56104319 | 0.016935 | rs10876870 | 4.78 | rs2069391 | -0.000759 | 3.01 | 4.27 | 290 | 6 | enet | -0.00011 | 0.33 | 5.42 | 5.97E-08 | 1.42E-04 |
| ENSG00000135482.6 | 12 | 56118158 | 56118159 | 0.03218 | rs10876870 | 4.78 | rs10876840 | 0.00751 | 3.38 | -1.19 | 295 | 3 | lasso | 0.011 | 0.0074 | -3.63 | 2.79E-04 | 3.94E-02 |
| ENSG00000139641.12 | 12 | 56128055 | 56128056 | 0.041872 | rs10876870 | 4.78 | rs11171710 | -0.00167 | -2.78 | -4.73 | 299 | 299 | susie | 0.0075 | 0.023 | 3.77 | 1.66E-04 | 2.99E-02 |
| ENSG00000092841.18 | 12 | 56158160 | 56158161 | 0.057013 | rs10876870 | 4.78 | rs773649 | 0.00851 | 3.77 | 4.04 | 276 | 1 | top1 | 0.0085 | 0.017 | 4.04 | 5.41E-05 | 1.33E-02 |
| ENSG00000181852.17 | 12 | 56221932 | 56221933 | 0.006837 | rs10876870 | 4.78 | rs773643 | 0.00277 | -2.1 | 3.7 | 259 | 1 | top1 | 0.0028 | 0.11 | -3.7 | 2.15E-04 | 3.35E-02 |
| ENSG00000139579.12 | 12 | 56222014 | 56222015 | 0.050347 | rs10876870 | 4.78 | rs773643 | 0.0134 | -4.34 | 3.7 | 258 | 258 | susie | 0.017 | 0.001 | -3.93 | 8.39E-05 | 1.77E-02 |
| ENSG00000089009.15 | 12 | 112411441 | 112411442 | -0.005706 | rs17630235 | -7.06 | rs232925 | -0.000891 | -2.18 | -4.19 | 283 | 1 | top1 | -0.00089 | 0.48 | 4.19 | 2.73E-05 | 8.52E-03 |
| ENSG00000224429.7 | 13 | 21348720 | 21348721 | 0.280145 | rs4770192 | 3.18 | rs35811168 | 0.0477 | 7.13 | 2.91749 | 468 | 35 | enet | 0.17 | 3.30E-24 | 4.11235 | 3.92E-05 | 1.15E-02 |
| ENSG00000133103.16 | 13 | 39655626 | 39655627 | 0.133953 | rs4129745 | 6.49 | rs4254189 | 0.067 | -7.1 | 6.24976 | 524 | 524 | susie | 0.15 | 4.20E-21 | -3.96026 | 7.49E-05 | 1.69E-02 |
| ENSG00000072042.12 | 14 | 67695813 | 67695814 | 0.07673 | rs12433356 | 4.41 | rs742869 | 0.0158 | -4.6 | -3.72486 | 361 | 1 | top1 | 0.016 | 0.0017 | 3.72486 | 1.95E-04 | 3.26E-02 |
| ENSG00000182185.18 | 14 | 67819778 | 67819779 | 0.001748 | rs1957570 | 4.79 | rs4902562 | -0.00156 | 2.95 | -3.31649 | 366 | 9 | enet | 0.00082 | 0.23 | -3.74481 | 1.81E-04 | 3.12E-02 |
| ENSG00000119718.10 | 14 | 75002910 | 75002911 | 0.163887 | rs175435 | -3.79 | rs175040 | 0.202 | -10.92 | -3.63528 | 413 | 413 | susie | 0.21 | 1.70E-30 | 3.60313 | 3.14E-04 | 4.18E-02 |
| ENSG00000119684.15 | 14 | 75051531 | 75051532 | 0.02786 | rs175714 | -4.06 | rs175071 | 0.0365 | -4.84 | -3.73836 | 409 | 1 | top1 | 0.036 | 3.30E-06 | 3.73836 | 1.85E-04 | 3.16E-02 |
| ENSG00000278493.1 | 15 | 41908713 | 41908714 | 0.006099 | rs7171675 | 3.86 | rs8043408 | -0.000706 | 3.18 | -2.37847 | 426 | 426 | susie | 0.0056 | 0.042 | -3.61775 | 2.97E-04 | 4.05E-02 |
| ENSG00000269951.1 | 15 | 77068324 | 77068325 | 0.090685 | rs11639079 | -3.82 | rs11639079 | 0.0225 | 5.59 | -3.82233 | 251 | 13 | enet | 0.045 | 2.70E-07 | -3.65362 | 2.59E-04 | 3.72E-02 |
| ENSG00000140391.14 | 15 | 77083983 | 77083984 | 0.526264 | rs11639079 | -3.82 | rs11639197 | 0.138 | 9.7 | -3.81098 | 255 | 39 | enet | 0.28 | 1.90E-41 | -3.78346 | 1.55E-04 | 2.92E-02 |
| ENSG00000262222.1 | 16 | 10940718 | 10940719 | 0.052848 | rs11074938 | 4.34 | rs11074938 | -0.00174 | -3.52 | 4.341599 | 555 | 3 | lasso | 0.0014 | 0.18 | -3.565476 | 3.63E-04 | 4.57E-02 |
| ENSG00000233232.6 | 16 | 28471174 | 28471175 | 0.104268 | rs11646653 | -4.74 | rs34835 | 0.157 | -9.58 | -4.146913 | 213 | 1 | top1 | 0.16 | 1.40E-22 | 4.146913 | 3.37E-05 | 1.03E-02 |
| ENSG00000275441.1 | 16 | 28554139 | 28554140 | 0.007837 | rs11646653 | -4.74 | rs149299 | -0.00127 | 2.51 | -4.094207 | 196 | 1 | lasso | -0.00077 | 0.45 | -4.094207 | 4.24E-05 | 1.17E-02 |
| ENSG00000197165.10 | 16 | 28597108 | 28597109 | 0.391281 | rs11646653 | -4.74 | rs3859172 | 0.585 | 18.04 | -4.057998 | 186 | 29 | enet | 0.6 | 8.30E-112 | -4.259588 | 2.05E-05 | 7.56E-03 |
| ENSG00000184110.14 | 16 | 28688557 | 28688558 | 0.043578 | rs11646653 | -4.74 | rs149299 | 0.0358 | 5.35 | -4.094207 | 201 | 201 | susie | 0.036 | 3.90E-06 | -4.427605 | 9.53E-06 | 5.03E-03 |
| ENSG00000168488.18 | 16 | 28824137 | 28824138 | 0.092075 | rs11646653 | -4.74 | rs2008514 | 0.0805 | -6.98 | -4.3146 | 181 | 1 | top1 | 0.081 | 5.10E-12 | 4.3146 | 1.60E-05 | 6.49E-03 |
| ENSG00000178952.10 | 16 | 28846407 | 28846408 | 0.376634 | rs11646653 | -4.74 | rs8049439 | 0.574 | 18.02 | -4.299124 | 182 | 35 | enet | 0.58 | 6.30E-107 | -4.198805 | 2.68E-05 | 8.52E-03 |
| ENSG00000178188.14 | 16 | 28846599 | 28846600 | 0.022713 | rs11646653 | -4.74 | rs8062405 | 0.0142 | -3.97 | -4.333301 | 182 | 1 | top1 | 0.014 | 0.0028 | 4.333301 | 1.47E-05 | 6.28E-03 |
| ENSG00000196296.13 | 16 | 28878404 | 28878405 | 0.029187 | rs11646653 | -4.74 | rs12325113 | 0.0145 | 3.89 | -4.386997 | 182 | 182 | susie | 0.017 | 0.001 | -4.388722 | 1.14E-05 | 5.18E-03 |
| ENSG00000102882.11 | 16 | 30123505 | 30123506 | 0.160804 | rs4238961 | 3.81 | rs2005219 | 0.205 | 11.49 | 3.611986 | 227 | 15 | enet | 0.26 | 3.50E-38 | 3.647815 | 2.64E-04 | 3.76E-02 |
| ENSG00000268218.1 | 16 | 89269938 | 89269939 | 0.025241 | rs3114891 | 4.98 | rs3096294 | 0.0231 | -4.66 | 3.176161 | 393 | 6 | enet | 0.033 | 8.50E-06 | -4.006196 | 6.17E-05 | 1.45E-02 |
| ENSG00000167522.14 | 16 | 89490560 | 89490561 | 0.123225 | rs3114891 | 4.98 | rs2965939 | 0.097 | -8.05 | 4.124936 | 420 | 16 | enet | 0.11 | 1.10E-15 | -4.903763 | 9.40E-07 | 1.17E-03 |
| ENSG00000003249.13 | 16 | 90020042 | 90020043 | 0.13958 | rs457372 | -3.92 | rs4442808 | 0.0787 | 7.64 | 3.534656 | 275 | 5 | lasso | 0.1 | 2.60E-15 | 3.718994 | 2.00E-04 | 3.28E-02 |
| ENSG00000175826.11 | 17 | 7252022 | 7252023 | 0.168719 | rs12935952 | -4.16 | rs2654185 | 0.142 | -8.95 | -3.68649 | 468 | 1 | top1 | 0.14 | 1.80E-20 | 3.68649 | 2.27E-04 | 3.41E-02 |
| ENSG00000033627.16 | 17 | 42458843 | 42458844 | 0.069211 | rs1024091 | 4.55 | rs3760387 | 0.0133 | 4.54 | -3.05703 | 251 | 251 | susie | 0.024 | 0.00015 | -3.77197 | 1.62E-04 | 2.97E-02 |
| ENSG00000108786.10 | 17 | 42549213 | 42549214 | 0.104536 | rs1024091 | 4.55 | rs2676531 | 0.0954 | 7.54 | 4.41505 | 249 | 5 | lasso | 0.1 | 3.90E-15 | 4.44456 | 8.81E-06 | 4.93E-03 |
| ENSG00000037042.8 | 17 | 42659304 | 42659305 | 0.062216 | rs1024091 | 4.55 | rs9897724 | 0.0106 | -3.67 | 3.78542 | 257 | 1 | top1 | 0.011 | 0.0085 | -3.78542 | 1.53E-04 | 2.92E-02 |
| ENSG00000131467.10 | 17 | 42824384 | 42824385 | 0.007952 | rs1024091 | 4.55 | rs2097219 | -0.00169 | 2.54 | -3.09874 | 232 | 232 | susie | -0.0015 | 0.7 | -4.83167 | 1.35E-06 | 1.46E-03 |
| ENSG00000264920.1 | 17 | 47895811 | 47895812 | 0.145447 | rs12939739 | -3.45 | rs1130932 | 0.0302 | 5.68 | -3.39796 | 379 | 14 | enet | 0.062 | 1.40E-09 | -3.89057 | 1.00E-04 | 2.03E-02 |
| ENSG00000267654.1 | 18 | 12749420 | 12749421 | 0.388328 | rs2847297 | -3.78 | rs2542157 | 0.163 | -10.13 | 2.53216 | 394 | 11 | lasso | 0.22 | 9.50E-33 | -3.54605 | 3.91E-04 | 4.72E-02 |
| ENSG00000152217.16 | 18 | 44680172 | 44680173 | -0.00501 | rs7238082 | 3.63 | rs7238082 | 0.00185 | 3.37 | 3.6264 | 413 | 1 | top1 | 0.0018 | 0.15 | 3.62639 | 2.87E-04 | 4.01E-02 |
| ENSG00000105376.4 | 19 | 10289980 | 10289981 | 0.043034 | rs2304256 | 5.35 | rs12720356 | 0.0366 | 4.74 | 4.906334 | 379 | 1 | top1 | 0.037 | 3.10E-06 | 4.90633 | 9.28E-07 | 1.17E-03 |
| ENSG00000161847.13 | 19 | 10333434 | 10333435 | 0.056236 | rs2304256 | 5.35 | rs12720356 | 0.0127 | 4.37 | 4.906334 | 382 | 1 | top1 | 0.013 | 0.0045 | 4.90633 | 9.28E-07 | 1.17E-03 |
| ENSG00000105397.13 | 19 | 10380675 | 10380676 | 0.13208 | rs2304256 | 5.35 | rs2304256 | 0.0553 | 6.83 | 5.346311 | 375 | 14 | enet | 0.088 | 4.80E-13 | 4.4896 | 7.14E-06 | 4.83E-03 |
| ENSG00000213339.8 | 19 | 10701450 | 10701451 | 0.016848 | rs2304256 | 5.35 | rs281413 | -0.00154 | -3.02 | -3.558438 | 379 | 2 | lasso | 0.0029 | 0.11 | 4.28175 | 1.85E-05 | 7.15E-03 |
| ENSG00000099203.6 | 19 | 10836317 | 10836318 | 0.067048 | rs2304256 | 5.35 | rs10775614 | 0.012 | -4.93 | 0.885833 | 386 | 4 | lasso | 0.031 | 1.70E-05 | -3.69036 | 2.24E-04 | 3.40E-02 |
| ENSG00000207166.1 | 19 | 17862587 | 17862588 | 0.002283 | rs11554159 | 4.52 | rs885683 | -0.00163 | -3.1 | 3.798934 | 429 | 1 | lasso | -0.00078 | 0.45 | -3.79893 | 1.45E-04 | 2.83E-02 |
| ENSG00000254858.9 | 19 | 18193181 | 18193182 | 0.073492 | rs11554159 | 4.52 | rs2271881 | 0.0341 | 5.61 | 4.506464 | 420 | 1 | top1 | 0.034 | 6.80E-06 | 4.50646 | 6.59E-06 | 4.78E-03 |
| ENSG00000130518.16 | 19 | 18274508 | 18274509 | 0.233699 | rs11554159 | 4.52 | rs12610691 | 0.164 | 10.16 | 4.12932 | 410 | 410 | susie | 0.23 | 3.20E-33 | 3.73199 | 1.90E-04 | 3.21E-02 |
| ENSG00000159915.12 | 19 | 44259879 | 44259880 | -0.017699 | rs2927438 | 2.8 | rs2927438 | -0.00179 | -3.29 | 2.799251 | 467 | 13 | enet | -0.00064 | 0.42 | -3.52487 | 4.24E-04 | 4.95E-02 |
| ENSG00000249210.1 | 19 | 46558640 | 46558641 | 0.041533 | rs2110577 | 3.16 | rs8101216 | -0.000615 | -3.33 | 1.257296 | 369 | 369 | susie | 0.0092 | 0.013 | -3.60507 | 3.12E-04 | 4.18E-02 |
| ENSG00000101019.21 | 20 | 35412140 | 35412141 | 0.100859 | rs6060435 | 4.38 | rs1540927 | 0.113 | -8.16 | 3.691628 | 330 | 1 | top1 | 0.11 | 2.30E-16 | -3.691628 | 2.23E-04 | 3.40E-02 |
| ENSG00000125510.15 | 20 | 64080172 | 64080173 | 0.207532 | rs8121509 | -5.07 | rs8121509 | 0.127 | -9.11 | -5.06646 | 312 | 4 | lasso | 0.15 | 1.70E-21 | 4.471003 | 7.79E-06 | 4.93E-03 |
| ENSG00000278558.4 | 22 | 18527801 | 18527802 | 0.011742 | rs17810512 | 3.58 | rs17810512 | 0.00185 | 3.31 | 3.58 | 123 | 1 | top1 | 0.0018 | 0.15 | 3.582 | 3.41E-04 | 4.39E-02 |
| ENSG00000100316.15 | 22 | 39320388 | 39320389 | 0.030634 | rs12627761 | -4.45 | rs5757582 | -0.000882 | -3.52 | -3.24 | 394 | 2 | lasso | 0.0012 | 0.19 | 3.98701 | 6.69E-05 | 1.55E-02 |
| ENSG00000100321.14 | 22 | 39349924 | 39349925 | 0.284668 | rs12627761 | -4.45 | rs2069235 | 0.18 | 10.96 | -4.08 | 385 | 15 | enet | 0.23 | 5.50E-34 | -4.83575 | 1.33E-06 | 1.46E-03 |

**Supplemental Table S17. Candidate genes in blood associated with RA (replication) using TWAS.**

| **ID** | **CHR** | **Start** | **End** | **HSQ** | **BEST.GWAS.ID** | **BEST.GWAS.Z** | **EQTL.ID** | **EQTL.R2** | **EQTL.Z** | **EQTL.GWAS.Z** | **NSNP** | **NWGT** | **MODEL** | **MODELCV.R2** | **MODELCV.PV** | **TWAS.Z** | **TWAS.P** | **padj** |
| --- | --- | --- | --- | --- | --- | --- | --- | --- | --- | --- | --- | --- | --- | --- | --- | --- | --- | --- |
| ENSG00000157870.14 | 1 | 2586490 | 2586491 | 0.1339 | rs2843401 | -5.888 | rs4445406 | 0.105 | -9.05 | -5.22947 | 366 | 24 | enet | 0.16 | 8.60E-24 | 4.33052 | 1.49E-05 | 3.67E-03 |
| ENSG00000142606.15 | 1 | 2632989 | 2632990 | 0.1636 | rs2843401 | -5.888 | rs3748816 | 0.126 | -9.37 | -5.5535 | 356 | 32 | enet | 0.19 | 4.80E-27 | 4.16017 | 3.18E-05 | 6.75E-03 |
| ENSG00000215912.12 | 1 | 2801716 | 2801717 | 0.0673 | rs2843401 | -5.888 | rs4648356 | 0.0699 | 6.88 | -3.75987 | 369 | 369 | susie | 0.073 | 5.40E-11 | -5.20078 | 1.98E-07 | 1.04E-04 |
| ENSG00000159363.17 | 1 | 17011927 | 17011928 | 0.0808 | rs2240335 | -5.782 | rs2076600 | 0.0297 | -5.11 | -3.33927 | 344 | 344 | susie | 0.034 | 7.20E-06 | 3.80956 | 0.000139 | 2.24E-02 |
| ENSG00000204084.12 | 1 | 37947056 | 37947057 | 0.4959 | rs28411352 | 5.841 | rs36084352 | 0.417 | -15.31 | -4.01281 | 459 | 8 | lasso | 0.46 | 8.20E-76 | 4.98186 | 6.30E-07 | 2.57E-04 |
| ENSG00000081985.10 | 1 | 67307363 | 67307364 | 0.2702 | rs6679356 | 4.587 | rs17129794 | 0.191 | 10.42 | 4.4399 | 498 | 498 | susie | 0.19 | 8.10E-28 | 4.13326 | 3.58E-05 | 7.24E-03 |
| ENSG00000232450.1 | 1 | 113699630 | 113699631 | 0.0912 | rs2476601 | 26.043 | rs6669008 | 0.0224 | -5.22 | -6.59335 | 486 | 2 | lasso | 0.034 | 6.90E-06 | 5.29191 | 1.21E-07 | 7.34E-05 |
| ENSG00000134242.15 | 1 | 113871758 | 113871759 | 0.0427 | rs2476601 | 26.043 | rs2279380 | -0.00116 | 3.6 | 2.22621 | 483 | 15 | enet | 0.008 | 0.02 | 6.74693 | 1.51E-11 | 5.96E-08 |
| ENSG00000160856.20 | 1 | 157700856 | 157700857 | 0.1746 | rs3761959 | 4.378 | rs3761959 | 0.263 | 12.18 | 4.37759 | 537 | 1 | top1 | 0.26 | 6.50E-39 | 4.37759 | 1.20E-05 | 3.05E-03 |
| ENSG00000163534.14 | 1 | 157820104 | 157820105 | 0.1356 | rs3761959 | 4.378 | rs6689427 | 0.115 | -8.28 | 3.71902 | 500 | 5 | lasso | 0.13 | 2.50E-19 | -4.2573 | 2.07E-05 | 4.78E-03 |
| ENSG00000225217.1 | 1 | 161606290 | 161606291 | 0.3608 | rs1801274 | 5.327 | rs12142755 | 0.0209 | -5.96 | 1.73354 | 449 | 45 | enet | 0.15 | 1.80E-21 | -3.62932 | 0.000284 | 3.67E-02 |
| ENSG00000072694.20 | 1 | 161663146 | 161663147 | 0.4366 | rs1801274 | 5.327 | rs35835689 | 0.159 | 9.57 | 2.95764 | 477 | 46 | enet | 0.21 | 4.20E-31 | 3.77007 | 0.000163 | 2.43E-02 |
| ENSG00000162927.13 | 2 | 61018160 | 61018161 | 0.1044 | rs34695944 | 7.55 | rs35217978 | 0.0108 | 5.35 | 4.0751 | 324 | 11 | enet | 0.014 | 0.0032 | 5.33165 | 9.73E-08 | 6.40E-05 |
| ENSG00000152284.4 | 2 | 85133409 | 85133410 | 0.1212 | rs10460586 | 3.94 | rs1053560 | 0.102 | -8.01 | 3.7911 | 411 | 9 | lasso | 0.11 | 1.20E-15 | -3.77911 | 0.000157 | 2.38E-02 |
| ENSG00000246575.2 | 2 | 85315040 | 85315041 | 0.1435 | rs10460586 | 3.94 | rs1053560 | 0.11 | -8.31 | 3.7911 | 446 | 446 | susie | 0.11 | 1.20E-16 | -3.79253 | 0.000149 | 2.30E-02 |
| ENSG00000228486.10 | 2 | 97664216 | 97664217 | 0.1167 | rs12989231 | 4.16 | rs298913 | 0.0135 | 4.22 | 2.7114 | 143 | 40 | enet | 0.04 | 1.10E-06 | 4.01302 | 5.99E-05 | 1.12E-02 |
| ENSG00000144218.18 | 2 | 100142738 | 100142739 | 0.0708 | rs9653442 | 6.95 | rs2309811 | 0.0154 | 5.35 | 4.4907 | 456 | 456 | susie | 0.037 | 3.30E-06 | 5.56183 | 2.67E-08 | 2.34E-05 |
| ENSG00000123609.10 | 2 | 151290056 | 151290057 | 0.0584 | rs446791 | -3.66 | rs4664349 | 0.038 | 5.87 | 2.6606 | 448 | 2 | lasso | 0.047 | 1.30E-07 | 3.58885 | 0.000332 | 3.91E-02 |
| ENSG00000115896.15 | 2 | 197804701 | 197804702 | 0.2725 | rs1065953 | -4.25 | rs1579695 | 0.226 | 11.53 | -3.775 | 308 | 308 | susie | 0.23 | 6.90E-34 | -3.70037 | 0.000215 | 2.87E-02 |
| ENSG00000250526.1 | 5 | 14641017 | 14641018 | 0.0617 | rs13189930 | 4.74 | rs31929 | 0.0311 | -4.95 | 3.8674 | 467 | 1 | top1 | 0.031 | 1.70E-05 | -3.8674 | 0.00011 | 1.85E-02 |
| ENSG00000145723.16 | 5 | 103120147 | 103120148 | 0.0312 | rs35801 | -6.18 | rs26262 | 0.0129 | -3.97 | -3.8461 | 318 | 318 | susie | 0.021 | 0.00031 | 3.90066 | 9.59E-05 | 1.64E-02 |
| ENSG00000112486.15 | 6 | 167111806 | 167111807 | 0.0723 | rs3093024 | 7.62 | rs3093026 | 0.054261 | -6.08 | -6.26619 | 541 | 541 | susie | 0.056 | 8.70E-09 | 6.238857 | 4.41E-10 | 8.70E-07 |
| ENSG00000128604.19 | 7 | 128937611 | 128937612 | 0.4487 | rs10488631 | 6.77 | rs4728142 | 0.339 | 14.3 | 5.861 | 332 | 332 | susie | 0.43 | 5.00E-70 | 5.673132 | 1.40E-08 | 1.84E-05 |
| ENSG00000275106.1 | 7 | 128953315 | 128953316 | 0.2572 | rs10488631 | 6.77 | rs10239340 | 0.243 | 11.71 | -4.3965 | 330 | 1 | top1 | 0.24 | 8.70E-36 | -4.39652 | 1.10E-05 | 2.89E-03 |
| ENSG00000154319.14 | 8 | 11474714 | 11474715 | 0.3431 | rs13277113 | 5.22 | rs4840568 | 0.28 | 12.6 | 4.91452 | 624 | 7 | lasso | 0.28 | 1.50E-42 | 4.6952 | 2.66E-06 | 8.74E-04 |
| ENSG00000136573.12 | 8 | 11494000 | 11494001 | 0.2104 | rs13277113 | 5.22 | rs998683 | 0.143 | -9.51 | 4.825 | 629 | 18 | enet | 0.16 | 1.20E-22 | -4.2637 | 2.01E-05 | 4.78E-03 |
| ENSG00000255518.1 | 8 | 11557960 | 11557961 | 0.0424 | rs13277113 | 5.22 | rs2736340 | 0.0379 | -5.08 | 5.18159 | 602 | 1 | top1 | 0.038 | 2.20E-06 | -5.1816 | 2.20E-07 | 1.08E-04 |
| ENSG00000255354.1 | 8 | 11560019 | 11560020 | 0.1422 | rs13277113 | 5.22 | rs4840568 | 0.053 | -6.21 | 4.91452 | 601 | 601 | susie | 0.062 | 1.40E-09 | -5.2604 | 1.44E-07 | 8.11E-05 |
| ENSG00000184428.12 | 8 | 143359978 | 143359979 | 0.1556 | rs878238 | 3.32 | rs3814772 | 0.00537 | -5.21 | -2.13208 | 369 | 23 | enet | 0.053 | 2.40E-08 | 3.9061 | 9.38E-05 | 1.64E-02 |
| ENSG00000095261.13 | 9 | 120842983 | 120842984 | 0.043 | rs10818482 | 5.62 | rs7037673 | 0.0133 | -4.71 | -4.41717 | 368 | 8 | enet | 0.027 | 5.10E-05 | 3.7389 | 0.000185 | 2.65E-02 |
| ENSG00000119403.13 | 9 | 120894895 | 120894896 | 0.0261 | rs10818482 | 5.62 | rs11794516 | 0.0128 | -4.21 | 5.42181 | 357 | 10 | enet | 0.02 | 0.00046 | -5.1364 | 2.80E-07 | 1.30E-04 |
| ENSG00000056558.10 | 9 | 120929172 | 120929173 | 0.2339 | rs10818482 | 5.62 | rs10760130 | 0.0717 | 6.6 | 5.32672 | 347 | 54 | enet | 0.085 | 1.30E-12 | 4.1552 | 3.25E-05 | 6.75E-03 |
| ENSG00000122378.13 | 10 | 80407828 | 80407829 | 0.0995 | rs2343306 | 4.47 | rs12220642 | 0.0854 | -8.25 | 3.97629 | 547 | 12 | enet | 0.11 | 3.10E-16 | -3.7146 | 0.000204 | 2.82E-02 |
| ENSG00000134825.15 | 11 | 61792635 | 61792636 | 0.0432 | rs968567 | -4.95 | rs174538 | 0.035791 | 5.53 | -3.6623 | 401 | 6 | enet | 0.044 | 3.70E-07 | -4.7665 | 1.87E-06 | 6.41E-04 |
| ENSG00000134824.13 | 11 | 61816255 | 61816256 | 0.7034 | rs968567 | -4.95 | rs968567 | 0.619193 | 18.57 | -4.9506 | 414 | 6 | lasso | 0.63 | 5.70E-121 | -4.9755 | 6.51E-07 | 2.57E-04 |
| ENSG00000149485.18 | 11 | 61829317 | 61829318 | 0.0505 | rs968567 | -4.95 | rs102275 | 0.05024 | 5.61 | -3.3426 | 421 | 3 | lasso | 0.06 | 2.90E-09 | -4.552 | 5.31E-06 | 1.55E-03 |
| ENSG00000110367.11 | 11 | 118791146 | 118791147 | 0.1028 | rs4938573 | -7.76 | rs2077579 | 0.03069 | -5.61 | -7.5983 | 363 | 363 | susie | 0.094 | 7.00E-14 | 3.8028 | 0.000143 | 2.26E-02 |
| ENSG00000139531.12 | 12 | 55997179 | 55997180 | 0.2161 | rs705699 | -5.33 | rs705700 | 0.179 | -10.23 | -5.2935 | 293 | 45 | enet | 0.19 | 6.20E-27 | 5.36296 | 8.19E-08 | 5.87E-05 |
| ENSG00000197728.9 | 12 | 56041852 | 56041853 | 0.5972 | rs705699 | -5.33 | rs10876864 | 0.669 | 19.31 | -4.78616 | 290 | 17 | enet | 0.68 | 3.40E-141 | -5.0614 | 4.16E-07 | 1.82E-04 |
| ENSG00000092841.18 | 12 | 56158160 | 56158161 | 0.057 | rs705699 | -5.33 | rs773649 | 0.00851 | 3.77 | -3.6068 | 276 | 1 | top1 | 0.0085 | 0.017 | -3.6068 | 0.00031 | 3.76E-02 |
| ENSG00000135452.9 | 12 | 57739735 | 57739736 | 0.0439 | rs238516 | -5.22 | rs701008 | 0.0266 | -5.21 | -4.40146 | 341 | 1 | top1 | 0.027 | 6.50E-05 | 4.40146 | 1.08E-05 | 2.89E-03 |
| ENSG00000123427.16 | 12 | 57771605 | 57771606 | 0.3498 | rs238516 | -5.22 | rs10877016 | 0.375 | 14.61 | -4.4573 | 347 | 347 | susie | 0.38 | 6.60E-60 | -4.4036 | 1.06E-05 | 2.89E-03 |
| ENSG00000259703.5 | 15 | 69835233 | 69835234 | 0.0651 | rs8026898 | 8.47 | rs4777182 | 0.0167 | -4.05 | -4.54475 | 533 | 20 | enet | 0.028 | 3.80E-05 | 4.06704 | 4.76E-05 | 9.16E-03 |
| ENSG00000178952.10 | 16 | 28846407 | 28846408 | 0.3766 | rs7500321 | 4.45 | rs8049439 | 0.574 | 18.02 | 3.745549 | 182 | 35 | enet | 0.58 | 6.30E-107 | 3.66085 | 0.000251 | 3.30E-02 |
| ENSG00000178188.14 | 16 | 28846599 | 28846600 | 0.0227 | rs7500321 | 4.45 | rs8062405 | 0.0142 | -3.97 | 3.719016 | 182 | 1 | top1 | 0.014 | 0.0028 | -3.71902 | 2.00E-04 | 2.82E-02 |
| ENSG00000169682.17 | 16 | 28975088 | 28975089 | 0.5088 | rs7500321 | 4.45 | rs8045689 | 0.36 | 14.21 | 4.165993 | 183 | 183 | susie | 0.38 | 4.80E-60 | 4.15706 | 3.22E-05 | 6.75E-03 |
| ENSG00000260114.2 | 16 | 29921904 | 29921905 | 0.0491 | rs4787495 | 4.02 | rs4238961 | 0.00817 | 4.47 | -2.716381 | 181 | 14 | enet | 0.025 | 1.00E-04 | -4.00927 | 6.09E-05 | 1.12E-02 |
| ENSG00000183604.14 | 16 | 30335373 | 30335374 | 0.1175 | rs8058578 | 4.67 | rs9924308 | 0.107 | -8.18 | 3.826459 | 209 | 209 | susie | 0.11 | 9.30E-16 | -3.60216 | 0.000316 | 3.78E-02 |
| ENSG00000103549.21 | 16 | 30763117 | 30763118 | 0.1415 | rs8058578 | 4.67 | rs8058578 | 0.0623 | 6.39 | 4.67082 | 209 | 209 | susie | 0.09 | 2.60E-13 | 3.85988 | 0.000113 | 1.86E-02 |
| ENSG00000262903.1 | 17 | 3658003 | 3658004 | 0.051 | rs11657606 | -3.78 | rs220488 | -0.00105 | -3.3 | -2.4838 | 528 | 6 | lasso | 0.004 | 0.072 | 3.90108 | 9.58E-05 | 1.64E-02 |
| ENSG00000161395.13 | 17 | 39696796 | 39696797 | 0.0359 | rs2872507 | 5.9 | rs3816470 | -0.0018 | 3.35 | -5.4661 | 318 | 318 | susie | 0.0024 | 0.13 | -4.63425 | 3.58E-06 | 1.09E-03 |
| ENSG00000161405.16 | 17 | 39864187 | 39864188 | 0.0593 | rs2872507 | 5.9 | rs907091 | 0.0599 | -6.53 | -5.4953 | 291 | 291 | susie | 0.061 | 1.80E-09 | 5.58471 | 2.34E-08 | 2.31E-05 |
| ENSG00000073605.18 | 17 | 39918649 | 39918650 | 0.3089 | rs2872507 | 5.9 | rs8067378 | 0.392 | 14.83 | -5.4953 | 290 | 290 | susie | 0.42 | 4.40E-68 | -5.43983 | 5.33E-08 | 4.20E-05 |
| ENSG00000172057.9 | 17 | 39927600 | 39927601 | 0.2158 | rs2872507 | 5.9 | rs8067378 | 0.265 | 13.07 | -5.4953 | 292 | 16 | enet | 0.33 | 2.50E-51 | -5.64592 | 1.64E-08 | 1.85E-05 |
| ENSG00000267654.1 | 18 | 12749420 | 12749421 | 0.3883 | rs12971201 | -5.84 | rs2542157 | 0.16284 | -10.13 | -4.825 | 394 | 11 | lasso | 0.22 | 9.50E-33 | 4.8766 | 1.08E-06 | 3.87E-04 |
| ENSG00000260302.1 | 18 | 12775922 | 12775923 | 0.2899 | rs12971201 | -5.84 | rs2542157 | 0.18113 | -10.08 | -4.825 | 385 | 30 | enet | 0.19 | 6.20E-28 | 4.6502 | 3.32E-06 | 1.05E-03 |
| ENSG00000105397.13 | 19 | 10380675 | 10380676 | 0.1321 | rs2304256 | -6.47 | rs2304256 | 0.0553 | 6.83 | -6.46695 | 376 | 14 | enet | 0.088 | 4.80E-13 | -6.94254 | 3.85E-12 | 3.04E-08 |
| ENSG00000178093.13 | 19 | 19515684 | 19515685 | 0.0731 | rs11085264 | 4.24 | rs998732 | 0.0484 | 5.71 | 3.75987 | 356 | 1 | top1 | 0.048 | 9.10E-08 | 3.75987 | 0.00017 | 2.48E-02 |
| ENSG00000088298.12 | 20 | 35147363 | 35147364 | 0.0697 | rs3746429 | -3.6 | rs3746429 | 0.0467 | -6.19 | -3.59855 | 345 | 5 | enet | 0.051 | 4.70E-08 | 3.618 | 0.000297 | 3.70E-02 |
| ENSG00000124203.6 | 20 | 59123380 | 59123381 | 0.0699 | rs259956 | 3.64 | rs259964 | 0.0233 | -4.4 | 3.6153 | 537 | 1 | top1 | 0.023 | 0.00018 | -3.6153 | 3.00E-04 | 3.70E-02 |
| ENSG00000124205.15 | 20 | 59300426 | 59300427 | 0.0986 | rs259956 | 3.64 | rs259964 | 0.0688 | -6.46 | 3.6153 | 554 | 1 | top1 | 0.069 | 1.90E-10 | -3.6153 | 3.00E-04 | 3.70E-02 |
| ENSG00000125510.15 | 20 | 64080172 | 64080173 | 0.2075 | rs4408777 | 3.48 | rs8121509 | 0.127 | -9.11 | 3.40568 | 312 | 4 | lasso | 0.15 | 1.70E-21 | -3.706 | 0.000211 | 2.87E-02 |
| ENSG00000159128.14 | 21 | 33402895 | 33402896 | 0.081 | rs11702844 | -5.33 | rs9808753 | 0.0783 | 6.71 | -4.89164 | 463 | 1 | top1 | 0.078 | 1.00E-11 | -4.8916 | 1.00E-06 | 3.76E-04 |
| ENSG00000142188.16 | 21 | 33480010 | 33480011 | 0.1463 | rs11702844 | -5.33 | rs2284555 | 0.183 | -10.23 | -4.09074 | 458 | 458 | susie | 0.18 | 1.40E-26 | 4.1211 | 3.77E-05 | 7.44E-03 |
| ENSG00000160185.14 | 21 | 42403446 | 42403447 | 0.3338 | rs1893592 | -5.73 | rs1893592 | 0.265 | 12.19 | -5.73415 | 759 | 1 | top1 | 0.27 | 2.60E-39 | -5.7342 | 9.80E-09 | 1.55E-05 |
| ENSG00000185651.14 | 22 | 21567546 | 21567547 | 0.1104 | rs11089637 | 5 | rs4821130 | 0.107 | 8.48 | 4.31445 | 286 | 286 | susie | 0.12 | 5.40E-17 | 4.25171 | 2.12E-05 | 4.78E-03 |
| ENSG00000100221.10 | 22 | 38701555 | 38701556 | 0.0587 | rs6001251 | -3.63 | rs5750668 | 0.0282 | -5.35 | 2.68284 | 374 | 16 | enet | 0.046 | 1.90E-07 | -3.54019 | 4.00E-04 | 4.64E-02 |
| ENSG00000100321.14 | 22 | 39349924 | 39349925 | 0.2847 | rs2069235 | 6.3 | rs2069235 | 0.18 | 10.96 | 6.29878 | 385 | 15 | enet | 0.23 | 5.50E-34 | 6.64884 | 2.95E-11 | 7.76E-08 |

**Supplemental Table S18. The common genes identified by TWAS for hypothyroidism and RA (discovery).**

| **ID** | **TWAS.P_hypothyroidism** | **padj_hypothyroidism** | **TWAS.P_RA (discovery)** | **padj_RA (discovery)** |
| --- | --- | --- | --- | --- |
| ENSG00000224870.6 | 1.00E-08 | 2.73E-06 | 3.33E-04 | 4.32E-02 |
| ENSG00000242485.5 | 4.15E-06 | 4.29E-04 | 5.09E-05 | 1.32E-02 |
| ENSG00000142606.15 | 2.28E-05 | 1.78E-03 | 3.46E-04 | 4.42E-02 |
| ENSG00000215912.12 | 9.97E-08 | 2.09E-05 | 8.15E-06 | 4.93E-03 |
| ENSG00000126698.10 | 6.64E-05 | 4.25E-03 | 2.97E-04 | 4.05E-02 |
| ENSG00000134242.15 | 3.78E-04 | 1.59E-02 | 7.25E-12 | 3.92E-08 |
| ENSG00000118655.4 | 2.37E-28 | 6.37E-25 | 7.71E-37 | 1.25E-32 |
| ENSG00000198821.10 | 3.76E-04 | 1.59E-02 | 6.69E-07 | 1.17E-03 |
| ENSG00000144218.18 | 6.41E-04 | 2.35E-02 | 5.13E-05 | 1.32E-02 |
| ENSG00000135913.10 | 1.37E-03 | 3.96E-02 | 2.52E-04 | 3.65E-02 |
| ENSG00000144580.13 | 1.40E-03 | 4.02E-02 | 2.34E-04 | 3.45E-02 |
| ENSG00000115596.3 | 1.58E-04 | 8.32E-03 | 4.79E-05 | 1.27E-02 |
| ENSG00000026297.15 | 2.78E-29 | 8.96E-26 | 8.58E-06 | 4.93E-03 |
| ENSG00000227598.1 | 5.06E-27 | 9.06E-24 | 4.47E-06 | 3.45E-03 |
| ENSG00000112486.15 | 5.92E-12 | 3.41E-09 | 4.40E-06 | 3.45E-03 |
| ENSG00000128604.19 | 8.49E-04 | 2.91E-02 | 2.98E-08 | 9.67E-05 |
| ENSG00000230359.5 | 3.81E-05 | 2.68E-03 | 6.14E-08 | 1.42E-04 |
| ENSG00000095261.13 | 4.63E-07 | 7.54E-05 | 1.02E-04 | 2.04E-02 |
| ENSG00000119403.13 | 1.26E-06 | 1.61E-04 | 9.87E-05 | 2.03E-02 |
| ENSG00000119396.10 | 2.10E-05 | 1.68E-03 | 1.32E-04 | 2.61E-02 |
| ENSG00000135414.9 | 3.59E-09 | 1.05E-06 | 8.17E-05 | 1.77E-02 |
| ENSG00000139531.12 | 2.09E-13 | 1.58E-10 | 3.98E-05 | 1.15E-02 |
| ENSG00000197728.9 | 1.80E-14 | 1.53E-11 | 2.36E-04 | 3.45E-02 |
| ENSG00000170515.13 | 2.76E-06 | 3.03E-04 | 5.97E-08 | 1.42E-04 |
| ENSG00000135482.6 | 3.87E-06 | 4.05E-04 | 2.79E-04 | 3.94E-02 |
| ENSG00000139641.12 | 2.06E-06 | 2.41E-04 | 1.66E-04 | 2.99E-02 |
| ENSG00000089009.15 | 4.88E-17 | 6.55E-14 | 2.73E-05 | 8.52E-03 |
| ENSG00000182185.18 | 1.04E-03 | 3.30E-02 | 1.81E-04 | 3.12E-02 |
| ENSG00000119718.10 | 6.74E-04 | 2.43E-02 | 3.14E-04 | 4.18E-02 |
| ENSG00000033627.16 | 9.71E-04 | 3.17E-02 | 1.62E-04 | 2.97E-02 |
| ENSG00000264920.1 | 3.13E-04 | 1.37E-02 | 1.00E-04 | 2.03E-02 |
| ENSG00000267654.1 | 1.82E-07 | 3.41E-05 | 3.91E-04 | 4.72E-02 |
| ENSG00000105397.13 | 1.10E-10 | 5.37E-08 | 7.14E-06 | 4.83E-03 |
| ENSG00000254858.9 | 2.03E-06 | 2.41E-04 | 6.59E-06 | 4.78E-03 |
| ENSG00000130518.16 | 2.45E-07 | 4.29E-05 | 1.90E-04 | 3.21E-02 |
| ENSG00000100316.15 | 6.38E-07 | 9.61E-05 | 6.69E-05 | 1.55E-02 |
| ENSG00000100321.14 | 4.31E-08 | 1.01E-05 | 1.33E-06 | 1.46E-03 |

RA: rheumatoid arthritis.

**Supplemental Table S19. The common genes identified by TWAS for hypothyroidism and RA (replication)**

| **ID** | **TWAS.P_hypothyroidism** | **padj_hypothyroidism** | **TWAS.P_RA (replication)** | **padj_RA (replication)** |
| --- | --- | --- | --- | --- |
| ENSG00000157870.14 | 1.89E-05 | 1.55E-03 | 1.49E-05 | 3.67E-03 |
| ENSG00000142606.15 | 2.28E-05 | 1.78E-03 | 3.18E-05 | 6.75E-03 |
| ENSG00000215912.12 | 9.97E-08 | 2.09E-05 | 1.98E-07 | 1.04E-04 |
| ENSG00000204084.12 | 1.11E-11 | 6.17E-09 | 6.30E-07 | 2.57E-04 |
| ENSG00000081985.10 | 2.31E-08 | 5.89E-06 | 3.58E-05 | 7.24E-03 |
| ENSG00000134242.15 | 3.78E-04 | 1.59E-02 | 1.51E-11 | 5.96E-08 |
| ENSG00000160856.20 | 5.17E-07 | 8.09E-05 | 1.20E-05 | 3.05E-03 |
| ENSG00000163534.14 | 4.89E-04 | 1.95E-02 | 2.07E-05 | 4.78E-03 |
| ENSG00000228486.10 | 1.08E-04 | 6.22E-03 | 5.99E-05 | 1.12E-02 |
| ENSG00000144218.18 | 6.41E-04 | 2.35E-02 | 2.67E-08 | 2.34E-05 |
| ENSG00000145723.16 | 4.67E-06 | 4.71E-04 | 9.59E-05 | 1.64E-02 |
| ENSG00000112486.15 | 5.92E-12 | 3.41E-09 | 4.41E-10 | 8.70E-07 |
| ENSG00000128604.19 | 8.49E-04 | 2.91E-02 | 1.40E-08 | 1.84E-05 |
| ENSG00000095261.13 | 4.63E-07 | 7.54E-05 | 1.85E-04 | 2.65E-02 |
| ENSG00000119403.13 | 1.26E-06 | 1.61E-04 | 2.80E-07 | 1.30E-04 |
| ENSG00000134825.15 | 1.32E-09 | 4.17E-07 | 1.87E-06 | 6.41E-04 |
| ENSG00000134824.13 | 2.81E-07 | 4.80E-05 | 6.51E-07 | 2.57E-04 |
| ENSG00000149485.18 | 3.59E-10 | 1.35E-07 | 5.31E-06 | 1.55E-03 |
| ENSG00000139531.12 | 2.09E-13 | 1.58E-10 | 8.19E-08 | 5.87E-05 |
| ENSG00000197728.9 | 1.80E-14 | 1.53E-11 | 4.16E-07 | 1.82E-04 |
| ENSG00000183604.14 | 1.30E-03 | 3.85E-02 | 3.16E-04 | 3.78E-02 |
| ENSG00000267654.1 | 1.82E-07 | 3.41E-05 | 1.08E-06 | 3.87E-04 |
| ENSG00000260302.1 | 9.83E-06 | 9.05E-04 | 3.32E-06 | 1.05E-03 |
| ENSG00000105397.13 | 1.10E-10 | 5.37E-08 | 3.85E-12 | 3.04E-08 |
| ENSG00000160185.14 | 2.71E-06 | 2.99E-04 | 9.80E-09 | 1.55E-05 |
| ENSG00000185651.14 | 3.92E-04 | 1.62E-02 | 2.12E-05 | 4.78E-03 |
| ENSG00000100321.14 | 4.31E-08 | 1.01E-05 | 2.95E-11 | 7.76E-08 |

RA: rheumatoid arthritis
